# A loop-counting method for covariate-corrected low-rank biclustering of gene-expression and genome-wide association study data

**DOI:** 10.1371/journal.pcbi.1006105

**Published:** 2018-05-14

**Authors:** Aaditya V. Rangan, Caroline C. McGrouther, John Kelsoe, Nicholas Schork, Eli Stahl, Qian Zhu, Arjun Krishnan, Vicky Yao, Olga Troyanskaya, Seda Bilaloglu, Preeti Raghavan, Sarah Bergen, Anders Jureus, Mikael Landen, Bipolar Disorders Working Group of the Psychiatric Genomics Consortium

**Affiliations:** 1 Mathematics, New York University, New York, New York, United States of America; 2 Center for Computational Biology, Flatiron Institute, New York, New York, United States of America; 3 Psychiatry, University of California, San Diego, California, United States of America; 4 Human Biology, J. Craig Venters Institute, La Jolla, California, United States of America; 5 Genetics and Genomic Sciences, Mount Sinai Medical School, New York, New York, United States of America; 6 Computer Science, Princeton University, Princeton, New Jersey, United States of America; 7 Computational Mathematics Science and Engineering, Michigan State University, East Lansing, Michigan, United States of America; 8 Department of Rehabilitation Medicine, New York University Medical School, New York, New York, United States of America; 9 Department of Medical Epidemiology and Biostatistics, Karolinska Institutet, Stockholm, Sweden; 10 Physiology and Biophysics, University of Gothenburg, Gothenburg, Sweden; Microsoft Research, UNITED STATES

## Abstract

A common goal in data-analysis is to sift through a large data-matrix and detect any significant submatrices (i.e., biclusters) that have a low numerical rank. We present a simple algorithm for tackling this biclustering problem. Our algorithm accumulates information about 2-by-2 submatrices (i.e., ‘loops’) within the data-matrix, and focuses on rows and columns of the data-matrix that participate in an abundance of low-rank loops. We demonstrate, through analysis and numerical-experiments, that this loop-counting method performs well in a variety of scenarios, outperforming simple spectral methods in many situations of interest. Another important feature of our method is that it can easily be modified to account for aspects of experimental design which commonly arise in practice. For example, our algorithm can be modified to correct for controls, categorical- and continuous-covariates, as well as sparsity within the data. We demonstrate these practical features with two examples; the first drawn from gene-expression analysis and the second drawn from a much larger genome-wide-association-study (GWAS).

## Author summary

An important problem in genomics is how to detect the genetic signatures associated with disease. When a disease is caused by a single well-defined biological mechanism, the genetic signature often involves a handful of genes present across the majority of the diseased patients. On the other hand, when a disease is complicated or poorly understood there may be many possible biological mechanisms at play. Any genetic signatures associated with such a disease may involve multiple genes, and each signature might only manifest across a subset of the diseased patients. Finding the signatures responsible for this heterogeneity requires searching through subsets of genes *and* subsets of patients—a problem referred to as ‘biclustering’. In this paper we present a new biclustering method which can scale up efficiently to handle large genomic data sets, such as GWAS-data. Our method is quite accurate, outperforming current ‘spectral’ biclustering methods for many problems of interest. Perhaps most importantly, our method can be corrected for many features of experimental design, such as controls, covariates and sparsity—all of which are especially important when analyzing real data sets.

## Introduction

Many applications in data-analysis involve some form of ‘biclustering’—also referred to as co-clustering, two-mode clustering, two-way clustering, block clustering, and coupled two-way clustering, to name a few (see, e.g., [1–5]). Broadly speaking, the goal of biclustering is to search through a large data-array and reveal components that have special structure. Typically, these structured components involve only a subset of the rows and columns in the data-array, and finding them can be rather difficult (i.e., biclustering is NP-complete [6]). Because this problem is so general, it should come as no surprise that there are many different kinds of biclustering algorithms developed for a variety of applications, ranging from political science to neuroscience [7, 8]. Despite the abundance of biclustering methods in the literature, many existing biclustering methods are too computationally intensive to be successfully applied to the large data-sets that arise in genomics.

In this paper we’ll present a simple method for biclustering which is well-suited for gene-expression and genome-wide-association-study (GWAS) data. We refer to our method as ‘loop-counting’, because it accumulates information about 2 × 2 submatrices within the data-array; we refer to these 2 × 2 submatrices as ‘loops’. We establish a connection between our loop-counting method and spectral methods, using both analysis and simulation-studies to describe the regimes where our method performs well. We demonstrate the efficacy of our loop-counting method by applying it to a gene-expression data-set and a GWAS data-set, using gene-enrichment analysis as a form of validation.

One of our main goals is to ensure that our loop-counting method is practical and capable of accommodating the issues that arise when analyzing real experimental data. We emphasize that our method can be corrected for many features of experimental design, such as controls, covariates and sparsity—all of which are especially important when analyzing GWAS data sets (which commonly aggregate batches of patients taken from across the world). With this goal in mind, we’ll briefly delineate some features of our approach, contrasting them against some of the better-known methods for biclustering this kind of data.

During our discussion below we’ll often refer to the appendices in the Supporting Information (see S1 and S2 Text), which will be denoted with an ‘A’ before the section number.

### Some other approaches to biclustering

Within the field of bioinformatics there are a variety of approaches to biclustering, many of which differ in the types of structures they try and find, their assumptions regarding the ‘noise’ that might obscure these structured elements, and their goals in classifying the data (for review, see [3–5, 8–12]). To frame the discussion for now, we’ll imagine an *M* × *N* data-array *D* comprising *N* genetic-measurements (which we’ll refer to as ‘genes’) taken across *M* patients. Within this context, an *m* × *n* ‘bicluster’ would correspond to a rectangular submatrix associated with *m* patients and *n* genes that—together—share a special structure.

**Fig 1:**
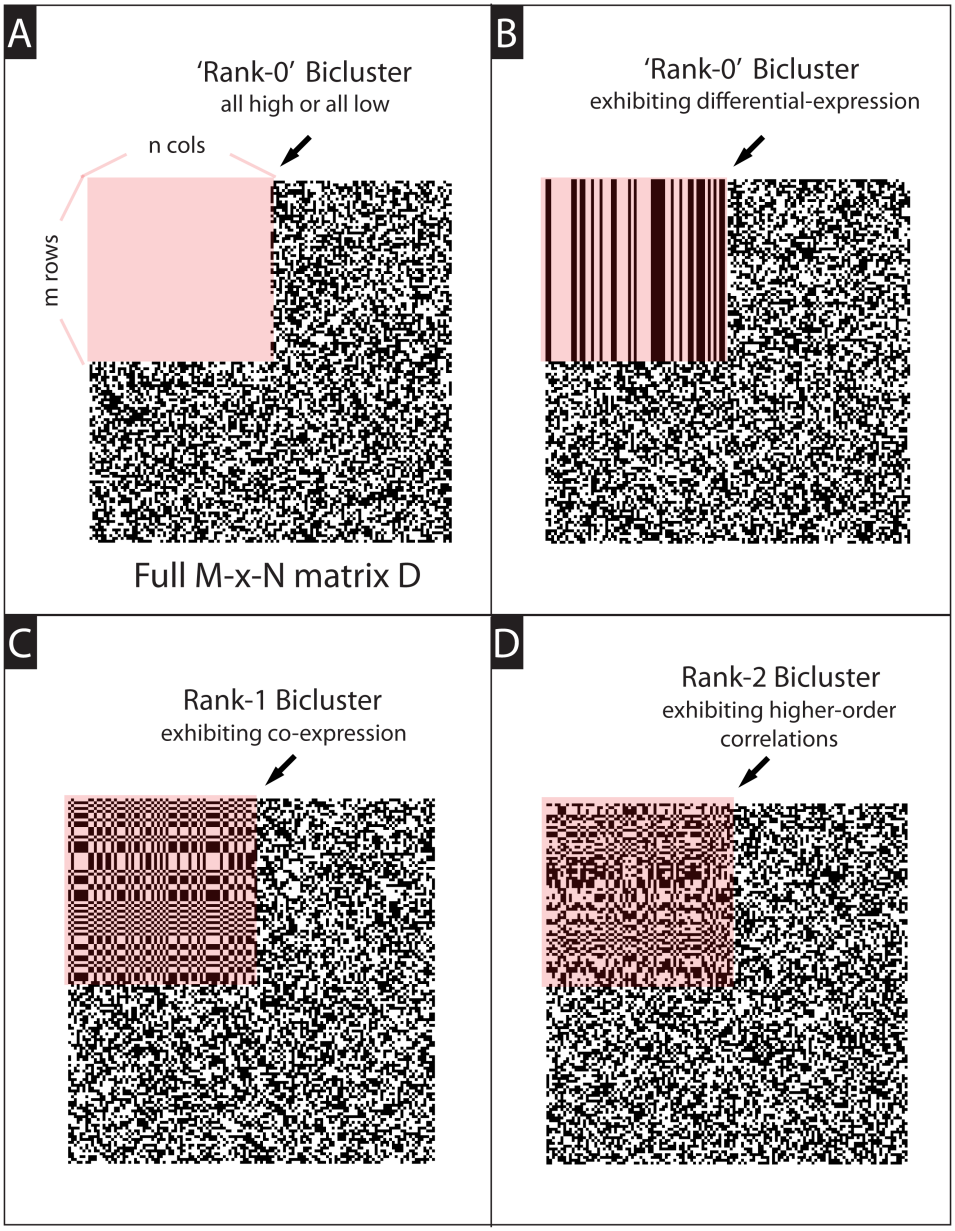
A highly idealized cartoon of different kinds of biclusters. In each panel we show a heat-map of an *M* × *N* matrix ‘*D*’, which contains a large embedded bicluster (highlighted in pink) with a special structure. In this cartoon, light and dark pixels correspond to high and low values for the corresponding matrix-entry. Many approaches to biclustering search for structures containing mostly ‘large’ or ‘small’ values—as shown in Panels A and B. Such a bicluster can be thought of as delineating a subset of columns which are ‘differentially-expressed’ with respect to the remaining rows of *D*. Our algorithm generalizes this notion, searching for biclusters that are ‘low-rank’. Examples of low-rank biclusters include those shown in Panels A and B, as well as ‘rank-1’ biclusters which can exhibit co-expression without necessarily exhibiting differential-expression (see Panel-C and Fig 5 later on). Also encompassed are ‘rank-2’ and higher biclusters which exhibit higher-order correlations that are not necessarily obvious to the eye (see Panel-D and Fig 7 later on). Note that, while the biclusters shown in this cartoon are very large and essentially noiseless, our algorithm can readily discover biclusters that are much smaller and noisier (see section A5 of S1 Text).

**Types of structure to search for:** Several methods attempt to find biclusters containing mostly ‘large’ or ‘small’ values; i.e., comprising a subset of genes which are differentially-expressed relative to the rest of the patients in *D* [2, 13–21]. While there are many situations where these kinds of differentially-expressed structures do indeed exist, we prefer to generalize this notion somewhat. Our loop-counting method can find not only biclusters consisting of differentially-expressed genes, but also biclusters consisting of highly correlated genes. Mathematically speaking, our method is well suited to detect biclusters that are ‘numerically-low-rank’ (i.e., that have a spectrum of singular values that decays relatively quickly). These low-rank biclusters include (but are not limited to) the differentially-expressed structures mentioned above; also including structures that exhibit co-expression without differential-expression (see Fig 1). We believe that, by searching for these more general low-rank structures, we can expose many kinds of co-regulation in gene-expression data, as well as certain epistatic interactions in GWAS data. Other methods which also attempt to locate low-rank biclusters include [22–26].
**Assumptions regarding the noise:** Some methods assume that the data-array *D* is itself composed of various biclusters along with additive noise [16, 27–30]. While the additive noise model is reasonable in many contexts (e.g., experimentally induced noise in gene-expression experiments), we believe that it may be too restrictive in others. For example, additive-noise is not directly compatible with the genotyped-data of GWAS, which is typically discrete in nature. For this reason we assume instead that the data-array *D* may have some correlated structures implanted within it, with the remainder drawn from a less correlated distribution. Thus, we allow for certain portions of *D* to be more tightly correlated than others, without necessarily containing entries that are large in magnitude. These assumptions are not too different from assuming additive noise within the empirical covariance of the data [31, 32], and are similar to the assumptions used to define the ‘planted-clique’ and ‘planted-biclique’ problems in theoretical computer science, playing a role in the methodology of [15, 33–35].
**Goals when classifying the data:** Some methods attempt to categorize the entire data-array *D*, placing each row and column into one or more biclusters (perhaps disjoint, or perhaps overlapping) [20, 21, 36]. Other methods assume that much of the data might be noisy, messy or unstructured, and only try to find a few biclusters that might be statistically significant [13, 18, 27, 30]. Our loop-counting method adopts an even less ambitous perspective: there is often no reason to suspect a-priori that the data-set contains even a single well-defined bicluster. Moreover, as we argue in sections A7.3 and A14.3 of S1 and S2 Text, there are plenty of structures within real data-sets that cannot be fully captured with a single rectangular submatrix. Therefore, our algorithm attempts to produce a useful ranking of the rows and columns that will expose structures of interest, revealing the largest/strongest bicluster if possible. After delineating and extracting this dominant structure, we can find additional structure by rerunning our algorithm. Another method which adopts a similar perspective is the ‘LAS’-method of [18, 28, 29].

Along with the aforementioned considerations, we have tried to design our methodology so that we don’t lean too heavily on any single assumption. For example, our loop-counting method often functions sensibly even in situations where the hidden biclusters are mostly large or small; when the noise is additive, correlated, or heterogeneous; or when there are multiple overlapping structures to be found.

### Accounting for experimental design

While our method certainly isn’t the fastest, most accurate or most sensitive method possible, we believe that it is robust enough to deal with many of the vagaries of real data, and that it can be applied to many real problems in bioinformatics. To this end, we have designed our method to account for the following features of experimental design which commonly arise when analyzing genomic data:

**Cases-versus-Controls:** Some patients may suffer from a certain disease (i.e., ‘cases’) while others do not. By correcting for controls we can search for correlated structures that are limited to the case-population. These case-specific structures may be useful for clinical diagnosis or for revealing disease mechanisms.
**Categorical- and continuous-covariates:** Often patients come from different studies, or are measured with different machines. Each patient may also be associated with a vector of continuous-covariates (e.g., a vector of mds-components correlated with genetic ancestry [37, 38]). It is often critical to correct for the influence of these covariates when looking for significant patterns.
**Sparsity:** In certain circumstances (e.g., when dealing with genotyped data) the data-matrix can be sparse. Moreover, different columns of the data-matrix can have different sparsity-coefficients (e.g., different minor-allele-frequencies). It is typically important to take this sparsity into account when determining which patterns are significant and which are not.

### Practical features

In addition to accounting for experimental-design, our loop-counting method also has the following practical features:

**Few to no parameters:** Aside from a parameter ‘*I*_req_’ which specifies what it means to correct for categorical-covariates (see section A9 of S1 Text), our method has essentially no free parameters which need to be specified by the user.
**Scales to GWAS-data:** Our method scales up well, and can be used to analyze large GWAS data-sets containing ≳ 10^4^ patients and ≳ 10^5^ SNPs with a total computation time ranging from a few hours to a few days (depending on the range of minor-allele-frequencies and the number of covariates involved).
**Provides a p-value:** In addition to searching for biclusters, our methodology provides p-values for whatever structures are found via a permutation test compatible with the experimental-design (e.g., respecting the covariate structure).
**Statistical guarantees:** While not immediately relevant to real data, our method has provable performance guarantees when applied to the (idealized) ‘planted-bicluster’ problem. While our method is certainly not ‘optimal’ for this idealized problem, we do outperform simple spectral biclustering schemes for many regimes of interest.

In the following sections we’ll describe our algorithm, sketch out the analytical intuition associated with its performance, and present some examples applied to real gene-expression and GWAS data. As mentioned above, references to S1 and S2 Text will be have an ‘A’-prefix before the section number.

## Results

As alluded to in the introduction, we have tried to ensure that our loop-counting method is useful in practice; i.e., it can be applied to real data-sets in a reasonable amount of time, while accounting for experimental-design and providing a p-value to assess statistical-significance. In the following subsections we give a brief overview of our algorithm. To reiterate, our method accumulates information about ‘loops’ (i.e., 2 × 2 submatrices) within the data-array. We start out with the simplest possible situation and explain when we expect our algorithm to work. Then we compare the performance of our algorithm to a related algorithm—namely a simple spectral method; this comparison allows us to discuss the relationship between our method and the more sophisticated message-passing algorithms in the literature. Afterwards, we explain how to generalize our algorithm to incorporate controls, covariates and sparse data. Finally, we present some examples taken from gene-expression and GWAS data, and comment on some practical considerations, such as finding p-values for a bicluster and delineating the boundaries of a bicluster.

### Simple case: *D* only

In the simplest situation there are no controls, covariates, or sparsity considerations, and we are tasked with exposing low-rank structures within an *M* × *N* case-matrix *D*. In this case our loop-counting algorithm reduces to the following very simple iteration, described earlier in [39] and in more detail within sections A2 and A3 of S1 Text:

Step 0 Binarize *D*, sending each entry to either +1 or −1, depending on its sign (i.e., *D* = sign(*D*));
Step 1 Calculate ‘loop-scores’ for each row and column. In their simplest form the loop-scores for each row are given by the diagonal entries:
  $$\begin{array}{c} Z_{\text{ROW}}=\text{diag}(DD^{⊺}DD^{⊺})\text{,} \end{array}$$ (1)
  and the loop-scores for each column are given by the diagonal entries:
  $$\begin{array}{c} Z_{\text{COL}}=\text{diag}(D^{⊺}DD^{⊺}D)\text{;} \end{array}$$ (2)
Step 2 Restrict attention to the row-indices for which *Z*_ROW_ is highest (i.e., most positive) and the column indices for which *Z*_COL_ is highest—e.g., throw away the rows/columns for which *Z*_ROW_ and *Z*_COL_ are lowest (i.e., most negative).
Step 3 Go back to step 1.

Note that our algorithm is iterative; Steps 1 and 2 involve repeatedly recalculating scores and eliminating portions of the data-array *D* (this recalculation can be done efficiently using a low-rank update, as discussed in section A3.1 of S1 Text). Eventually, after repeating this process multiple times, we will eliminate almost all the rows and columns of *D*. As a consequence of this simple iteration, the output of the algorithm is a listing of row- and column-indices in the order that they were eliminated. If there were indeed a low-rank bicluster hiding within *D*, then (assuming certain criteria are satisfied) our algorithm will usually find it; retaining the rows and columns of the bicluster until the end (see section A5 of S1 Text). After finding the first bicluster in this manner, the entries of *D* corresponding to this first bicluster can be scrambled (i.e., destroying their low-rank structure), and the next bicluster can be found by running the algorithm again (see section A14.3 of S2 Text).

**Fig 2:**
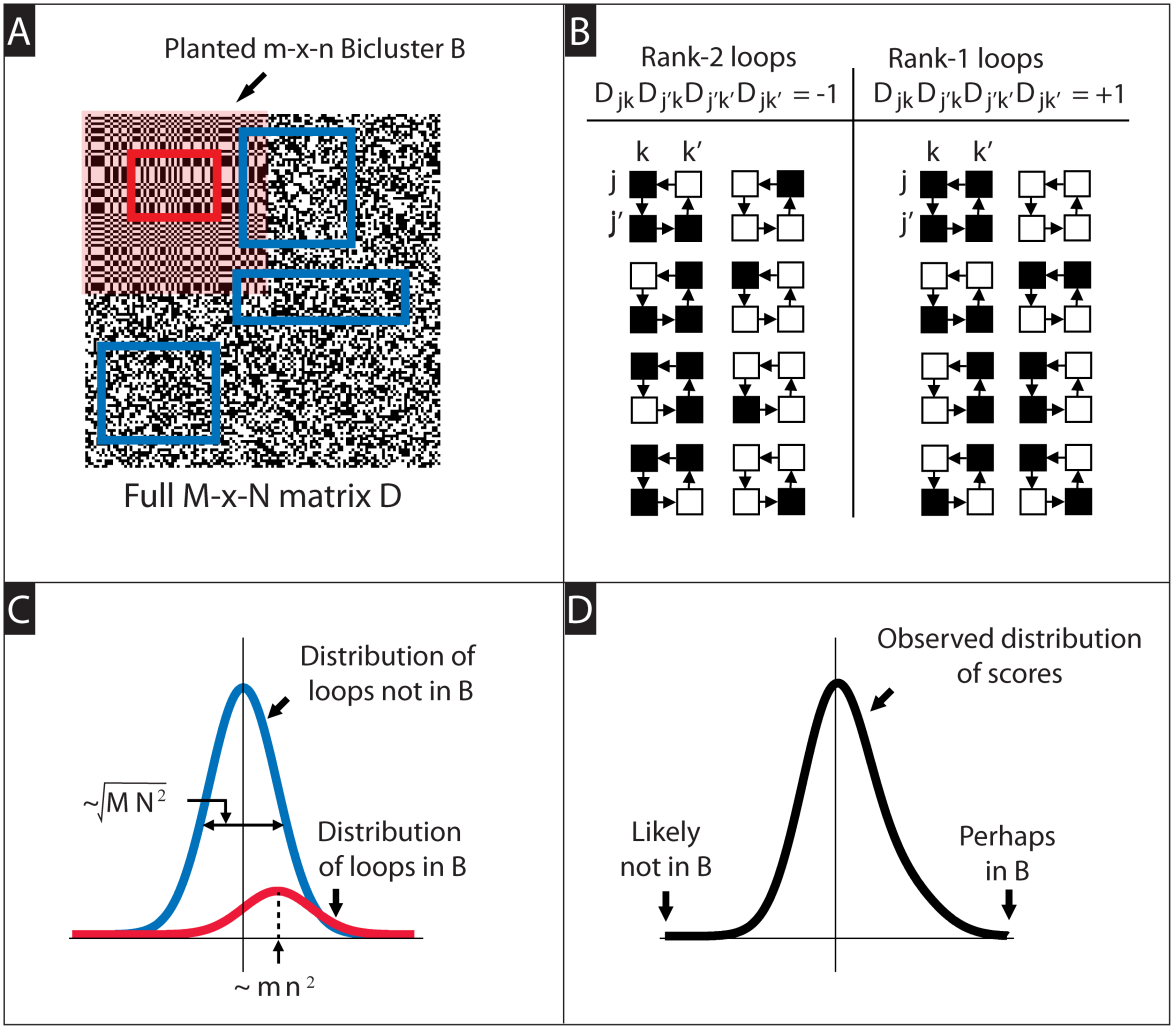
Illustration of the algorithm operating on a case-matrix alone (i.e., *D* only). In Panel-A we show a large *M* × *N* binarized matrix *D* (black and white pixels correspond to values of ±1, respectively). In the upper left corner of *D* we’ve inserted a large rank-1 bicluster *B* (shaded in pink). Our algorithm considers all 2 × 2 submatrices (i.e., ‘loops’) within *D*. Several such loops are highlighted via the blue rectangles (the corners of each rectangle pick out a 2 × 2 submatrix). Generally speaking, loops are equally likely to be rank-1 or rank-2. Some loops, such as the loop shown in red, are entirely contained within *B*. These loops are more likely to be rank-1 than rank-2. In Panel-B we show some examples of rank-2 and rank-1 loops. Given a loop with row-indices *j*, *j*′ and column-indices *k*, *k*′, the rank of the loop is determined by the sign of $D_{jk}D_{kj^{\prime }}^{⊺}D_{j^{\prime }k^{\prime }}D_{k^{\prime }j}^{⊺}$. Our algorithm accumulates a ‘loop-score’ for each row *j* and each column *k*. In its simplest form, the loop-score for a particular row *j* is given by $\Sigma_{j^{\prime },k,k^{\prime }}D_{jk}D_{kj^{\prime }}^{⊺}D_{j^{\prime }k^{\prime }}D_{k^{\prime }j}^{⊺}={\left[DD^{⊺}DD^{⊺}\right]}_{jj}$. Analogously, the loop-score for a column *k* is given by ${\left[D^{⊺}DD^{⊺}D\right]}_{kk}$. In Panel-C we show the distribution of loop-scores we might expect from the rows or columns within *D*. The blue-curve corresponds to the distribution of scores expected from the rows/cols of *D* that are not in *B*, whereas the red-curve corresponds to the distribution of scores expected from the rows/cols of *B*. In Panel-D we show the distribution of loop-scores we might expect by pooling all rows or columns of *D*. The rows or columns that correspond to the lowest scores are not likely to be part of *B*.

This algorithm can be understood in terms of loops (i.e., 2 × 2-submatrices) of the data-matrix *D*; each loop involves up to two rows and two columns of *D* (see Fig 2). The row-scores [*Z*_ROW_]_*j*_ accumulate a sum over all loops that intersect row-*j* of *D*. This sum contributes a ‘+1’ for each loop that is rank-1, and a ‘−1’ for each loop that is not rank-1. Similarly, the column-score [*Z*_COL_]_*k*_ tallies the ranks of the loops intersecting column-*k* of *D*. As explained in section A4 of S1 Text, these loop-scores contain ‘signals’ driven by the various structured elements within the data-matrix *D*. Specifically, if *D* contains an *m* × *n* bicluster *B* of low numerical-rank spanning rows *J*_*B*_ and columns *K*_*B*_, then *B* will add ∼ *mn*^2^ to the row-scores of *j* ∈ *J*_*B*_, and ∼ *m*^2^*n* to the column-scores of *k* ∈ *K*_*B*_.

The loop-scores contain these signals because of the following fact regarding high dimensional space: a random planar projection of an eccentric gaussian-distribution is typically concentrated in non-adjacent quadrants. Put in more colloquial terms, one can imagine flipping a dowel (numerical-rank 1) or a discus (numerical-rank-2) into the air so that it casts a shadow centered on the origin of a two-dimensional plane. While it is certainly possible for the shadow to be cast equally across all four quadrants, it is much more likely that the shadow will fall mostly into opposite quadrants (see Fig A21 in section A5 of S1 Text). This fact implies that the loops of *D* contain substantial information about biclusters within *D*.

As explained in section A5 of S1 Text, there are many situations where we can quantify when our loop-counting algorithm will work. One example includes the ‘planted-bicluster’ problem (section A6 of S1 Text). In this scenario we consider a large *M* × *M* data-matrix matrix *D* with entries chosen independently from a distribution with median 0. After creating *D* we’ll embed within *D* an *m* × *m* submatrix *B*, which is rank-l with ‘spectral-error’ *ε* (i.e., the first *l* singular-values of *B* are equally large, but the (*l* + 1)^st^ singular-value of *B* is *ε* times the first singular-value—see section A5.1 of S1 Text). We’ll assume that *M* ≫ *m* ≫ 1. Given *B*, we can derive an equation for the probability *g*_*l*,*ε*,*m*_ for a loop in *B* to be rank-1 rather than rank-2. Given *g*_*l*,*ε*,*m*_, our loop-counting algorithm will detect *B* with high probability whenever $m^{3}\left(2g_{l,\varepsilon ,m}-1\right)≳\sqrt{2M^{3}}$. This means that, when $\varepsilon \sqrt{m}\ll 1$, our algorithm should work well if $m≳\sqrt{M}$. In addition, if $\varepsilon \sqrt{m}∼1$, then our algorithm should work well if $m≳\sqrt[{3}]{\pi^{2}/4}\cdot {\left[\varepsilon \sqrt{m}\right]}^{4/3}\cdot \sqrt{M}$.

**Fig 3:**
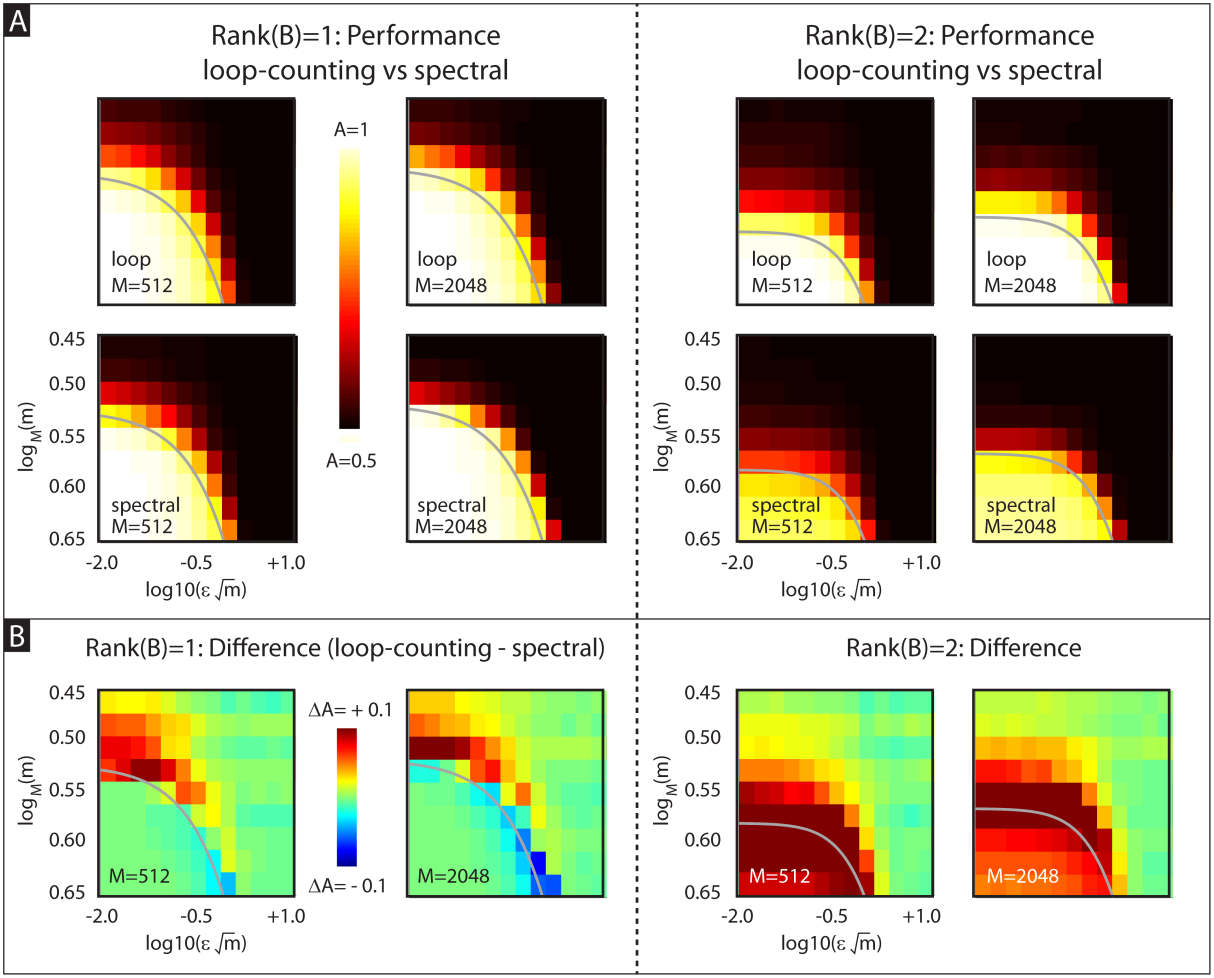
Performance of loop-scores vs spectral-biclustering applied to the planted-bicluster problem. For each instantiation of the planted-bicluster problem we choose an *M*, *m*, *ε* and *l*; we use these parameters to generate a random *M* × *M* matrix *D* and embedded *m* × *m* rank-*l* submatrix *B* with spectral noise *ε*. For each instantiation, our algorithm produces a list of row- and column-indices of *D* in the order in which they are eliminated; those rows and columns retained the longest are expected to be members of *B*. To assess the success of our algorithm we calculate the auc *A*_*R*_ (i.e., area under the receiver operator characteristic curve) associated with the row-indices of *B* with respect to the output list from our algorithm. The value *A*_*R*_ is equal to the probability that: given a randomly chosen row from *B* as well as a randomly chosen row from outside of *B*, our algorithm eliminates the latter before the former (i.e., the latter is lower on our list than the former); We calculate the auc *A*_*C*_ for the columns similarly. Finally, we use *A* = (*A*_*R*_ + *A*_*C*_)/2 as a metric of success; values of *A* near 1 mean that the rows and columns of *B* were filtered to the top by our algorithm, whereas values of *A* near 0.5 mean that our algorithm failed to detect *B*. In the top of Panel-A we show the trial-averaged auc *A* for our loop-counting method as a function of $\varepsilon \sqrt{m}$ and log_*M*_ (*m*). Results for *l* = 1 are shown on the left; *l* = 2 is shown on the right. Each subplot takes the form of a heatmap, with each pixel showing the value of *A* for a given value of ${\text{log}}_{10}\left(\varepsilon \sqrt{m}\right)$ and log_*M*_ (*m*) (averaged over at least 128 trials). The different subplots correspond to different values for *M*. Note that our loop-counting algorithm is generally successful when ${\text{log}}_{10}\left(\varepsilon \sqrt{m}\right)≲0$ and ${\text{log}}_{M}\left(m\right)≳0.5$. In the bottom of Panel-A we show the analogous auc *A* for a simple implementation of the spectral method (see section A22 of S2 Text). In Panel-B we show the difference in trial-averaged *A* between these two methods (see colorbar for scale). Note that when *l* ≥ 2 or the noise is small, our loop-score generally has a higher rate of success than the spectral method. On the other hand, there do exist parameters when *l* = 1 and $\varepsilon \sqrt{m}∼1$ where the spectral method has a higher rate of success. In each panel the thin grey line shows the detection-boundary for our loop-counting method (calculated using $m^{3}\left(2g_{l,\varepsilon ,m}-1\right)=\sqrt{2M^{3}}$).

Numerical experiments corroborating this analysis are shown in Fig 3A. These numerical experiments illustrate the performance of our algorithm on the planted-bicluster problem described above, with the rank fixed at *l* = 1 on the left and *l* = 2 on the right. See Figs A29, A30, and A31 in S1 Text for slightly more detail, as well as the *l* = 3 case. A more detailed discussion of our loop-counting algorithm (specifically, motivation for the binarization, loop-counting, and iteration steps) is found in section A7 of S1 Text. A comparison between our loop-counting method and some publicly available implementations of other biclustering methods is found in section A7.4 of S1 Text.

### Comparison with a simple spectral method

As we mentioned earlier, the loop-score associated with each row (or column) tallies the ranks of all loops from that row (or column) to itself. One natural generalization of this notion is to consider not merely loops—which are paths of length 4—but longer paths of length 2*d*+2 through the data-matrix *D*. Specifically, we can define ‘*d*-scores’—denoted as $Z_{\text{ROW}}^{d}$ and $Z_{\text{COL}}^{d}$—using the diagonal entries diag (*DD*^⊺^)^*d*+1^ and diag (*D*^⊺^*D*)^*d*+1^, respectively. The *d*-score associated with any row (or column) tallies the ranks of all 2*d* + 2-step paths leading from that row (or column) to itself. Note that as *d* → ∞, these *d*-scores converge in direction to the entrywise-square of the dominant singular vectors of *D*.

These observations motivate the following simple spectral-biclustering method, which is closely related to the spectral methods of [22, 40]:

Step 0 Binarize *D*, sending each entry to either +1 or −1 (i.e., *D* = sign(*D*) or *D* = 2(*D* > 0) − 1).
Step 1 Calculate the singular-value-decomposition *D* = *U*Σ*V*^⊺^. Set $\vec{u}$ and $\vec{v}$ to be the first columns of *U* and *V*.
Step 2 Set ${\left[Z_{\text{ROW}}\right]}_{j}={\vec{u}}_{j}^{2}$ and ${\left[Z_{\text{COL}}\right]}_{k}={\vec{v}}_{k}^{2}$ to be the entrywise-squares of the singular-vectors $\vec{u}$ and $\vec{v}$.
Step 3 Use *Z*_ROW_ and *Z*_COL_ to produce a ranked list of rows and columns of *D*.

When considering the planted-bicluster problem, this simple spectral method has detection-thresholds which are similar to those of the loop-counting method (see section A22 of S2 Text). However, as shown in Fig 3B, our loop-counting method often outperforms the simple spectral method, particularly when the implanted bicluster is rank *l* > 1.

This phenomenon has to do with the asymptotic behavior of the singular-vectors of *D* close to the detection-threshold (see [31, 32]) and is discussed in more detail in section A22.3 of S2 Text. In brief, when *l* > 1 and *m* is close to $\sqrt{M}$, our loop-scores are more useful than the entries of the dominant singular vector with regards to a binary classifier. This is one of the main reasons that we have focused on our loop-counting method, and why we have not pursued spectral methods for biclustering.

**Remark:** Above we’ve introduced a family of *d*-scores, with *d* = 1 corresponding to our loop-scores, and *d* = ∞ corresponding to the simple-spectral scores. As one may expect, for any given planted-bicluster problem the most useful *d*-score is neither the loop-score nor the spectral-score, but one with an intermediate value of *d*. Taking this reasoning further, one might imagine a score that is constructed nonlinearly; instead of building the *d*-score $\left[Z_{\text{ROW}}^{d}\right]$ by applying power-iteration to the covariance-matrix *DD*^⊺^, one might try and build an even better score by applying a nonlinearity in between each stage of the power-iteration.

The message-passing algorithms of [33], [19], and [41] proceed along these lines. Generally speaking, these algorithms choose an appropriate nonlinearity to apply between stages of a ‘message-passing procedure’ similar to power-iteration. By choosing this nonlinearity carefully, these methods can significantly reduce their detection-thresholds for a variety of problems very similar to our planted-bicluster problem (such as, e.g., the planted-clique problem). Given the success of the message-passing algorithms of [33], [19], and [41], it seems certain that there exists a message-passing algorithm that outperforms our loop-counting algorithm when applied to the planted-bicluster problem; we fully intend to pursue this line of research in the future (see the discussion in section A22.4 of S2 Text).

### Incorporating experimental design

While appropriate for idealized situations such as the planted-bicluster problem, the simple loop-counting algorithm described above is not immediately applicable to real data. The reason is that the loop-scores in Eqs 1 and 2 do not take into account experimental design. To tackle this issue we can redefine our scores, ensuring that the structures that generate the largest signals correspond to those that are most relevant.

To describe this strategy in more detail, we’ll first focus on a situation involving cases and controls. Specifically, let’s imagine that we are analyzing gene-expression data where *M*_*D*_ of the patients exhibit a certain disease, whereas the other *M*_*X*_ patients do not (i.e., cases and controls, respectively). Instead of arranging our data into a single *M* × *N* array, we will divide our data into an *M*_*D*_ × *N* array *D* describing the cases, and another *M*_*X*_ × *N* array *X* describing the controls. As is typical for gene-expression data, there will likely exist large subsets of genes which are correlated across large subsets of the population—including both *D* and *X*—without being particularly related to the disease. Such genes might include, e.g., genes related to development, or ‘housekeeping’ genes that are strongly coexpressed in most patients.

If we were to simply search for the *largest* biclusters, we would find these common genetic signatures, which are not related to the disease, and thus not of interest. Instead, we would like to search for biclusters which are restricted to a subset of the case-patient-population in *D* and which exhibit correlations which are *not* found in the control-population *X*. Such case-specific biclusters are more likely to be related to disease mechanisms.

We can achieve this goal by slightly modifying ‘Step-0’ and ‘Step-1’ of the *D*-only algorithm above. In Step-0 we now need to binarize both *D* and *X*. In Step-1 we need to calculate the loop-scores in a slightly different way. Given any fixed case-row *j* ∈ *D*, there will be two types of loops that contribute to the score: (i) loops that are contained within *D*, and (ii) loops that travel through *X*. If row-*j* were part of a bicluster which was restricted to *D*, then we would expect row-*j* to participate in an abundance of rank-1 loops of type-(i), but not of type-(ii). Based on this intuition, we score the first type of loop positively if it is rank-1 and negatively if it is rank-2. In addition, we score the second type of loop the other way; negatively if it is rank-1 and positively if it is rank-2. This strategy produces control-corrected row- and column-scores of the form:

$$\begin{array}{lll} {\left[Z_{\text{ROW}}\right]}_{j} & = & {\left[Z_{\text{ROW}}^{DD}\right]}_{j}-{\left[Z_{\text{ROW}}^{DX}\right]}_{j}\;\text{,}\;\text{where} \end{array}$$

$$\begin{array}{lll} {\left[Z_{\text{ROW}}^{DD}\right]}_{j} & = & \frac{1}{\left(M_{D}-1\right)N\left(N-1\right)}\left\{{\left[DD^{⊺}DD^{⊺}\right]}_{jj}-N\left(N+M_{D}-1\right)\right\}\text{,} \end{array}$$

$$\begin{array}{lll} {\left[Z_{\text{ROW}}^{DX}\right]}_{j} & = & \frac{1}{M_{X}N\left(N-1\right)}\left\{{\left[DX^{⊺}XD^{⊺}\right]}_{jj}-M_{X}N\right\}\text{,}\;\text{and} \end{array}$$

$$\begin{array}{lll} {\left[Z_{\text{COL}}\right]}_{k} & = & {\left[Z_{\text{COL}}^{DD}\right]}_{k}-{\left[Z_{\text{COL}}^{DX}\right]}_{k}\;\text{,}\;\text{where} \end{array}$$

$$\begin{array}{lll} {\left[Z_{\text{COL}}^{DD}\right]}_{k} & = & \frac{1}{\left(N-1\right)M_{D}\left(M_{D}-1\right)}\left\{{\left[D^{⊺}DD^{⊺}D\right]}_{kk}-M_{D}\left(M_{D}+N-1\right)\right\}\text{,} \end{array}$$

$$\begin{array}{lll} {\left[Z_{\text{COL}}^{DX}\right]}_{k} & = & \frac{1}{\left(N-1\right)M_{D}M_{X}}\left\{{\left[D^{⊺}DX^{⊺}X\right]}_{kk}-M_{D}M_{X}\right\}\text{.} \end{array}$$

These control-corrected loop-scores are designed so that biclusters which equally straddle both the cases and the controls will—on average—produce no signal, while biclusters which are significantly concentrated within the cases will still produce a signal. Biclusters which are fully case-specific will generate signals that are (on average) as large as they would have been using our original (uncorrected) loop-scores. A more detailed explanation of these control-corrected loop-scores, as well as corroborating numerical experiments, can be found in sections A8 and A15.2 of S1 and S2 Text.

A similar principle can be used to correct for categorical-covariates, multidimensional continuous-covariates, as well as sparsity. We briefly discuss these corrections in the Methods section, deferring the details to sections A9, A10 and A11 of S1 and S2 Text.

In the following subsections we briefly present two examples drawn from genomics. These examples serve as proofs-of-principle for our method, and demonstrate that our method functions in practice.

### Example-A: Gene expression analysis

Our first example is taken from the GSE48091 data-set available from the gene-expression-omnibus (found at http://www.ncbi.nlm.nih.gov/geo/query/acc.cgi?acc=GSE48091). See ‘Example-1b’ in section A1.2 of S1 Text for more details regarding this example.

**Fig 4:**
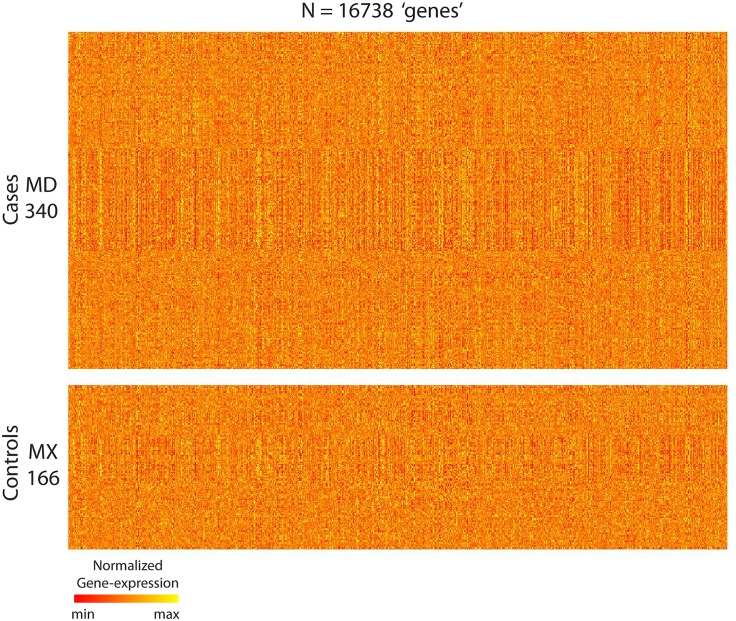
Illustration of the GSE48091 gene-expression data-set used in Example-A (see main text). Each row corresponds to a patient, and each column to a ‘gene’ (i.e., gene-expression measurement): the color of each pixel codes for the intensity of a particular measurement of a particular patient (see colorbar to the bottom).*M*_*D*_ = 340 of these patients are cases, the other *M*_*X*_ = 166 are controls; we group the former into the case-matrix ‘*D*’, and the latter into the control-matrix ‘*X*’.

The subset of data that we use comprises *N* = 16738 gene-expression measurements (referred to later as ‘genes’ for simplicity) collected across 506 patients, each diagnosed with breast-cancer. Of these patients, *M*_*D*_ = 340 were ‘case’-patients that developed distant metastatic disease. The remaining *M*_*X*_ = 166 were ‘control’-patients that did not. The data-set is illustrated in Fig 4, with each gene normalized to have median 0 across the patient-population.

We’ll use our control-corrected loop-counting algorithm to search this data-set for case-specific biclusters—namely subsets of genes that are structured in some way across a significantly large subset of the case-patients, while not being similarly structured across the control-population. These case-specific biclusters will have the potential to pinpoint genes useful for diagnosis and discrimination between case and control status.

**Fig 5:**
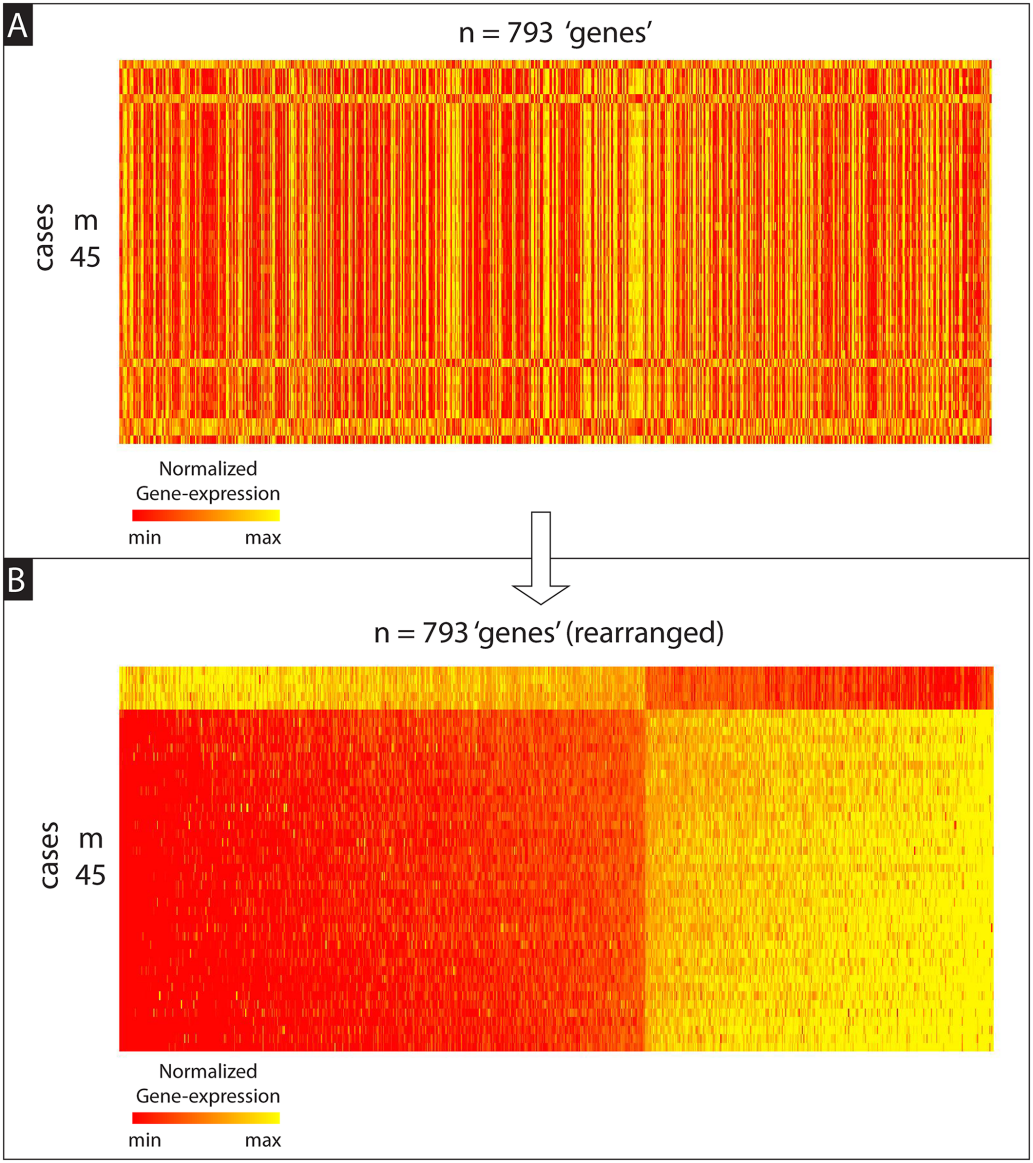
Illustration of bicluster found within gene-expression data-set. Both panels illustrate the same submatrix (i.e., bicluster) drawn from the full case-matrix shown at the top of Fig 4. This bicluster was found using our control-corrected biclustering algorithm (described in section A8 of S1 Text). In Panel-A we represent this bicluster using the row- and column-ordering given by the output of our algorithm. This ordering has certain advantages (see section A14 of S2 Text), but does not make the co-expression pattern particularly clear to the eye. Thus, to show this co-expression more clearly, we present the bicluster again in Panel-B, except this time with the rows and columns rearranged so that the coefficients of the first principal-component-vector change monotonically. As can be seen, there is a striking pattern of correlation across the 793 genes for the 45 cases shown.

Some of our results are shown in Fig 5, which illustrates a large bicluster—comprising *m* = 45 of the *M*_*D*_ = 340 cases and *n* = 793 of the *N* = 16738 genes—that was embedded in the case-matrix and discovered using our algorithm. This bicluster consists of genes that, taken individually, are neither significantly over-expressed nor under-expressed—relative to the control population. Instead, these 793 genes are significantly co-expressed (i.e., either strongly correlated or anti-correlated) across a significant fraction of the case population (in this case 45/340 ∼ 13% of the cases), without being as significantly co-expressed across a comparable fraction of the control population. While a little difficult to see in Fig 5A, we’ve rearranged the bicluster in Fig 5B to reveal its co-expression pattern; each patient in the bicluster is either strongly correlated or anti-correlated with this stereotyped pattern. This statement can be quantified as follows: Let’s define $v\in {\mathbb{R}}^{n}$ to be the dominant right-principal-compont of this bicluster, and let *c*_*j*_ be the pearson’s-correlation between the *j*^th^-patient in the bicluster and *v*. If we use the absolute value |*c*_*j*_| as a measure of ‘alignment’, most of the rows are aligned (with the stereotyped pattern) at a value of 90% or more.

**Fig 6:**
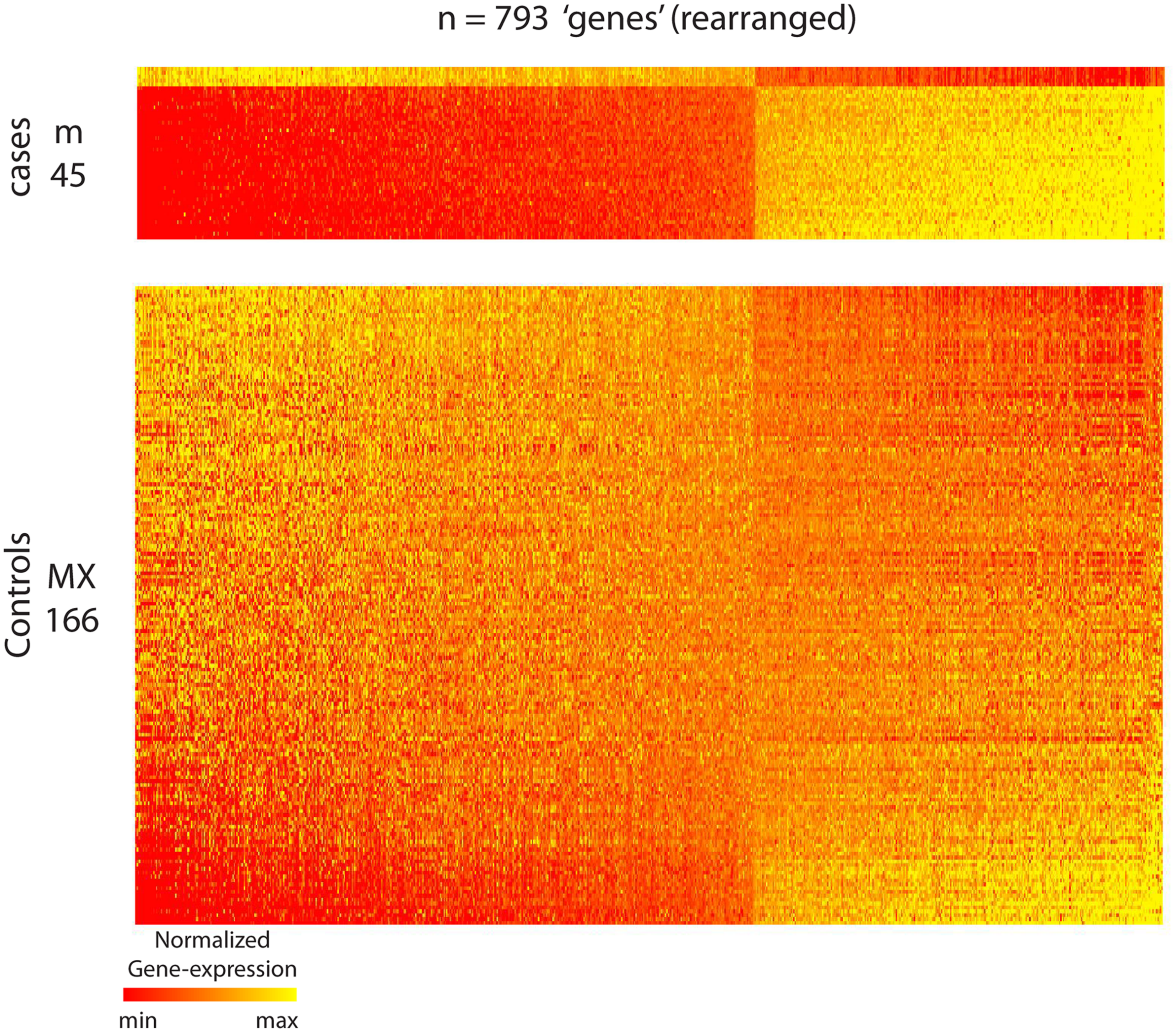
Contrasting a bicluster with controls. This shows the bicluster of Fig 5B on top, and the rest of the controls on the bottom. The control-patients have been rearranged in order of their correlation with the co-expression pattern of the bicluster. Even though a few of the controls (i.e,. ∼ 3/166) exhibit a coexpression pattern comparable to that expressed by the bicluster, the vast majority do not.

To illustrate that this stereotyped co-expression pattern is indeed case-specific (i.e., not comparably shared across the controls), we replot the bicluster at the top of Fig 6 and below we plot the control data—reorganized in an attempt to reveal co-expression patterns. As one can see, while there are certainly some control patients that exhibit strong correlation or anti-correlation with the stereotyped gene-expression pattern of the bicluster, the majority are not so strongly aligned. In fact, for this example, only 3 of the 166 controls (i.e., significantly less than 45/340) have an alignment |*c*_*j*_| that is greater than 90%; most only exhibit an alignment around 0%−50%. This statement can be further quantified as follows: The distribution of alignments |*c*_*j*_| for the patients in the bicluster is significantly different than the distribution of alignments for the controls; the AUC (i.e., area under the receiver-operator-characteristic curve) for these two distributions is 98%, meaning that there is a 98% chance that a randomly drawn patient from the bicluster will have a higher alignment than a randomly drawn control. Note that this AUC only implies a high prediction accuracy when discriminating cases *within* the bicluster from the controls; this AUC does not translate into high case/control prediction accuracy overall.

Finally, we can ask: How significant is this bicluster? As described in more detail within the Methods, this bicluster has a P-value of ≲ 0.008. We obtain this P-value by comparing this bicluster against the distribution of biclusters obtained under a suitable ‘label-shuffled’ null-hypothesis—i.e., formed from shuffling the case-vs-control labels (see also section A14.2 and Figs A57 and A58 in S2 Text). This level of statistical significance implies that this signal is a ‘real’ feature of the data-set, and suggests that many of the genes implicated in this bicluster may be important for distant metastatic disease.

If this were true, we would expect many of these genes to serve similar functions or affect the same pathway. This is indeed the case: a gene-enrichment analysis performed on these *n* = 793 genes reveals a significant enrichment for mitosis (p = 2e-9), DNA-replication (3e-8), chromosome segregation (p = 2e-5) and many more; including several pathways that are likely to play a role in the development of cancer. See Supplementary-material S2 Data for a full list of gene-enrichment obtained using ‘Seek’ [42]. While these results are encouraging, we would still need to perform some kind of replication study (involving an independent data-set) to ensure that this bicluster is biologically-significant.

### Example-B: Genome-wide-association-study (GWAS)

Our second example is a subset of a Genome-Wide-Association-Study used with permission from the Bipolar Disorders Working Group of the Psychiatric Genomics Consortium (PGC-BIP) [43] Due to data-usage agreements, only cursory information regarding this data-set will be provided here. A more detailed description of the data-set, as well as the structures we’ve found within it, will be provided in a later publication. See section A1.4 of S1 Text for more details regarding this example.

This data-set includes *N* = 276768 alleles with varying minor-allele-frequency genotyped across 16577 patients. These patients fall into two phenotypic categories: 9752 are neurotypical, whereas the remaining 6825 exhibit a particular psychiatric disorder. For this example we’ll try and find a signal within the neurotypical patients that is not shared by those with the disorder; We’ll use the phenotypic information to divide the patients into *M*_*D*_ = 9752 cases and *M*_*X*_ = 6825 controls. Note that, within this example, our nomenclature is non-standard; neurotypical patients are typically referred to as ‘controls’ and not cases. The reason we deviate from this standard is because, below, we will try to find a bicluster within the neurotypical patients that does not extend to include the remaining patients. In order to remain consistent with our notation and equations throughout the rest of the manuscript (as well as S1 and S2 Text), we will refer to these neurotypical patients as cases, and we will store their information in the case-matrix *D*.

In addition to their genotyped data, each patient is also associated with a *N*_*T*_ = 2-dimensional vector of ‘mds-components’ that serve as a continuous-covariate. In this case the continuous-covariate plays the role of a proxy for each patient’s genetic ancestry [37, 38].

Our objective in this situation is similar to Example-A: We would like to search for case-specific biclusters involving subsets of alleles that are structured in some way across a significantly large subset of the case-patients, while not being similarly structured across the control-population. In addition, we’d like to ensure that the biclusters we find are well-distributed with regard to the continuous-covariate (i.e., we don’t want to focus on a subset of patients that all have the same ancestry).

**Fig 7:**
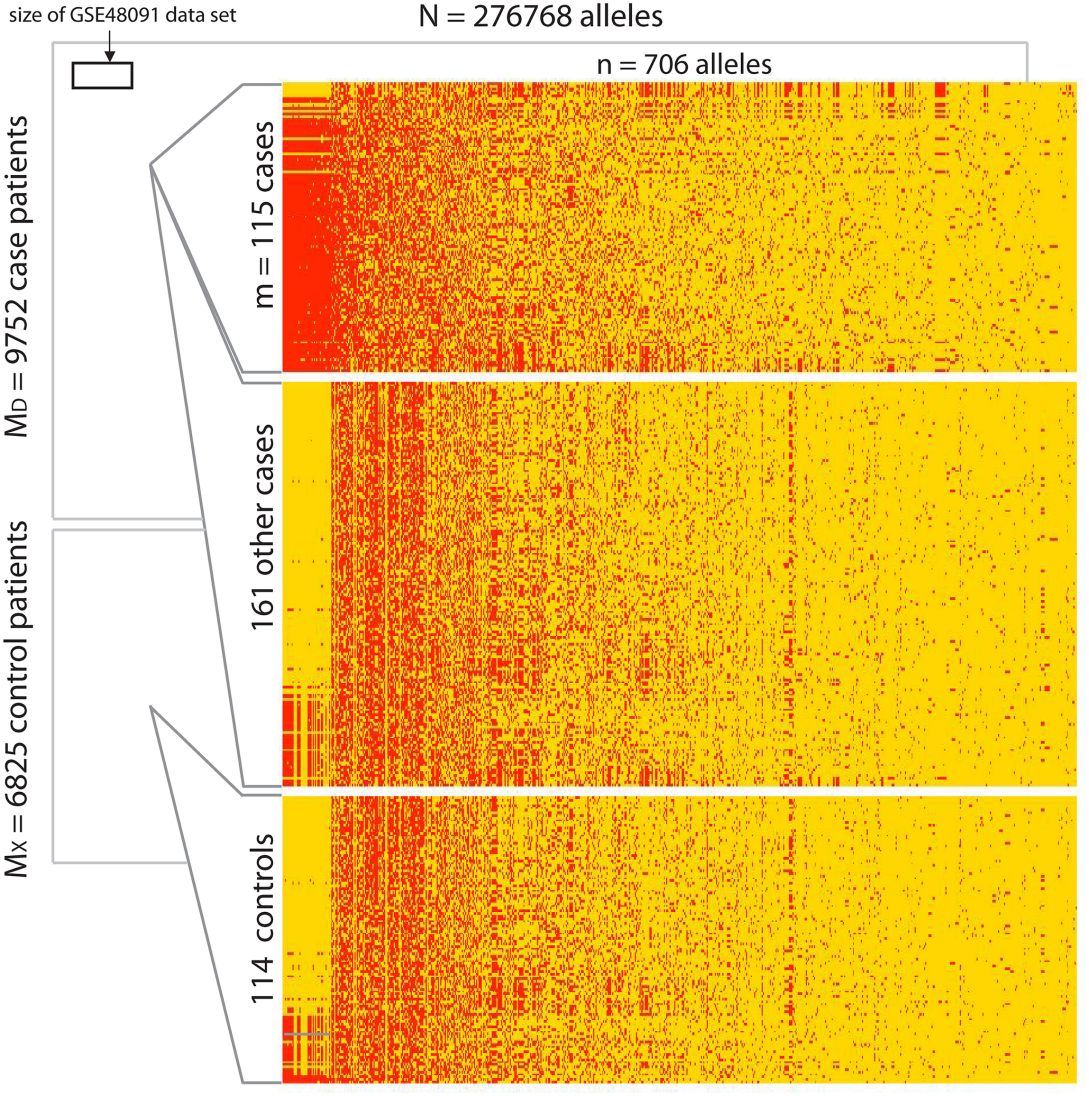
Illustration of bicluster found within genome-wide-association-study dataset. In this figure we illustrate the genome-wide association-study (i.e., GWAS) data-set discussed in Example-B (see main text). This data-set involves 16577 patients, each genotyped across 276768 genetic base-pair-locations (i.e., alleles). Many of these patients have a particular psychological disorder, while the remainder do not. We use this phenotype to separate the patients into *M*_*D*_ = 9752 cases and *M*_*X*_ = 6825 controls. The size of this GWAS data-set is indicated in the background of this picture, and dwarfs the size of the gene-expression data-set used in Example-A (inset for comparison). At the top of the foreground we illustrate an *m* = 115 by *n* = 706 submatrix found within the case-patients. This submatrix is a low-rank bicluster, and the alleles are strongly correlated across these particular case-patients. The order of the patients and alleles within this submatrix has been chosen to emphasize this correlation. For comparison, we pull out a few other randomly-chosen case-patients and control-patients, and present their associated submatrices (defined using the same 706 alleles) further down.

Fig 7 illustrates the size of this data-set, as well as one case-specific low-rank bicluster which we discovered using a version of our algorithm that corrects for controls, as well as sparsity and continuous-covariates (see the ‘2-sided’ covariate-correction described in section A10 of S1 Text). As can be seen from Fig 7, the pattern shown within the bicluster is rather different than the pattern exhibited by the typical control. What may not be obvious from visual inspection is that this bicluster is essentially ‘rank-2’; i.e., the dominant two principal components of this bicluster are large compared to the rest. Put another way, the patients within this bicluster exhibit a second-order correlation across the subset of alleles in the bicluster; a correlation not exhibited by the population at large. We illustrate this second-order structure in Fig A12 in S1 Text.

If we calculate the distribution of alignments across individuals in this bicluster and compare them to the distribution of alignments across the controls (see, e.g., Example-A), we obtain an AUC of > 99.75%. As in Example-A, this AUC by itself only implies that the pattern within the bicluster is significantly different than the pattern outside the bicluster; it does not imply that the bicluster itself is statistically significant. That is to say, this AUC only implies a high prediction accuracy when discriminating cases *within* the bicluster from the controls; this AUC does not translate into high case/control prediction accuracy overall. Nevertheless, as described in more detail in section A14.2 of S2 Text, this bicluster is a statistically-significant feature of the data-set—with a P-value of ≪ 0.05 associated with the label-shuffled null-hypothesis ‘H0x’.

**Fig 8:**
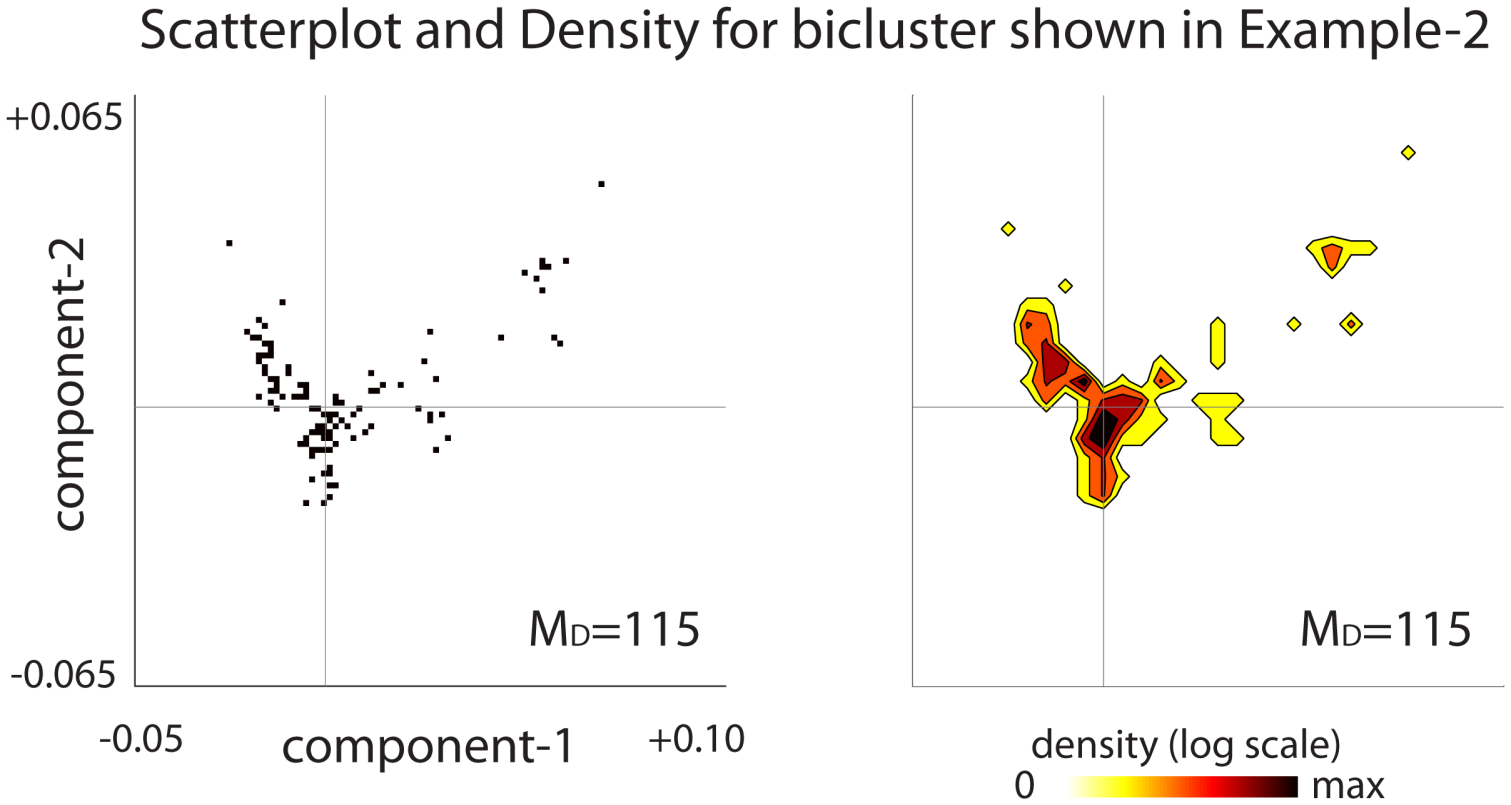
Continuous–covariate-distribution for the bicluster shown in Example-B. As mentioned in the introduction, our algorithm proceeds iteratively, removing rows and columns from the case-matrix until there are none left. One of our goals is to ensure that, during this process, our algorithm focuses on biclusters which involve case-patients that are relatively well balanced in covariate-space. On the left we show a scatterplot illustrating the 2-dimensional distribution of covariate-components across the remaining *m* = 115 case-patients within the bicluster shown in Example-B (i.e., Fig 7). The horizontal and vertical lines in each subplot indicate the medians of the components of the covariate-distribution. On the right we show the same data again, except in contour form (note colorbar). The continuous-covariates remain relatively well-distributed even though relatively few case-patients are left (compare with Fig 9).

**Fig 9:**
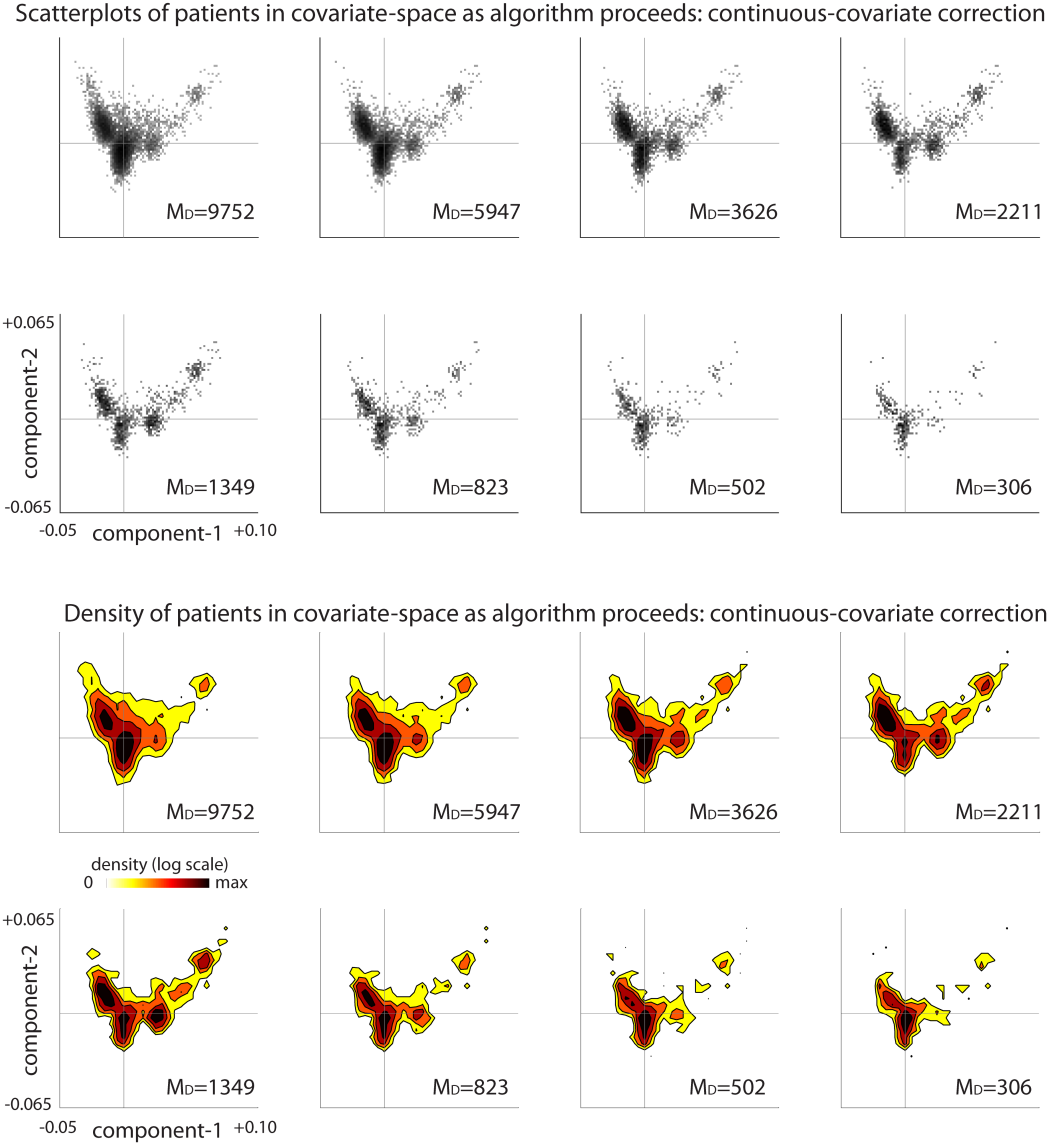
Continuous–covariate-distribution from Example-B as the loop-counting algorithm proceeds. On top we show several scatterplots, sampling from different iterations as our algorithm proceeds. Each scatterplot illustrates the 2-dimensional distribution of covariate-components across the remaining case-patients at that point in the iteration. The horizontal and vertical lines in each subplot indicate the medians of the components of the covariate-distribution. Below we show the same data again, except in contour form (note colorbar). Note that the covariate-distribution remains relatively well-distributed as the algorithm proceeds.

In addition to ensuring that this bicluster was case-specific, our covariate-corrected algorithm has also successfully ensured that this bicluster is balanced with regards to the continuous-covariate. This balance is illustrated in Figs 8 and 9, and further corroborated by Fig A18 in S1 Text. If we were to run our algorithm *without* correcting for the continuous-covariates, then we would find spurious biclusters involving patients that were highly concentrated in just a few regions of covariate-space (see Fig A15 in S1 Text).

If we take a closer look at the bicluster itself, we see that the 706 genetic-loci within the bicluster correspond to 124 different genes. These genes are enriched for many pathways, including: phosphate-ion transmembrane transport (p = 1.6e-4), metal-ion transmembrane transport (p = 5.7e-4), calcium-ion binding (p = 1.2e-3), GTPase-activation (p = 2.2e-3), ion-gated channel activity (p = 3.3e-3), calcium-ion transport (p = 4.3e-3), voltage-gated channel activity (p = 1.0e-2), calcium-signaling (p = 1.1e-3), long-term-potentiation (p = 1.1e-3) and glutamatergic-synapses (p = 7e-3). See S4 Data for a full list of gene-enrichment obtained using ‘Seek’. Because this bicluster was found within the neurotypical patients, it is possible that these genes play a protective role in delaying the development or onset of the psychiatric disorder associated with this data-set. Nevertheless, just as in our previous example, these encouraging results do not yet demonstrate that this bicluster is biologically-significant. In order to truly demonstrate biological-significance, we would need to search for a similar signal within an independent data-set (i.e., perform a replication study).

## Methods

Below we’ll briefly describe our approach to correcting for categorical- and continuous-covariates. In each situation we’ll discuss the simplest possible case, involving case-patients only and disregarding controls. The full details of our method—including how to combine the control-correction with covariate- and sparsity-corrections—is given in section A12 of S2 Text.

### Categorical covariates

If the patients are drawn from different categories (e.g., study-number, platform type, etc.), then it is typically important to ‘correct’ for the effect of this categorical-covariate, and to find biclusters that are independent of covariate-category. In this section we discuss the simplest situation involving only two covariate-categories of equal size (i.e., a binary covariate, such as gender).

Within the context of this scenario, we would like to ignore biclusters that include patients from only one gender, favoring instead biclusters that include a reasonable mixture of patients from both genders. Our strategy is to design a loop-score which tracks the loops associated with the different covariate-categories, comparing them against one another. This loop-score will demote the signal produced by any bicluster down to the signal that would be produced by that bicluster’s ‘balanced component’ (i.e., the subset of that bicluster which straddles both genders equally). As a result, ‘imbalanced’ biclusters that are concentrated in one gender will produce almost no signal. At the same time, ‘balanced’ biclusters that include an equal number of males and females will still produce a strong signal (equivalent, on average, to what they would have produced if we did not correct for covariate-category).

In the simplest scenario (ignoring controls), the covariate-corrected loop-score for any male patient *j* is given by:

$$\begin{array}{c} {\left[Z_{\text{ROW}}\right]}_{j}=\text{min}\left\{{\left[Z_{\text{ROW}}^{1}\right]}_{j}\frac{1}{p}\text{,}\;{\left[Z_{\text{ROW}}^{2}\right]}_{j}\frac{1}{q}\right\}\text{,}\;\text{where} \end{array}$$

$$\begin{array}{c} {\left[Z_{\text{ROW}}^{1}\right]}_{j}=\sum_{\begin{array}{c} j\;\;\text{fixed,}\;\;j^{\prime }\;\;\text{male,}\;\;j^{\prime }\neq j\\ k^{\prime }\neq k \end{array}}D1_{jk}D1_{j^{\prime }k}D1_{j^{\prime }k^{\prime }}D1_{jk^{\prime }}\text{,}\;\text{and} \end{array}$$

$${\left[Z_{\text{ROW}}^{2}\right]}_{j}=\sum_{\begin{array}{c} j\;\;\text{fixed,}\;\;j^{\prime }\;\;\text{female,}\\ k^{\prime }\neq k \end{array}}D1_{jk}D2_{j^{\prime }k}D2_{j^{\prime }k^{\prime }}D1_{jk^{\prime }}$$

where we have divided our data-array *D* into male-patients ‘*D*1’, and female-patients ‘*D*2’, and the fractions *p* = (*M*_*D*1_ − 1)/(*M* − 1) and *q* = (*M*_*D*2_)/(*M* − 1) are the fraction of other males and females, respectively. The covariate-corrected loop-score for a female patient is defined analogously.

Regarding the column-scores:

$$\begin{array}{c} {\left[Z_{\text{COL}}\right]}_{k}=\text{min}\left\{{\left[Z_{\text{COL}}^{11}\right]}_{k}\frac{1}{\alpha_{11}}\text{,}\;{\left[Z_{\text{COL}}^{12}\right]}_{k}\frac{1}{\alpha_{12}}\text{,}\;{\left[Z_{\text{COL}}^{21}\right]}_{k}\frac{1}{\alpha_{21}}\text{,}\;{\left[Z_{\text{COL}}^{22}\right]}_{k}\frac{1}{\alpha_{22}}\right\}\text{,}\;\text{where} \end{array}$$

$$\begin{array}{c} {\left[Z_{\text{COL}}^{11}\right]}_{k}=\sum_{\begin{array}{c} j^{\prime },j\;\;\text{male,}\;\;j^{\prime }\neq j\\ k\;\;\text{fixed,}\;\;k^{\prime }\neq k \end{array}}D1_{jk}D1_{jk^{\prime }}D1_{j^{\prime }k^{\prime }}D1_{j^{\prime }k}\text{,}\;\text{and}\; \end{array}$$

$$\begin{array}{c} {\left[Z_{\text{COL}}^{12}\right]}_{k}=\sum_{\begin{array}{c} j\;\;\text{male,}\;\;j^{\prime }\text{female,}\\ k\;\;\text{fixed,}\;\;k^{\prime }\neq k \end{array}}D1_{jk}D1_{jk^{\prime }}D2_{j^{\prime }k^{\prime }}D2_{j^{\prime }k}\text{,}\;\text{and}\; \end{array}$$

$$\begin{array}{c} {\left[Z_{\text{COL}}^{21}\right]}_{k}=\sum_{\begin{array}{c} j\;\;\text{female,}\;\;j^{\prime }\text{male,}\\ k\;\;\text{fixed,}\;\;k^{\prime }\neq k \end{array}}D2_{jk}D2_{jk^{\prime }}D1_{j^{\prime }k^{\prime }}D1_{j^{\prime }k}\text{,}\;\text{and}\; \end{array}$$

$$\begin{array}{c} {\left[Z_{\text{COL}}^{22}\right]}_{k}=\sum_{\begin{array}{c} j^{\prime },j\;\;\text{female,}\;\;j^{\prime }\neq j\\ k\;\;\text{fixed,}\;\;k^{\prime }\neq k \end{array}}D2_{jk}D2_{jk^{\prime }}D2_{j^{\prime }k^{\prime }}D2_{j^{\prime }k}\text{,}\; \end{array}$$

where fractions *α*_11_, *α*_12_, *α*_21_, *α*_22_ are defined to be $\alpha_{11}=M_{D1}\left(M_{D1}-1\right)/{\bar{M}}^{2}$, $\alpha_{12}=\alpha_{21}=M_{D1}M_{D2}/{\bar{M}}^{2}$, and $\alpha_{22}=M_{D2}\left(M_{D2}-1\right)/{\bar{M}}^{2}$, with ${\bar{M}}^{2}=M^{2}-M_{D1}-M_{D2}$.

A more detailed explanation of this construction, as well as an extension to the general case with three or more covariate-categories, is given in section A9 of S1 Text. An example illustrating this methodology applied to a gene-expression data-set is given in section A1.3 of S1 Text.

One of the important aspects of the general case is that, often, one is interested in biclusters that straddle many covariate-categories, even if they are not fully balanced across *all* the covariate-categories. An example might be a data-set collecting patients across *I*_cat_ separate studies. In such a scenario one might be interested in biclusters that include patients from at least *I*_req_ < *I*_cat_ of these individual studies. Our general method for categorical-covariate-correction requires the user to specify this required *I*_req_, which is used in defining our loop-scores. Details are given in section A9 of S1 Text.

### Continuous covariates

For certain applications each patient is associated with a high-dimensional continuous-covariate. For example, in genome-wide association studies, each patient is often equipped with an *N*_*T*_-dimensional vector of ‘mds-components’, serving as a proxy for the genetic similarity of those patients’ ancestors [37, 38]. Consequently, when attempting to control for genetic-ancestry, we are not interested in ‘imbalanced’ biclusters involving only patients that are concentrated together in mds-space. Instead, we would like to ignore these mds-specific biclusters and focus on ‘balanced’ biclusters which involve patients that are widely dispersed across mds-space.

Our basic strategy for continuous-covariate correction will be similar to our strategy above. We’ll design a loop-score which tracks the continuous-covariates associated with each loop, comparing them against one another. This loop-score will demote the signal of any imbalanced biclusters down to 0. At the same time, balanced biclusters will produce a signal that is—on average—as large as it would have been without covariate-correction.

In the simplest scenario (ignoring controls and categorical-covariates), the covariate-corrected loop-scores are given by:

$$\begin{array}{c} {\left[Z_{\text{ROW}}\right]}_{j}^{2}={\left\lfloor Z_{\text{ROW}}^{\text{base}}\right\rfloor }_{j}^{2}-{\left[Z_{\text{ROW}}^{\left[T\right]}\right]}_{j}^{2}\text{,}\;\;{\left[Z_{\text{COL}}\right]}_{k}={\left[Z_{\text{COL}}^{\text{base}}\right]}_{k}-{\left[Z_{\text{COL}}^{\left[T\right]}\right]}_{k}\text{,}\; \end{array}$$

where the ‘base’ scores are defined via:

$$\begin{array}{c} {\left[Z_{\text{ROW}}^{\text{base}}\right]}_{j}=\underset{\begin{array}{c} j\;\;\text{fixed,}\;\;j^{\prime }\in D\text{,}\;\;j^{\prime }\neq j\text{;}\\ k^{\prime }\neq k \end{array}}{\tilde{\Sigma }}D_{jk}D_{j^{\prime }k}D_{j^{\prime }k^{\prime }}D_{jk^{\prime }}\text{,}\;\text{and} \end{array}$$

$$\begin{array}{c} {\left[Z_{\text{COL}}^{\text{base}}\right]}_{k}=\underset{\begin{array}{c} j^{\prime },j\in D\text{,}\;\;j^{\prime }\neq j\text{;}\\ k\;\;\text{fixed,}\;\;k^{\prime }\neq k \end{array}}{\tilde{\Sigma }}D_{jk}D_{jk^{\prime }}D_{j^{\prime }k^{\prime }}D_{j^{\prime }k}\text{,} \end{array}$$

and the covariate-averaged scores are defined via:

$${\left[Z_{\text{ROW}}^{\left[T\right]}\right]}_{j}^{2}=\frac{1}{\kappa^{2}}\underset{t}{\tilde{\Sigma }}{\left\lfloor Z_{\text{ROW}}^{\left[t\right]}\right\rfloor }_{j}^{2}\text{,}\;\;\text{and}\;\;{\left[Z_{\text{COL}}^{\left[T\right]}\right]}_{k}=\frac{1}{\kappa^{2}}\underset{t}{\tilde{\Sigma }}{\left[Z_{\text{COL}}^{\left[t\right]}\right]}_{k}\text{,}\;\text{with}$$

$${\left[Z_{\text{ROW}}^{\left[t\right]}\right]}_{j}=\underset{\begin{array}{c} j\;\;\text{fixed,}\;\;j^{\prime }\in D\text{,}j^{\prime }\neq j\text{;}\\ k^{\prime }\neq k \end{array}}{\tilde{\Sigma }}D_{jk}D_{j^{\prime }k}T_{j^{\prime }t}D_{j^{\prime }k^{\prime }}D_{jk^{\prime }}T_{jt}\text{,}\;\text{for}\;\text{each}\;t\in \left\{1,\ldots .N_{T}\right\}\text{,}\;\text{and}$$

$${\left[Z_{\text{COL}}^{\left[t\right]}\right]}_{k}=\underset{\begin{array}{c} j^{\prime },j\in D\text{,}\;\;j^{\prime }\neq j\text{;}\\ k\;\;\text{fixed,}\;\;k^{\prime }\neq k \end{array}}{\tilde{\Sigma }}D_{jk}T_{jt}D_{jk^{\prime }}D_{j^{\prime }k^{\prime }}T_{j^{\prime }t}D_{j^{\prime }k}\text{,}\;\text{for}\;\text{each}\;t\in \left\{1,.\ldots N_{T}\right\}\text{.}$$

In the above expressions we denote by *T* the *M* × *N*_*T*_ matrix containing the continuous-covariates (i.e., row-*j* of *T* contains the covariates for patient *j*). The sum $\tilde{\Sigma }$ denotes a normalized sum (i.e., the total is divided by the number of summands), the function ⌊*x*⌋ = max(0, *x*), and *κ*^2^ is a parameter that depends on *N*_*T*_ (for *N*_*T*_ = 2, *κ*^2^ ≈ 0.34).

A more detailed explanation of this construction, including the calculation of *κ*^2^ along with corroborating numerical experiments, is given in section A10 of S1 and S2 Text.

### Sparsity

Up to this point we’ve assumed that—after binarization—each column of the data-matrix has a comparable number of positive and negative entries. This assumption is often valid when dealing with gene-expression data (where we are free to normalize around the median of each column), but not when dealing with genotyped data. Indeed, single-nucleotide-polymorphisms (SNPs) can often involve minor-allele-frequencies (MAFs) that are quite small (e.g., 0.1 or smaller), giving rise to a large imbalance between ±1-entries in each column of the data-matrix.

If a subset of columns in the data-matrix has a surplus of, say, negative entries, then that portion of the data-matrix will be ‘sparse’ (i.e., it will contain far fewer ‘+1’-entries than ‘−1’-entries). Such a sparse region is likely to contain large submatrices consisting of mostly negative entries, simply due to their abundance. The loop-counting algorithm we’ve described so far will typically focus on these large submatrices, even though they are not statistically significant. The reason for this is that the loop-score above assumes that each loop carries the same weight, regardless of whether or not that loop comprises positive or negative entries.

It is straightforward to correct for sparsity by ‘normalizing’ each column of *D*; This normalization rescores each loop so that ±1-entries that are otherwise abundant do not add much to the score. Conversely, entries that are otherwise rare add more to the score.

Assuming that column-*k* of the data-matrix *D* has sparsity coefficient *p*_*k*_, and setting *q*_*k*_ = 1−*p*_*k*_, we calculate the sparsity-corrected loop-scores as follows (ignoring controls and covariates for now):

$${\left[Z_{\text{ROW}}\right]}_{j}=\underset{\begin{array}{c} j\;\;\text{fixed,}\;\;j^{\prime }\neq j\text{;}\\ k^{\prime }\neq k \end{array}}{\tilde{\Sigma }}{\left[D-1\alpha^{⊺}\right]}_{jk}\Delta_{kk}{\left[D^{⊺}-\alpha 1^{⊺}\right]}_{kj^{\prime }}{\left[D-1\alpha^{⊺}\right]}_{j^{\prime }k^{\prime }}\Delta_{k^{\prime }k^{\prime }}{\left[D^{⊺}-\alpha 1^{⊺}\right]}_{k^{\prime }j}\text{,}$$

$$\begin{array}{c} {\left[Z_{\text{COL}}\right]}_{k}=\underset{\begin{array}{c} j^{\prime },j\in D\text{,}\;\;j^{\prime }\neq j\text{;}\\ k\;\text{fixed,}\;\;k^{\prime }\neq k \end{array}}{\tilde{\Sigma }}{\left[D^{⊺}-\alpha 1^{⊺}\right]}_{kj}{\left[D-1\alpha^{⊺}\right]}_{jk^{\prime }}\Delta_{k^{\prime }k^{\prime }}{\left[D^{⊺}-\alpha 1^{⊺}\right]}_{k^{\prime }j^{\prime }}{\left[D-1\alpha^{⊺}\right]}_{j^{\prime }k}\Delta_{kk}\text{,} \end{array}$$

where $\vec{\alpha }\in {\mathbb{R}}^{N}$ is the *N* × 1 vector of means *α*_*k*_ = *p*_*k*_ − *q*_*k*_, $\Delta \in {\mathbb{R}}^{N\times N}$ is the diagonal matrix with entries $\Delta_{kk}^{-1}=4p_{k}q_{k}$, and $\vec{1}\in {\mathbb{R}}^{M}$ is the *M* × 1 vector of all ones. See section A11 of S2 Text for more details, as well as corroborating numerical experiments.

### Combining all the corrections

In the previous sections we’ve discussed modifications to our algorithm that correct for controls, covariates and sparsity. These can be combined in the following order to produce a single loop-score that corrects for all these features simultaneously:

1. Correct for sparsity.
2. Correct for continuous covariates.
3. Correct for categorical covariates.
4. Correct for cases versus controls.

The motivation underlying these choices, as well as computational details and corroborating numerical experiments, are found in section A12 and A13 of S2 Text.

### Obtaining a p-value

In addition to listing the rows and columns in the order that they were eliminated, our loop-counting algorithm also produces a list of the average row- and column-scores taken across the remaining data-matrix at each iteration. We refer to these average row- and column-scores as row- and column-traces, as they are both proportional to the trace of *DD*^⊺^*DD*^⊺^ in the *D*-only situation. These trace-lists allow us to determine a p-value for our bicluster without applying a threshold to determine the ‘boundary’ of the bicluster.

To describe this process, let’s focus on Example-A (i.e., the gene-expression analysis). In this example we are comparing case-patients to control-patients. The row- and column-traces in this case can be rescaled to lie between −2 and +2 (and usually fall in the interval [0, 1]). These traces provide a measure of how tightly correlated the remaining data-matrix is at each iteration. More specifically: if, after sufficiently many iterations, the row- or column-traces become large (i.e., came close to +1), then the remaining rows and columns should form a highly correlated low-rank bicluster.

Our typical hypothesis (say, H1) is that there exists some disease-related structure in the case-patients that is not exhibited by the control-patients. This is in contrast to the null-hypothesis (H0) in which the case- and control-labels are actually arranged randomly and have no disease-related structure. Under this null-hypothesis the traces we observe after running our algorithm on the original data should be similar to the traces we would find if we were to shuffle the case-control labels randomly (across patients). To draw a sample from this null-hypothesis H0, we shuffle the case-control labels of the patients indiscriminantly while retaining the same number of cases and controls. If we were to correct for covariates, then we would restrict our null-hypothesis slightly (H0x) so that the random-shuffles respect the covariates. For example, if we were correcting for gender as a categorical-covariate, we would shuffle the labels of case-males only with control-males, and shuffle the labels of case-females only with control-females.

**Fig 10:**
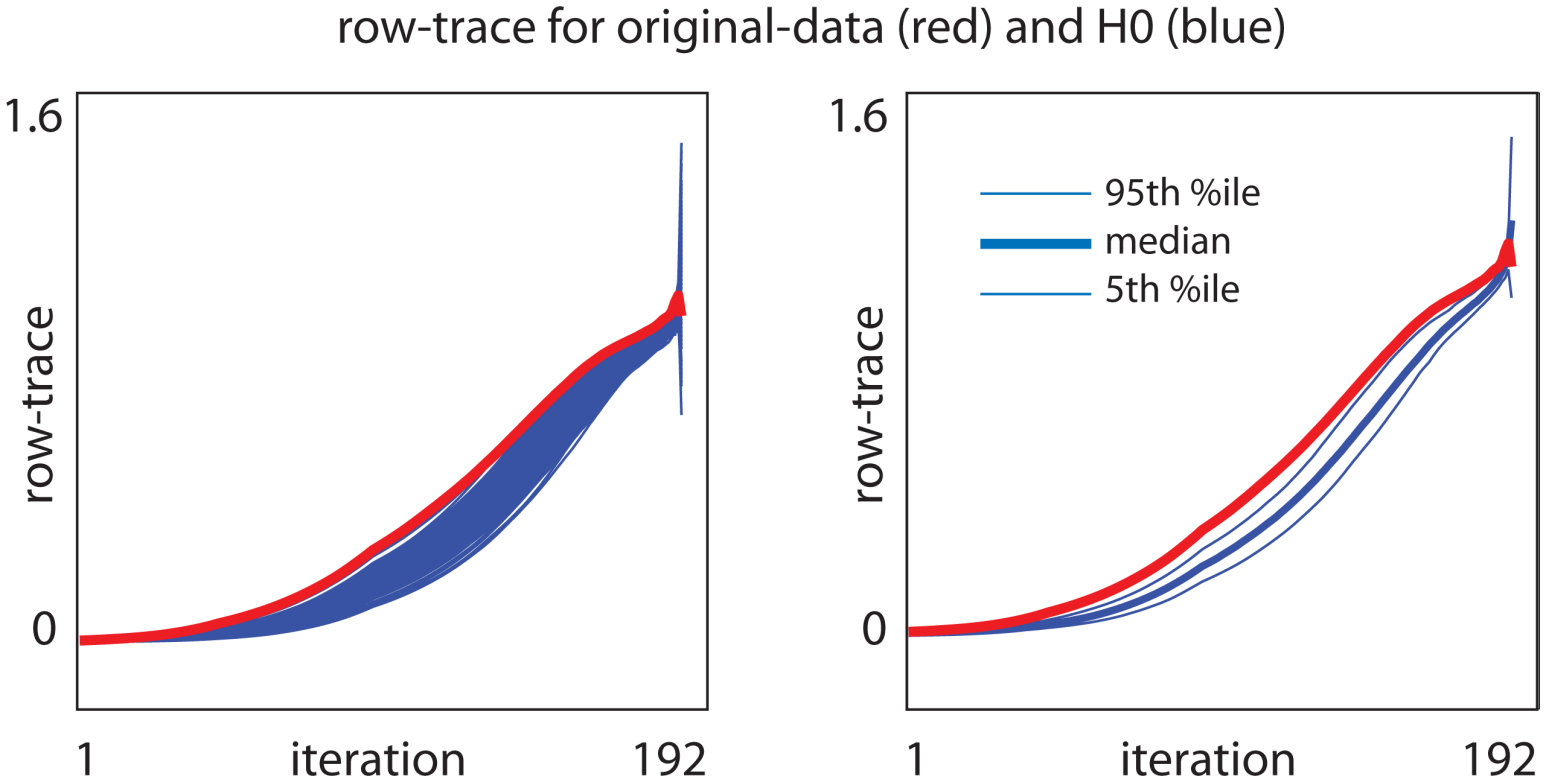
Row-traces for the bicluster shown in Example-A. This bicluster was found by running our algorithm on the data shown in Fig 4. Because we corrected for controls, we compare our original-data to the distribution we obtain under the null-hypothesis H0 (see Methods). On the left we show the row-trace as a function of iteration for the original-data (red) as well as each of the 256 random shuffles (blue). On the right we replot this same trace data, showing the 5th, 50th and 95th percentile (across iterations) of the H0 distribution. Because we are not correcting for any covariates, the column-traces are identical to the row-traces.

**Fig 11:**
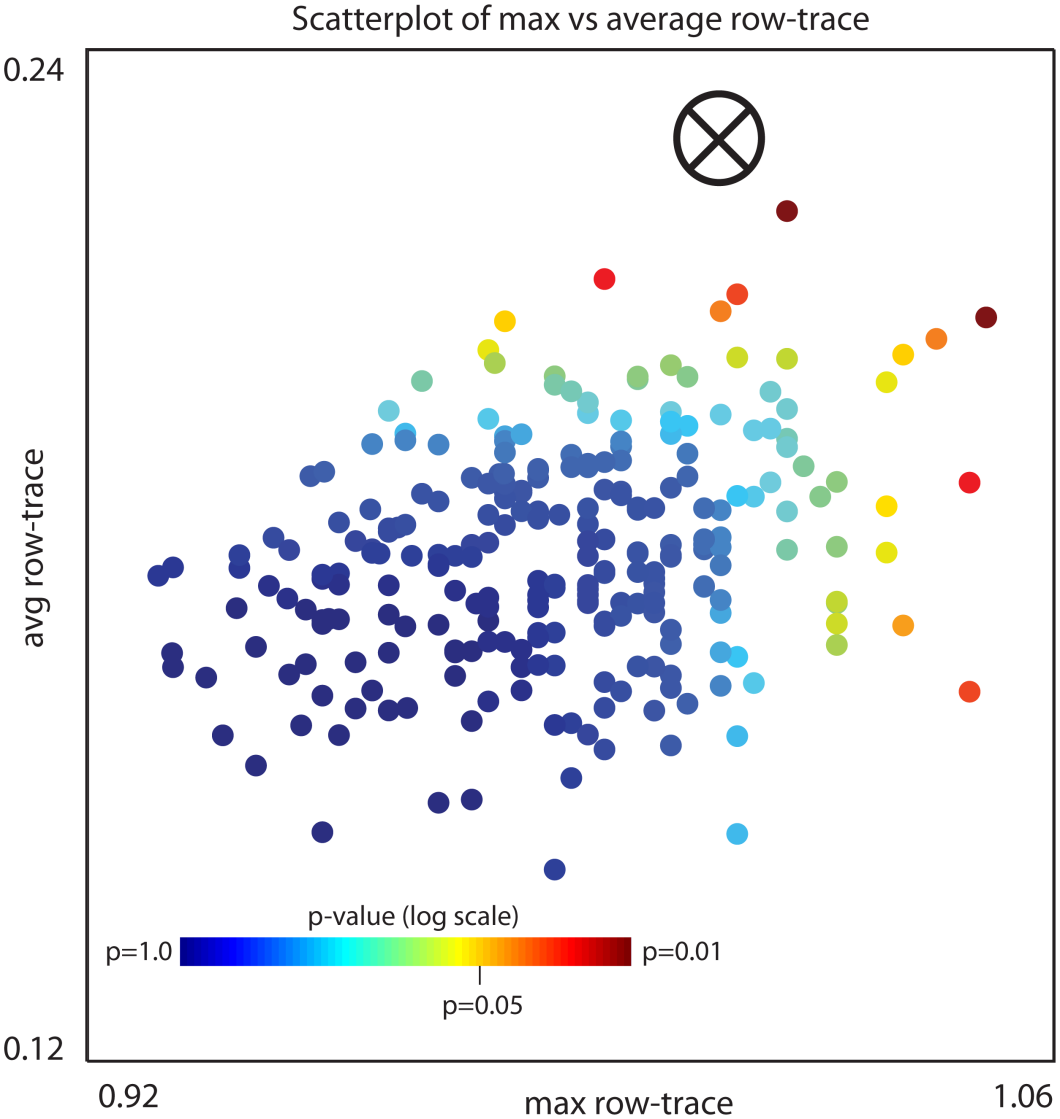
A scatterplot of the data shown in Fig 10. Each row-trace shown on the left in Fig 10 is plotted as a single point in 2-dimensional space; the horizontal-axis corresponds to the maximum row-trace and the vertical-axis corresponds to the average row-trace (taken across the iterations). The original-data is indicated with a ‘⊗’, and each of the random shuffles with a colored ‘•’. The *p*-value for any point $\vec{w}$ in this plane is equal to the fraction of label-shuffled-traces that have either an *x*-position larger than *x*_*w*_ or a *y*-position larger than *y*_*w*_, where *x*_*w*_ and *y*_*w*_ are the *x*- and *y*-percentiles associated with the most extreme coordinate of $\vec{w}$ (details given in section A14.2) of S2 Text. Each random shuffle is colored by its p-value determined by the label-shuffled-distribution. By comparing the original-trace with the shuffled-distribution we can read off a p-value for the original-data of ≲ 0.008.

For each label-shuffled trial we rerun our algorithm, and collect the output. Each trial does not depend on any of the other trials, and they can all be processed in parallel. This library of label-shuffled traces produces a distribution associated with H0 (or H0x). We use this label-shuffled distribution to calculate a p-value for the traces produced by the original data. An illustration along these lines is shown in Figs 10 and 11, which corresponds to our Example-A for gene-expression-analysis.

A full description of this procedure, as well as an analogous procedure used to find a p-value for Example-B, can be found in section A14 of S2 Text. This section also describes how we delineate each bicluster, and how we search for multiple biclusters.

## Discussion

This paper has focused so far on the detection of low-rank biclusters within a larger data-matrix. Our loop-counting method does a reasonable job of locating these low-rank biclusters (see sections A5 and A6 of S1 Text for analytical bounds), and can be adapted to deal with many common features of experimental design (see sections A8, A9, A10 and A11 of S1 and S2 Text).

Our methods can also be easily extended to tackle many related problems. For example, we can treat ‘genetic controls’, look for ‘rank-0’ biclusters (i.e., differentially-expressed biclusters), and even look for ‘triclusters’. The first two of these topics are explained in more detail in sections A15.1 and A15.2 of S2 Text, and we briefly discuss the third here.

There are often situations where the data-paradigm we’ve assumed—involving *N* measurements taken across *M* patients—doesn’t suffice. For example, in a clinical study there may be *M* patients, each of which are subjected to *P* different kinds of treatments (e.g., therapy regimes, medications, etc). For each of these *P* treatments, *N* different variables may be measured for each patient. Within this paradigm, the data isn’t best represented as a 2-dimensional array (e.g., a matrix), but rather as a 3-dimensional array (i.e., a box or a cube) comprising both rows and columns, as well as ‘layers’. In formal terms, we imagine our data arranged into an array *D* of dimension *M* × *N* × *P*, where *D*_*j*,*k*,*l*_ corresponds to the *k*^th^ measurement of the *j*^th^-patient as they undergo therapy *l*.

Within this 3-dimensional array, it is often prudent to search for subsets of patients that exhibit some kind of simple structure across a subset of measurements as well as a subset of treatments. Such a ‘tricluster’ would correspond to a ‘sub-cube’ of the data, rather than simply a submatrix. The techniques we have discussed in the main text can readily be extended to search for these kinds of objects as well.

In the simplest case (i.e., ignoring controls, covariates and sparsity), we first binarize the data-cube *D*, sending each entry to either +1 or −1, depending on its sign. Once we binarize *D*, we can calculate the following scores:

$$\begin{array}{ccc} {\left[Z_{\text{ROW}}\right]}_{j} & =\underset{\begin{array}{c} j\;\;\text{fixed,}\;\;j^{\prime }\neq j\text{;}\\ k^{\prime }\neq k\;\;\text{;}\;\;l \end{array}}{\tilde{\Sigma }} & D_{jkl}D_{j^{\prime }kl}D_{j^{\prime }k^{\prime }l}D_{jk^{\prime }l}+\underset{\begin{array}{c} j\;\;\text{fixed,}\;\;j^{\prime }\neq j\text{;}\\ k\;\;\text{;}\;\;l^{\prime }\neq l \end{array}}{\tilde{\Sigma }}D_{jkl}D_{j^{\prime }kl}D_{j^{\prime }kl^{\prime }}D_{jkl^{\prime }}\text{,}\; \end{array}$$

$$\begin{array}{ccc} {\left[Z_{\text{COL}}\right]}_{k} & = & \underset{\begin{array}{c} k\;\;\text{fixed,}\;\;k^{\prime }\neq k\text{;}\\ j^{\prime }\neq j\;\;\text{;}\;\;l \end{array}}{\tilde{\Sigma }}D_{jkl}D_{jk^{\prime }l}D_{j^{\prime }k^{\prime }l}D_{j^{\prime }kl}+\underset{\begin{array}{c} k\;\;\text{fixed,}\;\;k^{\prime }\neq k\text{;}\\ j\;\;\text{;}\;\;l^{\prime }\neq l \end{array}}{\tilde{\Sigma }}D_{jkl}D_{jk^{\prime }l}D_{jk^{\prime }l^{\prime }}D_{jkl^{\prime }}\text{,}\; \end{array}$$

$$\begin{array}{ccc} {\left[Z_{\text{LYR}}\right]}_{l} & =\underset{\begin{array}{c} l\;\;\text{fixed,}\;\;l^{\prime }\neq l\text{;}\\ j^{\prime }\neq j\;\;\text{;}\;\;k \end{array}}{\tilde{\Sigma }} & D_{jkl}D_{jkl^{\prime }}D_{j^{\prime }kl^{\prime }}D_{j^{\prime }kl}+\underset{\begin{array}{c} l\;\;\text{fixed,}\;\;l^{\prime }\neq l\text{;}\\ j\;\;\text{;}\;\;k^{\prime }\neq k \end{array}}{\tilde{\Sigma }}D_{jkl}D_{jkl^{\prime }}D_{jk^{\prime }l^{\prime }}D_{jk^{\prime }l}\text{,}\; \end{array}$$

corresponding to the row-, column- and layer-scores, respectively. Once we’ve calculated these scores, we can remove the rows, columns and layers with low scores, and repeat the entire process. As before, this process will focus on the rows, columns and layers of any embedded ‘triclusters’ *B* with high probability as long as they are sufficiently large and sufficiently low-rank.

**Fig 12:**
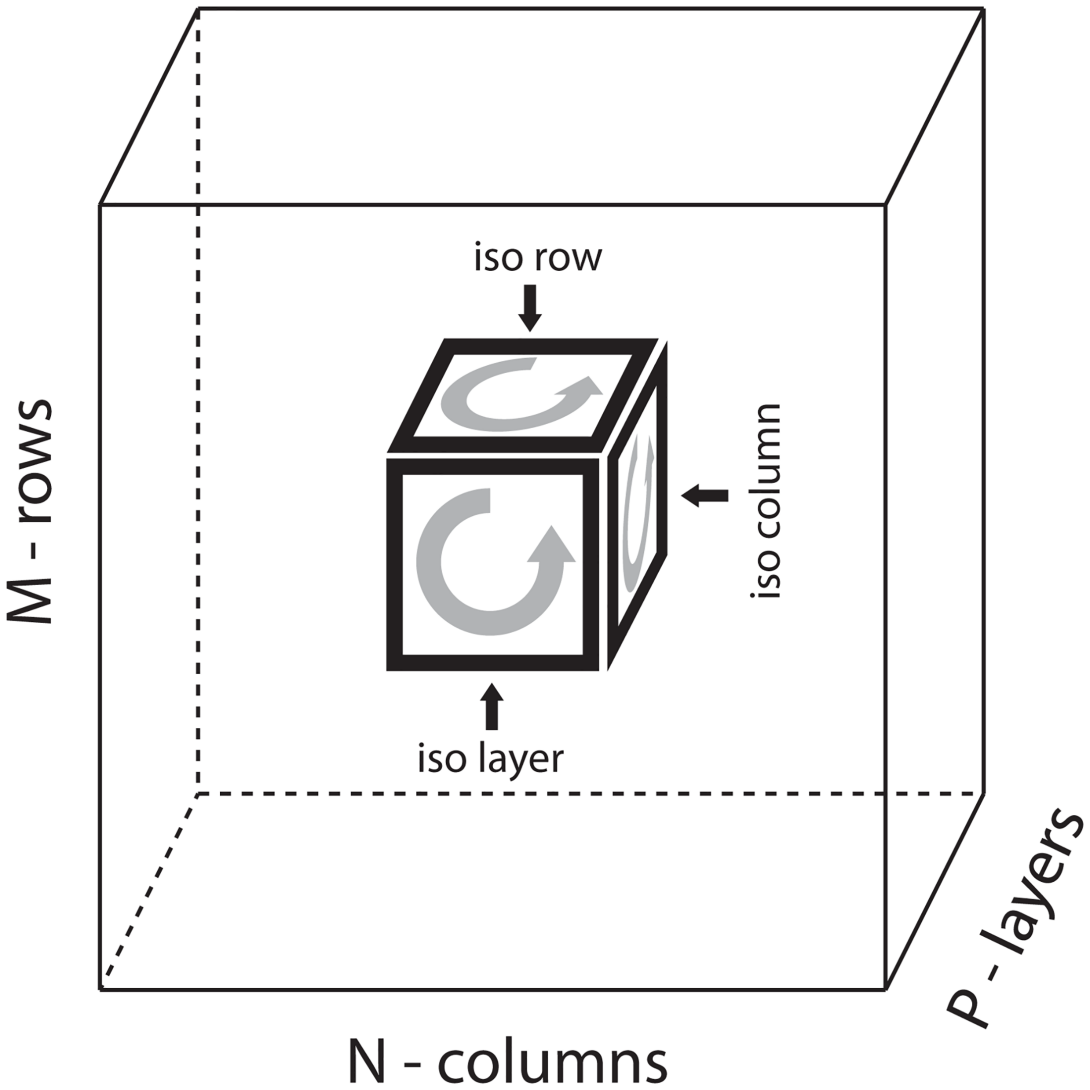
Illustration of the loops within a 3-dimensional array. We sketch the structure of a 3-dimensional data-array *D*, with *J* rows, *K* columns and *P* ‘layers’. Each entry *D*_*j*,*k*,*l*_ will lie in the cube shown. The loops within *D* can be divided into 3-categories: (a) iso-layer loops that stretch across 2 rows and 2 columns, (b) iso-column loops that stretch across 2 rows and 2 layers, and (c) iso-row loops that stretch across 2 columns and 2 layers. The row-score [*Z*_ROW_]_*j*_ aggregates all the iso-column and iso-layer loops associated with row-*j*. The column-score [*Z*_COL_]_*k*_ aggregates all the iso-row and iso-layer loops associated with column-*k*. The layer-score [*Z*_LYR_]_*l*_ aggregates all the iso-row and iso-column loops associated with layer-*l*.

The reason this process works is that—as before—the scores accumulate the ranks of the various loops within *D*. However, unlike the simpler situation discussed in the main text, *D* is a 3-dimensional array (and not merely a matrix). Consequently, there are 3 different kinds of loops within *D*, each traversing a different pair of array-dimensions (see Fig 12). Any loop within *D* that does not lie entirely within *B* is just as likely to be rank-1 as it is to be rank-2. On the other hand, loops within *D* that are entirely contained within *B* are more likely to be rank-1 than rank-2. This probability is not 100%, but it is still significantly greater than 50%, with the exact value dependent on the kind of structure exhibited within *B*. Moreover, this probability is still significantly greater than 50% even in the presence of a moderate amount of noise (e.g., if *B* were not exactly a sum of outer products).

Slightly more detail, along with numerical experiments, can be found in section A15.3 of S2 Text. Techniques along these lines have been used to find triclusters in clinical data involving several patients, measurements and therapies. See [44] for some preliminary results.

## Supporting information

S1 Data Gene enrichment analysis of Example-1a in S1 Text. This spreadsheet contains the full list of pathways produced by a gene-enrichment analysis of the bicluster shown in the first example in the Supplementary-text (involving gene-expression data). To perform this gene-enrichment analysis we used ‘Seek’. Each page of this spreadsheet lists the enrichment results using one of the 11 different gene-ontology databases available within the ‘Seek’ software.
(XLSX)

S2 Data Gene enrichment analysis of Example-A in main text (Example-1b in S1 Text). This spreadsheet contains the full list of pathways produced by a gene-enrichment analysis of the bicluster shown in Example-A in the Main text (involving gene-expression data). To perform this gene-enrichment analysis we used ‘Seek’. Each page of this spreadsheet lists the enrichment results using one of the 11 different gene-ontology databases available within the ‘Seek’ software.
(XLSX)

S3 Data Gene enrichment analysis of Example-2 in S1 Text. This spreadsheet contains the full list of pathways produced by a gene-enrichment analysis of the bicluster shown in the Example-2 in the Supplementary-text (involving gene-expression data). To perform this gene-enrichment analysis we used ‘Seek’. Each page of this spreadsheet lists the enrichment results using one of the 11 different gene-ontology databases available within the ‘Seek’ software.
(XLSX)

S4 Data Gene enrichment analysis of Example-B in main text (Example-3 in S1 Text). This spreadsheet contains the full list of pathways produced by a gene-enrichment analysis of the bicluster shown in Example-B in the main text (involving GWAS-data). To perform this gene-enrichment analysis we used ‘Seek’. Each page of this spreadsheet lists the enrichment results using one of the 11 different gene-ontology databases available within the ‘Seek’ software.
(XLSX)

S1 Text Supplementary Information Part 1. The first part of this 2-part document describes in more detail the examples shown in the main text. This document also contains a more detailed analysis of our method.
(PDF)

S2 Text Supplementary Information Part 2. The second part of this 2-part document describes in more detail the examples shown in the main text. This document also contains proofs of certain inequalities, and a comparison with a simple spectral method.
(PDF)

S1 Source Code Matlab source code. This archive contains Matlab source code for our loop-counting methods, along with a tutorial (also written in Matlab) which will guide users through our our first example (involving gene-expression data).
(GZ) Click here for additional data file.

S2 Source Code C source code. This archive contains C source code for our loop-counting methods, along with several drivers (written in Matlab) which allow users to replicate each of our numerical experiments (shown in the main text and supplementary information).
(GZ) Click here for additional data file.
