# Supplementary material for: A loop-counting method for covariate-corrected low-rank biclustering of gene-expression and genome-wide association study data
Source: PLoS Comput Biol. 2018 May 14;14(5):e1006105. doi: 10.1371/journal.pcbi.1006105 (PMC5997363; doi:10.1371/journal.pcbi.1006105)
Supplement: S1 Text — The first part of this 2-part document describes in more detail the examples shown in the main text. This document also contains a more detailed analysis of our method. (PDF) [file pcbi.1006105.s005.pdf]

Supplementary Information for: A loop-counting method for covariate-corrected low-rank biclustering of gene-expression and genome-wide association study data

Aaditya V. Rangan   Caroline C. McGrouther   John Kelsoe   Nicholas Schork   Eli Stahl
Qian Zhu   Arjun Krishnan   Vicky Yao   Olga Troyanskaya   Seda Bilaloglu
Preeti Raghavan   Sarah Bergen   Anders Jureus   Mikael Landen
Bipolar Disorders Working Group of the Psychiatric Genomics Consortium

Contents

1 Introduction **4**
1.1 Background . . . 4
1.2 Additional information for Example-1: Gene expression analysis . . . 6
1.3 Additional information for Example-2: Gene expression analysis . . . 10
1.4 Additional Information for Example-3: Genome-Wide-Association-Study (GWAS) . . . 17

2 Simple case: D only **20**

3 Calculating the scores: **29**
3.1 Updating the scores using a low-rank update: . . . 30

4 Interpreting the scores: **30**

5 Generalization to noisy biclusters **31**
5.1 Constructing a low-rank bicluster B: . . . 35
5.2 Generating a sparse low-rank B . . . 37

6 Application to the Planted-Bicluster problem: **37**

7 More detailed discussion of the algorithm: **38**
7.1 Why binarize? Advantages and disadvantages . . . 40
7.2 Why use loops? Advantages and disadvantages . . . 44
7.3 Why iterate? . . . 48
7.4 Comparison with some other biclustering methods . . . 48

8 Correcting for Controls: D and X **56**

9 Correcting for Categorical Covariates: **59**
9.1 Interpretation when $I_{\text{cat}} = 2$ and $I_{\text{req}} = 2$ . . . 62

10 Correcting for Continuous Covariates: **63**

11 Correcting for Sparsity **72**

12 Putting it all together **75**

13 Notes regarding computation **81**

14 Notes regarding application **85**
14.1 Interpreting the output: . . . 85
14.2 Determining significance: . . . 90
14.3 Delineating a bicluster: . . . 91
14.4 Finding secondary biclusters: . . . 91

15 Additional Applications **96**
15.1 Accounting for genetic-controls: . . . 96
15.2 Searching for 'rank-0' (i.e., differentially-expressed) biclusters: . . . 98
15.3 Searching for triclusters: . . . 106

16 Various properties of multivariate gaussians **114**
16.1 one-dimensional gaussian: . . . 114
16.2 convolution of two one-dimensional gaussians: . . . 116
16.3 two-dimensional gaussian: . . . 116
16.4 isotropic two-dimensional gaussian: . . . 116
16.5 restriction of two-dimensional gaussian to quadrants: . . . 116
16.6 approximation of orthonormal $\hat{u}, \hat{v}$: . . . 117
16.7 convolution of two two-dimensional gaussians: . . . 117

17 Asymptotic formula for $g_{1,\varepsilon,m}$: **118**

18 Low-noise limit for $B^{\intercal}B$: **118**

19 Information-theoretic phase-transitions for rank-1 planted-bicluster problem **120**

20 Does binarization destroy angular information? **121**

21 Analysis of loop-scores **121**

22 Comparison with a simple spectral-biclustering method **124**
22.1 Size-threshold: . . . 126
22.2 Noise-threshold: . . . 127
22.3 Behavior near the detection-threshold . . . 128
22.4 A remark regarding message-passing algorithms . . . 131
22.5 Accounting for controls: . . . 132

1 Introduction

This document contains supplementary information for the manuscript entitled *A loop-counting method for covariate-corrected low-rank biclustering of gene-expression and genome-wide association study data.* This supplementary information describes our method in detail, presenting both analysis and numerical experiments where appropriate. Occasionally we will reference citations within the bibliography of the main text. We will denote these citations with an $\mathcal{M}$ before the citation number.

1.1 Background

Many applications in data-analysis involve 'low-rank biclustering'; that is, searching through a large data-matrix to find submatrices which have a low numerical-rank (i.e., for which the rows and columns are strongly correlated – see Fig 1). To give an example from genomics, one might imagine a data-matrix involving several genetic-measurements taken across many patients. In this context a 'bicluster' would correspond to a subset of genetic-measurents that are correlated across a subset of the patients. While some biclusters might include many genes, or extend across most (or all) of the patients, it is also possible for biclusters to include only a small subset of genes and extend across only a small subset of patients. Detecting biclusters such as these provides a first step towards unraveling the physiological mechanisms underlying the heterogeneity within a patient population. With this picture in mind, a natural question is: given a large data-array, how can one quickly detect any such low-rank biclusters? In this document we address this issue, expanding on a biclustering algorithm originally introduced in [$\mathcal{M}$39].

In its most basic form our algorithm is very simple: Given a large $M \times N$ data-matrix D, we 'binarize' the data by sending each entry of D to either $+1$ or -1, depending on its sign. Then we form row-scores $Z_{\text{ROW}} \in \mathbb{R}^M$ by taking the diagonal entries of $DD^\intercal DD^\intercal$, and column-scores $Z_{\text{COL}} \in \mathbb{R}^N$ by taking the diagonal entries of $D^\intercal DD^\intercal D$. We then eliminate the rows and columns of D for which Z_{ROW} and Z_{COL} are small, and repeat the entire process. Eventually, after repeating this process multiple times, we will eliminate almost all the rows and columns of D. If there were indeed a low-rank bicluster hiding within D, then (assuming certain criteria are satisfied) our algorithm will usually find it; retaining the rows and columns of the bicluster until the end.

This algorithm works due to the following observation regarding high dimensional space: a random planar projection of an eccentric gaussian-distribution is typically concentrated in non-adjacent quadrants. This fact implies that 2×2 submatrices of D (referred to as 'loops' in the following sections) contain substantial information about biclusters within D; the rows and columns of any low-rank bicluster will correspond to large values of Z_{ROW} and Z_{COL}, and will thus be retained as the algorithm proceeds. In the sections below we'll analyze our algorithm a little more carefully, and explain under what conditions it is expected to work.

Our ultimate goal is to provide a working code which can be applied to real problems in data-science. To this end, we introduce several modifications to the original algorithm of [$\mathcal{M}$39]; these modifications allow us to account for many considerations which commonly arise in practice. For example, within the context of genomics, the $M \times N$ data-matrix above may correspond to N different measurements each taken across M patients. Within this paradigm, it is often important to correct for:

1. Cases-versus-Controls: Some patients may suffer from a certain disease (i.e., 'cases') while others do not. By correcting for controls we can search for correlation patterns that are limited to the case-population. These case-specific patterns may be useful for clinical diagnosis or for revealing disease mechanisms.

2. Categorical- and continuous-covariates: Often patients come from different studies, or are measured with different machines. Each patient may also be associated with a vector of continuous-covariates (e.g., a vector of mds-components correlated with genetic ancestry). It is often critical to correct for the influence of these covariates when looking for significant patterns.

3. Sparsity: In certain circumstances (e.g., when dealing with genotyped data) the data-matrix can be sparse. Moreover, different columns of the data-matrix can have different sparsity-coefficients (e.g., different minor-allele-frequencies). It is typically important to take this sparsity into account when determining which patterns are significant and which are not.

Before we describe our algorithm in detail we'll briefly present three examples drawn from genomics. Example-1b and Example-3 below correspond to Example-A and Example-B from the main-text, respectively. Collectively, these examples highlight some of the practical considerations necessary for a biclustering algorithm to be useful.

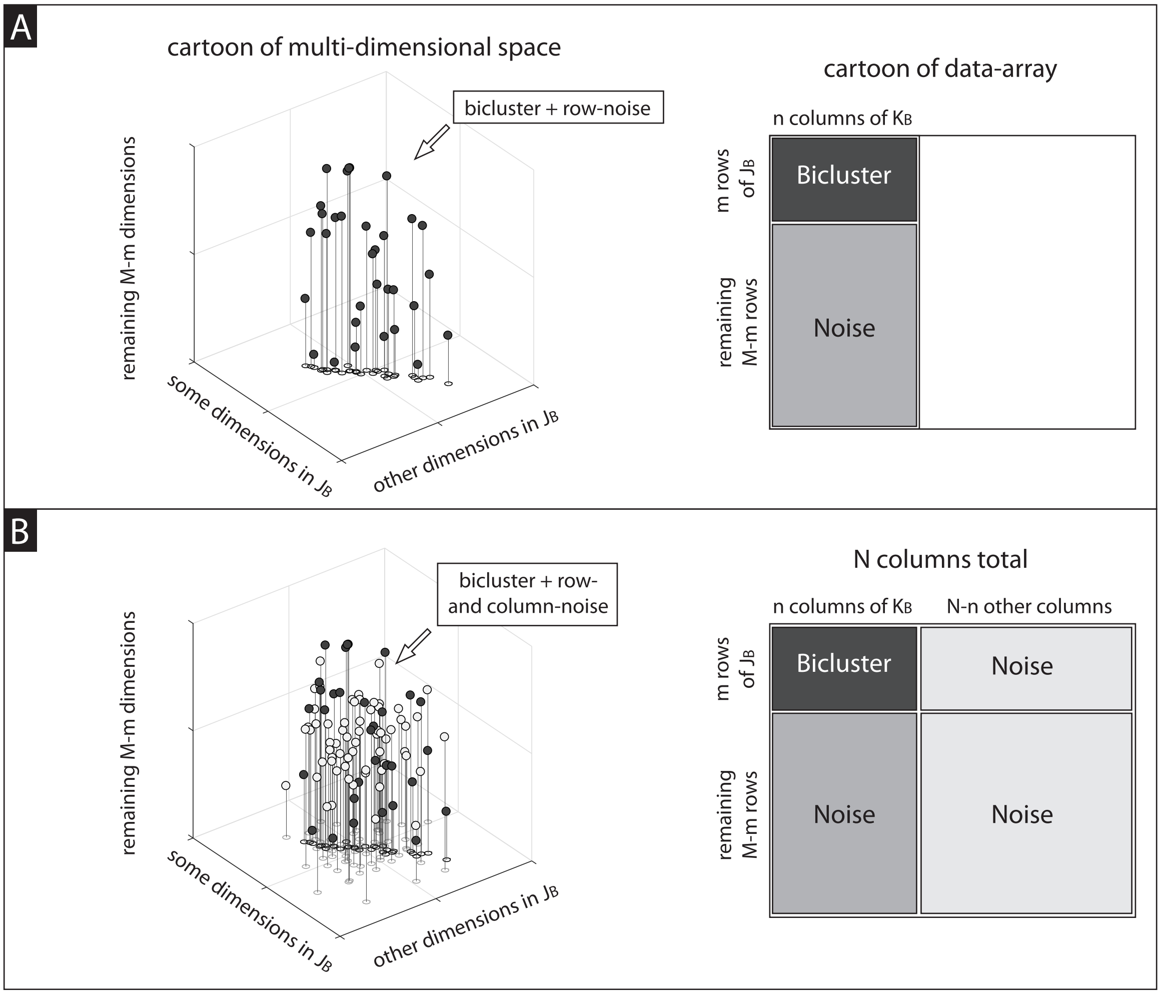

Figure 1: A cartoon illustrating the geometry underlying a 'low-rank bicluster'. In panel-A we imagine a situation involving an $M \times n$ data-set. This data-set contains m correlated rows (which will later form a bicluster in panel-B), as well as $M - m$ noisy rows. We'll assume that the m correlated rows are described by the subset J_B. On the left side of panel-A we've plotted each of the columns of this data-set as though they were points in $\mathbb{R}^M$ (see dark dots). For this illustration we've plotted the m-dimensions of J_B in the xy-plane, and used the z-axis to represent the remaining $M - m$ dimensions. Note that, due to the noisy rows, the data-points will not be confined to a very low-dimensional space.In order to reveal the low-rank structure within the data, it is necessary to first restrict our attention to the m rows of the bicluster. In other words, we need to project the data-points onto the subset of dimensions described by J_B – which is in this case the xy-plane. Only after such a projection will the data lie in a low-dimensional space (note that the shadows of the data-points in the xy-plane align). In panel-B we illustrate an $M \times N$ data-set containing the data shown in panel-A, as well as an additional $N - n$ noisy columns. The correlated $m \times n$ submatrix within this data-set is described by subsets J_B and K_B, and is an example of a low-rank bicluster. On the right side of panel-B we've plotted each of the columns of this $M \times N$ data-set as though they were points in $\mathbb{R}^M$. The points from the columns in K_B are plotted as dark dots (identical to panel-A), whereas the noisy columns drawn from outside K_B are plotted as white dots. After projecting all the dots onto the m-dimensions of J_B (i.e., onto the xy-plane), only the dark shadows from K_B align. The remaining shadows from the noisy columns are scattered about the xy-plane, and do not exhibit any particular correlation. The low-rank bicluster within the data-set has two defining subsets: first, the axes of the projection (i.e., the rows within J_B), and second, the identity of the dark dots whose shadows align (i.e., the columns of K_B). Detecting the bicluster requires the identification of both these subsets. The algorithm presented in this document strives to detect these kinds of biclusters within large data-sets – identifying the subsets J_B and K_B as best it can.

1.2 Additional information for Example-1: Gene expression analysis

Our first example is taken from the GSE48091 data-set available from the gene-expression-omnibus[1] uploaded in 2015. The original data-set comprises 28499 gene-expression measurements (referred to later as 'genes' for simplicity) collected across 623 patients diagnosed with primary breast cancer.

Within this data-set some of these patients were 'cases' that developed distant metastatic disease, whereas others were 'controls' that did not. This data-set was prepared specifically so that the control-population could be compared to the case-population: the control-population was randomly matched to the case-population by adjuvant therapy, age and calendar period at diagnosis [1]. The original data-set also had a nested case-control design, with several levels to the case-control hierarchy: most of the patients (506/623) were assigned to the base-level of this hierarchy, while a minority (117/623) were assigned to deeper levels of the hierarchy. For this example we will limit our analysis to the patients at the base-level: i.e., we'll focus on the $M_D = 340$ base-level 'cases' (who developed distant metastatic disease) and $M_X = 166$ base-level 'controls' (who did not). This restriction will allow us to perform a straightforward comparison between cases and controls.

Similar to the original research for which this data-set was created, our ultimate objective will be to find signals that are specific to those case-patients that developed distant metastatic disease. A natural first step towards answering this question would be to look for genes which are differentially-expressed with respect to the case- and control-populations. There are indeed many such genes. In fact, 11711 of the original 28499 genes are significantly differentially-expressed (with a p-value less than 0.05). Many of these differentially-expressed genes have already been considered by the original paper (see [1]).

Example-1a: biclustering the strongly differentially-expressed genes

To begin with, we'll analyze these $N = 11711$ strongly differentially-expressed genes. This data is shown in Fig 2, with each column (i.e., gene) normalized to have median 0 across the patient-population.

Our goal will be to find a bicluster within the case-population. To achieve this goal we can run our control-corrected loop-counting algorithm (described in section 8). Alternatively, because we know a-priori that the bicluster we are searching for involves differentially-expressed genes, we can instead run our control-corrected half-loop algorithm (described in section 15.2).

Regardless of which method we choose, the results in both cases will be very similar. This is because the data-set shown in Fig 2 contains a very large case-specific bicluster. The exact delineation of this bicluster (i.e., which genes and patients are inside versus outside) will depend on the specific methodology used (see section 14.3). Nevertheless, both algorithms will return essentially the same result (i.e., the detected bicluster will be very similar, with a large overlap in terms of patients and genes).

Shown in Fig 3 is an illustration of this large bicluster – comprising $m = 65$ of the $M_D = 340$ cases and $n = 3984$ of the $N = 11711$ genes, detected using our half-loop algorithm. For this example we set the internal parameter γ in our algorithm to ≤ 0.05 (see Fig 32 later on for justification). If we had used our loop-counting algorithm instead of our half-loop algorithm, then the detected bicluster would have been almost the same (i.e., exhibiting a very large overlap with the one shown).

The bicluster shown in Fig 3 consists of genes that, taken individually, are either significantly over-expressed or under-expressed – relative to the control population. We will later refer to this kind of bicluster as a 'rank-0' bicluster, because the rows/columns of the bicluster cluster together near a single point in high-dimensional space (see section 15.2). To illustrate the stereotyped differential-expression pattern within this bicluster, we replot the bicluster at the top of Fig 4, and below we plot the control data – reorganized to reveal the differential-expression. While there are some control-patients (towards the bottom of the picture) that exhibit similar expression-levels to the case-patients from the bicluster, the majority do not.

This statement can be quantified as follows: Let's define $v \in \mathbb{R}^n$ to be the dominant right-principal-compont of this bicluster, and let c_j be the pearson's-correlation between the jth-patient in the bicluster and v (recall that the pearson's correlation is a measure of correlation that is normalized to lie within -1 and $+1$). If we use the value c_j as a measure of 'alignment', most of the rows in the bicluster are aligned (with the stereotyped pattern) at a value of 75% or more. By contrast, most of the rows in the control-matrix are aligned with a value of -50% to $+10\%$; only 1 of the 166 control-patients has an alignment greater than 75%. This statement can be further quantified as follows: The distribution of alignments c_j for the patients in the bicluster is significantly different than the distribution of alignments for the controls; the AUC (i.e., area under the receiver-operator-characteristic curve) for these two distributions is $> 99\%$, meaning that there is a $> 99\%$ chance that a randomly drawn patient from the bicluster will have a higher alignment than a randomly drawn control. Note that this AUC only reveals that the gene-expression pattern is significantly different between patients within and outside this bicluster. This AUC by itself does not imply that this bicluster is statistically significant or biologically relevant. Put another way, this AUC only implies a high prediction accuracy when discriminating cases *within* the bicluster from the controls; this AUC does not necessarily translate into high case/control prediction accuracy overall.

[1]found at `http://www.ncbi.nlm.nih.gov/geo/query/acc.cgi?acc=GSE48091`

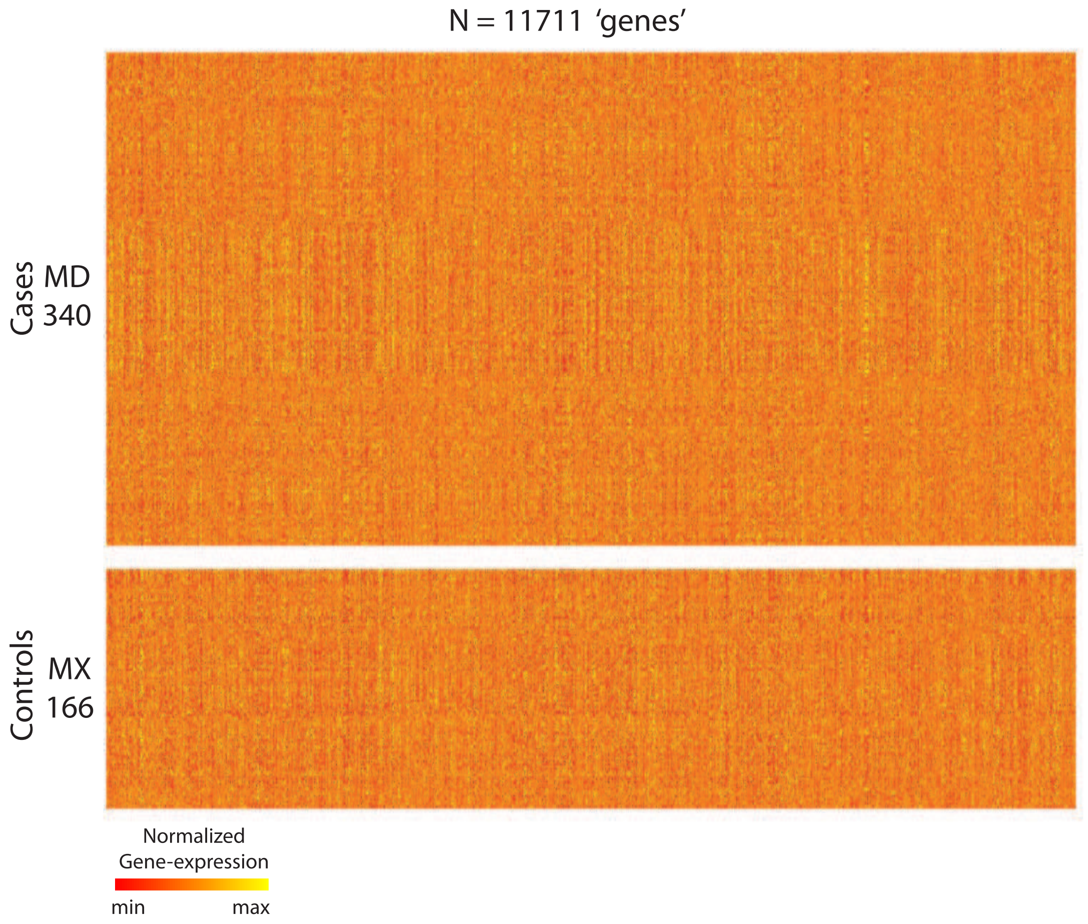

Figure 2: This figure illustrates the GSE48091 gene-expression data-set used in Example-1a. Each row corresponds to a patient, and each column to a 'gene' (i.e., gene-expression measurement): the color of each pixel codes for the intensity of a particular measurement of a particular patient (see colorbar to the bottom). $M_D = 340$ of these patients are cases, the other $M_X = 166$ are controls; we group the former into the case-matrix 'D', and the latter into the control-matrix 'X'. The original data-set had 28499 genes; here we focus only on the $N = 11711$ genes that are each differentially-expressed with respect to the case- and control-populations at a significance level of $p < 0.05$.

Based on the results above, we are lead to ask: How statistically-significant is this bicluster, and is it biologically relevant? As described later on in section 14.2 and Figs 61 and 62, this bicluster has a P-value of $\lesssim 0.002$, which we obtain by comparing it against the distribution of biclusters obtained under a suitable 'label-shuffled' null-hypothesis (i.e., formed from shuffling the case-vs-control labels). This level of statistical significance implies that this signal is 'real', and suggests that many of the genes implicated in this bicluster may be important for distant metastatic disease.

If this bicluster were indeed biologically-relevant, then we would expect many of these genes to serve similar functions or affect the same pathway. This is certainly the case: We used 'Seek' [$\mathcal{M}$42] to perform a gene-enrichment analysis on these $n = 3984$ genes. Using the '`go_bp_iea`' ontology, this gene-enrichment analysis revealed a significant enrichment for processes related to mitosis (p=2e-10), chromosome-segregation (p=3e-9), cell division (4e-8), DNA-dependent-DNA-replication (p=4e-5), spindle-organization (p=2e-6), microtubule organization (p=1e-4) and many more. A full list can be found in the attached worksheet 'S1 Data'. Each page of this worksheet lists the enrichment results using one of the 11 different gene-ontology databases available within the 'Seek' software.

Example-1b: biclustering the remaining genes

Now we'll look at the remaining $N = 28449 - 11711 = 16738$ genes. By construction, these genes are not significantly differentially-expressed with respect to the case- and control-populations; such genes are often ignored by many conventional analyses. These remaining genes are shown in Fig 5, where again each column (i.e., gene) is normalized to have median 0 across the patient-population.

Our goal will be to find a bicluster within the case-population. Because we don't expect any of the genes in this population to be differentially-expressed, we will look for a bicluster that is 'low-rank'. That is to say, we'll look for a subset of genes that are co-expressed (i.e., correlated) across a subset of the patients. It is important to note, however,

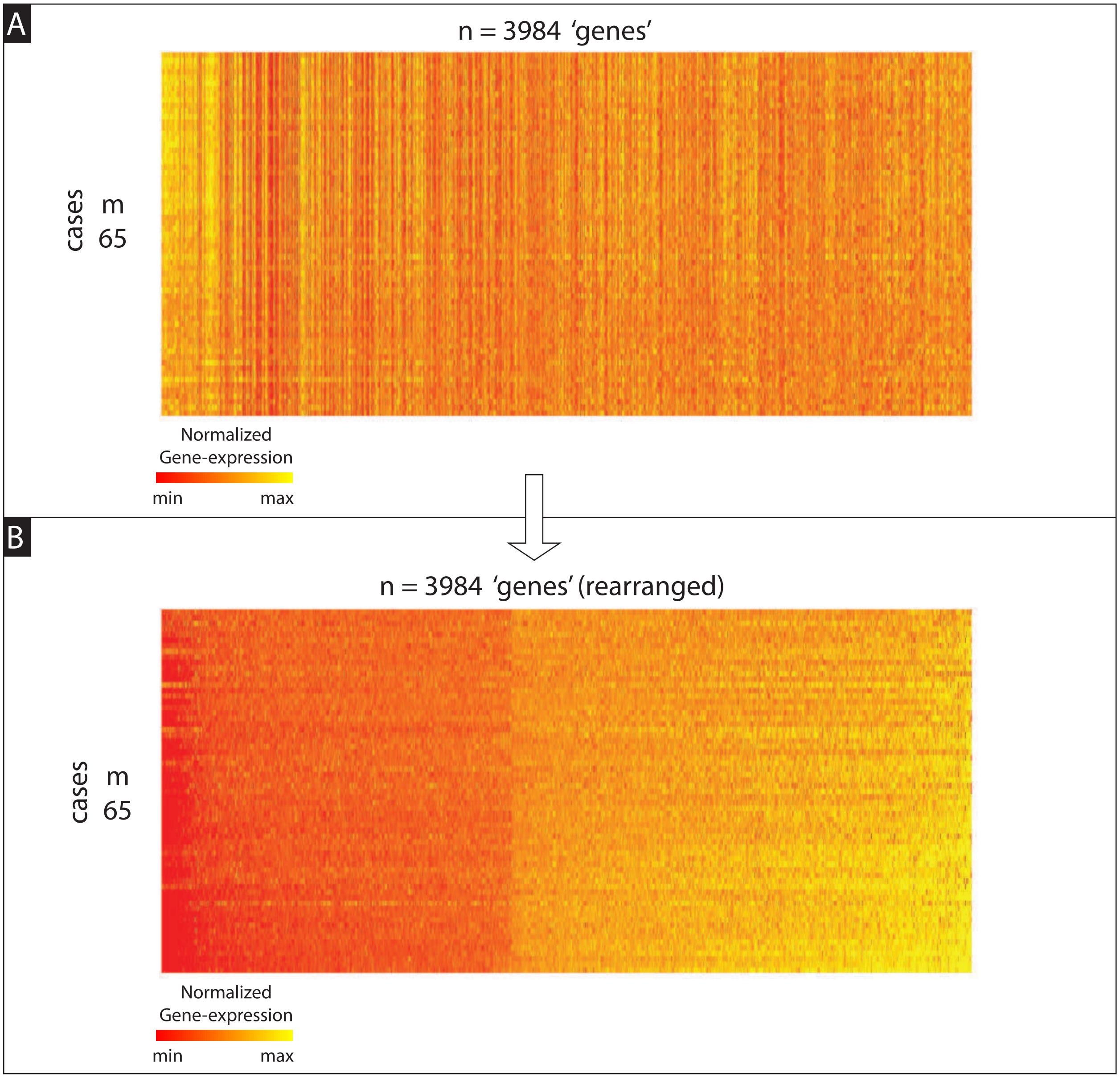

Figure 3: Both panels illustrate the same submatrix (i.e., bicluster) drawn from the full case-matrix shown at the top of Fig 2. This bicluster was found using our control-corrected biclustering algorithm (described in sections 15.2). In Panel-A we represent this bicluster using the row- and column-ordering given by the output of our algorithm. This ordering has certain advantages, which we'll discuss later on, but does not make the differential-expression pattern particularly clear to the eye. Thus, to show this differential-expression more clearly, we present the bicluster again in Panel-B, except this time with the rows and columns rearranged so that the coefficients of the first principal-component-vector change monotonically. As can be seen, there is a striking pattern of differential-expression across the 3984 genes for the 65 cases shown.

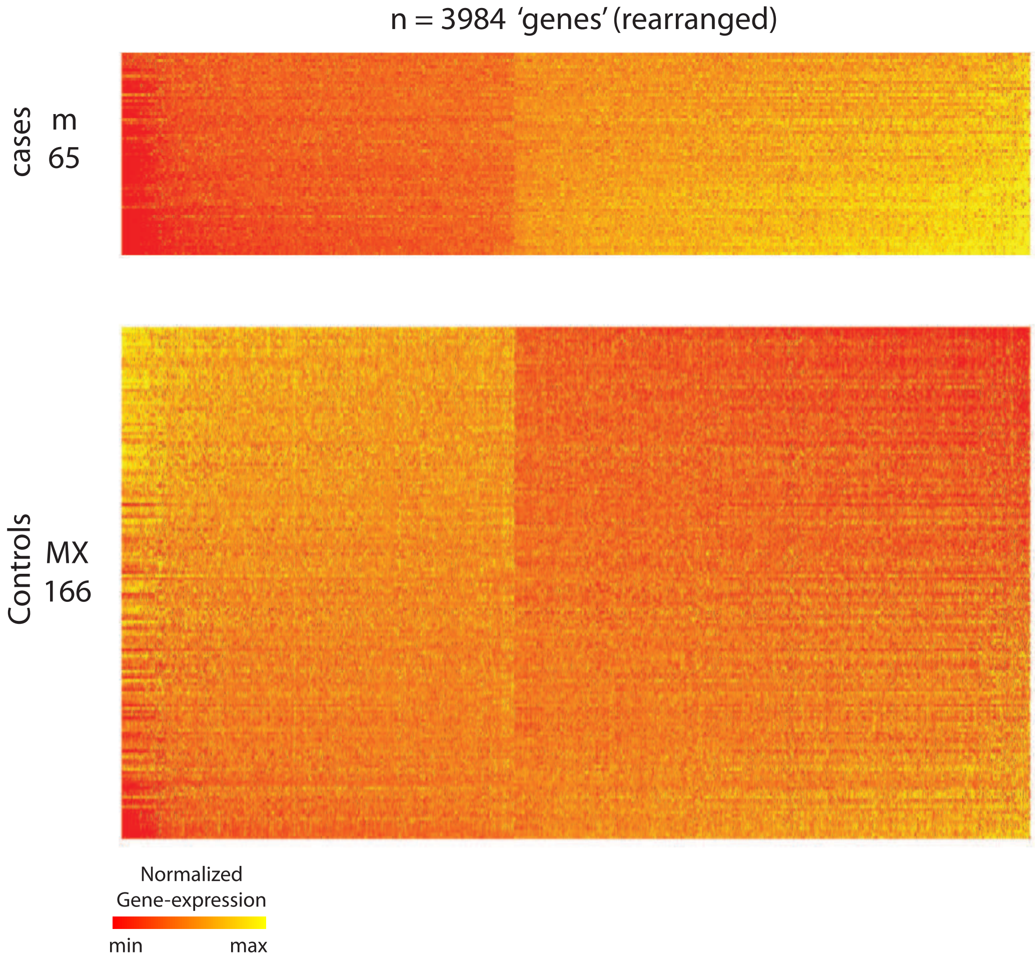

Figure 4: This shows the bicluster of Fig 3B on top, and the rest of the controls from Fig 2 on the bottom. The control-patients have been rearranged in order of their correlation with the co-expression pattern of the bicluster. Even though one of the controls (i.e,. $\sim 1/166$) exhibit a coexpression pattern comparable to that expressed by the bicluster, the vast majority do not.

that many genes are correlated across the entire population — including both the cases and controls. In other words, this data-set includes a few 'low-rank clusters' which are not specific to either the cases or the controls. Due to these non-specific correlations, the largest low-rank submatrices within this kind of gene-expression data are typically not informative of case-control status — these submatrices will simply include all the genes that are expressed in a coordinated fashion across the entire population.

Our objective will be somewhat more specific. We'll search this data-set for case-specific biclusters – namely subsets of genes that are structured in some way across a significantly large subset of the case-patients, while not being similarly structured across the control-population. To achieve this goal we will run our control-corrected loop-counting algorithm (described in section 8). As usual, for this (and all subsequent examples), we set the internal parameter $\gamma \leq 0.05$.

Shown in Fig 6 is an illustration of a large bicluster – comprising $m = 45$ of the $M_D = 340$ cases and $n = 793$ of the $N = 16738$ genes, detected using our loop-counting algorithm. This bicluster consists of genes that, taken individually, are neither significantly over-expressed nor under-expressed – relative to the control population. Instead, these 793 genes are significantly co-expressed (i.e., either strongly correlated or anti-correlated) across a significant fraction of the case population (in this case $45/340 \sim 13\%$ of the cases), without being as significantly co-expressed across a comparable fraction of the control population. While a little difficult to see in Fig 6A, we've rearranged the bicluster in Fig 6B to reveal its co-expression pattern; each patient in the bicluster is either strongly correlated or anti-correlated with this stereotyped pattern. We will later refer to this kind of bicluster as a 'low-rank' bicluster; in this case the rank is approximately 1.

This statement can be quantified as follows: Similar to Example-1a, we'll define $v \in \mathbb{R}^n$ to be the dominant right-principal-compont of this bicluster, and let c_j be the pearson's-correlation between the jth-patient in the bicluster and v. This time, however, we'll use the absolute-value $|c_j|$ as a measure of 'alignment'; we take absolute-values to include both strong positive- and negative-correlations in our definition of co-expression. When using this definition of alignment, most of the rows in the bicluster are aligned (with the stereotyped pattern) at a value of 90% or more. By contrast, most of the rows in the control-matrix are aligned with a value of 0% to 50%; only 3 of the 166 control-patients have an alignment greater than 90%.

To illustrate the stereotyped co-expression pattern within this bicluster, we replot the bicluster at the top of Fig 7, and below we plot the control data – reorganized to reveal the co-expression. While there are some control-patients (towards the top and bottom of the control-matrix) that exhibit similar alignment to the case-patients from the bicluster, the majority do not. This statement can be further quantified: The distribution of alignments $\|c_j\|$ for the patients in the bicluster is significantly different than the distribution of alignments for the controls; the AUC for these two distributions is $> 98\%$. As before, this AUC only reveals that the gene-expression pattern is significantly different between patients within and outside this bicluster, and does not imply that this bicluster is statistically significant or biologically relevant.

Using the same methodology as before, we can ask whether or not this bicluster is statistically-significant, and whether or not it is biologically relevant. As shown in Figs 57 and 58 in section 14.2, this bicluster has a P-value of $\lesssim 0.008$ (which, as before, we obtained by comparing this bicluster against the distribution of biclusters obtained under a 'label-shuffled' null-hypothesis). Using the 'go_bp_iea' ontology in 'Seek' to perform gene-enrichment-analysis on the $n = 793$ genes in this bicluster, we find significant enrichment for mitosis (p=2e-9), DNA-replication (3e-8), chromosome segregation (p=2e-5) and many more; including several pathways that are likely to play a role in the development of cancer. A full list can be found in the attached worksheet 'S2 Data'. Each page of this worksheet lists the enrichment results using one of the 11 different gene-ontology databases available within the 'Seek' software.

We remark that the $n = 793$ gene-expression measurements for the bicluster shown in Fig 6A are completely distinct from the $n = 3984$ differentially-expressed gene-expression-measurements shown in Fig 3A. The patient-subsets are also largely distinct; in fact, the $m = 45$ patients shown in Fig 6A and the $m = 65$ patients shown in Fig 3A have an overlap of only 4, significantly less than one would expect by chance ($p < 0.04$).

1.3 Additional information for Example-2: Gene expression analysis

Our second example is taken from the GSE17536 data-set available from the gene-expression-omnibus[2] uploaded in 2009. See the supplementary-tutorial 'S1 Source Code' for the matlab source code, as well as a full description of how this data-set was pre- and post-processed. This tutorial can be used to reproduce the results in this section (using the 'n2x' normalization convention), and the source code can be used to bicluster many other gene-expression data sets as well (including the GSE48091 set shown previously).

The subset of data that we use comprises $N = 17942$ gene-expression measurements (i.e., 'genes') collected across 175 patients, each diagnosed with colorectal-cancer. Of these patients, $M_D = 55$ patients have alread died, with colorectal-cancer determined to be the significant cause-of-death. The remaining $M_X = 120$ patients either have not yet died (as of 2009), or have died of other causes.

Similar to the original research from which this data is drawn, we'll try to find signals that are related to mortality [3, 2]. With this objective in mind, we'll use the cause-of-death to divide our patient population into cases and controls, respectively. While using the cause-of-death as a case-control classification is far from ideal [4], we'll proceed under the

[2]found at http://www.ncbi.nlm.nih.gov/geo/query/acc.cgi?acc=GSE17536

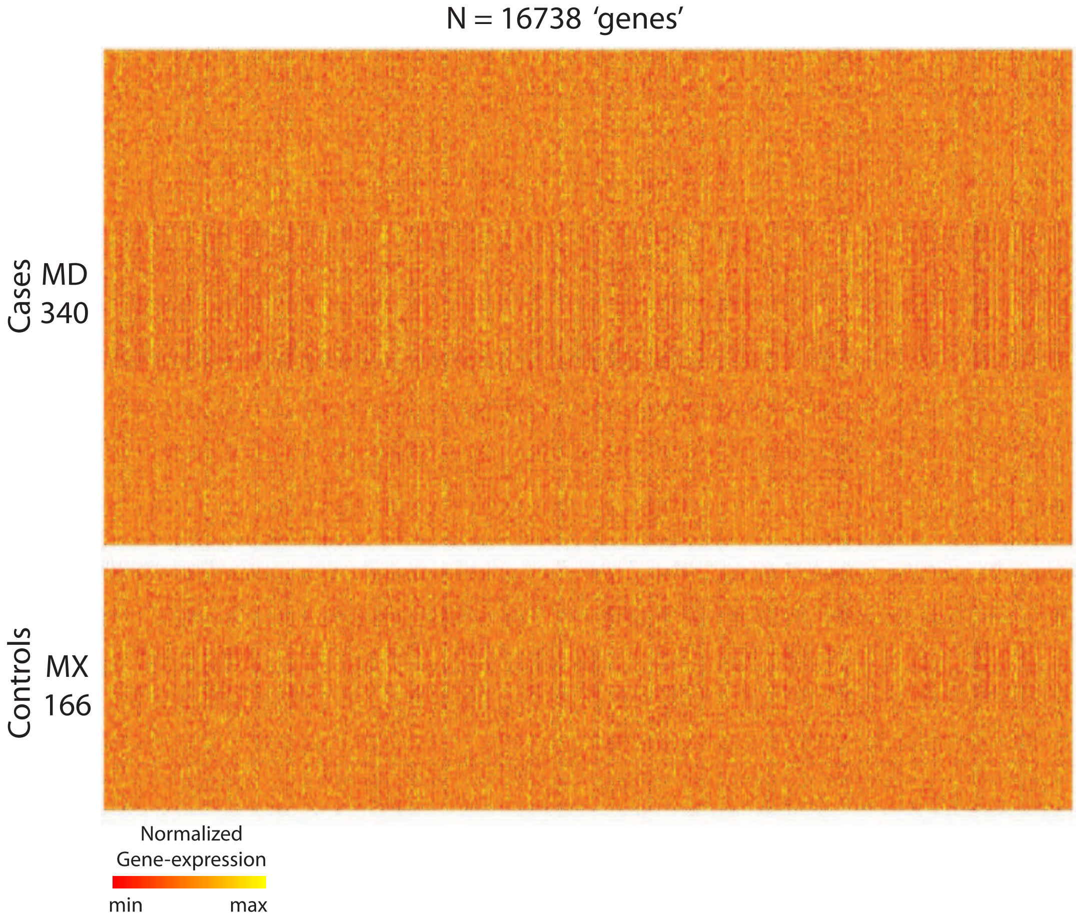

Figure 5: This figure illustrates the GSE48091 gene-expression data-set used in Example-1b (see Example-A in the main-text). The format is similar to Fig 2, except that this time we illustrate the $N = 16738$ genes that are *not* significantly differentially-expressed with respect to the case- and control-populations (i.e., we only include those genes which were excluded in Fig 2).

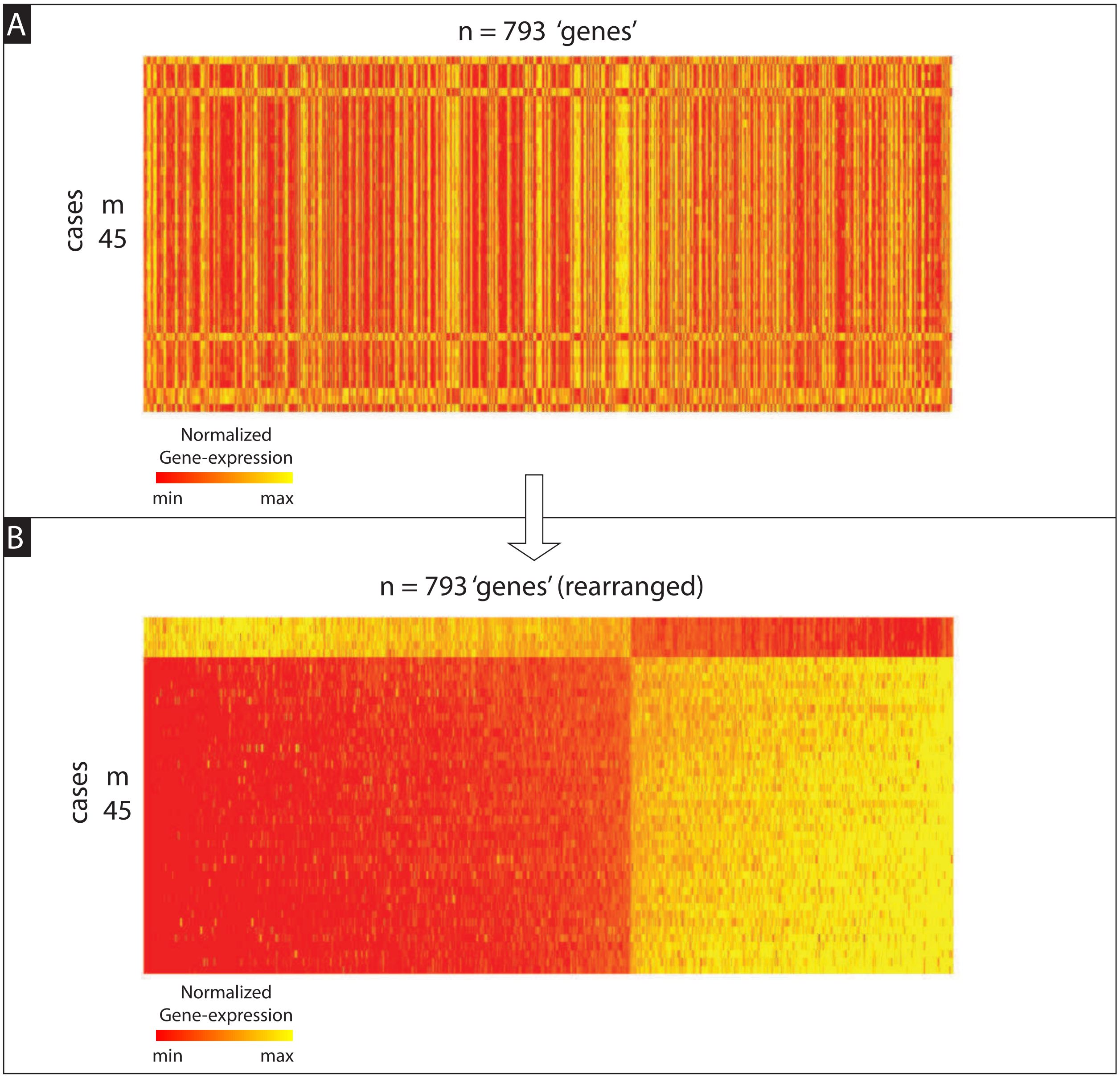

Figure 6: Both panels illustrate the same submatrix (i.e., bicluster) drawn from the full case-matrix shown at the top of Fig 5. This bicluster was found using our control-corrected biclustering algorithm (described in sections 8). The format is similar to Fig 3, except that this time the bicluster includes fewer genes and patients. Nevertheless, there is a striking pattern of differential-expression across the 793 genes for the 45 cases shown. As we discuss in the text, this bicluster is largely distinct from the bicluster shown in Fig 3; the genes are completely distinct, and the patient-overlap is significantly lower than that expected by chance.

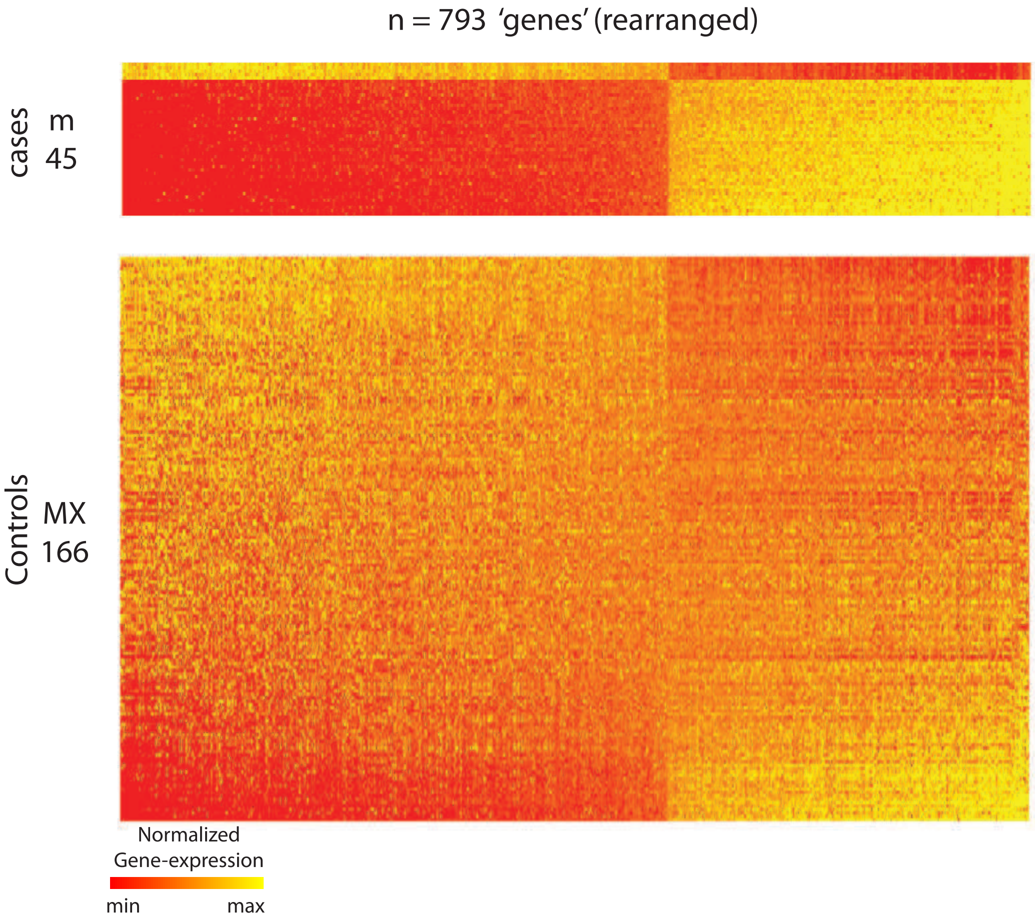

Figure 7: This shows the bicluster of Fig 6B on top, and the rest of the controls from Fig 5 on the bottom. The control-patients have been rearranged in order of their correlation with the co-expression pattern of the bicluster. Even though a few of the controls (i.e,. $\sim 3/166$) exhibit a coexpression pattern comparable to that expressed by the bicluster, the vast majority do not.

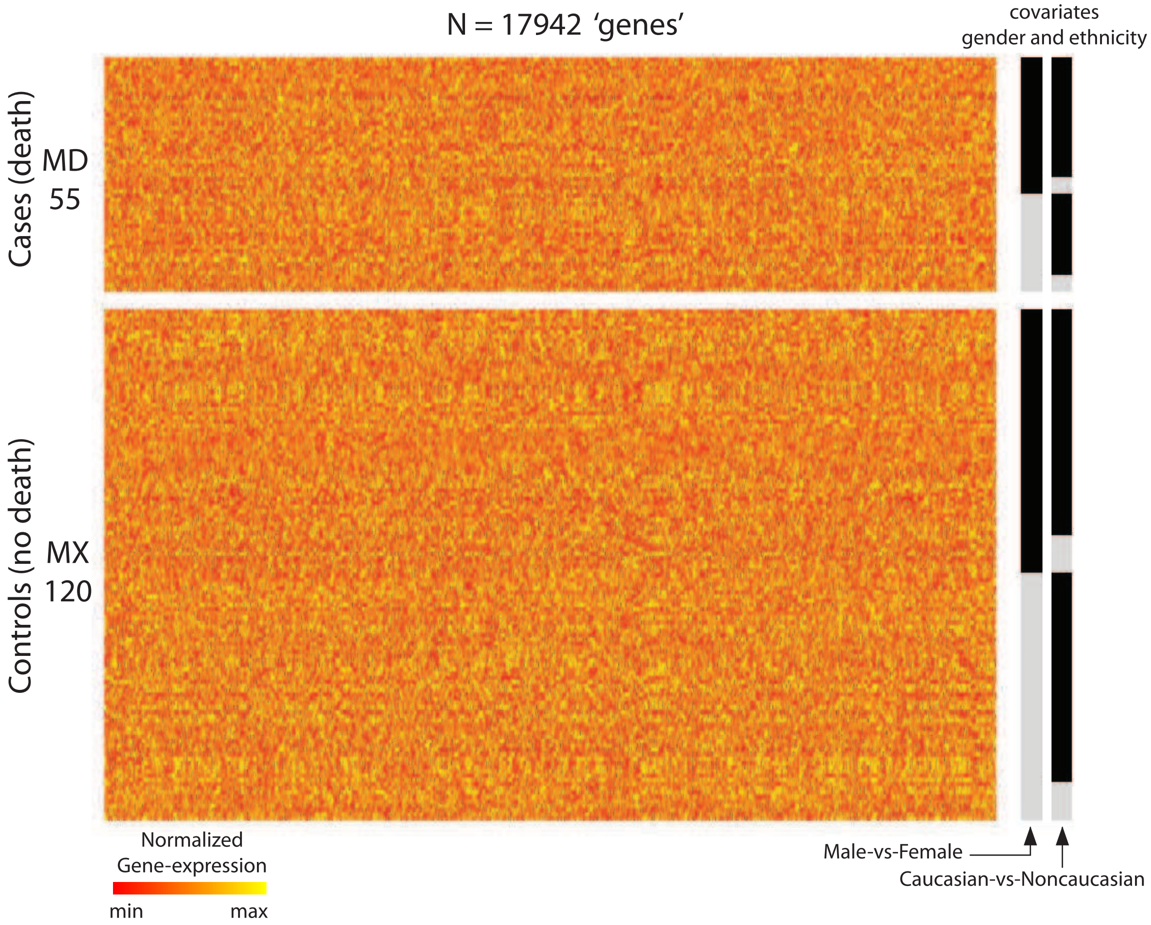

Figure 8: This figure illustrates the GSE17536 gene-expression data-set used in Example-2. The format is similar to Fig 5. For this data set only $M_D = 55$ of the patients are cases, the other $M_X = 120$ are controls; we group the former into the case-matrix 'D', and the latter into the control-matrix 'X'. The covariates are shown to the far right, (i.e., grey vs black). Given two binary categories, there are a total of $I_{\text{cat}} = 4$ covariate-categories in total (ranging from caucasian-male to non-caucasian-female). These covariate-categories can be used to further divide the case- and control-populations.

assumption that the cause-of-death is at least somewhat correlated with the severity of cancer. Our hope is that the subset of case-patients (that died of cancer) will be enriched for patients that exhibit disease-related co-expression across some subset of their genes.

The data-set is illustrated in Fig 8. Note that, aside from their gene-expression data, each patient is also endowed with a variety of other characteristics, including their gender and ethnicity, both of which we'll use as covariates in our analysis.

As in section 1.2, we'll search this data-set for case-specific biclusters. These case-specific biclusters will pinpoint genes useful for diagnosis and discrimination between case and control status. While conducting this search, we'll also try and correct for the covariates. That is, we'll try and focus our efforts on biclusters which include a reasonable mixture of genders and ethnicities.

Fig 9 shows a large bicluster – comprising $m = 14$ of the $M_D = 55$ cases and $n = 966$ of the $N = 17942$ genes – that was hidden within the case-matrix and discovered using a covariate-corrected version of our algorithm. This bicluster consists of genes that, taken individually, are neither significantly over-expressed nor under-expressed – relative to the control population. Instead, these 966 genes are significantly co-expressed (i.e., either strongly correlated or anti-correlated) across a significant fraction of the case population (in this case $14/55 \sim 25\%$ of the cases), without being as significantly co-expressed across a comparable fraction of the control population. Similar to the bicluster from Example-1b from section 1.2, this bicluster is a 'low-rank' bicluster of rank approximately 1.

To illustrate that this stereotyped co-expression pattern is indeed case-specific (i.e., not comparably shared across the controls), we replot the bicluster at the top of Fig 10 and below we plot the control data – reorganized in an attempt to reveal co-expression patterns. As one can see, while there are certainly some control patients that exhibit strong correlation or anti-correlation with the stereotyped gene-expression pattern of the bicluster, the majority are not so strongly aligned. Using our definition of $|c_j|$ as alignment (from Example-1b in section 1.2), we see that almost all the rows in the bicluster

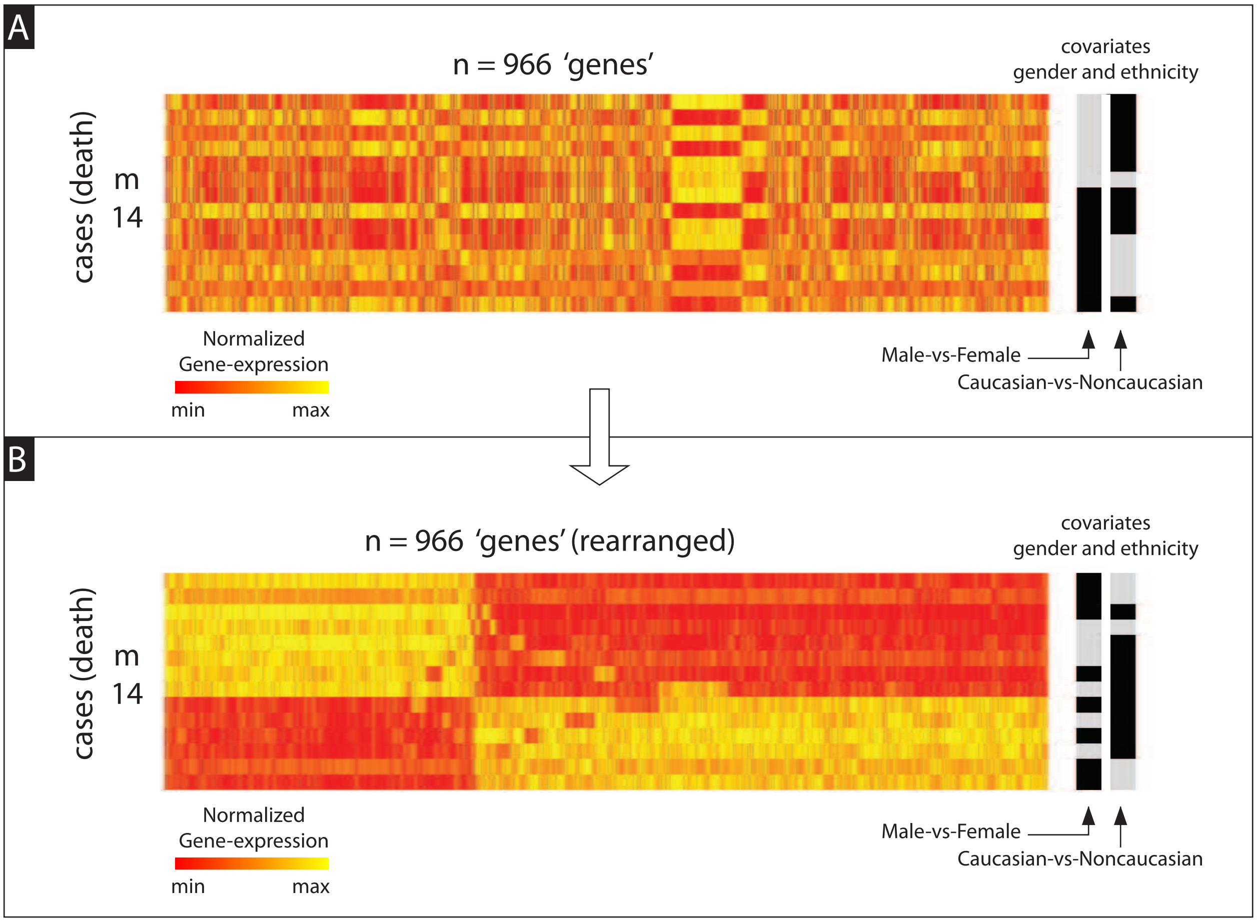

Figure 9: Both panels illustrate the same submatrix (i.e., bicluster) drawn from the full case-matrix shown at the top of Fig 8. This bicluster was found using our covariate-corrected biclustering algorithm (described in sections 8 and 9). In Panel-A we represent this bicluster using the row- and column-ordering given by the output of our algorithm. This ordering has certain advantages, which we'll discuss later on, but does not make the co-expression pattern particularly clear to the eye. Thus, to show this co-expression more clearly, we present the bicluster again in Panel-B, except this time with the rows and columns rearranged so that the coefficients of the first principal-component-vector change monotonically. As can be seen, there is a striking pattern of correlation across the 966 genes for the 14 cases shown.

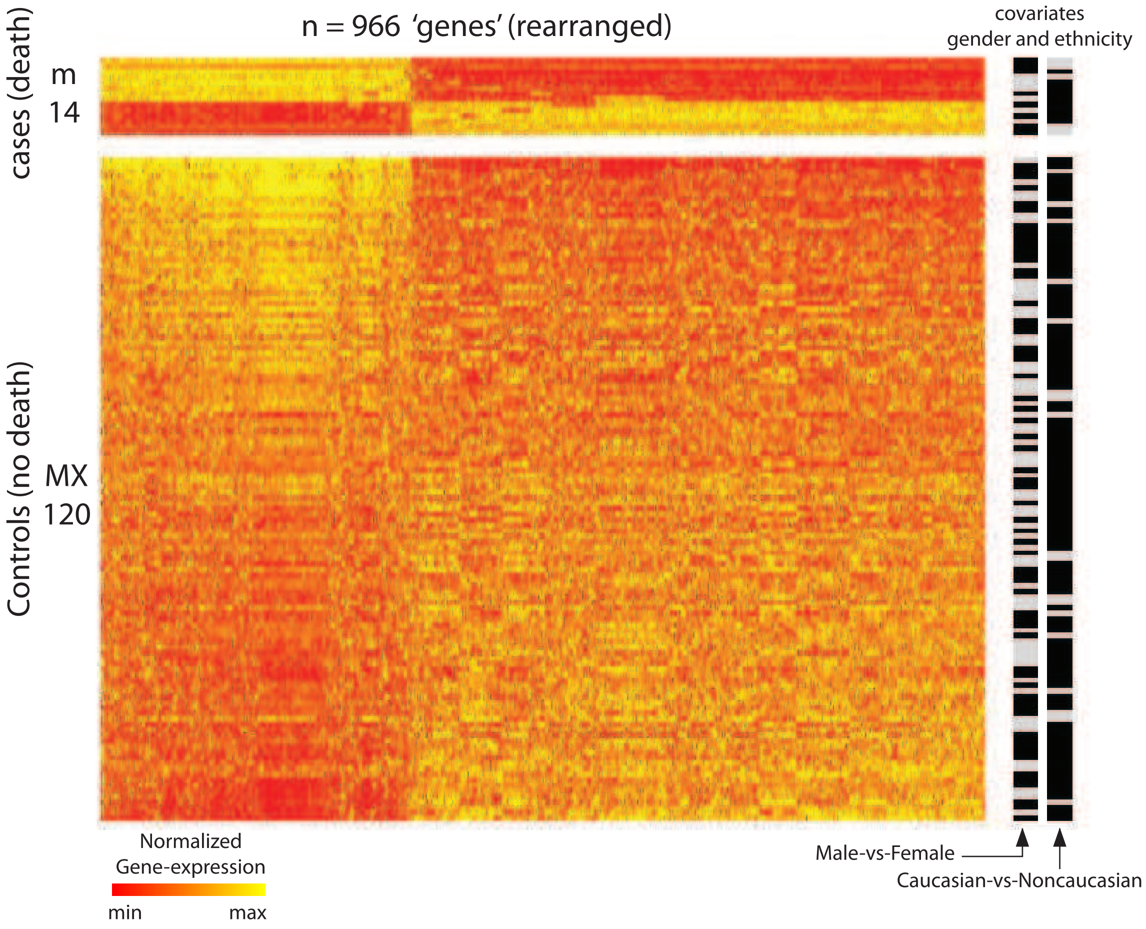

Figure 10: This shows the bicluster of Fig 9B on top, and the rest of the controls on the bottom. The control-patients have been rearranged in order of their correlation with the co-expression pattern of the bicluster. Even though some of the controls (i.e,. $\sim 5/120$) exhibit a coexpression pattern comparable to that expressed by the bicluster, the vast majority do not.

are aligned at a value of 70% or more, whereas only 5 of the 120 controls (i.e., significantly less than 14/55) have an alignment that is greater than 70%; most only exhibit an alignment around 20% − 30%. The distribution of alignments $|c_j|$ for the patients in the bicluster is significantly different than the distribution of alignments for the controls; the AUC for these two distributions is 97%. As before, this AUC only reveals that the gene-expression pattern is significantly different between patients within and outside this bicluster, and does not imply that this bicluster is statistically significant or biologically relevant.

At this point we remark on the covariates. Gender and ethnicity are both covariates that strongly influence the expression levels of many genes throughout the body. It is therefore rather typical for gene-expression data-sets to contain large biclusters driven solely by these covariates. If we were to run our biclustering algorithm without taking these covariates into account, it is likely that we would reveal one of these large covariate-driven biclusters. To account for this we conducted this example using a covariate-corrected version of our algorithm. The covariate-corrected algorithm attempts to find biclusters that are well balanced across the covariate-categories. The algorithm was successful in this case: as seen in Fig 9, the 14 patients that exhibit this co-expression are not all male, nor all female, nor all of one ethnicity. This balance is reassuring, and hints that the signal we are seeing is not driven solely by gender or ethnicity.

Finally, we can ask: How statistically-significant is this bicluster, and is it biologically relevant? As described later on in section 14.2 and Figs 59 and 60, this bicluster has a P-value of ~ 0.027, which we obtain by comparing it against the distribution of biclusters obtained under an appropriate 'label-shuffled' null-hypothesis (i.e., formed from shuffling the case-vs-control labels, while respecting covariate categories). This level of statistical significance implies that this signal is 'real', and suggests that many of the genes implicated in this bicluster may be important for colorectal cancer.

Using the '`go_bp_iea`' ontology in 'Seek' to perform gene-enrichment analysis on the $n = 966$ genes within the bicluster, we find significant enrichment for processes related to mitosis (p=9e-17), chromosome-segregation (p=9e-12), DNA-dependent-DNA-replication (p=9e-9), microtubule organization (p=2e-8), spindle-organization (p=6e-8), RNA-splicing (p=1e-5), mitotic recombination (p=5e-4), and many more, including many pathways that are likely to play a role in the

development of cancer. A full list can be found in the attached worksheet 'S3 Data'. Each page of this worksheet lists the enrichment results using one of the 11 different gene-ontology databases available within the 'Seek' software.

To further probe the potential relevance of this bicluster, we can check to see if the $n = 966$ genes in the bicluster overlap significantly with 'recognized' genes that are already understood to play a role in colon-cancer. For this example we'll generate this latter list by first choosing three of the most well documented genes which influence colon-cancer: MLH1, MSH2 and MSH6 [5]. We then use 'Seek' once again to generate a list of genes that are commonly co-expressed alongside these three cancer-related genes. We then define our list of 'recognized' genes to be all the genes which have a combined co-expression value (with MLH1, MSH2 and MSH6) of at least 0.75. The list of recognized-genes generated this way comprises 1659 of our original 17941 genes. After generating this list, we can check to see if the $n = 966$ genes in our bicluster significantly overlap with the list of recognized-genes. Indeed they do: the intersection is 174, which is significantly higher than chance (p=1e-20).

Given the analysis above, it comes as no surprise that the list of recognized-genes also enriches strongly for the same pathways we saw within our bicluster (i.e., DNA-replication, DNA-repair, mitosis, etc.). While this similarity is encouraging (and further suggests that our bicluster might be biologically relevant), it begs the following question: Could we possibly search for a bicluster within this data set that was distinct from the genes that are already well-recognized? Such a search might unveil previously undiscovered sets of genes that also play a role the pathology of colon cancer for certain subsets of patients. We perform this analysis later on in section 15.1, using this same list of recognized-genes as genetic-controls.

Before we move on to the next example, we add one final comment on the advantages of biclustering. As we mentioned above, biclustering allows us to search for subsets of genes that are coexpressed across only a subset of the patients. Because the biclustering process is capable of ignoring 'noisy' patients that do not participate in the coexpression pattern, it is possible for our algorithm to find gene-subsets that are very tightly related. More traditional algorithms that try to cluster genes (across the entire case-patient population) may not be as successful in highlighting relevant gene-subsets. For example, we can contrast our biclustering approach with a standard logistic-regression analysis (performed on a gene-by-gene basis across the full set of case- and control-patients after rank-normalizing the data). To compare the logistic-regression results with our biclustering, we consider the top $n = 966$ genes with the most significant regression-coefficients (i.e., the $n = 966$ 'top-hits'). These top-hits do not display many significant enrichments for disease-related processes. In fact, the only significant enrichment we found involved a Cytosolic DNA sensing pathway (p=5.6e-4) (using the kegg ontology). Moreover, these top-hits did not significantly overlap with the list of recognized-genes (intersection 91, p-value ~ 0.5). In summary, for this example it seems that a standard logistic-regression analysis does not identify the same kinds of disease-related signals as our biclustering approach.

1.4 Additional Information for Example-3: Genome-Wide-Association-Study (GWAS)

Our third example is a subset of a Genome-Wide-Association-Study used with permission from the Bipolar Disorders Working Group of the Psychiatric Genomics Consortium (PGC-BIP) [6] [3]. This data-set includes $N = 276768$ alleles genotyped across 16577 patients of european ancestry. These patients are drawn from the following studies (i.e., cohorts): `bip_gain_eur_sr-qc`, `bip_dub1_eur_sr-qc`, `bip_top7_eur_sr-qc`, `bip_swa2_eur_sr-qc`, `bip_fat2_eur_sr-qc`, `bip_wtcc_eur_sr-qc`, `bip_edi1_eur_sr-qc`, `bip_uclo_eur_sr-qc`, `bip_stp1_eur_sr-qc` and `bip_st2c_eur_sr-qc` described within the supplementary information of [7] and the supplementary note of [8].

The patients themselves fell into two phenotypic categories: 9752 are neurotypical, whereas the remaining 6825 exhibit a particular psychiatric disorder. For this example we'll try and find a signal within the neurotypical patients that is not shared by those with the disorder; We'll use the phenotypic information to divide the patients into $M_D = 9752$ cases and $M_X = 6825$ controls. Note that, for this example, our nomenclature is non-standard; neurotypical patients are typically referred to as 'controls' and not cases. The reason we deviate from this standard is because, below, we will try to find a bicluster within the neurotypical patients that does not extend to include the remaining patients. In order to remain consistent with our notation and equations in the rest of this manuscript, we will refer to these neurotypical patients as cases, and we will store their information in the case-matrix D.

To select our set of alleles, we considered all the fully-genotyped alleles available for the studies listed above, limiting ourselves to those with a minor-allele-frequency of at least 10%. We used all the alleles that fell within this bound; We did not use imputation or correct for linkage-disequilibrium. In addition to their genotyped data, each patient is also associated with an $N_T = 2$-dimensional vector of 'mds-components' that serve as a continuous-covariate. In this case the continuous-covariate plays the role of a proxy for each patient's genetic ancestry.

Our objective in this situation is similar to Example-1b and Example-2: We would like to search for case-specific biclusters involving subsets of alleles that are structured in some way across a significantly large subset of the case-patients, while not being similarly structured across the control-population. In addition, we'd like to ensure that the

[3]Due to data-usage agreements, only cursory information regarding this data-set will be provided here. A more detailed description of the data-set, as well as the structures we've found within it, will be provided in a later publication.

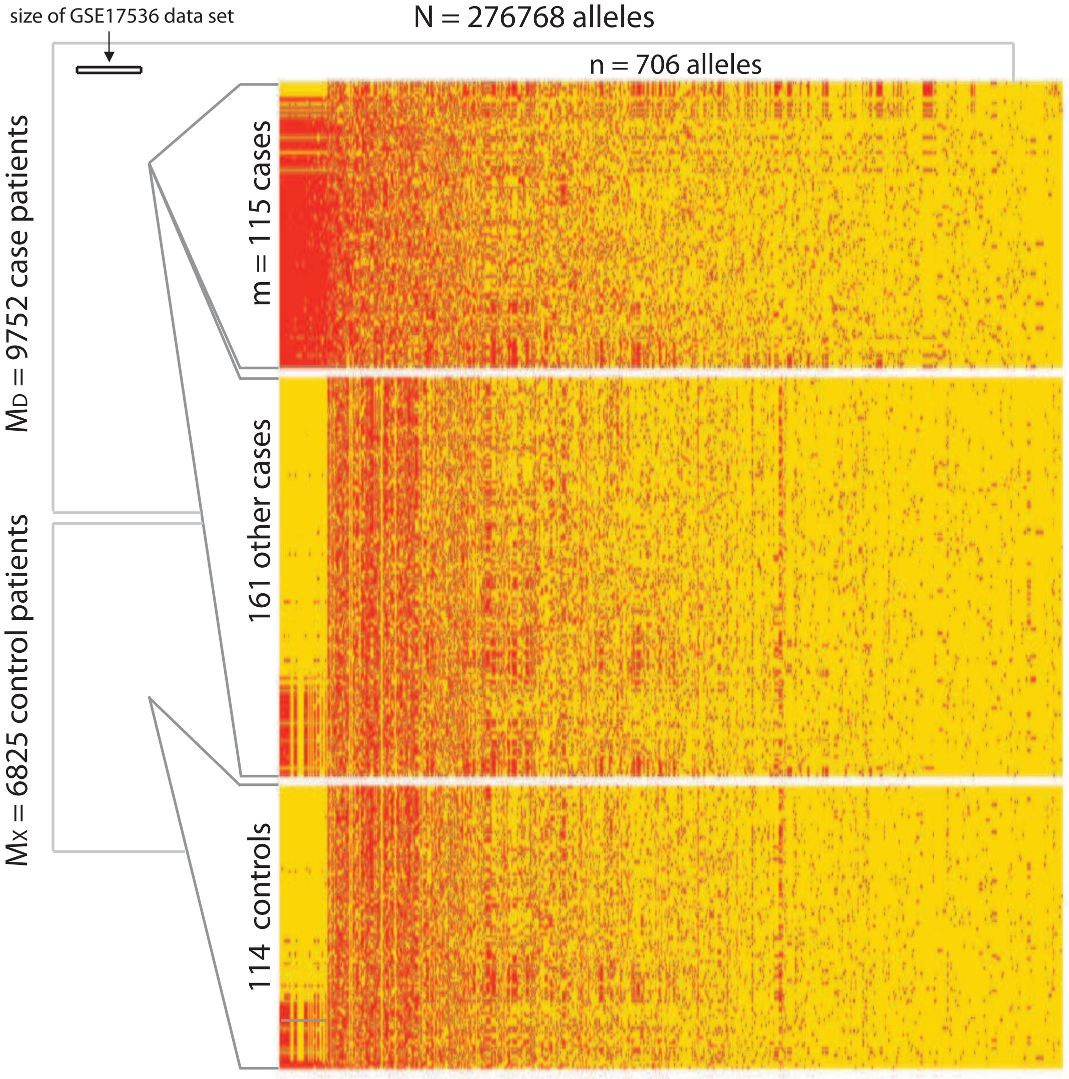

Figure 11: In this figure we illustrate the genome-wide association-study (i.e., GWAS) data-set discussed in Example-3 (see Example-B in the main text). This data-set involves 16577 patients, each genotyped across 276768 genetic base-pair-locations (i.e., alleles). Many of these patients have a particular psychological disorder, while the remainder do not. We use this phenotype to separate the patients into $M_D = 9752$ cases and $M_X = 6825$ controls. The size of this GWAS data-set is indicated in the background of this picture, and dwarfs the the size of the gene-expression data-set used in Example-2 (inset for comparison). At the top of the foreground we illustrate an $m = 115$ by $n = 706$ submatrix found within the case-patients. This submatrix is a low-rank bicluster, and the alleles are strongly correlated across these particular case-patients. The order of the patients and alleles within this submatrix has been chosen to emphasize this correlation. For comparison, we pull out a few other randomly-chosen case-patients and control-patients, and present their associated submatrices (defined using the same 706 alleles) further down.

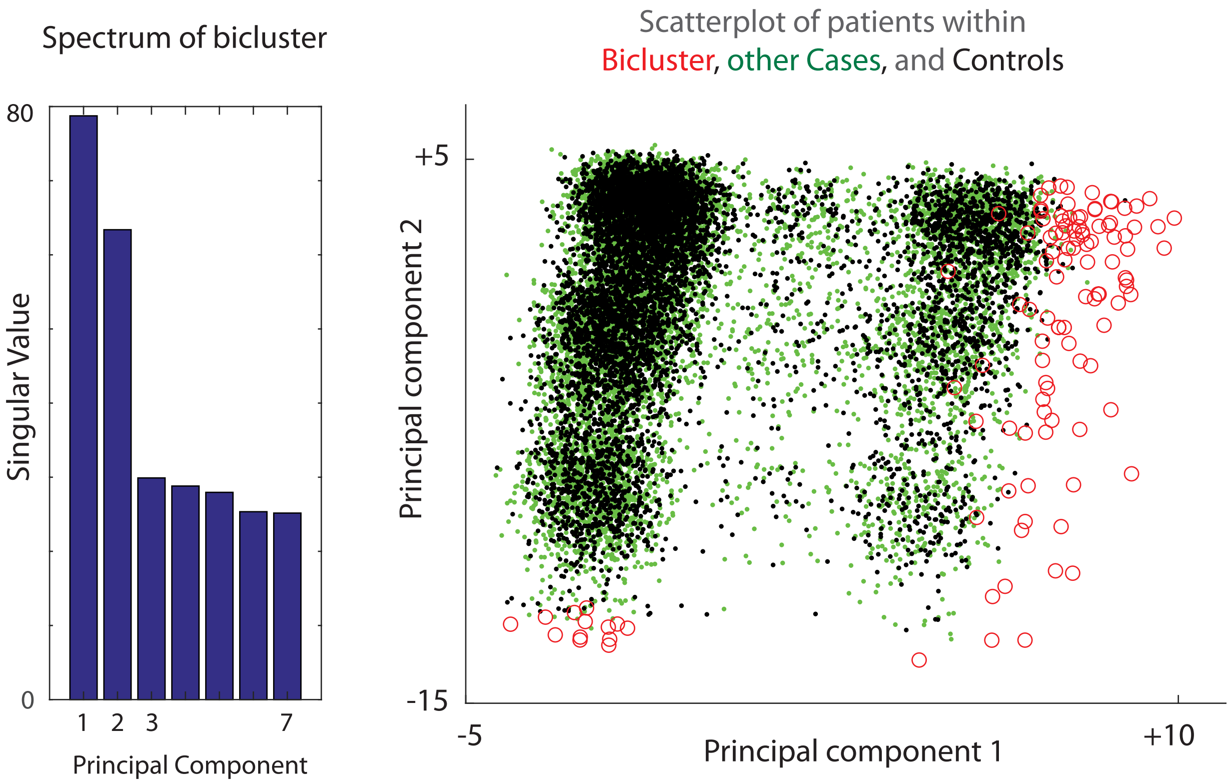

Figure 12: On the left we show the first 7 singular-values of the bicluster shown in Fig 11. Note that the first two singular-values are much larger than the rest; this bicluster is effectively rank-2. On the right we show a scatterplot of the patients in the data-set, as projected onto the first two dominant principal-components of the bicluster. The patients within the bicluster are shown with red circles. The remaining case-patients are shown with green dots, whereas the control-patients are shown with black-dots. Note that the patients within the bicluster mostly lie outside the distribution defined by the other patients, but that the bicluster-patients cannot easily be separated from the rest after projecting onto a straight-line (i.e,. the bicluster is rank-2, but not rank-1).

biclusters we find are well-distributed with regards to the continuous-covariate (i.e., we don't want to focus on a subset of patients that all have the same ancestry).

Fig 11 illustrates the size of this data-set, as well as one case-specific low-rank bicluster which we discovered. For this example we used a version of our algorithm that corrects for continuous-covariates; specifically, we use the '2-sided' covariate-correction described in section 10. As can be seen from Fig 11, the pattern shown within the bicluster is rather different than the pattern exhibited by the typical control. Indeed, as described later on in section 14.2, this bicluster has a P-value of $\ll 0.05$.

What may not be obvious from visual inspection is that this bicluster is essentially 'rank-2'; i.e., the dominant two principal components of this bicluster are large compared to the rest. Put another way, the patients within this bicluster exhibit a second-order correlation across the subset of alleles in the bicluster; a correlation not exhibited by the population at large. We illustrate this second-order structure in Fig 12. As can be seen in Fig 12B, the distribution of patients within the bicluster is markedly different from the distribution of the other patients. Even though this bicluster is essentially rank-2, a substantial amount of the variance is still captured by the first principal-component. Indeed, if we calculate the distribution of alignments across individuals in this bicluster (similar to Example-1b and Example-2) and compare them to the distribution of alignments across the controls, we obtain an AUC of $> 99.75\%$. Just as in our previous examples, we should interpret this AUC as evidence that the allele-pattern exhibited by the patients in the bicluster is indeed significantly different than the allele-pattern exhibited by the remaining patients. That is to say, this AUC only implies a high prediction accuracy when discriminating cases *within* the bicluster from the controls; this AUC does not translate into high case/control prediction accuracy overall.

We remark that the above observations alone do not prove that the bicluster is biologically significant! The patterns we observe within this bicluster could very well be due to a covariate that we did not correct for, or some other artifact of the data-set. One way to substantiate the biological relevance of this bicluster would be to perform a replication study on an independent data-set. Ideally, the projection of a fresh set of patients onto the first two principal-components of the bicluster discovered above should reveal a similarly distinct set of distributions, with a similar proportion of the case-patients falling far from the other patients and lying near the red-circles shown in Fig 12B.

Nevertheless, given the significance-level and strength of the signal within this bicluster, we might expect many of the alleles from the bicluster to affect the same physiological functions. Using 'Seek' once again, we find that the 124 genes involved in these 706 genetic-loci are enriched for many pathways. Using the '`go_mf_iea`' and '`kegg`' ontologies, we find enrichment for: phosphate-ion transmembrane transport (p=1.6e-4), metal-ion transmembrane transport (p=5.7e-4), calcium-ion binding (p=1.2e-3), GTPase-activation (p=2.2e-3), ion-gated channel activity (p=3.3e-3), calcium-ion transport (p=4.3e-3), voltage-gated channel activity (p=1.0e-2), calcium-signaling (p=1.1e-3), long-term-potentiation (p=1.1e-3), glutamatergic-synapses (p=7e-3) and many others. A full list can be found in the attached worksheet 'S4 Data', with a format similar to that of 'S1 Data'. Because this bicluster was found within the neurotypical patients, it is possible that these genes play a protective role in delaying the development or onset of the psychiatric disorder associated with this data-set.

In addition to finding this bicluster, our covariate-corrected algorithm has also successfully ensured that this bicluster is balanced with regards to the continuous-covariate. This balance is illustrated in Figs 13 and 14, which shows the joint-distribution (across patients) of the first two mds-components as our algorithm proceeds. If we were to run our algorithm *without* correcting for the continuous-covariates, then we would find spurious biclusters involving patients that were highly concentrated in just a few regions of covariate-space (see Fig 15).

We remark that the patients in this data-set actually have more than just two mds-components; there are also higher-order mds-components numbered 3, 4, 5, etc. that may also correlate with the patients' ancestry. For this particular example, however, the patients are all of european ancestry; there is not too much structure within the higher-order mds-components. Thus, even though we do not explicitly control for the higher-order mds-components, our algorithm nevertheless produces biclusters which are balanced with regards to these higher-order mds-components. We illustrate this balance by showing the joint-distribution of the mds-components 3+4 in Figs 16 and 17. While this joint-distribution alone does not certify that our algorithm has maintained a balance across all higher-order mds-components, we do indeed see a qualitatively similar phenomenon for the joint-distributions of the mds-components 1+3, 1+4, 2+3 and 2+4. A similar story holds for mds-components 5, 6, etc., which are even more unstructured than components 3 and 4.

It so happens that, for this data-set, each patient is not only associated with the continuous-covariate mentioned above, but also a categorical-covariate; each patient is drawn from one of $I_{\text{cat}} = 10$ studies. These different studies were carried out by different research groups within the PGC, involving different experimental designs and patient populations. Much like the continuous-covariate, these different studies can give rise to spurious signals within the data-set. While we could attempt to correct for both continuous- and categorical-covariates simultaneously, it turns out that many of the individual studies involve patient cohorts that are localized in covariate-space. In other words, for this example the continuous-covariate and categorical-covariate are correlated. Consequently, a bicluster that is balanced with respect to the continuous-covariate will typically involve patients drawn from many different studies. As a result, even though we chose to correct for continuous-covariates alone, this correction was sufficient to ensure that the bicluster found was rather well balanced with respect to study (in addition to being balanced with respect to the continuous-covariate). This balance is demonstrated in Figs 18 and 19.

In the following few sections we describe our algorithm in more detail. We start out with the simplest possible situation, explaining when we expect our algorithm to work and comparing its performance to that of a simple spectral method. Afterwards, we explain how to generalize our algorithm to incorporate controls, covariates and sparse data. Finally, we comment on some practical considerations, such as finding p-values for a bicluster and delineating the boundaries of a bicluster.

2 Simple case: D only

In the simplest situation there are no controls, covariates, or sparsity considerations, and we are tasked with finding low-rank biclusters within an $M \times N$ case-matrix D. In this case our algorithm reduces to the following very simple iteration:

Step 0 Binarize D, sending each entry to either $+1$ or -1, depending on its sign (i.e., $D = \text{sign}(D)$);

Step 1 Calculate row-scores and column-scores. In their simplest form these are: $Z_{\text{ROW}} = \text{diag}(DD^{\intercal}DD^{\intercal})$, and $Z_{\text{COL}} = \text{diag}(D^{\intercal}DD^{\intercal}D)$;

Step 2 Restrict attention to the row-indices for which Z_{ROW} is largest (i.e., most positive) and the column indices for which Z_{COL} is largest – e.g., throw away the rows/columns for which Z_{ROW} and Z_{COL} are smallest (i.e., most negative).

Step 3 Go back to step 1.

As a consequence of this simple iteration, the output of the algorithm is a listing of row- and col-indices in the order that they were eliminated. Those rows and columns which are retained the longest are most likely to be part of a low-rank

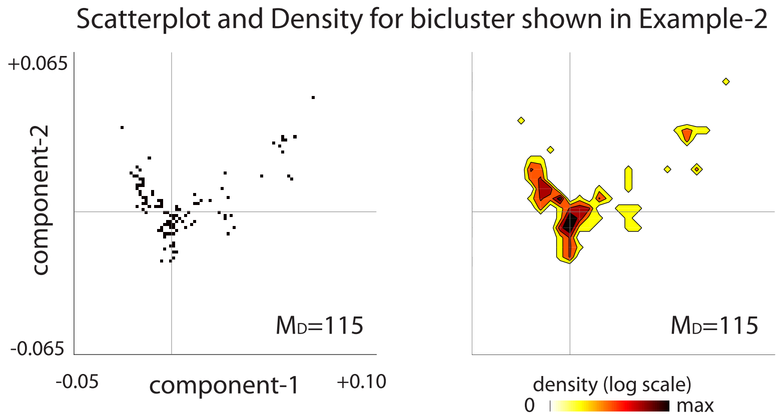

Figure 13: Joint-distribution (across patients) of continuous-covariates for the bicluster shown in Example-3. As mentioned in the introduction, our algorithm proceeds iteratively, removing rows and columns from the case-matrix until there are none left. One of our goals is to ensure that, during this process, our algorithm focuses on biclusters which involve case-patients that are relatively well balanced in covariate-space. On the left we show a scatterplot illustrating the 2-dimensional joint-distribution of covariate-components across the remaining $m = 115$ case-patients within the bicluster shown in Example-3 (i.e., Fig 11). The horizontal and vertical lines in each subplot indicate the medians of the components of the covariate-distribution. On the right we show the same data again, except in contour form (note colorbar). The continuous-covariates remain relatively well-distributed even though relatively few case-patients are left (compare with Fig 14).

bicluster. After finding the first bicluster in this manner, the entries of D corresponding to this first bicluster can be scrambled (i.e., destroying their low-rank structure), and the next bicluster can be found by running the algorithm again[4].

There are several positive features of this algorithm:

- It works: Specifically, this algorithm solves the 'planted-bicluster' problem with high probability. That is to say, if the case-matrix D is a large random matrix containing a hidden low-rank bicluster 'B' of size $m_B \times n$, then this algorithm will almost always find B as long as the spectrum of B decays sufficiently quickly, and m_B and n are larger than $\sqrt{M_D}$ and $\sqrt{N}$, respectively. We discuss this in more detail below.
- It's fast: Specifically, this algorithm requires only a handful of matrix-multiplications per iteration. Furthermore, the binarization step allows for very fast matrix-matrix multiplication that does not use floating-point operations. If desired, the recalculation of row- and col-scores in each iteration can be replaced by a low-rank update (see section 3.1), reducing the maximum total computation time across all iterations to $O\left(M_D N \max\left(M_D, N\right)\right)$ – asymptotically equivalent to matrix-matrix multiplication.
- It's easy: Specifically, this algorithm has few-to-no parameters. Notably, the user does not need to specify the number, size or rank of the biclusters beforehand. As long as any biclusters are sufficiently low-rank (e.g., rank $l = 1, 2, 3$) and sufficiently large (see the first point), then this algorithm will find them.
- It's generalizable: Specifically, this algorithm can easily be modified to account for controls and/or covariates. We'll discuss this after we explain why this algorithm works.

The reason this algorithm is successful is because, when calculated across a large matrix, the row- and col-scores Z_{ROW} and Z_{COL} are likely to be relatively high for row- and col-indices that correspond to any embedded low-rank submatrices, and relatively low for the other indices.

To see why this might be true, consider a random binary $M_D \times N$ D-matrix (with $+1/-1$ entries, as shown in Fig 20A) containing an embedded low-rank $m_B \times n$ bicluster B (tinted in pink). To aid discussion, let's also assume that this embedded low-rank bicluster is perfectly rank-1 (i.e., with no noise). As seen in Fig 20A, this means that the rows and columns of the embedded bicluster are perfectly correlated; any pair of rows or columns are either equal or are negatives of one another. As we'll see in a moment, this structure can be used to identify the bicluster.

To begin with we'll look at 2×2 submatrices of this D-matrix, and for brevity we'll refer to these 2×2 submatrices as 'loops'. Each loop is described by two row-indices and two col-indices which, together, pick out four entries within the matrix D. Four loops are indicated in Fig 20A, each with a rectangle whose corners correspond to their 4 entries. Some loops either don't pass through the bicluster at all, or have only 1 or 2 corners within the embedded bicluster (blue rectangles). Other loops are entirely contained within the bicluster (red rectangle).

[4] We discuss how exactly we delineate a bicluster later on in section 14.3.

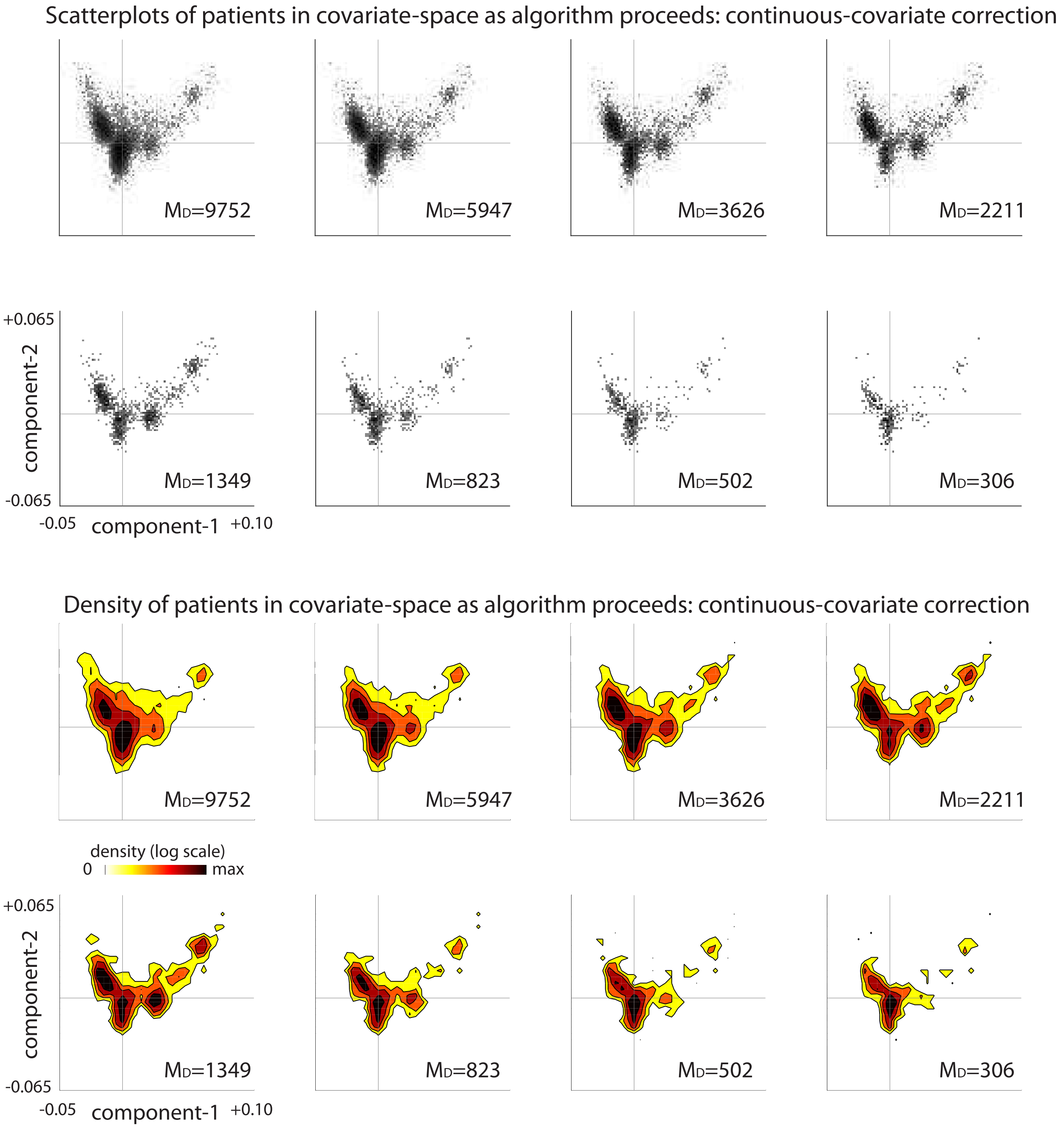

Figure 14: On top we show several scatterplots, sampling from different iterations as our algorithm proceeds. Each scatterplot illustrates the 2-dimensional joint-distribution of covariate-components across the remaining case-patients (i.e., the remaining M_D). The horizontal and vertical lines in each subplot indicate the medians of the components of the covariate-distribution. Below we show the same data again, except in contour form (note colorbar). Note that the covariate-distribution remains relatively well-distributed as the algorithm proceeds.

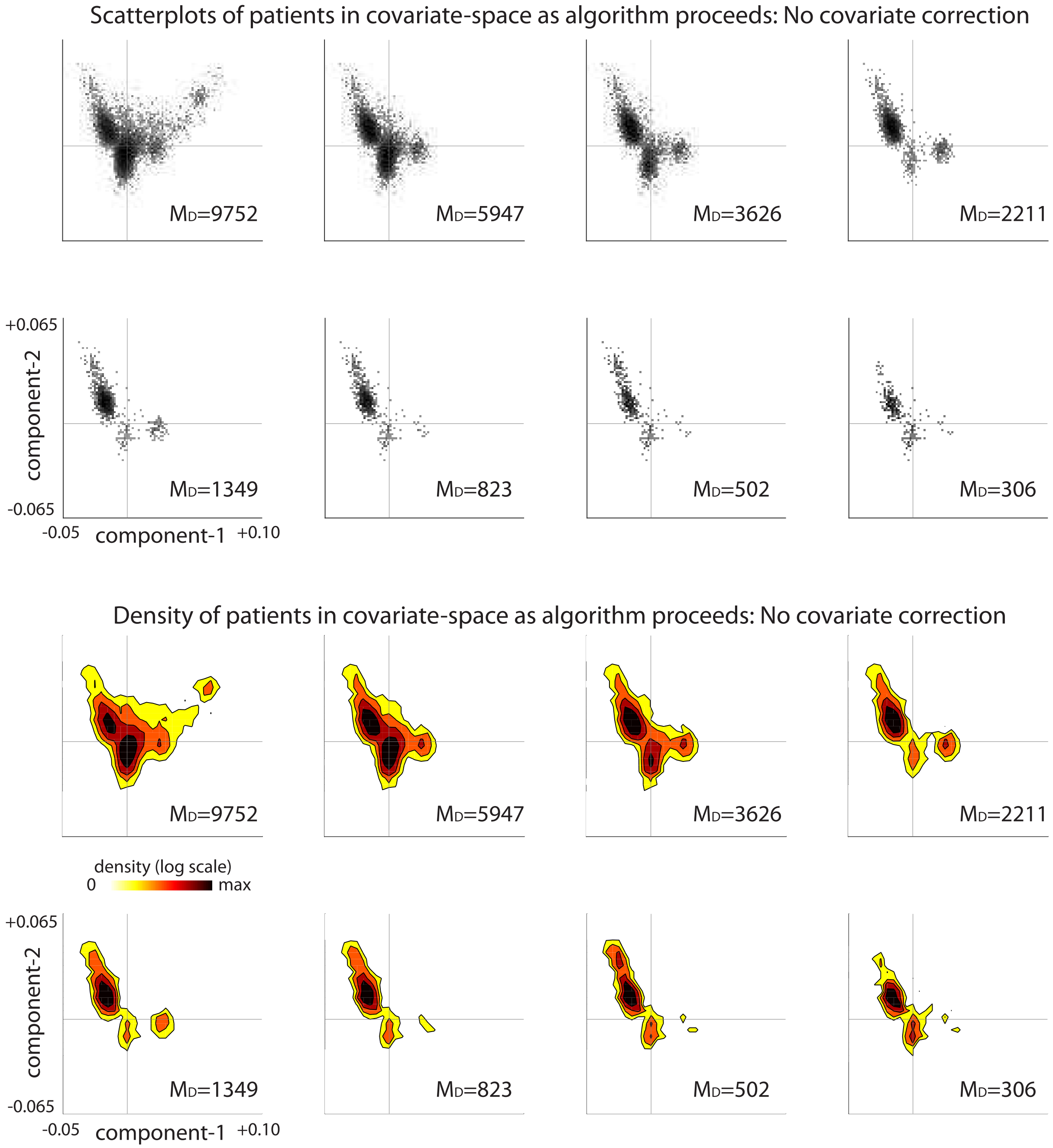

Figure 15: This figure has the same format as Fig 14, except that it shows the joint-distribution of continuous-covariates that would have resulted had we run our algorithm without correcting for continuous-covariates. Note that, in contrast to Fig 14, the covariate-distribution quickly becomes lopsided, involving mostly case-patients that are concentrated in a single quadrant of covariate-space.

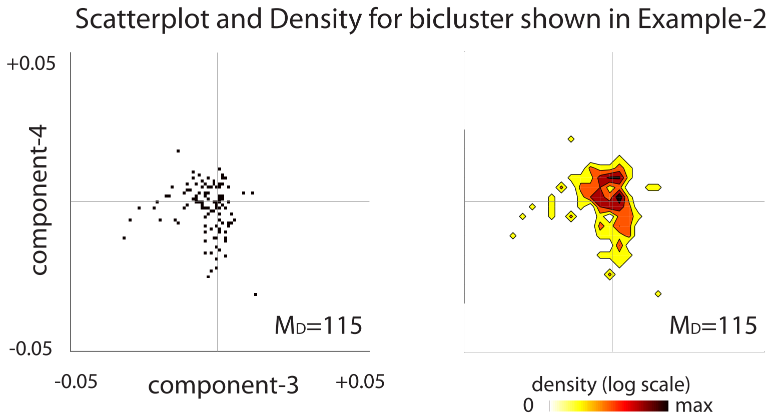

Figure 16: Joint-distribution of mds-components 3 and 4 for the bicluster shown in Example-3. This figure has the same format as Fig 13, with the exception that mds-components 3 and 4 are shown. Note that, even though we did not explicitly control for mds-components 3+4, they are relatively well distributed across the bicluster (see also Fig 17). A qualitatively similar story holds if we plot the joint-distribution of mds-components 1+3, 1+4, 2+3 or 2+4, and a similar trend holds for the later mds-components as well, which are even less structured than components 3 and 4.

The main observation that drives the success of our algorithm is that loops that are entirely contained within the embedded bicluster (such as the red loop) are guaranteed to be rank-1, whereas the other loops (such as the blue loops) are just as likely to be rank-2 as they are to be rank-1. Examples of rank-1 and rank-2 loops are shown in Fig 20B, with the row- and col-indices denoted by j, j' and k, k', respectively (there are $2^4 = 16$ possibilities).

Given this observation, we can ascribe to each row-index j the following row-score $[Z_{\text{ROW}}]_j$. We consider all the loops traversing the given row-j and accumulate a sum; adding 1 for every rank-1 loop, and subtracting 1 for every rank-2 loop. If we consider a situation where j is a row that does not participate in the bicluster B, then $[Z_{\text{ROW}}]_j$ will sum over $(M_D - 1)\,N\,(N-1)$ loops, roughly half of which will be rank-1 and half of which will be rank-2. This means that, when j does not participate in the bicluster, then the sum $[Z_{\text{ROW}}]_j$ will be roughly 0, with a standard-deviation (across the various j) close to $\sqrt{2}\sqrt{M_D N^2}$ (The factor of $\sqrt{2}$ arises because the loops are not all independent). On the other hand, when $\tilde{j}$ is a row that does participate in the bicluster, then $[Z_{\text{ROW}}]_{\tilde{j}}$ will sum over $(m_B - 1)\,n\,(n-1)$ loops that are fully contained within the bicluster B, and $(M_D - 1)\,N\,(N-1) - (m_B - 1)\,n\,(n-1)$ loops that straddle D as well as B. The loops within B will all be rank-1, and will collectively contribute a 'signal' of size $(m_B - 1)\,n\,(n-1) \sim m_B n^2$ to $[Z_{\text{ROW}}]_{\tilde{j}}$. The loops that straddle D will be roughly half rank-1 and half rank-2, contributing an average of 0 to $[Z_{\text{ROW}}]_{\tilde{j}}$, with a 'noise' – or standard-deviation – of roughly $\sqrt{2}\sqrt{M_D N^2 - m_B n^2}$ (taken across the various $\tilde{j}$).

Based on these considerations, we expect that the collection of row-scores associated with rows outside of B will be distributed like a gaussian with a mean of 0 and a standard-deviation $\sim \sqrt{2}\sqrt{M_D N^2}$ (blue curve in Fig 20C). On the other hand, the collection of row-scores associated with rows inside B will be distributed like a gaussian with a mean of $\sim m_B n^2$ and a standard-deviation of at most $\sim \sqrt{2}\sqrt{M_D N^2}$ (red curve in Fig 20C). If $m_B n^2$ is comparable to or larger than $\sqrt{M_D N^2}$, then we expect the 'signal' to be somewhat distinguishable from the 'noise'; the rows scores associated with the bicluster B should be significantly different from those associated with the rest of D.

Unfortunately, we usually don't know which rows are which, and so we don't see the blue- and red-curves shown in Fig 20C. Rather, after calculating all the row-scores we see something like Fig 20D. At first it might seem reasonable to guess that the rows corresponding to the highest scores are rows of B. While this statement is true when B is sufficiently large, it tends not to hold when B is much smaller than D. A better bet is to guess that the *lowest* row-scores are *not* from B. Quantitatively: assuming that $m_B n^2 \gtrsim \sqrt{M_D N^2}$, then the row with the lowest score is exponentially unlikely to be part of B.

Our strategy is built around this last observation: at every step we eliminate a few rows of D corresponding to the lowest row-scores. These eliminated rows are exponentially unlikely to come from B. We also do the same thing with the columns, eliminating the columns of D corresponding to the lowest col-scores (in this case the 'signal' associated with the columns of B is $\sim m_B^2 n$). After each such elimination, the row- and col-scores associated with the remaining rows and columns change, and so we recalculate the scores and repeat. Intuitively, we expect that, as we eliminate rows and columns, we are most likely to eliminate rows and columns of D, while leaving B untouched. Thus, we expect the 'noise' in our score-distribution to decrease (e.g., the noise associated with the row-scores is $\sim \sqrt{M_D N^2}$), whereas the 'signal' should remain relatively constant (e.g., the signal associated with the row-scores is $\sim m_B n^2$). This should result in the loop-scores of B becoming more and more distinguishable from the other loop-scores; the two distributions shown in Fig 20D should become narrower and narrower, while preserving their means. Put another way, as the algorithm progresses we expect the observed distribution of scores (shown in Fig 20D) to gradually evolve into a bimodal distribution with two

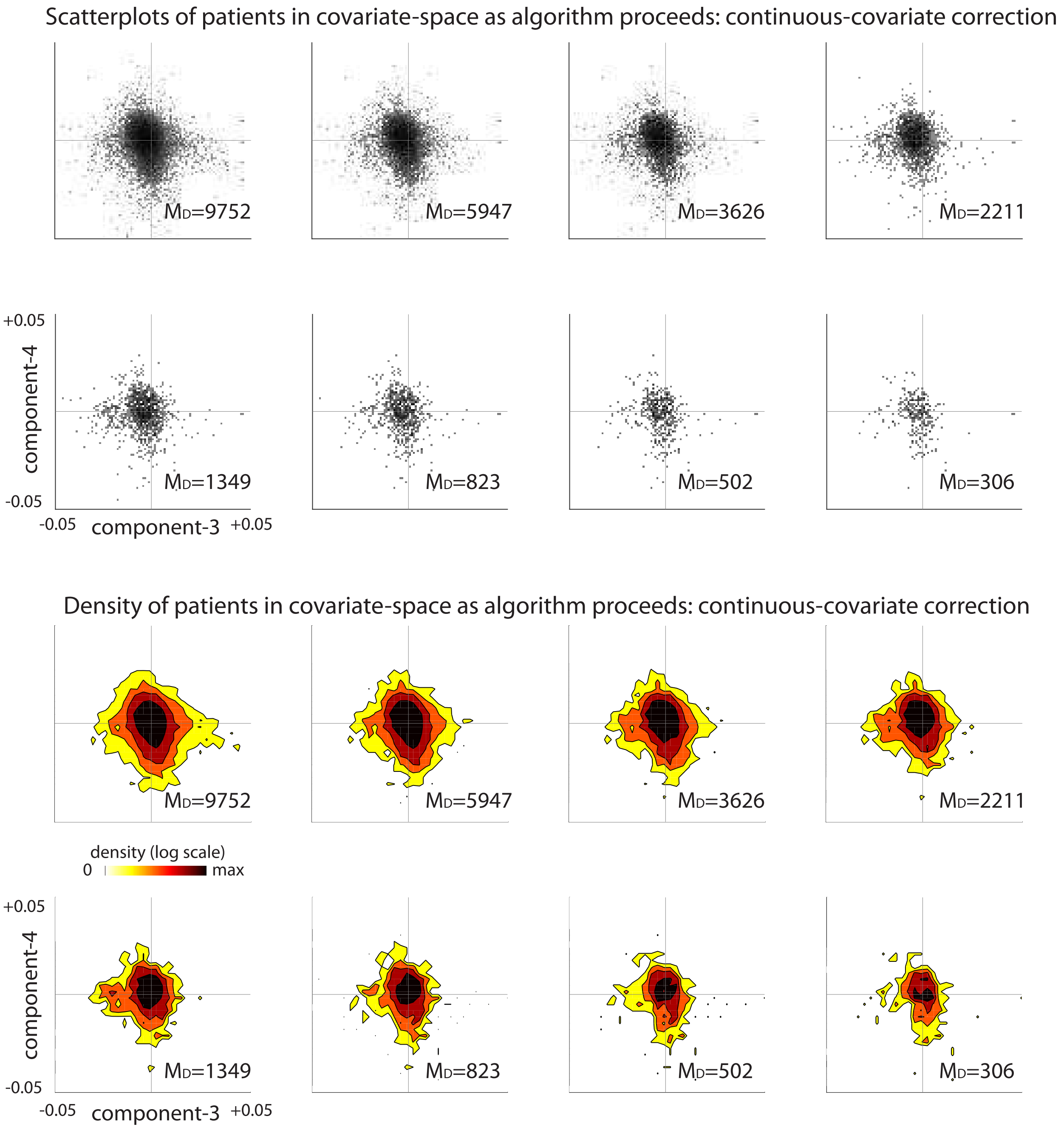

Figure 17: Joint-distribution of mds-components 3 and 4 as the algorithm proceeds. This figure has the same format as Fig 14, with the exception that mds-components 3 and 4 are shown. Note that these two mds-components are much less structured than the first two (shown in Fig 14). Even though we did not explicitly control for mds-components 3+4 when running our algorithm, these mds-components remain relatively well distributed as the algorithm proceeds. Just as in Fig 16, we see qualitatively similar results if we plot the joint-distribution of mds-components 1+3, 1+4, 2+3, 2+4, or any pair involving higher-order mds-components.

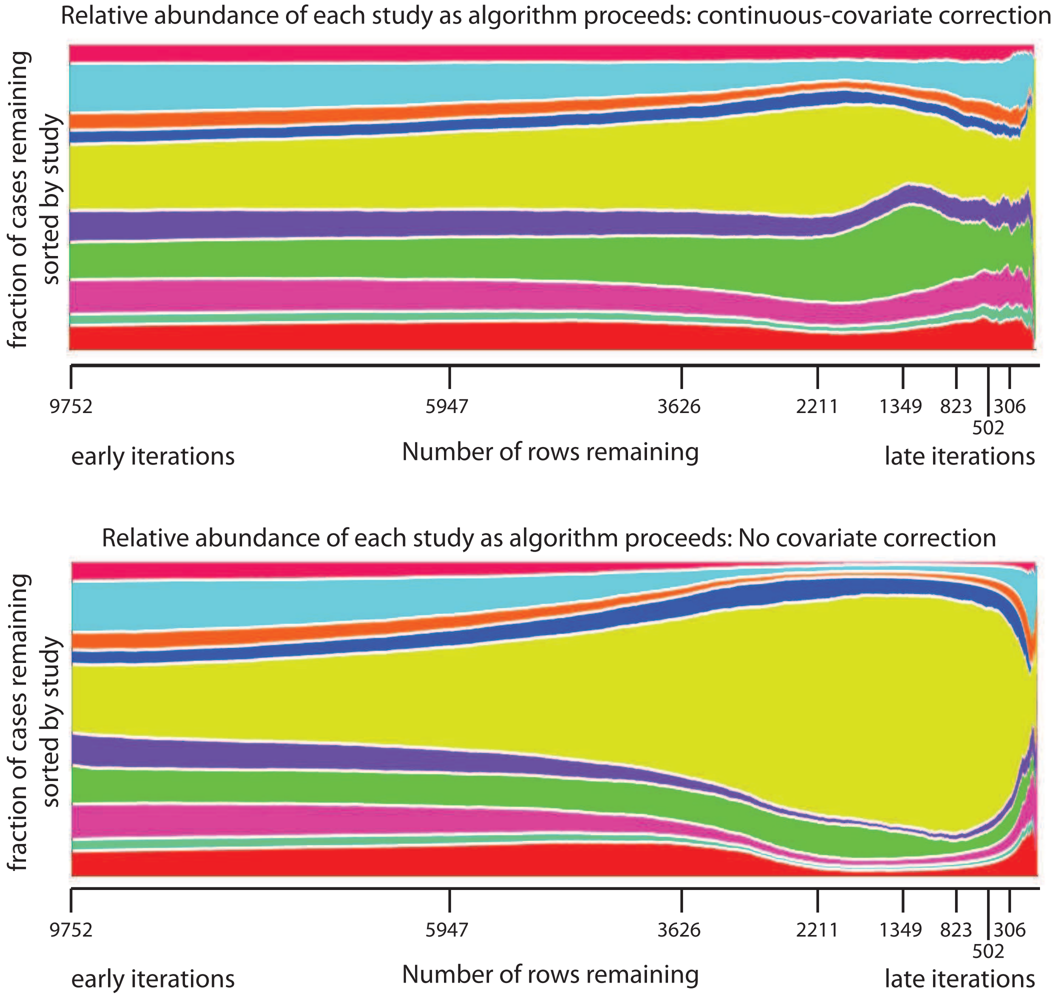

Figure 18: Study-distribution for Example-3. As mentioned previously, our algorithm proceeds iteratively, removing patients (i.e., rows) and alleles (i.e., columns) until none remain. The output of our algorithm includes a list of the case-patients in the order they were eliminated (with the patients that most strongly participate in the bicluster listed at the end). In this particular example each patient belongs to one of 10 studies, each collected by a different research group. It is important to ensure that any biclusters we find involve case-patients drawn from a reasonable mixture of studies. This is true for the Example shown here, even though we only explicitly corrected for continuous-covariates (and did not explicitly correct for study as a categorical-covariate). On top we display a stacked bar-graph illustrating the distribution of studies across the remaining case-patients as our algorithm proceeds. The vertical axis indicates study-fraction (different studies are colored differently), and the horizontal axis indicates the number of case-patients remaining. The iterations corresponding to the subplots in Fig 14 are indicated below the horizontal axis. Note that, for the most part, the remaining case-patients are relatively well-balanced across the 10 studies. The exact study-distribution for the 115 patients within the bicluster shown in Fig 11 is shown in Fig 19. On the bottom we show a similar stacked bar-graph for our algorithm run *without* covariate-correction of any kind. Note that – without covariate-correction – the latter half of the iterations are dominated by a few of the studies.

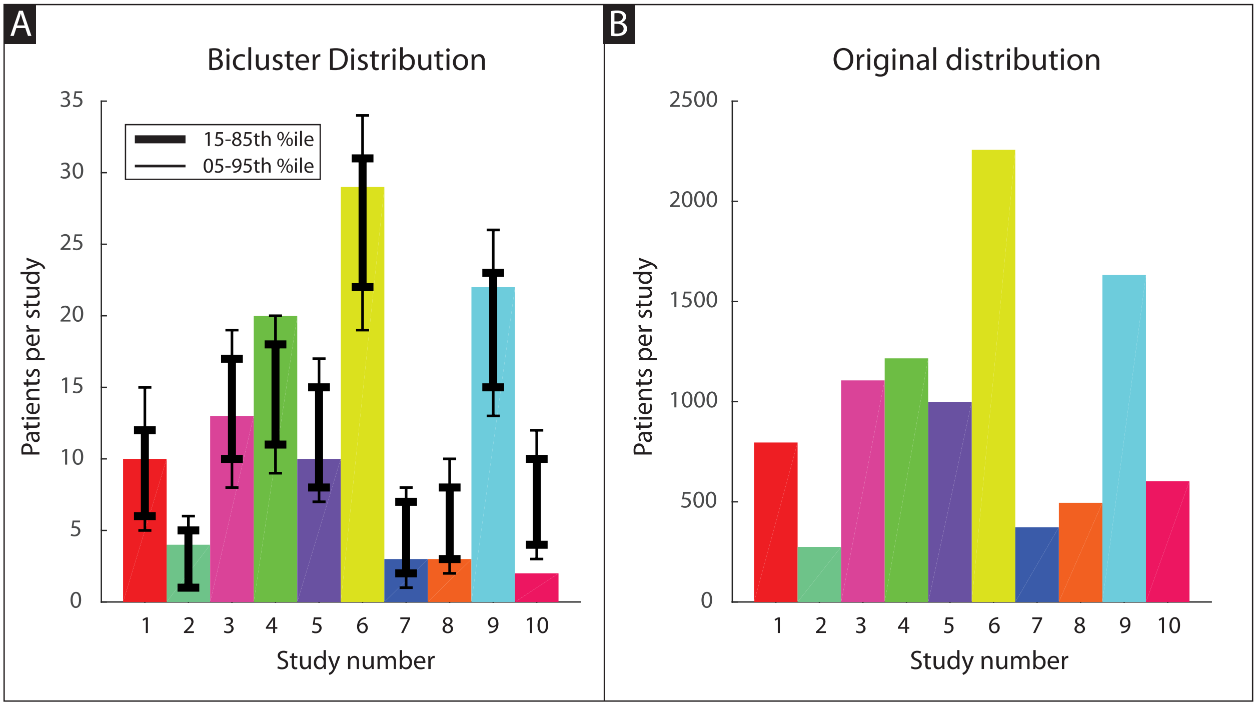

Figure 19: Study-distribution for the bicluster shown in Example-3. The particular bicluster shown in Example-3 was selected based on the peak of the z-score of the row-trace (see end of section 14.2). This bicluster involves 115 patients, distributed across the 10 studies mentioned in Fig 18. Even though we did not explicitly correct for study as a categorical-covariate, we did correct for continuous-covariates. As described above, since study is rather well correlated with the continuous-covariate, we expect that the bicluster we found should have a reasonable distribution across studies. This is indeed the case. In panel-A we show the study-distribution of the 115 patients within the bicluster. The color and ordering of each bar corresponds to the color and ordering of the 10 studies used in Fig 18. In panel-B we show the original study-distribution of the 9752 case-patients. Note that the study-distribution of the bicluster is not too different from the original study-distribution of the case-patients. To quantify this observation we drew multiple samples of 115 random patients from the original set, and measured the study-distribution of each random sample. Overlaid on panel-A we show the 5th, 15th, 85th and 95th percentiles (per study) for these randomly-sampled study-distributions. As can be seen, the study-distribution of the bicluster falls well within the bounds one might expect for a perfectly-balanced bicluster, with the possible exceptions of study 10 (which is underrepresented) and study 4 (which is slightly overrepresented).

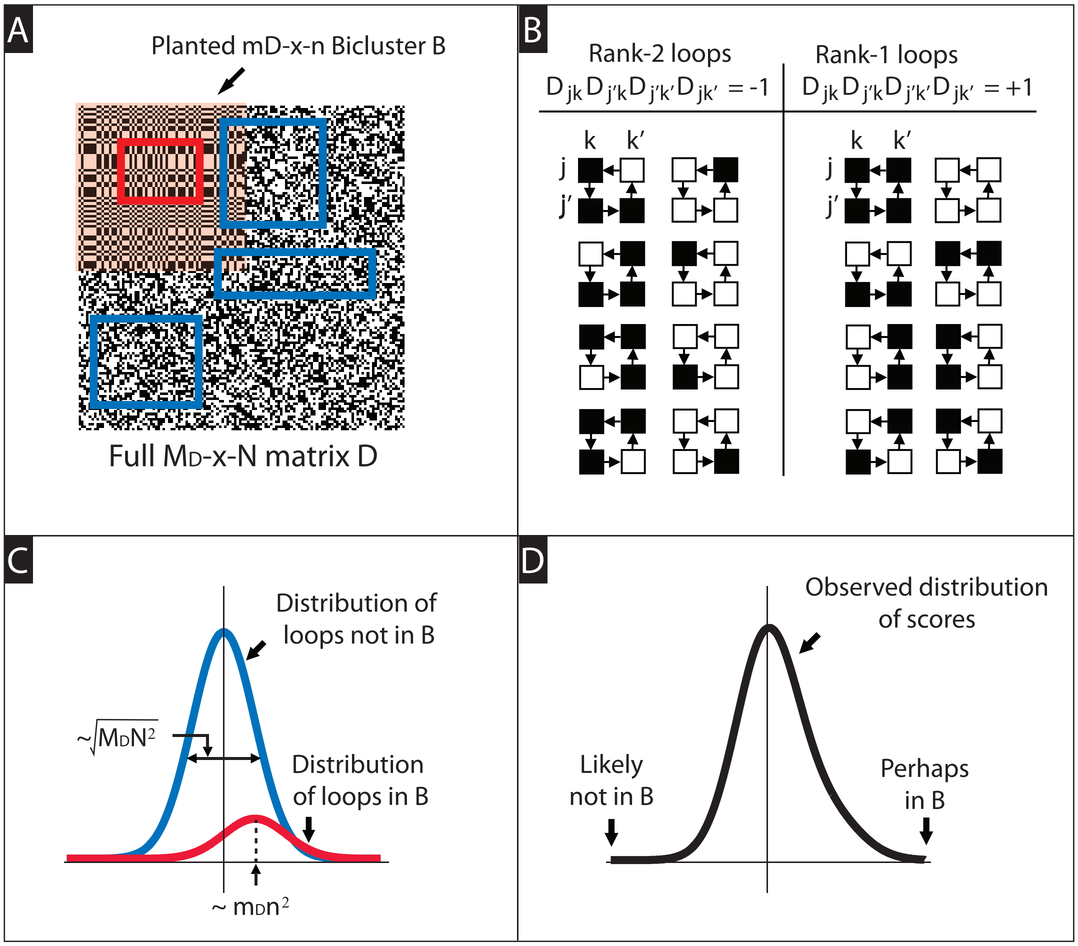

Figure 20: Illustration of the algorithm operating on a case-matrix alone (i.e., D only). In Panel-A we show a large $M \times N$ binarized matrix D (black and white pixels correspond to values of ± 1, respectively). In the upper left corner of D we've inserted a large rank-1 bicluster B (shaded in pink). Our algorithm considers all 2×2 submatrices (i.e., 'loops') within D. Several such loops are highlighted via the blue rectangles (the corners of each rectangle pick out a 2×2 submatrix). Generally speaking, loops are equally likely to be rank-1 or rank-2. Some loops, such as the loop shown in red, are entirely contained within B. These loops are more likely to be rank-1 than rank-2. In Panel-B we show some examples of rank-2 and rank-1 loops. Given a loop with row-indices j, j' and column-indices k, k', the rank of the loop is determined by the sign of $D_{jk} D^{\intercal}_{kj'} D_{j'k'} D^{\intercal}_{k'j}$. Our algorithm accumulates a 'loop-score' for each row j and each column k. In its simplest form, the loop-score for a particular row j is given by $\sum_{j',k,k'} D_{jk} D^{\intercal}_{kj'} D_{j'k'} D^{\intercal}_{k'j} = [DD^{\intercal}DD^{\intercal}]_{jj}$. Analogously, the loop-score for a column k is given by $[D^{\intercal}DD^{\intercal}D]_{kk}$. In Panel-C we show the distribution of loop-scores we might expect from the rows or columns within D. The blue-curve corresponds to the distribution of scores expected from the rows/cols of D that are not in B, whereas the red-curve corresponds to the distribution of scores expected from the rows/cols of B. In Panel-D we show the distribution of loop-scores we might expect by pooling all rows or columns of D. The rows or columns that correspond to the lowest scores are not likely to be part of B.

distinct peaks. While we don't have a proof of this phenomenon, our numerical experiments support this intuition.

3 Calculating the scores:

In terms of computation, the row-scores mentioned above can be computed as follows:

$$\begin{aligned}
[Z_{\text{ROW}}]_j &= \sum_{\substack{j \text{ fixed}, j'\neq j;\\ k'\neq k}} D_{jk}D_{j'k}D_{j'k'}D_{jk'} = \sum_{\substack{j\text{fixed}, j'\neq j;\\ k'\neq k}} D_{jk}D^{\intercal}_{kj'}D_{j'k'}D^{\intercal}_{k'j} \\
&= \sum_{j \text{ fixed}, j',k',k} (1-\delta_{jj'}-\delta_{kk'}+\delta_{jj'}\delta_{kk'})\, D_{jk}D^{\intercal}_{kj'}D_{j'k'}D^{\intercal}_{k'j} \\
&= \sum_{j \text{ fixed}, j',k',k} D_{jk}D^{\intercal}_{kj'}D_{j'k'}D^{\intercal}_{k'j} - \sum_{j \text{ fixed}, k',k} D_{jk}D^{\intercal}_{kj}D_{jk'}D^{\intercal}_{k'j} \\
&\quad - \sum_{j \text{ fixed}, j'k} D_{jk}D^{\intercal}_{kj'}D_{j'k}D^{\intercal}_{kj} + \sum_{j \text{ fixed}, k} D_{jk}D^{\intercal}_{kj}D_{jk}D^{\intercal}_{kj} \\
&= [DD^{\intercal}DD^{\intercal}]_{j,j} - N^2 - M_D N + N \\
&= [DD^{\intercal}DD^{\intercal}]_{j,j} - N\,(M_D + N - 1)\,.
\end{aligned}$$

The column-scores can be computed similarly, reducing to

$$[Z_{\text{COL}}]_k = [D^{\intercal}DD^{\intercal}D]_{k,k} - M_D\,(N + M_D - 1)\,.$$

Note that these scores can be calculated using only a few matrix-matrix multiplications.

The final constant on the right-hand-side above is chosen so that the left-hand-side excludes the contribution of all the loops that only travel across a single row- or column-index. If we were to draw these loops in Fig 20, they would describe line-segments (or points) rather than full rectangles. These 'collapsed' loops do not have the distribution of ranks that we discussed above – indeed, they are all rank-1. There are $N\,(M_D + N - 1)$ of these collapsed-loops for each row, and $M_D\,(N + M_D - 1)$ for each column. If we were to include these collapsed-loops in our score, they would each add a '+1' to their respective totals; the mean score of any row- or column- would then include the contribution from all these collapsed-loops.

Within the context of our D-only algorithm described in section 2, we don't actually need to exclude these collapsed-loops. Specifically, the collapsed-loops contribute the exact same quantity $N\,(M_D + N - 1)$ to each row-score in the matrix, regardless of whether or not that row is part of a bicluster or not. A similar story holds for the columns; the collapsed-loops contribute $M_D\,(N + M_D - 1)$ to each column-score. Our algorithm only considers the ranking of row- and column-scores, the identity of the removed rows and columns will be the same whether or not we exclude the collapsed-loops.

Nevertheless, we still typically exclude the collapsed-loops – removing their contribution from the row- and column-scores. The reason we do this is because, later on, we'll consider more complicated situations involving controls and covariates. In these more complicated situations it is convenient to exclude collapsed-loops so that, in the absence of biclusters, the mean score of each row- and column will be 0.

While not necessary at this point, we'll later rescale these scores so that they range from negative one to positive one:

$$\begin{aligned}
[Z_{\text{ROW}}]_j &= \frac{1}{(M-1)\,N\,(N-1)} \sum_{\substack{j \text{ fixed}, j'\neq j;\\ k'\neq k}} D_{jk}D_{j'k}D_{j'k'}D_{jk'}, \text{ and} \\
[Z_{\text{COL}}]_k &= \frac{1}{(N-1)\,M\,(M-1)} \sum_{\substack{j'\neq j;\\ k \text{ fixed}, k'\neq k}} D_{jk}D_{jk'}D_{j'k'}D_{j'k}.
\end{aligned}$$

When considering large random matrices, these rescaled row- and column-scores will have means of 0 and variances of roughly $2/\left(MN^2\right)$ and $2/\left(M^2N\right)$, respectively. To represent these rescaled scores more easily, we'll later introduce the notation of a 'rescaled sum' $\widetilde{\Sigma}$ which implicitly includes a prefactor equal to the number of summands:

$$\begin{aligned}
[Z_{\text{ROW}}]_j &= \widetilde{\sum_{\substack{j \text{ fixed}, j'\neq j;\\ k'\neq k}}} D_{jk}D_{j'k}D_{j'k'}D_{jk'}, \text{ and} \\
[Z_{\text{COL}}]_k &= \widetilde{\sum_{\substack{j'\neq j;\\ k \text{ fixed}, k'\neq k}}} D_{jk}D_{jk'}D_{j'k'}D_{j'k}.
\end{aligned}$$

3.1 Updating the scores using a low-rank update:

When removing a row or column the row- and col-scores change. In the simplest implementation the new row- and col-scores can be recalculated from scratch. However, it is often more efficient to simply update the older scores via a low-rank update. To see why this might be true, let's assume that D has the block structure

$$D = \begin{bmatrix} E & c \\ r & d \end{bmatrix},$$

where E is size $M_E \times N_E$, c is $M_E \times N_c$, r is $M_r \times N_E$, and d is $M_r \times N_c$. We'll also assume that we have already calculated the row-scores of D, and plan on eliminating r, c and d from D, leaving E remaining. We would like to determine how the row-scores of E depend on those of D, as well as on the eliminated submatrices r, c and d. This relationship is derived mainly from the following formula (note that, for the convenience of presentation, we have assumed that the submatrix E spans the first M_E rows of D):

$$\left[DD^{\intercal}DD^{\intercal}\right]_{j,j} = \left[EE^{\intercal}EE^{\intercal}\right]_{j,j} + \left[EE^{\intercal}cc^{\intercal} + cc^{\intercal}EE^{\intercal} + cc^{\intercal}cc^{\intercal} + \left(Er^{\intercal} + cd^{\intercal}\right)\left(rE^{\intercal} + dc^{\intercal}\right)\right]_{j,j} \text{ for } j = 1, \ldots, M_E.$$

Consequently, the row-scores for E can be calculated using those from D along with an update involving only matrix-vector multiplications such as $c^{\intercal}E$, $\left(c^{\intercal}E\right)E^{\intercal}$, $Er^{\intercal}$, and so forth. While forming the row-scores for D in the first place may require $O\left(M_D N \min\left(M_D, N\right)\right)$ operation, updating those scores to form the row-scores of E requires only $O\left(M_E N_E\right)$ operations. These updates are performed in the matlab source-code included in the supplementary tutorial 'S1 Source Code', as well as in the c source-code included in the supplementary material 'S2 Source Code' (both of which also use similar observations to update the column-scores).

4 Interpreting the scores:

There are many different ways to interpret our row-scores and column-scores, and we mention some here.

Sum over loops: As discussed above, our scores $[Z_{\text{ROW}}]_j$ and $[Z_{\text{COL}}]_k$ tally the ranks of the loops associated with row-j and column-k of D, respectively.

Signal: These scores serve as an accumulation of 'signals' driven by the various structured elements within the data-matrix. As we'll discuss in section 5, a large random $M \times N$ data-matrix D will give rise to row- and column-scores that have a mean of 0. If the matrix D were to contain an $m \times n$ low-rank bicluster B involving row-subset J_B and column-subset K_B, then the presence of B will impact the scores of J_B and K_B. Depending on how noisy it is, B will add a 'signal' of $\sim mn^2$ to the row-scores of J_B, and $\sim m^2n$ to the column-scores of K_B. If D were to contain multiple biclusters, then each would add their signals to the scores of their row- and column-subsets. Conversely, the score of any row or column in D will reflect the accumulation of signals it receives from all the biclusters it participates in. By eliminating the rows and columns with the smallest scores, we are eliminating those rows and columns that have the smallest signal. This removes rows and columns which either belong to no biclusters or belong to the fewest/weakest/smallest biclusters, allowing our algorithm to focus on the remaining rows and columns that have a strong signal. The ramifications of this iteration are discussed more in section 7.3.

Correlation: Our scores can also be interpreted as a measure of correlation. If we don't correct for collapsed-loops, the row- and column-scores are given by the diagonal entries of $DD^{\intercal}DD^{\intercal}$ and $D^{\intercal}DD^{\intercal}D$, respectively. The row-score $[Z_{\text{ROW}}]_j$ will then be proportional to the mean-squared correlation between row-j and all the rows of the case-matrix D. Similarly, the column-score $[Z_{\text{COL}}]_k$ is proportional to the mean-squared correlation between column-k and all the columns of D. By eliminating the rows and columns with the lowest scores we are eliminating the rows and columns that are least correlated with the rest, allowing the algorithm to focus on those remaining. As we describe in sections 5,6 and 7, this process facilitates the discovery of any tightly correlated subsets of rows and columns.

Trace: If we don't correct for collapsed-loops, the average row-score across all the rows in D will equal the average column-score across all the columns in D. Both averages will be proportional to the trace of $DD^{\intercal}DD^{\intercal}$, which is equal to $\sum \sigma_j^4$, where $\left\{\sigma_1, \ldots, \sigma_{\min(M,N)}\right\}$ are the singular-values of D. This is equal to the nuclear norm of $DD^{\intercal}DD^{\intercal}$, which will be high if D is low-rank (see, e.g., [$\mathcal{M}$35] for a discussion of the nuclear norm, and its applications to clustering and biclustering).

Rayleigh-Quotient: Again, if we don't correct for collapsed-loops, the row-score $[Z_{\text{ROW}}]_j$ will be proportional to the rayleigh-quotient $\vec{x}^{\intercal}D^{\intercal}D\vec{x}$, where $\vec{x}^{\intercal} = D_{j,:}$ is the jth-row of D. The column-score $[Z_{\text{COL}}]_k$ will be proportional to $\vec{y}^{\intercal}DD^{\intercal}\vec{y}$, where $\vec{y} = D_{:,k}$ is the kth-column of D. Our algorithm attempts to focus on those rows and columns which

correspond to large rayleigh-quotients; i.e., to those rows and columns which are most parallel to the dominant eigenvectors of $D^\intercal D$ and $DD^\intercal$, respectively. This perspective is discussed in more detail in [$\mathcal{M}$22] and [$\mathcal{M}$40] and in section 22.

5 Generalization to noisy biclusters

The discussion above was prefaced by the rather idealistic assumption that the embedded bicluster B was perfectly rank-1; i.e., that all the rows and columns of B were perfectly correlated with one another. In practice, of course, the situation is far messier. Any hidden low-rank bicluster B will be noisy; B won't be exactly rank-1, and its rows and columns will be imperfectly correlated.

It turns out that our methodology above still works in these messier situations. The main reason is that, even when B is noisy (and even when B isn't exactly rank-1 itself), the loops within a binarized version of B are still more likely to be rank-1 than to be rank-2. While this probability 'g' won't be 100% (as in the ideal case above), it will still be significantly greater than 50%, provided that the numerical rank of B is sufficiently small and the singular-values of B decay sufficiently quickly. Under these conditions (which we'll quantify momentarily), B will still have a 'signal' distinguishing it from the rest of D. For example, the row-scores associated with B will be drawn from a distribution with a mean that, while lower than the ideal $m_B n^2$ above, will be $m_B n^2 (2g - 1)$, which is still on the same order as $m_B n^2$. Similarly, the col-scores of B will be drawn from a distribution with a mean of $m_B^2 n (2g - 1)$, which is on the same order as $m_B^2 n$.

Now we turn to a discussion of just how noisy B can be before our algorithm fails to detect it. This discussion is essentially the same as that in [$\mathcal{M}$39]. To start with we imagine that $m_B < n$, and that each column of $B \in \mathbb{R}^{m_B \times n}$ is drawn from a distribution ρ in $\mathbb{R}^{m_B}$ which is constructed as follows. We fix a small number $l \lesssim 4$ and take ρ to be a randomly oriented multivariate gaussian distribution which has l large principal values equal to 1, and the remaining principal values equal to $\varepsilon < 1$ (see Fig 21A). This gaussian-distribution ρ can be thought of as a distribution where a significant fraction of its variance is accounted for by its first l principal components. Specifically, this fraction is $l/\left(l + \varepsilon^2 (m_B - l)\right) \sim l/\left(l + \varepsilon^2 m_B\right)$, which will be large as long as $\varepsilon^2 m_B$ is small. This construction of B then implies that B will be of numerical rank l, with a 'fuzziness' of ε. The contours of ρ look like ellipsoids, with eccentricity determined by $1/\varepsilon$. For example, if ε were to be 0, then ρ would be compressed to an l-dimensional hyperplane, and B would be exactly rank-l (i.e., very eccentric). If, on the other hand, ε were to be 1, then ρ would be a uniform gaussian and B would not be low-rank at all (i.e., not eccentric). For most typical applications in gene-expression analysis we can think of a fuzziness ε on the order of $\sim 1/30$ to $1/3$, and m on the order of 10 to 100. For applications in GWAS m may be a little larger, ranging up to 1000 or so.

What we'll endeavor to show below is that, when l and ε are sufficiently small, then the distribution of loops drawn from B will be different than the distribution of loops drawn from the rest of D. Specifically, we'll quantify the probability g_{l,ε,m_B} that a randomly chosen loop drawn from B will – after binarization – be rank-1 rather than rank-2. As we'll see, a soft 'threshold' will be crossed when $\varepsilon \gtrsim 1/\sqrt{m_B}$, at which point the bicluster B is essentially uncorrelated, g_{l,ε,m_B} will be essentially 1/2, loops of B will look like loops of D, and our algorithm will fail. Looking back at our definition of ρ, this threshold is natural, for when $\varepsilon \gtrsim 1/\sqrt{m_B}$ the distribition ρ is no longer well captured by its first l principal components. For most typical applications, we can expect $\varepsilon\sqrt{m}$ to be on the order of ~ 0.1 to 1. These typical biclusters will be mostly correlated (i.e., they will be well approximated by their first principal component), but this correlation will not necessarily be exhibited in each and every entry (e.g., see Fig 22).

Now let us consider an arbitrary 2-by-2 submatrix (i.e., loop) within such an ε-fuzzy rank-l matrix B. This loop spans row-indices j, j' and col-indices k, k'. This loop can be described by first drawing two m_B-dimensional vectors from ρ (i.e., two columns k and k' from B), and then choosing two coefficients to read from (i.e., two row-indices j and j'). Based on our assumptions, such a loop is drawn from a distribution constructed in two steps: the first step involves sampling two vectors from ρ, whereas the second step involves projecting those two vectors onto the plane spanned by 2 randomly-chosen coordinate-axes.

Now recall that we defined B in such a way that the columns of B were drawn from a randomly-oriented gaussian-distribution ρ. The distribution obtained by (i) starting with a randomly-oriented gaussian and then projecting onto a fixed plane is the same as the distribution obtained by (ii) starting with a fixed gaussian, and then projecting onto a randomly oriented plane. Thus, without loss of generality, we can assume that loops of B are drawn in the following manner (where we have used $m = m_B$ for brevity):

1. First define an eccentric gaussian-distribution ρ on $\mathbb{R}^m$ which has l principal-values equal to 1 and the remaining principal values equal to $\varepsilon < 1$. Then orient ρ so that the first l principal components align with the first l coordinate axes (and the remaining $(m - l)$ principal-components align with the other coordinate axes). This ρ is a fixed distribution that depends only on l, ε and m.

2. Now select a uniformly-distributed randomly-oriented planar orthogonal-projection $P^{2\leftarrow m} : \mathbb{R}^m \to \mathbb{R}^2$, and define the projected distribution $\tilde{\rho} = P^{2\leftarrow m}\rho$ as a distribution on $\mathbb{R}^2$. Note that, as ρ was a multivariate gaussian, so too

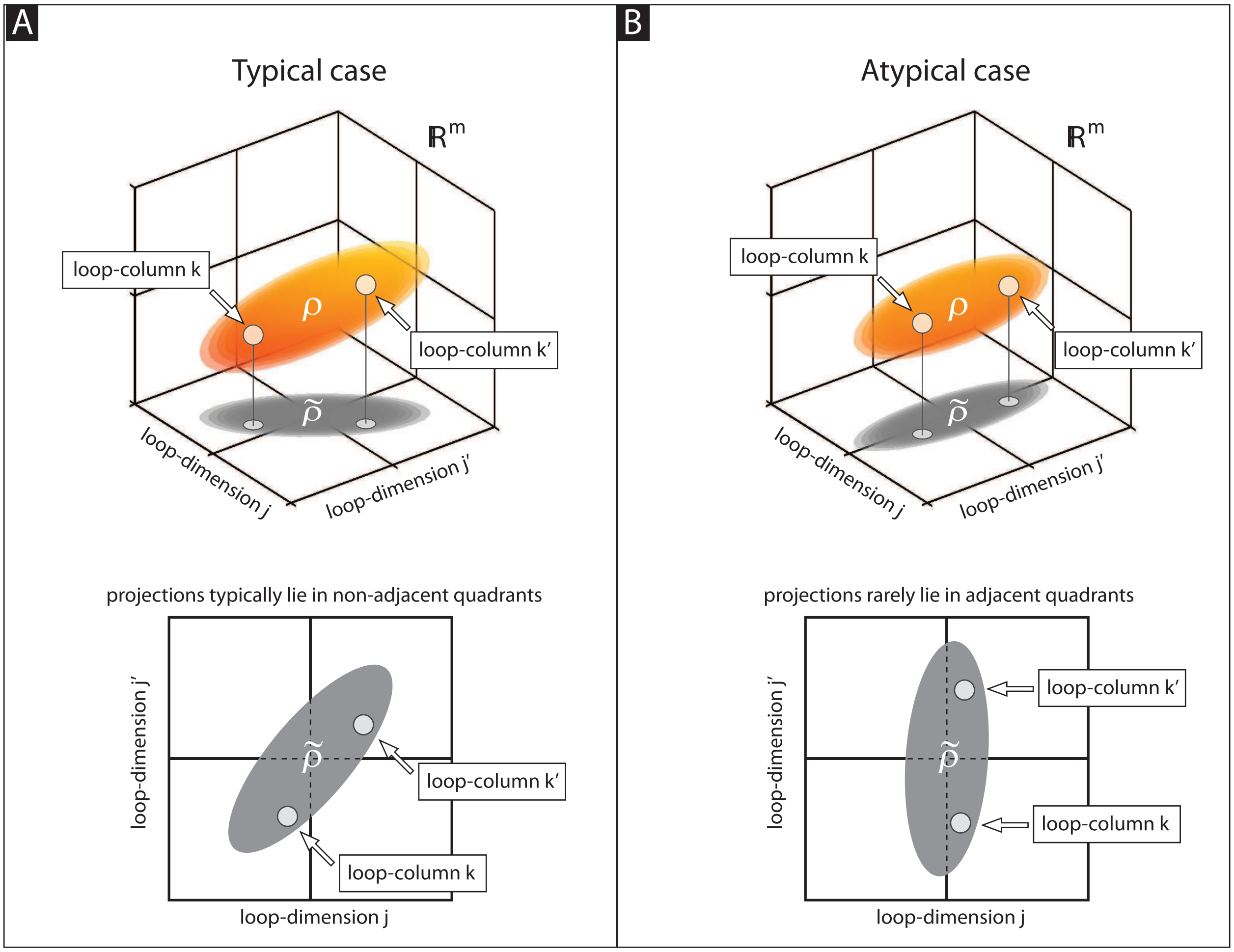

Figure 21: This figure illustrates the main geometric feature underlying the success of our algorithm: namely, a 2-dimensonal projection of a randomly-oriented eccentric gaussian-distribution is typically concentrated in non-adjacent quadrants (see discussion in section 5). To set the stage, we sketch in Panel-A a randomly-oriented eccentric gaussian distribution ρ in $\mathbb{R}^m$ (orange). Any loop drawn from such a distribution involves first projecting ρ onto a 2 dimensional distribution $\tilde{\rho}$, and then sampling two points at random from $\tilde{\rho}$. The row-indices j and j' of the loop determine the range of the projection, while the column indices k and k' label the random samples (indicated here with two circles). After binarization, the rank of such a loop is determined by where its two samples lie in the plane. On one hand, if the two samples lie in the same or opposite quadrants, then the loop will be rank-1; this is the typical case, illustrated in panel-A. On the other hand, if the two samples lie in adjacent quadrants, then the loop will be rank-2; this can occur almost half the time if the major axis of $\tilde{\rho}$ happens to be closely aligned with a coordinate-axes – one such example is illustrated in panel-B. As we describe in section 5, case-A is more likely than case-B, and this probability increases significantly as ρ becomes more eccentric. Later we'll quantify this probability with a value g_{l,ε,m_B}, which measures the probability that a loop drawn from ρ will be rank-1 (rather than rank-2).

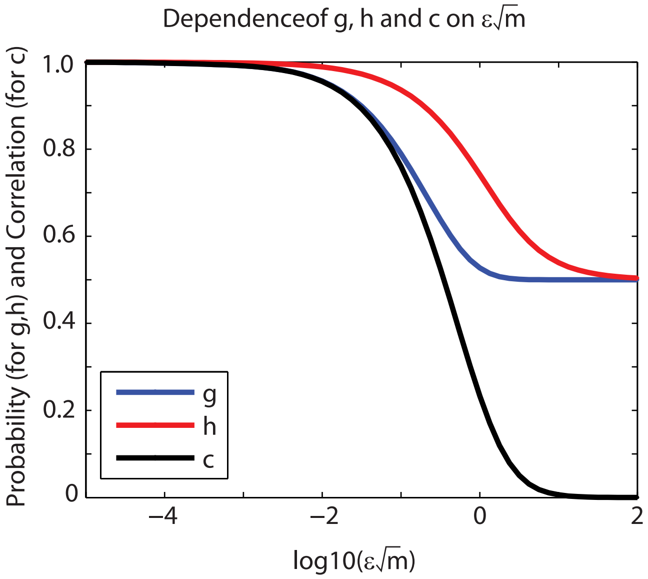

Figure 22: Dependence of $g_{1,\varepsilon\sqrt{m}} := g_{1,\varepsilon,m}$ (blue), $h_{1,\varepsilon\sqrt{m}}$ (red) and $c_{1,\varepsilon\sqrt{m}}$ (black) on $\varepsilon\sqrt{m}$. Given a randomly oriented rank-1 bicluster with spectral noise ε and size $m \times m$, the quantity $g_{1,\varepsilon\sqrt{m}}$ is the probability that a randomly chosen 'loop' will be rank-1 rather than rank-2 after binarization. The quantity $h_{1,\varepsilon\sqrt{m}}$ is the probability that a randomly chosen entry will have a sign opposite to that predicted by the rank-1 approximation to the bicluster. The quantity $c_{1,\varepsilon\sqrt{m}}$ is the expected absolute-value of the cross-correlation between different rows (or columns) within the bicluster.

will be $\tilde{\rho}$, and the contours of $\tilde{\rho}$ will look like ellipses on $\mathbb{R}^2$. However, unlike ρ, the distribution $\tilde{\rho}$ is not fixed, and will itself be drawn from a distribution (denoted by the measure $d\tilde{\rho}$, below).

3. Once $P^{2\leftarrow m}$ has been selected and $\tilde{\rho}$ has been determined, a loop of B will correspond to two 2-dimensional vectors, say v_1, $v_2 \in \mathbb{R}^2$, each drawn independently from $\tilde{\rho}$.

Binarization of B will send each of v_1, v_2 to one of the four corners of the unit-square in $\mathbb{R}^2$, determined by which quadrant of $\mathbb{R}^2$ the vector lies in. Thus, after binarization, the loop of B will be rank-1 if v_1 and v_2 happened to lie in the same quadrant (i.e., if, after binarization, v_1 and v_2 fall onto the same corner of the unit-square). Similarly, v_1 and v_2 will correspond to a rank-1 loop if they happened to lie in opposite quadrants (thus falling onto opposite corners of the unit-square). Only when v_1 and v_2 fall into adjacent quadrants (i.e., adjacent corners of the unit-square) will they correspond to a rank-2 loop. Finally, we note that since $\tilde{\rho}$ is a multivariate gaussian in $\mathbb{R}^2$, the fraction of $\tilde{\rho}$ contained within any quadrant must be the same as the fraction of $\tilde{\rho}$ contained within the opposite quadrant (e.g., the fraction of $\tilde{\rho}$ within the first-quadrant equals the fraction of $\tilde{\rho}$ within the third-quadrant). Summarizing all of these observations, we can state that, if $p\{\tilde{\rho}\}$ is the fraction of $\tilde{\rho}$ which is restricted to the first- and third-quadrants, then the fraction of $\tilde{\rho}$ restricted to the second- and fourth-quadrants is $1-p$. Consequently, the probability that the binarized loop of B will be rank-1 is simply $p^2 + (1-p)^2$, or $1 - 2p + 2p^2$. Thus, we can see that

$$g_{l,\varepsilon,m} = \int \left[1 - 2p\{\tilde{\rho}\} + 2p^2\{\tilde{\rho}\}\right] d\tilde{\rho},$$

where $d\tilde{\rho}$ is the measure on the distributions $\tilde{\rho}$ that one would obtain by following the prescription above.

At this point we'll embark on a description of $d\tilde{\rho}$, the measure from which $\tilde{\rho}$ is drawn. To simplify our notation, we will use $\rho_\sigma(x)$ to denote the normal distribution $N(0, \sigma^2)$ on x. In addition, we'll use $\rho_{a,b,\theta}(\vec{x})$ to denote the mean 0 anisotropic multivariate gaussian distribution in 2-dimensions with variances a^2 and b^2, and first principal-component oriented at angle θ (see section 16 for details).

The randomly chosen projection $P^{2\leftarrow m}$ can be determined by drawing two randomly chosen orthonormal vectors u^x, $u^y \in \mathbb{R}^m$, and then constructing $P^{2\leftarrow m} = [u^x, u^y]^\intercal$. Drawing u^x and u^y in this way would be slightly nontrivial, since we would need to constrain u^x to be $\perp$ to u^y. However, for sufficiently large $m \gg l$, we can approximate $P^{2\leftarrow m}$ just by drawing two random vectors $[w^x, w^y]^\intercal$ instead, where each entry of w^x and w^y is drawn independently from $\rho_{\sqrt{1/m}}$. These new vectors w^x and w^y won't be exactly length 1, nor exactly $\perp$ to one another. Nevertheless, they will typically be close to orthonormal (i.e., orthonormal to $O(1/\sqrt{m})$), which will be sufficient for our purposes. This kind of replacement – i.e., using w^x, w^y instead of u^x, u^y – will incur a small error, but will allow us to easily construct an approximation to $\tilde{\rho}$ which will be valid when $m \gg l$.

Using our notation above, we can see that $\tilde{\rho}$ is well approximated by:

$$\tilde{\rho} \sim [w^x, w^y]^\intercal \rho = \sum_{j=1}^{l} \begin{bmatrix} w_j^x \\ w_j^y \end{bmatrix} x_j + \sum_{j=l+1}^{m} \begin{bmatrix} w_j^x \\ w_j^y \end{bmatrix} x_j,$$

$$\text{or equivalently, } \tilde{\rho} \sim \sum_{j=1}^{l} \hat{w}_j x_j + \sum_{j=l+1}^{m} \hat{w}_j x_j.$$

In the expression above each of the $\hat{w}_j$ represents a 2-element vector $\left[w_j^x, w_j^y\right]^\intercal$, and each of the x_j are drawn from the fixed version of ρ described above; that is to say: $x_1, \ldots, x_l$ are each drawn independently from $\rho_1(x)$, and $x_{l+1}, \ldots, x_m$ are each drawn independently from $\rho_\varepsilon(x)$. Each of the terms $\hat{w}_j x_j$ is a distribution in $\mathbb{R}^2$, but one that is compressed onto the line running through $\hat{w}_j$. Because of how we constructed the w^x, w^y, each pair $\hat{w}_j$ is drawn from $\rho_{\sqrt{1/m},\sqrt{1/m},0}$. Since this latter distribution is isotropic, we can deduce that the orientation θ_j of the vector $\hat{w}_j$ is uniformly distributed in $[0, 2\pi]$. Similarly, we can deduce that the magnitude r_j is drawn from the Rayleigh distribution $mr\exp(-mr^2/2)$, which can also be written as $r\sqrt{2\pi m}\rho_{\sqrt{1/m}}(r)$ if we use our shorthand for a gaussian distribution (see section 16.4). This means that, when $j \leq l$, each of the terms $\hat{w}_j x_j$ is represented by some $\rho_{r_j,0,\theta_j}$. Consequently, the sum of any two $\hat{w}_j x_j + \hat{w}_{j'} x_{j'}$ will just be drawn from a distribution which is the convolution of the distributions from which the individual $\hat{w}_j x_j$ and $\hat{w}_{j'} x_{j'}$ are drawn. The latter sum $\sum_{j=l+1}^{m} \hat{w}_j x_j$, on the other hand, represents what is essentially a random 2-d projection of a uniform ε^2-variance gaussian distribution on $\mathbb{R}^m$ (since $m \gg l$). Such a projection will again be a uniform ε^2-variance gaussian distribution, looking like $\rho_{\varepsilon,\varepsilon,0}$.

Combining all these observations, $\tilde{\rho}$ can be rewritten as:

$$\tilde{\rho} \sim \{\rho_{r_1,0,\theta_1} \star \cdots \star \rho_{r_l,0,\theta_l}\} \star \rho_{\varepsilon,\varepsilon,0},$$

where each $r_1, \ldots, r_l$ is drawn independently from $r\sqrt{2\pi m}\rho_{\sqrt{1/m}}(r)$, and each $\theta_1, \ldots, \theta_l$ is drawn independently from the uniform distribution on $[0, 2\pi]$. The multiple convolution $\{\rho_{r_1,0,\theta_1} \star \cdots \star \rho_{r_l,0,\theta_l}\}$ is again a multivariate gaussian $\rho_{\alpha,\beta,\omega}$ for some α, β, ω. As a result, the distribution $\tilde{\rho}$ can be approximated by:

$$\tilde{\rho} \sim \rho_{\alpha,\beta,\omega} \star \rho_{\varepsilon,\varepsilon,0}$$

for some (yet undetermined) α, β, ω. This expression is simply a uniformly-ε-mollified version of the eccentric gaussian $\rho_{\alpha,\beta,\omega}$, which is merely a slightly less eccentric gaussian with the same orientation. That is to say,

$$\tilde{\rho} \sim \rho_{\sqrt{\alpha^2+\varepsilon^2},\sqrt{\beta^2+\varepsilon^2},\omega}.$$

Given this representation of $\tilde{\rho}$, a simple calculation shows that the fraction $p\{\tilde{\rho}\}$ of $\tilde{\rho}$ which is restricted to the first- and third-quadrants is:

$$p\{\tilde{\rho}\} = \frac{1}{\pi} \operatorname{arccot}\left(\left(\sqrt{\frac{\beta^2+\varepsilon^2}{\alpha^2+\varepsilon^2}} - \sqrt{\frac{\alpha^2+\varepsilon^2}{\beta^2+\varepsilon^2}}\right) \frac{1}{2}\sin 2\omega\right).$$

While precisely determining α, β, ω is tedious when $l > 1$, one can observe that the principal-values α, β of

$$\rho_{\alpha,\beta,\omega} = \{\rho_{r_1,0,\theta_1} \star \cdots \star \rho_{r_l,0,\theta_l}\}$$

depend on m in a simple way: both α and β scale with $1/\sqrt{m}$. As a result, $p\{\tilde{\rho}\}$ depends only on the combination $\varepsilon\sqrt{m}$, and not on ε or $\sqrt{m}$ independently. Because $g_{l,\varepsilon,m}$ is also a function of $\tilde{\rho}$ we expect that, for fixed l and sufficiently large m, the probability $g_{l,\varepsilon,m}$ will also be a function of $\varepsilon\sqrt{m}$.

At this point we can write down the probability $g_{l,\varepsilon,m}$ for the various l. In the case $l = 1$, $\rho_{\alpha,\beta,\omega}$ is trivially $\rho_{\alpha,\beta,\omega} = \rho_{r_1,0,\theta_1}$. This means that the probability $g_{1,\varepsilon,m}$ can be expressed as:

$$g_{1,\varepsilon,m} \sim \int_{r_1=0}^{r_1=\infty} \int_0^{2\pi} \frac{1}{2\pi} \left[1 - 2p\left(\theta_1, \frac{\varepsilon\sqrt{m}}{r_1}\right) + 2p\left(\theta_1, \frac{\varepsilon\sqrt{m}}{r_1}\right)^2\right] r_1 \exp\left(\frac{-r_1^2}{2}\right) d\theta_1 dr_1, \quad (1)$$

$$\text{with } p(\omega, \delta) = \frac{1}{\pi} \arctan\left(\frac{\delta\sqrt{1+\delta^2}}{-\frac{1}{2}\sin 2\omega}\right).$$

For the case $l > 1$ we can write out formulae for $g_{l,\varepsilon,m}$ via induction using Eq. 2 in section 16. This expression for $g_{l,\varepsilon,m}$ is very accurate even for moderate values of $m \gtrsim 64$. Numerical experiments confirming the accuracy of our approximation

for $g_{l,\varepsilon,m}$ are shown in Fig 23. As can be seen from these figures, when $l \lesssim 5$, the value of $g_{l,\varepsilon,m}$ is significantly greater than $1/2$ so long as $\varepsilon\sqrt{m} \lesssim 1$.

To summarize: When considering the bicluster B, the row-scores corresponding to B will take on values of $\sim mn^2 (2g_{l,\varepsilon,m} - 1)$ on average. If we hope to detect B, then these average scores should be on the same order as (or larger than) the standard-deviation of the remaining scores associated with rows and columns of D that are not in B. That is to say, we would expect to detect B when $mn^2 (2g_{l,\varepsilon,m} - 1) \sim \sqrt{2MN^2}$. In principle this implies that we should be able to detect B for arbitrarily low values of $g_{l,\varepsilon,m}$, so long as m, n are sufficiently large. However, in practice, this kind of limit is not typically achieved. For most real gene-expression data-sets we should expect $m := m_B$ to be no larger than ~ 32 to 100, (with M_D no larger than ~ 1000 to $10,000$), implying that our algorithm will succeed so long as $\varepsilon \lesssim 0.1$ or so.

5.1 Constructing a low-rank bicluster B:

In the above discussion (and further on below) we perform numerical experiments which involve constructing and binarizing $m \times n$ matrices B which are randomly-oriented, rank-l, and ε-error. When m, n are small we construct B using our original prescription above:

1. Given l, m, n, ε, construct a randomly-oriented multivariate gaussian distribution ρ in $\mathbb{R}^m$, with l large principal values equal to 1 and the remaining principal values equal to ε.
2. Construct A to be an $m \times n$ matrix by drawing each of the n columns of A independently from ρ.
3. Construct $B = \text{sign}(A)$.

When m, n are sufficiently large (e.g., $m, n > 128$), it becomes cumbersome for us to construct B this way, and instead we approximate B as follows:

1. Construct $\tilde{A}$ to be an $m \times n$ matrix with independent entries drawn from a standard normal distribution.
2. Form the singular-value-decomposition $\tilde{A} = USV^\intercal$.
3. Redefine the diagonal entries of S by setting $S(l,l) = 1$, and $S(j,j) = \varepsilon$ for $j > l$.
4. Redefine $\tilde{A}$ via $\tilde{A} := USV^\intercal$.
5. Construct $\tilde{B} = \text{sign}\left(\tilde{A}\right)$

The matrices $\tilde{A}$ formed using this svd-based method will not be drawn from the same distribution as the matrices A formed via our original prescription. Nevertheless, the matrices $\tilde{A}$ will still be rank-l with error-ε; when m is large the binarized matrices B will be very close (in distribution) to the binarized matrices $\tilde{B}$.

When m, n are even larger (e.g., $m, n > 1024$), even this svd-based prescription might be too slow. In these large-m, n scenarios, we take the following shortcut (in the cases where $l = 1$ and $m \leq n$):

1. Given $l = 1$, $m, n \gg 1$ and ε, determine $g := g_{1,\varepsilon,m}$ using Eq. 1 above.
2. Define the 'flip-probability' $f := f_{\varepsilon\sqrt{m}}$ as follows: $1-g = 4f^3(1-f)+4f(1-f)^3$, or $1-g = 4f(1-f)(1-2f(1-f))$. This can be done by first solving for $F = f(1-f)$ via $1 - g = 4F(1-2F)$, and then solving for f.
3. Construct $\hat{A}$ to be a rank-1 $m \times n$ binary-matrix $\hat{A} = uv^\intercal$, where u and v are both random binary vectors of length m and n respectively.
4. Choose fmn of the entries of $\hat{A}$ at random, and flip their sign.
5. Define $\hat{B} = \hat{A}$.

Note that the flip-probability f can be approximated by:

$$f \approx 1 - g \approx \frac{4}{\pi}\varepsilon\sqrt{m}\left[\frac{1}{\sqrt{2\pi}} + \frac{2}{\sqrt{2\pi}}\log\left(\frac{\pi}{2\varepsilon\sqrt{m}}\right) - \frac{2}{\pi}[0.7961]\right] \text{ when } \varepsilon\sqrt{m} \lesssim 0.01,$$

$$\text{and } f \approx \frac{1}{2} - \frac{1}{\sqrt{2\pi}\cdot\varepsilon\sqrt{m}} \text{ when } \varepsilon\sqrt{m} \gtrsim 10.$$

This sign-flipping method produces a random binary $m \times n$ matrix $\hat{B}$. Note that the matrices $\hat{B}$ will not be drawn from the same distribution as the matrices B or $\tilde{B}$. For example, the entries of the covariance matrix $\hat{B}\hat{B}^\intercal$ will be drawn from a

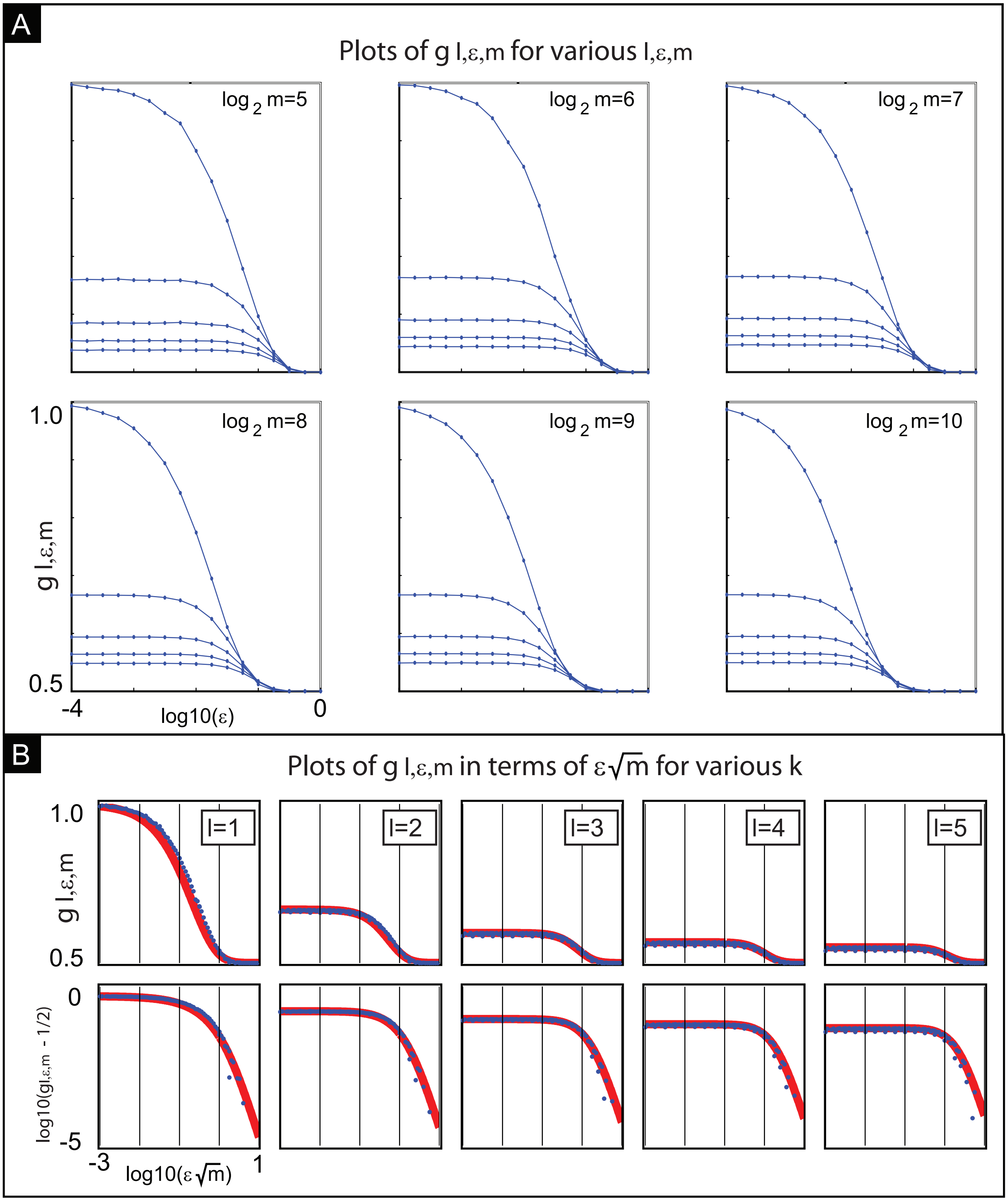

Figure 23: Numerical experiments confirming the behavior of $g_{l,\varepsilon,m}$. In Panel-A we show several curves for $g_{l,\varepsilon,m}$ as a function of ε for various m. Each subplot contains curves for $l = 1, 2, \ldots, 5$, with the higher curves corresponding to lower values of l. In Panel-B we rearrange the data from our numerical experiments as a function of $\varepsilon\sqrt{m}$. We show this data for $g_{l,\varepsilon,m}$ on top and $\log_{10}(g_{l,\varepsilon,m} - 0.5)$ on the bottom. Overlayed on these numerical experiments is the large-m approximation for $g_{l,\varepsilon,m}$ derived above (red curve).

different distribution than the entries of the covariance matrices $BB^\intercal$ or $\tilde{B}\tilde{B}^\intercal$. Nevertheless, the mean-squared-covariance of $\hat{B}$ will (on average) match the mean-squared-covariance of B and $\tilde{B}$. That is to say, the average value (across multiple trials) of $\text{mean}\Big(\text{diag}\left(\hat{B}\hat{B}^\intercal\hat{B}\hat{B}^\intercal\right)\Big)$ will equal the average value (across multiple trials) of $\text{mean}(\text{diag}\,(BB^\intercal BB^\intercal))$, as well as the average value of $\text{mean}\Big(\text{diag}\left(\tilde{B}\tilde{B}^\intercal\tilde{B}\tilde{B}^\intercal\right)\Big)$. This is equivalent to saying that the average row-score $[Z_{\text{ROW}}]_j$ of a row from $\hat{B}$ will equal the average row-score of a row from B or $\tilde{B}$; a similar statement holds for the column-scores $[Z_{\text{COL}}]_k$. Put in more colloquial terms, the average level of coherence between rows of $\hat{B}$ is the same as the average level of coherence between rows of B or $\tilde{B}$. Thus, we see little difference in our numerical experiments if we use $B = \text{sign}(A)$, $\tilde{B} = \text{sign}(\tilde{A})$ or $\hat{B} = \hat{A}$.

Finally, we note that all the matrices generated above will have roughly the same number of positive and negative entries. That is to say, the fraction of positive entries (i.e., sparsity-coefficient) will be $p \sim 0.5$, and the fraction of negative entries will be $q = 1 - p \sim 0.5$. If we wish to generate a rank-1 matrix that has, say, a sparsity-coefficient $\tilde{p} < 0.5$ (i.e., more negative entries than positive entries), then we can use a slightly different algorithm, described in section 5.2.

5.2 Generating a sparse low-rank B

To generate a rank-1 error-ε matrix that has, say, a sparsity-coefficient $\tilde{p} < 0.5$ (i.e., more negative entries than positive entries), then we take the following shortcut:

1. Given ε, determine $g := g_{1,\varepsilon,m}$ and $f := f_{\varepsilon\sqrt{m}}$ as in the construction of $\hat{A}$ in section 5.1.
2. Given $\tilde{p}$, Set probability $\bar{f} = \min(f, \tilde{p})$, and $h = \left(\tilde{p} - \bar{f}\right) / \left(1 - 2\bar{f}\right)$.
3. Set probability $\hat{p} = 0.5 - 0.5\sqrt{1 - 2\min(h, 1-h)}$ and $\hat{q} = 1 - \hat{p}$
4. Generate an $m \times 1$ binary vector $\vec{u}$ by choosing each entry to be either $+1$ or -1, with probability $\hat{p}$ and $\hat{q}$, respectively.
5. Generate an $n \times 1$ binary-vector $\vec{v}$ by choosing each entry to be either $+1$ or -1, with probability $\hat{q}$ and $\hat{p}$, respectively. Note that the sign-associations for $\vec{v}$ are opposite to those of $\vec{u}$.
6. Form $\overline{A} = \vec{u} \times \vec{v}^\intercal$.
7. Choose $\bar{f}mn$ of the entries of $\overline{A}$ at random and flip their sign.
8. Define $\overline{B} = \overline{A}$.

This method will generate a binary-matrix $\overline{B}$ that has a fraction $\tilde{p}$ of positive entries. While the spectrum of $\overline{B}$ will not look like the spectrum of B or $\tilde{B}$ from section 5.1, a loop drawn from $\overline{B}$ will have the same probability $g_{1,\varepsilon,m}$ of being rank-1.

If $\tilde{p}$ were to be > 0.5 instead of < 0.5, then we could perform the above procedure for a sparsity-coefficient of $1 - \tilde{p}$, and then negate the result.

6 Application to the Planted-Bicluster problem:

The algorithm we propose in section 2 is often capable of solving the 'planted bicluster' problem. As a demonstration, we consider a large $M \times M$ data-matrix matrix D with entries chosen independently from a distribution with median 0. After creating D we'll embed within D an $m \times m$ submatrix B, which is rank-1 with error ε (as described in section 5). We'll assume that $M \gg m \gg 1$. Our algorithm will detect B with high probability whenever $m^3\left(2g_{1,\varepsilon,m} - 1\right) \gtrsim \sqrt{2M^3}$. This means that, when $\varepsilon\sqrt{m} < 1$, our algorithm generally will work well if $m \gtrsim \sqrt{M}$, but not if $m = o(\sqrt{M})$. In addition, if $\varepsilon\sqrt{m} > 1$, then (by using the asymptotic formula for $g_{1,\varepsilon,m}$ in section 17) we see that our algorithm will work well if $m \gtrsim \sqrt[3]{\pi^2/4} \cdot [\varepsilon\sqrt{m}]^{4/3} \cdot \sqrt{M}$. However, as commented on above, most applications in gene-expression analysis do not involve sufficiently many patients (and do not have sufficiently large biclusters) for this second statement to be very meaningful. If we limit ourselves to the typical ranges of m and M which we see in most applications, we can state that our algorithm works well when the following two detection-thresholds are met:

Size-Threshold: m should be $\gtrsim \sqrt{M}$, and **Noise-Threshold**: $\varepsilon\sqrt{m}$ should be $\lesssim 1$.

These two conditions represent two distinct detection-thresholds associated with our algorithm (see Figs 24, 25 and 26). Analogous detection-thresholds can be derived using $g_{l,\varepsilon,m}$ when the planted bicluster B is numerical-rank l instead of 1 (see Figs 30 and 31).

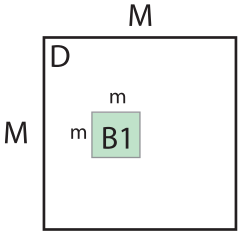

Figure 24: This figure illustrates the setup for the numerical experiments shown in Fig 26. These numerical experiments illustrate the performance of our algorithm on a simple planted-bicluster problem involving case-patients only (i.e., D only). For each numerical experiment we let D be a large $M \times M$ random matrix with entries chosen independently from a distribution with median 0. We implant within D a smaller $m \times m$ bicluster B that is rank-1 with error ε (see discussion at the end of section 5, as well as Fig 25). Our algorithm produces a list of row- and column-indices of D in the order in which they are eliminated; those rows and columns retained the longest are expected to be members of B. For each numerical experiment we calculate the auc A_R (i.e., area under the receiver operator characteristic curve) associated with the row-indices of B with respect to the output list from our algorithm. The value A_R is equal to the probability that: given a randomly chosen row from B as well as a randomly chosen row from outside of B, our algorithm eliminates the latter before the former (i.e., the latter is lower on our list than the former). This value A_R is equal to $(\bar{r} - (m+1)/2) / (M - m)$, where $\bar{r}$ is the average rank of the rows of B within our list. We calculate the auc A_C for the columns similarly. Finally, we use $A = (A_R + A_C)/2$ as a metric of success; values of A near 1 mean that the rows and columns of B were filtered to the top by our algorithm. Values of A near 0.5 mean that our algorithm failed to detect B.

We note that these two detection-thresholds are not the same as the 'information-theoretic' phase-transitions for the planted-bicluster problem. That is to say, our algorithm cannot typically detect biclusters with a size $m \ll \sqrt{M}$ or a noise $\varepsilon\sqrt{m} \gg 1$, even though such biclusters might be very significant (in a statistical sense). For a more detailed discussion regarding these information-theoretic phase-transitions see section 19.

Behavior when D is correlated: Our analysis above also sheds light on situations when D is itself numerically low-rank (instead of having independent entries). To discuss this situation, let's imagine that the $M \times M$ matrix D were numerical-rank l_D with error ε_D prior to binarization. We'll embed within D an $m \times m$ planted-bicluster B of numerical-rank l_B with error ε_B, involving the row-subset J_B and column-subset K_B. Let's refer to the matrix-entries of D that are not in B as $B^{\complement}$.

Just as before, a typical loop drawn from B will be rank-1 with probability $g_{l_B,\varepsilon_B,m} > 1/2$. However, unlike before, a typical loop drawn from D will also be rank-1 with probability $g_{l_D,\varepsilon_D,M} > 1/2$. This means that the overall 'signal' coming from B now has to compete with a signal coming from D. In fact, the typical row-score of a row in $J_{B^{\complement}}$ is no longer 0, but now has an expected-value of

$$\widetilde{\sum_{j \in J_{B^{\complement}}}} [Z_{\text{ROW}}]_j \sim M^3 \left(2g_{l_D,\varepsilon_D,M} - 1\right),$$

which we'll denote by $\bar{Z}$. In addition, the standard-deviation of the row-score across rows in $J_{B^{\complement}}$ is no longer simply $\sqrt{2M^3}$, but is now a function of $\varepsilon_D\sqrt{M}$, as well as l_D, which we'll denote by σ_Z. Our algorithm will detect B with high probability only when the typical value of a row-score from J_B is on the same order as (or larger than) $\bar{Z} + \sigma_Z$. If $\varepsilon_D\sqrt{M}$ and l_D are sufficiently large, then $\bar{Z} \sim 0$ and $\sigma_Z \sim \sqrt{2M^3}$; the detection-thresholds for our method will be comparable to the case where the entries of D are drawn independently. On the other hand, if l_D and $\varepsilon_D\sqrt{M}$ are sufficiently small (e.g., $l_D = 1$ and $\varepsilon_D\sqrt{M} \sim 0.1$), then $\bar{Z} + \sigma_Z$ can be several orders of magnitude larger than $\sqrt{2M^3}$, and our method will fail unless $m = O(M)$.

7 More detailed discussion of the algorithm:

As described in section 2, our algorithm has three main components: (i) Binarization, (ii) calculation of loop-scores, and (iii) iteration. In this section we discuss these three main components in more detail, providing some justification for our choices. In this section we'll often (but not always) use the planted-bicluster problem to frame our discussion. We'll also sometimes compare our algorithm to a simple spectral algorithm (discussed in more detail within section 22).

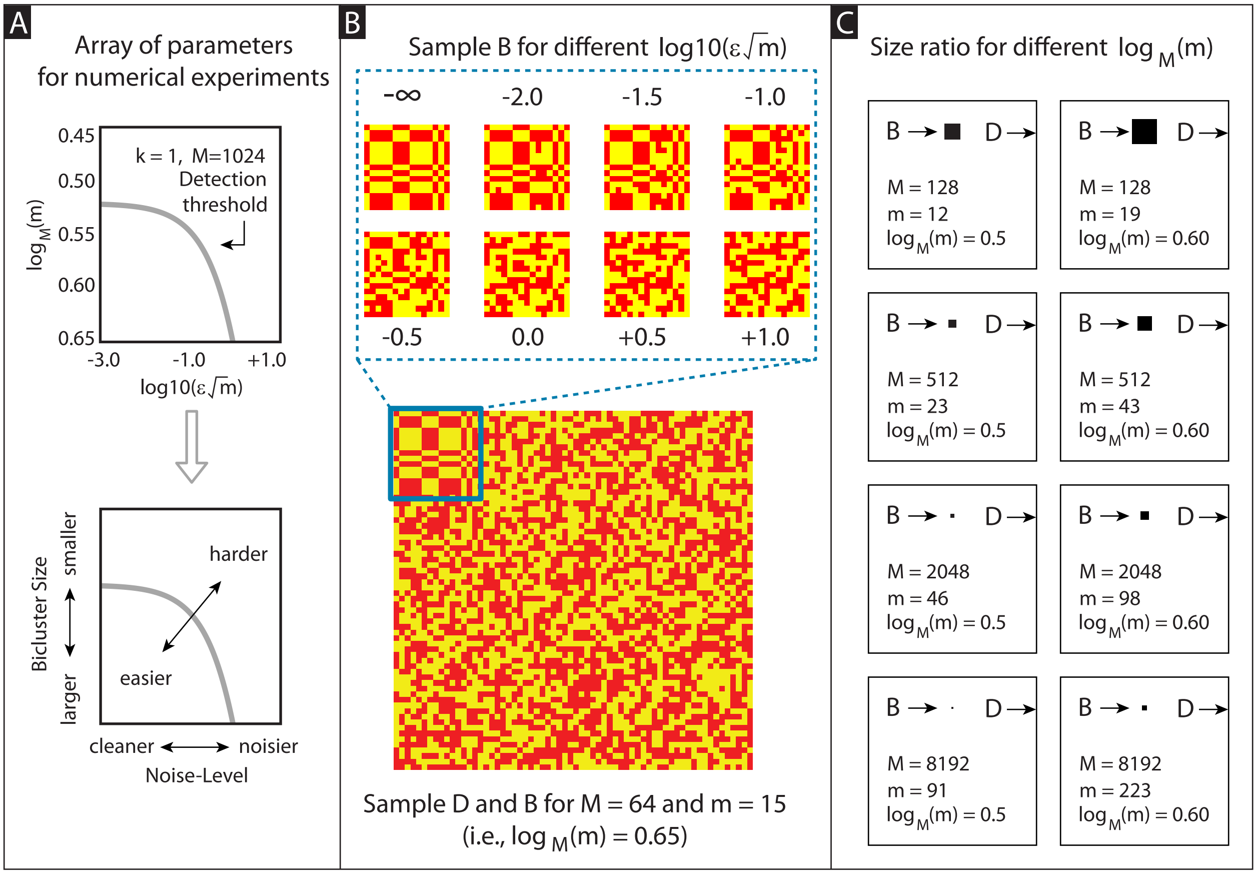

Figure 25: This figure describes some of the qualitative features of the numerical experiments shown in Fig 26. In Panel-A we show the array of parameter values used for $\varepsilon\sqrt{m}$ and $\log_M(m)$ within our numerical experiments. The first of these parameters controls the noise-level (horizontal axis), while the second controls the bicluster size (vertical axis). Situations where the noise-level is low and the bicluster-size is large correspond to planted-biclusters which are 'easier' to find. By contrast, situations where the noise-level is large and the bicluster-size is small correspond to planted-biclusters which are 'harder' to find. The thin grey line shown in Panel-A corresponds to the detection-threshold of our loop-counting algorithm when applied to the planted-bicluster problem with rank $l = 1$ and $M = 1024$ (see Fig 26). Our loop-counting algorithm typically succeeds at solving planted-bicluster problems that are on the 'easy side' of this detection-threshold. In Panel-B we show a sample matrix D of size $M = 64$ used for our numerical experiments. We have implanted within D a rank-1 bicluster B of size $m = 15$, corresponding to $\log_M(m) = 0.65$ and $\varepsilon\sqrt{m} = 0$. For illustration, we have rearranged the rows and columns of D to reveal B in the upper-left corner (see cyan box). This bicluster is large and the noise-level is very low, so finding B is very easy (similar to the lower left corner of our parameter-array). If we were to increase the noise-level, the bicluster B would no longer be exactly rank-1; sample biclusters with varying noise-level are shown above. In Panel-C we illustrate the size-ratio between D and B for different values of M and the parameter $\log_M(m)$. In each case the outer box (not filled) represents the size of D, whereas the interior box (filled) represents the size of B. Note that, when M is large and $\log_M(m)$ is small, the bicluster B can be quite a bit smaller than D.

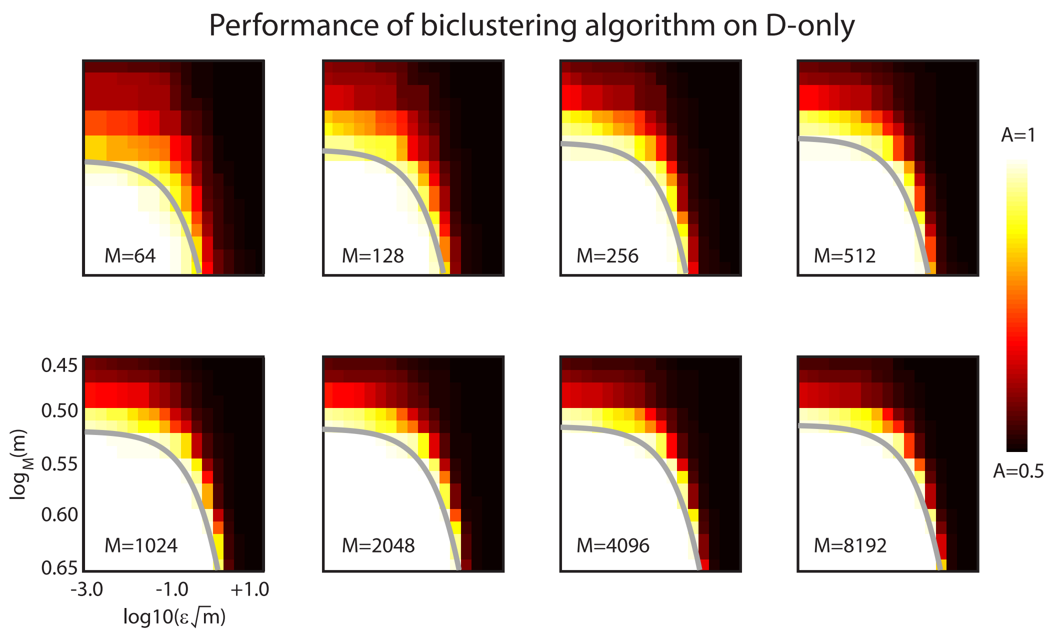

Figure 26: These numerical experiments illustrate the performance of our algorithm on a simple planted-bicluster problem involving case-patients only (see Figs 24 and 25). In the panels shown we plot the trial-averaged value of A as a function of $\varepsilon\sqrt{m}$ and $\log_M(m)$. Each subplot takes the form of a heatmap, with each pixel showing the value of A for a given value of $\log_{10}(\varepsilon\sqrt{m})$ and $\log_M(m)$ (averaged over at least 32 trials) . The different subplots correspond to different values for M. Our algorithm is generally successful when $\log_{10}(\varepsilon\sqrt{m}) \lesssim 0$ and $\log_M(m) \gtrsim 0.5$. The grey line superimposed on the heatmap is the detection-boundary calculated from the formula $m^3(2g_{1,\varepsilon,m} - 1) = \sqrt{2M^3}$. See Figs 30 and 31 for similar results pertaining to rank-2 and rank-3 planted-biclusters.

7.1 Why binarize? Advantages and disadvantages

The first step of our algorithm is to binarize the data: that is, to send each entry of D to -1 or $+1$, depending on its sign. This binarization step carries with it two main advantages.

Speed: The first and most obvious advantage is speed: A binarized matrix can be stored efficiently, and calculating row- and column-scores for a binarized matrix can be accomplished without use of floating-point operations (instead using bitwise operation such as xor). For example, storing a large data-matrix containing 2 million measurements taken across 2 thousand patients, requires 32 GB of memory to store in double-precision, but only 500 MB after binarization. Calculating simple vector-vector and matrix-vector products associated with such a binarized matrix can be $\gtrsim 64$ times faster than analogous operations performed in double-precision.

Normalization: Binarization serves as a canonical normalization which amplifies signals that are sharp, but of low magnitude. This comes at a small cost (which we'll touch on at the end of this section), but serves to expose many kinds of structures that would otherwise be difficult to detect.

To set the stage for this discussion, let's consider a large data-matrix D comprising N gene-expression measurements taken across M patients. A priori, we expect the entries of D to be mostly uncorrelated (or, say, weakly correlated). This is because, generally speaking, there will be multiple complicated biological mechanisms driving the gene-expression within D. Some mechanisms work together, others are antagonistic, while others are independent; we typically expect the accumulation of all these different mechanisms to act like a source of 'noise', de-correlating the genes in D.

Now let's assume that D contains an $m \times n$ low-rank bicluster B spanning row-subset J_B and column-subset K_B. The low-rank structure of B implies that, unlike the typical genes in D, the genes in K_B are strongly correlated (i.e., co-expressed) across the patients in J_B. The success of our methodology depends on how – and why – the genes in B are correlated.

More specifically, if B were indeed biologically significant, then we would expect there to be some underlying physiological reason for the co-expression within B. While there are many different possible mechanisms which might underly this co-expression (i.e., the drivers of gene-expression can be very complicated), we can abstractly group these mechanisms into two rough paradigms:

Spectral-amplification In this first paradigm we imagine that the mechanisms driving the co-expression in B do so by *adding* a new strong source of co-variation to the genes in K_B.

Spectral-suppression For the second paradigm we imagine that the mechanisms driving the co-expression in B do so by *removing* many of the small weak sources of noise which would ordinarily de-correlate the genes in K_B.

These two paradigms can be idealized mathematically with the following two generative models:

Spectral-amplification The low-rank bicluster B could be produced by amplifying low-order spectral information. For example, we can initially draw the entries of B from a noisy (uncorrelated) distribution. Once drawn, we then increase the magnitude of the low-order (i.e., dominant) principal-components of B, while leaving the high-order (i.e., less dominant) principal-components unchanged. This process will magnify the first few principal-components of B, adding a large new source of covariance to the bicluster's genes.

Spectral-suppression The bicluster B could instead be produced by suppressing high-order spectral information. For example, as above, we can start by drawing the entries of B from a noisy distribution. This time, however, we subsequently decrease the magnitude of the high-order principal-components of B, leaving the low-order principal-components unchanged. This process will end up introducing correlations into B by removing (or quenching) many of the smaller sources of variance that would have otherwise decorrelated the bicluster's genes.

Both of the paradigms described above give rise to low-rank biclusters, but with rather different properties. Foremost among these differences is that spectrally-suppressed biclusters are harder to detect than spectrally-amplified biclusters. The reason for this is that the rows and columns of a spectrally-suppressed bicluster are typically not large, and are usually buried within the distribution associated with the rest of the data. By contrast, the rows and columns of a spectrally-amplified bicluster are typically large, and often stick out of the rest of the data. Consequently, a spectrally-amplified bicluster can usually be detected simply by looking for 'outliers' in the data (e.g., a simple search based on entry-by-entry amplitude). Indeed, if the original data set lacks an abundance of outliers, one can usually rule out the existence of spectrally-amplified biclusters, leaving the harder task of detecting any hidden spectrally-suppressed biclusters.

It is for these reasons that we've designed our algorithm with the goal of detecting spectrally-suppressed biclusters. One of the major issues that arises when trying to detect spectrally-suppressed biclusters is that, even if a spectrally-suppressed bicluster has a 'sharp' Pearson's correlation (i.e., pairs of rows or columns within the bicluster have a normalized correlation close to ± 1), the actual covariances of that bicluster (i.e., dot-products of rows or columns) will typically be low in magnitude. Consequently, without some kind of normalization, the signal associated with a spectrally-suppressed bicluster will be proportional to its low-amplitude covariance, rather than to its possibly-high Pearson's correlation. Our binarization step rectifies this problem by putting the spectrally-suppressed bicluster on a similar footing with the rest of the data, allowing our loop-scores to detect it. An example of this kind of phenomenon is given in Fig 27. Note that the unbinarized loop-scores are uninformative, whereas the loop-scores after binarization expose the spectrally-suppressed bicluster.

We remark that our binarization step does indeed reduce the magnitude of the entries within any spectrally-amplified biclusters. However, because these biclusters were so easy to detect to begin with, we believe that binarization is an appropriate choice for most applications (i.e., when spectrally-amplified biclusters have already been ruled out).

Information loss?: It turns out that, in addition to obscuring signals associated with spectrally-amplified biclusters, binarization can also obscure the signals produced by biclusters that have essentially no noise. We'll argue later that this isn't a serious issue, but for now let's consider an 'essentially noiseless' bicluster with $\varepsilon\sqrt{m} \sim 0$. Regardless of whether this bicluster has been created through spectral-amplification or spectral-suppression, it can be easily detected prior to binarization using the following simple 'angle-matching' algorithm: This angle-matching algorithm involves sifting through the loops in the data-matrix, associating with each loop the angle θ between its two rows. Most loops will have an angle that ranges across the interval $[-\pi, +\pi]$, whereas the loops within the bicluster will have an angle that is very close to either 0 or $\pm\pi$. The bicluster can then be picked out simply by retaining the loops with $\theta \in \{0, \pm\pi\}$. The reason this angle-matching algorithm works is because, when $\varepsilon\sqrt{m} \sim 0$, there is a great deal of angular information in the distribution of θ. In this idealized no-noise scenario, binarizing the data destroys this extra angular information, obscuring the bicluster and preventing the angle-matching algorithm from working well.

We can quantify this phenomenon more precisely using the reasoning of section 5. Assuming that the bicluster B is defined as described in section 5, a pair of randomly chosen columns within the bicluster gives rise to a 2-dimensional distribution of the form $\tilde{\rho}$; i.e., each 1×2 row-vector within this column-pair is drawn from $\tilde{\rho}$. Consequently, $\tilde{\rho}$ induces a distribution $\rho_B(\omega)$ on the angle ω of each row-vector drawn from $\tilde{\rho}$ (see Fig 28A). Note that the angle θ associated with any loop in this column-pair will take the form of $\theta = \omega_1 - \omega_2$, with both ω_1, ω_2 drawn from $\rho_B(\omega)$; i.e., θ is drawn from $\rho_B \star \rho_B^c$, where $\rho_B^c(\omega) = \rho_B(-\omega)$.

The angular-information contained within this distribution of θ is thus derived from the angular-information contained within $\rho_B(\omega)$. When $\varepsilon\sqrt{m}$ is very tiny the 2-dimensional distribution $\tilde{\rho}$ will be a very eccentric ellipse dominated by its first principal-component; the angular-distribution $\rho_B(\omega)$ will be very sharply peaked around the major-axis of this ellipse, and the angular-distribution of θ will be peaked around $\{0, \pm\pi\}$. The angular-information contained within $\rho_B(\omega)$ can

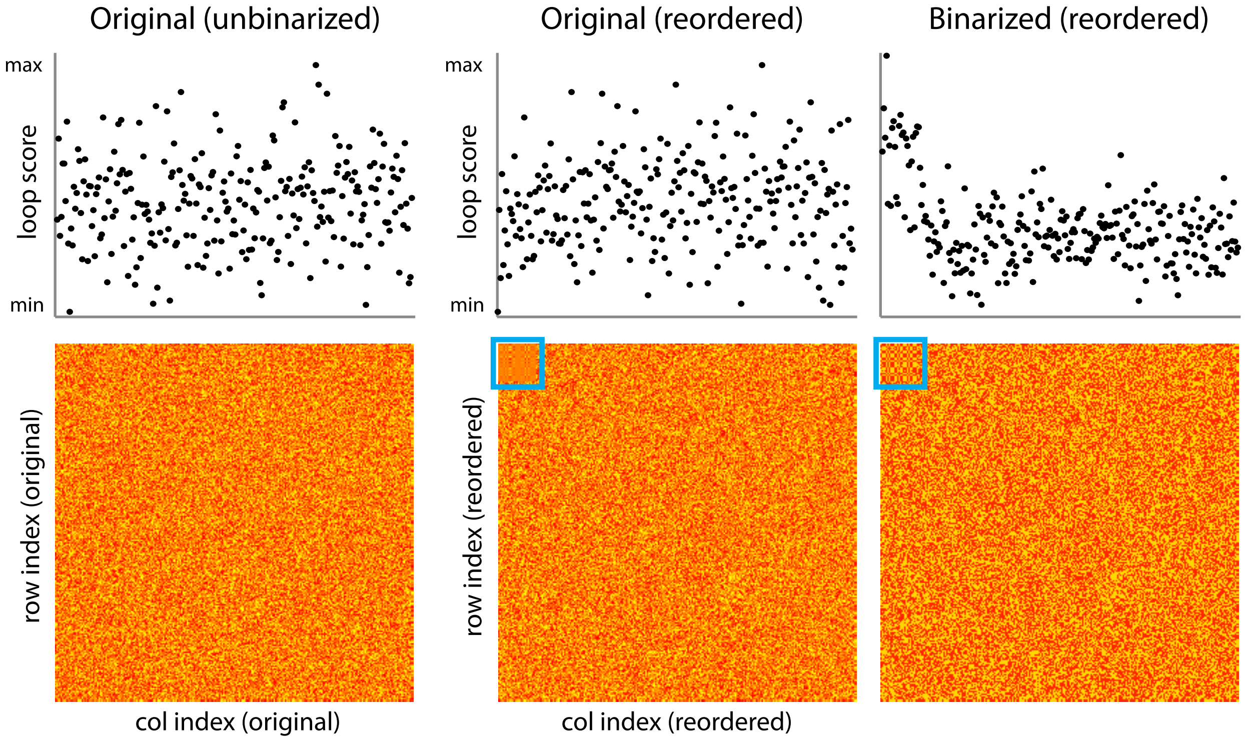

Figure 27: Binarization helps reveal spectrally suppressed biclusters. On the left we show a random matrix D (bottom) in which a spectrally-suppressed bicluster B has been created. In the middle (bottom) we rearrange the rows and columns of D to reveal the embedded bicluster B in the upper left corner (cyan square). The bicluster B was created by first choosing a subset of rows and columns of D. We then calculated a principal-component-analysis $USV^\intercal$ of this submatrix. We then reduced the high-order principal-values $S_{2,2}$, $S_{3,3}$, etc. by a factor of 10, while retaining the low-order principal-value $S_{1,1}$. We then reconstituted $B = USV^\intercal$, and reinserted B into D. The column-scores $[Z_{\text{COL}}] = \text{diag}(D^\intercal D D^\intercal D)$ for each column of the (unbinarized) data-matrix D are show above each matrix. Note that – prior to binarization – the column-scores do not clearly indicate the columns which constitute the bicluster B (neither would scores produced by the simple spectral biclustering method applied to this non-binarized version of D). On the right (bottom) we show the binarized matrix $D := \text{sign}(D) = 2(D > 0) - 1$, with the rows and columns reorganized to reveal B. The column-scores for each column of the (binarized) data-matrix D are shown above; note that – post binarization – the column-scores associated with D do indicate the columns of B (as would scores produced by the simple spectral method). The row-scores exhibit a similar phenomenon.

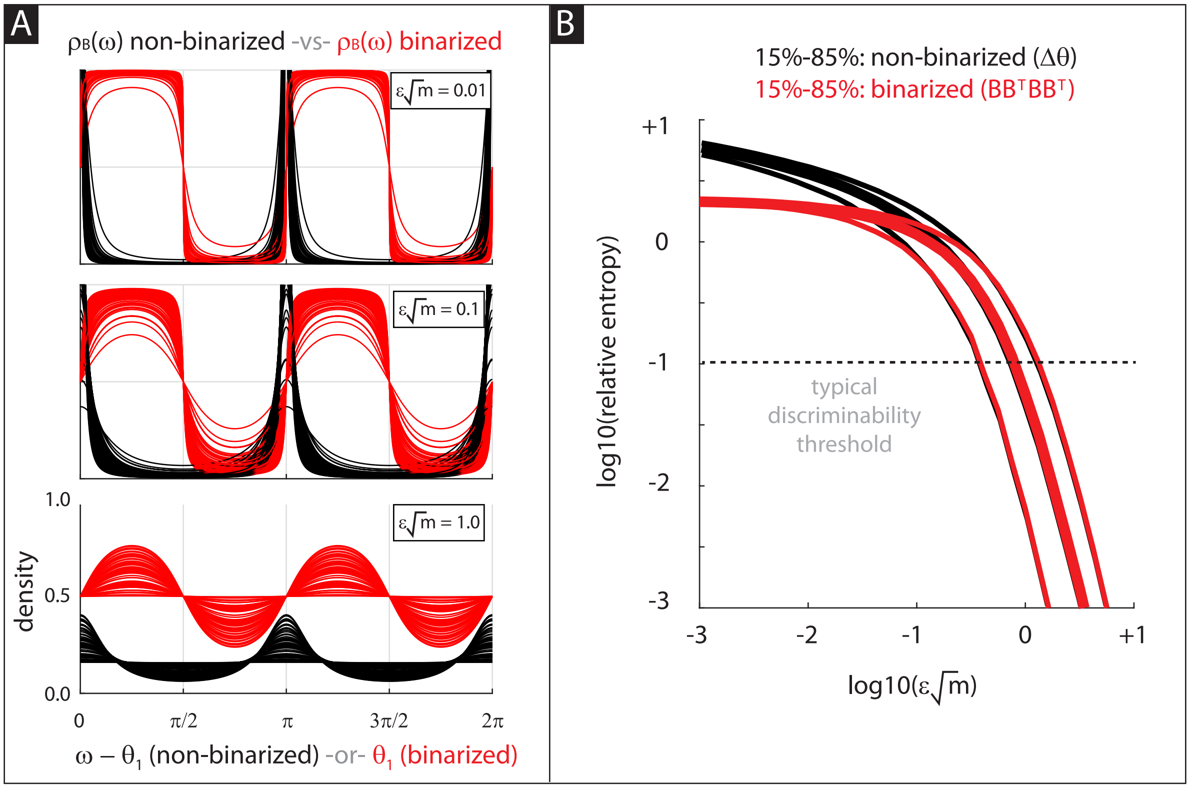

Figure 28: In panel-A we show a few sample distributions $\rho_B(\omega)$ associated with $m = 128$ and various values of $\varepsilon\sqrt{m}$. The original distribution $\rho_B(\omega)$ is shown in black (as a function of $\omega - \theta_1$), and the binarized version is shown in red (as a function of θ_1, assuming ω is in the first or third quadrant). Note that $\rho_B(\omega)$ becomes more and more similar to the uniform distribution $\rho_D(\omega)$ (not shown) as $\varepsilon\sqrt{m}$ increases. In panel-B we show the typical relative-entropy $\mathcal{D}_{KL}(\varepsilon\sqrt{m})$ for a large number of samples of non-binarized (black) and binarized (red) random rank-1 matrices (with m fixed at $m = 128$). We show the median, 15th and 85th percentiles of $\mathcal{D}_{KL}$ as a function of $\varepsilon\sqrt{m}$. Note that binarization makes little difference for $\varepsilon\sqrt{m} \gtrsim 1/10$. Even though this particular figure was generated with $m = 128$, changing the value of m makes little qualitative difference; binarization has essentially no detrimental effect as long as $\varepsilon\sqrt{m} \gtrsim 1/10$.

be quantified via the relative-entropy $\mathcal{D}_{KL}(\varepsilon\sqrt{m}) = \int \rho_B \log(\rho_B/\rho_D)d\omega$, which compares ρ_B to the uniform-distribution $\rho_D\,(\omega)$ expected outside of the bicluster. As we alluded to above, when $\varepsilon\sqrt{m} \sim 0$ this relative-entropy $\mathcal{D}_{KL}$ is very high for the original data, but is much lower for the binarized data (i.e., where half the loops in the data-matrix have $\theta = 0$ or $\pm\pi$). In this situation binarizing the data destroys this extra angular information, dramatically reducing the relative-entropy.

It turns out, however, that this phenomenon is restricted only to essentially noiseless biclusters. When $\varepsilon\sqrt{m}$ is significantly larger than 0 (say, $\gtrsim 0.01$), then the distribution $\tilde{\rho}$ is not so eccentric; the angular-distribution $\rho_B\,(\omega)$ will be distributed more evenly, and the distribution of θ will be closer to uniform. Thus, when $\varepsilon\sqrt{m} \gtrsim 0.01$, the relative-entropy $\mathcal{D}_{KL}$ is not much higher prior to binarization than afterwards. In fact, when $\varepsilon\sqrt{m} \gtrsim 1/10$ there is essentially no loss of angular information after binarization. We illustrate this point in Fig 28A, which shows many samples of $\rho_B(\omega)$ both pre- and post-binarization, as well as Fig 28B, which shows $\mathcal{D}_{KL}\,(\varepsilon\sqrt{m})$ sampled across many rank-1 biclusters (the details involved in generating Fig 28 are described in section 20). Consequently, if we expect our data to contain realistic biclusters (i.e., with $\varepsilon\sqrt{m} \gtrsim 1/10$), we don't have to consider any sort of angle-matching algorithm (which would be useless in practice), and we can binarize our data without fearing any information-loss.

In summary: We expect that realistic data-sets will not contain perfectly correlated biclusters; for realistic biclusters $\varepsilon\sqrt{m}$ will be on the order of 0.1, at which point binarization does not destroy a significant amount of angular-information. In addition, binarization has the added advantage of amplifying the signals produced by any spectrally-suppressed biclusters. While binarization does suppress the signals of any spectrally-amplified biclusters, these can usually be detected simply by looking for outliers in the data.

Finally, we remark that the binarization-step in our algorithm can be modified to accommodate a 'soft' binarization, wherein some values in the data-array are set to an intermediate number, rather than to ± 1. For example, if there is some reason to suspect that the noise within the data involves small-amplitude structured perturbations around the binarization-threshold, then all entries in the data-array close to the binarization-threshold can be set to 0.

7.2 Why use loops? Advantages and disadvantages

The second step of our algorithm involves calculating the loop-scores. These loop-scores will be indicative of the location of any hidden low-rank biclusters, provided that these hidden biclusters are sufficiently large (and not too noisy). These loop-scores have the obvious advantage that they are easy to calculate and, as we'll see later, it is easy to correct them for case-control status and various covariates. It is also the case that the loop-scores are 'close to optimal' regarding the size-transition in the low-noise limit; within the limits of our framework it is difficult to construct a more sophisticated score that is much better at detecting small planted-biclusters.

To see why this might be the case let's return to a special case of the planted-bicluster problem: let's assume that we have a large $M \times M$ binary-matrix D containing a planted-bicluster B of size $m \times m$ which is exactly rank-1 (i.e., $\varepsilon\sqrt{m} = 0$). These 'low-noise' biclusters are the easiest to detect, and will allow us to focus on the size-transition associated with the planted-bicluster problem. In terms of notation, let's say that the bicluster B is formed via $B = uv^{\intercal}$, where u and v are both random binary vectors of size m. The bicluster B is then implanted into D using the randomly chosen row- and column-subsets J_B and K_B (each of size m).

Recall that our algorithm proceeds by calculating the 'loop-scores' associated with each row and column of D. For the purposes of this discussion, let's use $l\,[j_1, j_2; k_1, k_2]$ to denote the 'loop' (i.e., 2×2 submatrix) spanning rows j_1 and j_2 as well as columns k_1 and k_2. Our loop-scores $[Z_{\text{ROW}}]_j$ for a given row j are formed as follows. First, in Step (i), we consider the set S_j of all loops $l\,[j_1, j_2; k_1, k_2]$ with $j \in \{j_1, j_2\}$. Then, in Step (ii), we measure something like the rank $\mu\,(l)$ for each $l \in S_j$; in our case $\mu\,(l)$ is chosen to be 3 minus twice the rank of l. Finally, in Step (iii), we aggregate the sum $[Z_{\text{ROW}}]_j = \sum_{l \in S_j} \mu\,(l)$.

Our hope is that the loop-scores Z_{ROW} (and also Z_{COL}, which are constructed similarly) are indicative of the rows J_B (and columns K_B) of B. As we've seen above, our loop-scores will be informative when the size of B is larger than the detection-threshold $\sqrt{M}$ (i.e., when $m \gtrsim \sqrt{M}$). A natural question is: are there better ways to score each row and column of D? Could we do better than the size-threshold of $\sqrt{M}$? Note that, for any given row j, our loop-score is one of a large class of 'c-scores' which are constructed using the following general template:

Step (i) Consider the set S_j of all $c \times c$-submatrices $l\,[j_1, \ldots, j_c; k_1, \ldots, k_c]$ with $j \in \{j_1, \ldots, j_c\}$.

Step (ii) Measure some scalar quantity $\mu\,(l)$ for each $l \in S_j$;

Step (iii) Aggregate the sum $[Z_{\text{ROW}}]_j = \sum_{l \in S_j} \mu\,(l)$.

Perhaps we could gain an advantage if we allowed for the more general steps (i) and (ii) above? Specifically, instead of merely looking at loops $l\,[j_1, j_2; k_1, k_2]$, we might consider larger $c \times c$ submatrices $l\,[j_1, \ldots, j_c; k_1, \ldots, k_c]$. Moreover, instead of simply measuring the rank $\mu\,(l)$, we could use some more informative measurement instead (e.g., any other scalar-valued function $\mu : \{-1, +1\}^{c \times c} \to \mathbb{R}$). It turns out that neither of these modifications can substantially improve the c-score; no matter which fixed c we choose, or how we construct μ, we won't be able to overcome the size-threshold of $\sqrt{M}$.

More specifically, we argue that – for fixed c and μ in the limit as m and M go to ∞ – no c-score will be informative when $m = o(\sqrt{M})$. The details of our argument can be found in section 21. We remark that our analysis only applies asymptotically, and does not guarantee that our method is the 'best' when m is on the order of $\sqrt{M}$ (e.g., if m were to be a constant multiple of $\sqrt{M}$ in the limit). Nevertheless, we believe that our method performs adequately when $m \sim \sqrt{M}$. For a demonstration of our method applied to the planted-bicluster problem, see Fig 29, as well as Figs 30 and 31, and section 22.

We should also emphasize that one can certainly design algorithms that perform better than ours on the planted-bicluster problem by stepping outside the restrictions of the template above. For example, one could consider the joint-distribution across multiple rows or columns of various $c \times c$-submatrices. Such an algorithm would avoid the simple aggregation in step (iii), and may involve constructing a score which depends on multiple row- and column-indices, rather than just one. The reason we do not consider such algorithms is because it is not yet clear to us how to make them competitive in terms of speed and storage.

Alternatively, one could consider a simple spectral method which computes scores related to the coefficients of the dominant singular-vectors of D. Such spectral scores adopt a very specific μ, but involve an unbounded c (i.e., $c \to \infty$), thus avoiding the finite-c restriction in step (i) of the template above (and potentially allowing for better performance). The reason that we do not pursue spectral methods is because the spectral scores are usually less useful than our loop-scores when attempting to locate small biclusters. This phenomenon is illustrated in Figs 29, 30, 31 and is discussed in more detail in section 22.

A more general approach is to consider a message-passing algorithm akin to [$\mathcal{M}$33], [$\mathcal{M}$19], or [$\mathcal{M}$41] These message-passing algorithms operate by (i) setting up a bigraph between the rows and columns of the data matrix, (ii) iteratively accumulating a nonlinear function of 'messages' between nodes and edges in the bigraph, and (iii) adaptively refining this nonlinearity over each iteration to maximize the signal to noise ratio in the data. Such an approach sidesteps many of the

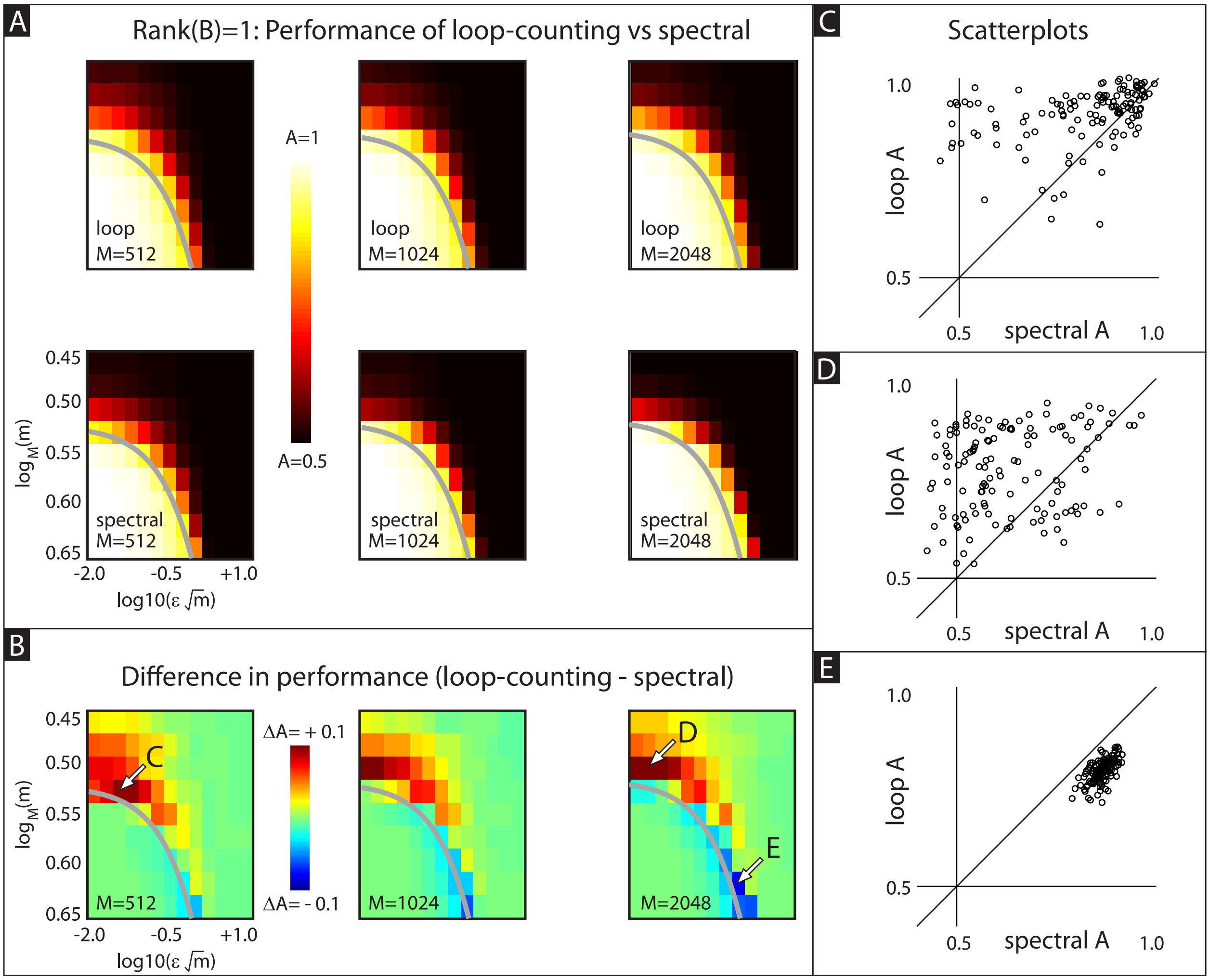

Figure 29: Performance of loop-counting vs spectral-biclustering applied to the rank-1 planted-bicluster problem. The format for this figure is similar to Fig 26. On top we show the trial-averaged auc A for our loop-counting method. In the middle we show the analogous auc for a simple implementation of the spectral method. For this simple spectral method we take the row- or column-score to be the entrywise-square of the first row- or column-wise principal vector of the binarized data (see section 22). On the bottom we show the difference in trial-averaged A between these two methods (see colorbar on right for scale). Note that, when the noise is small (i.e., when $\varepsilon\sqrt{m} \lesssim 1/3$), our loop-counting method has a higher rate of success than the spectral method. On the other hand, when the noise is large (i.e., $\varepsilon\sqrt{m} \gtrsim 1/3$) the spectral method has a higher rate of success. The thin grey line shows the detection-boundary for our loop-counting method (calculated using $m^3 \left(2g_{1,\varepsilon,m} - 1\right) = \sqrt{2M^3}$). Panels C,D,E show a few scatterplots of some of the same data used to construct the pictures in panels A and B. More specifically, Panel-C shows the results of 128 trials performed with $M = 512$, $\log_M(m) = 0.525$, and $\log_{10}(\varepsilon\sqrt{m}) = -1.5$ (indicated with an arrowhead in Panel-B). For each of these trials we plot the A-value for the spectral-method on the horizontal-axis, and the A-value for the loop-counting method on the vertical-axis. For this choice of parameters the loop-counting method typically achieves moderate success (e.g., $A \geq 0.75$), whereas the spectral method sometimes fails completely (e.g., $A \sim 0.5$). Panels D and E show similar scatter plots for other parameter values (indicated with arrowheads in Panel-B).

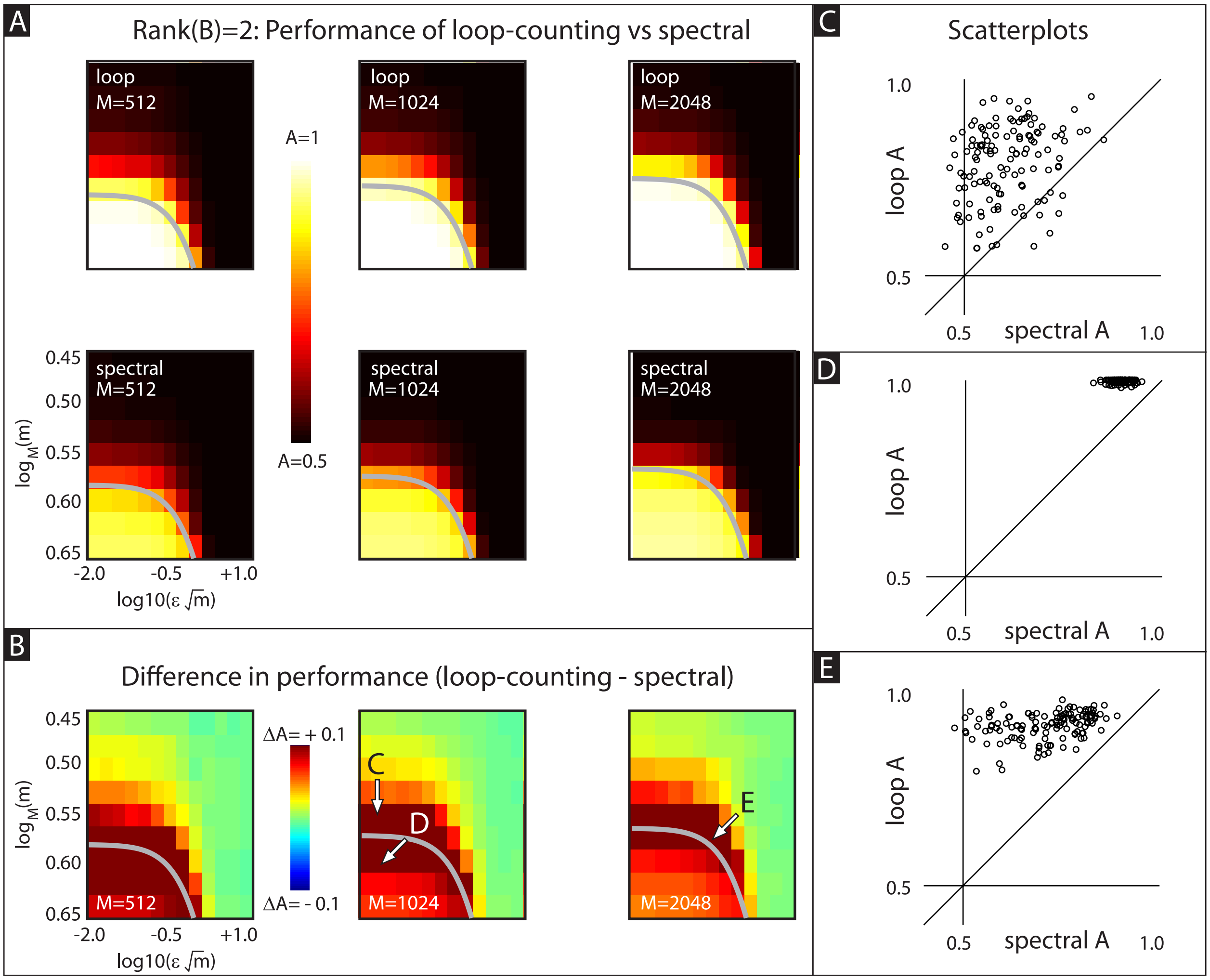

Figure 30: Performance of loop-scores vs spectral-biclustering for the rank-2 planted-bicluster problem. This figure has the same format as Fig 29. The thin grey line shows the detection-boundary for our loop-counting method (calculated using $m^3\,(2g_{2,\varepsilon,m} - 1) = \sqrt{2M^3}$).

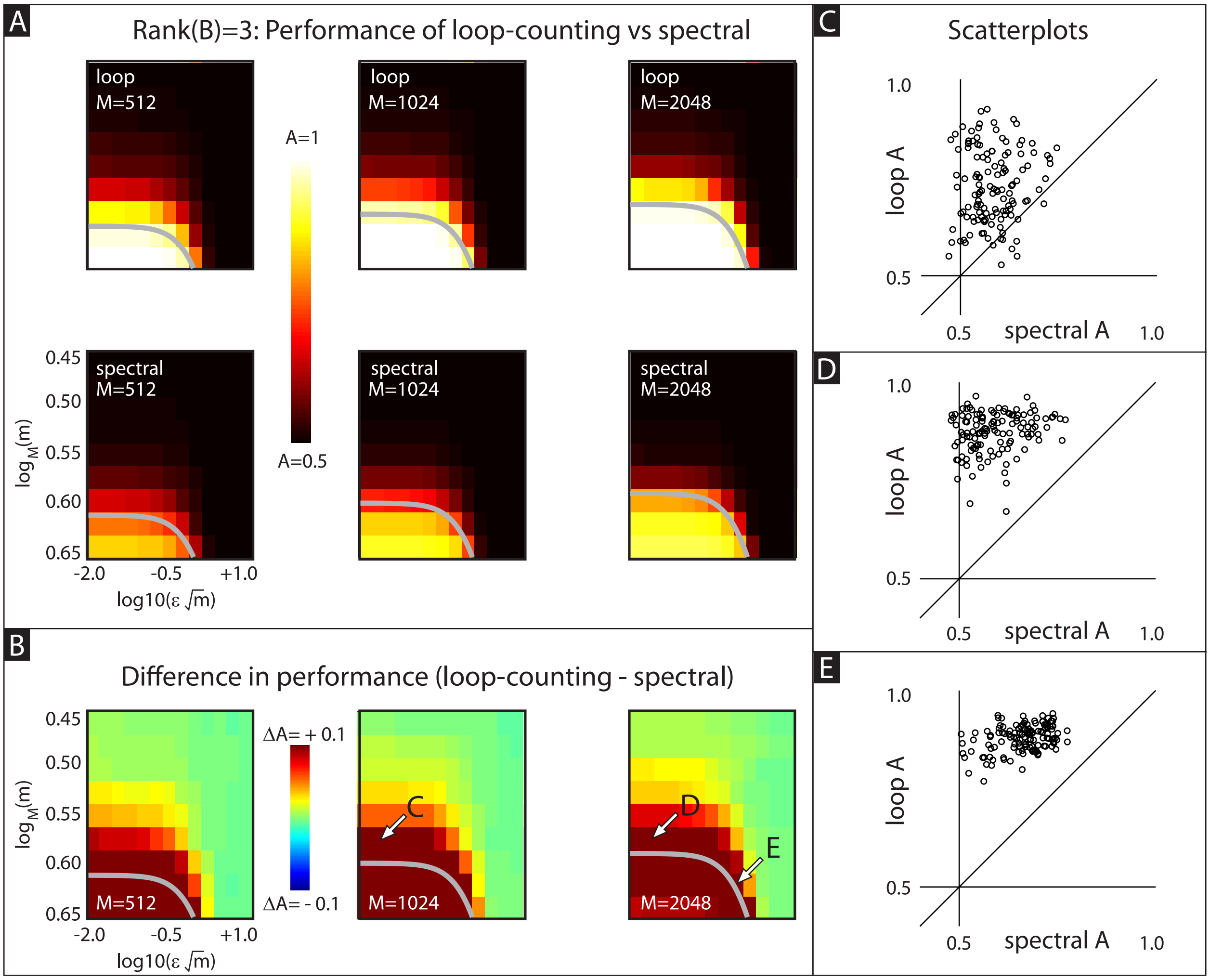

Figure 31: Performance of loop-scores vs spectral-biclustering for the rank-3 planted-bicluster problem. This figure has the same format as Fig 29. The thin grey line shows the detection-boundary for our loop-counting method (calculated using $m^3 (2g_{3,\varepsilon,m} - 1) = \sqrt{2M^3}$).

restrictions of the template above; by iteratively updating their messages in step (ii) these algorithms have no fixed c, and by adaptively choosing the nonlinearity in step (iii) they are not limited to a fixed μ. Furthermore, the framework of a message passing algorithm allows for messages to be processed 'jointly', and is not limited a simple aggregation of scores. As we discuss briefly in section 22.4, this methodology seems very promising, and will be a focus of our future work.

7.3 Why iterate?

The third major step of our algorithm is to iterate; that is, to eliminate the rows and columns with the lowest scores, recalculate the loop-scores for the remaining rows and columns, and repeat. This iteration serves two purposes.

Improved Performance: First and foremost, the iterative procedure improves the performance of our algorithm with regards to the planted-bicluster problem. While the rows and columns of the planted-bicluster may not have the highest loop-scores, they are likely not to have the lowest loop-scores. By eliminating these lowest-scoring rows and columns first, we increase the probability that the highest loop-scores in the remaining data-matrix correspond to the planted-bicluster. This iterative approach is naturally associated with a parameter 'γ', indicating the fraction of remaining rows and/or columns to eliminate at each step. Fig 32 illustrates this performance of our algorithm for various γ. Because we don't see much of a difference in our simulations for values of $\gamma \leq 0.05$, we have chosen $\gamma = 0.05$ or lower for the gene-expression and GWAS examples in this paper.

Focus on a single bicluster: In practice it is often the case that the data-matrix has multiple structures embedded within it. A single-pass algorithm can often be confused by the competing signals within the data, and may intermingle the various embedded structures. In these situations the iteration-step allows for the possibility that – as the rows and columns are eliminated – the remaining rows and columns will focus on a single embedded structure (usually the one with the strongest signal). An illustration of this phenomenon is given in Fig 33. For this example we embed two biclusters within a single data-matrix. The first bicluster $B1$ is indicated by cyan tags, the second bicluster $B2$ is indicated by magenta tags. When applying our iterative algorithm we not only detect both $B1$ and $B2$, but also separate them. A single-pass algorithm can also detect $B1$ and $B2$, but will typically intermingle the two.

We remark that the situation considered in Fig 33 is very idealized. In practice we rarely have crisp rectangular biclusters. Instead, the biclusters that appear in practice often have a soft 'core' of strongly correlated rows and columns, along with a loose collection of additional rows and columns that are less and less strongly correlated with the core. Iteration often affords an advantage in these situations as well; an iterative algorithm may be able to separate the 'cores' associated with different biclusters, focusing on the core with the strongest signal. An example illustrating this phenomenon is shown in Fig 34.

Finally, we comment that there are many different kinds of structures that can be embedded in a data-matrix, many of which can't even be described as a bicluster. To elaborate, the very concept of a bicluster assumes that there is some linear ordering of rows (and another linear ordering of columns) which reveals the bicluster. That is, a row- and column-list such that rows and columns higher up on the list 'participate' more strongly in the bicluster than rows and columns farther down on the list. There are many kinds of structures which cannot be represented with a single set of lists; one such example is shown in Fig 35.

7.4 Comparison with some other biclustering methods

As demonstrated via numerical experiments in Figs 29, 30, 31, our loop-counting method compares rather favorably to a simple spectral biclustering method when applied to the planted-bicluster problem described in Fig 24.

In this section we perform analogous numerical experiments, allowing us to compare our loop-counting method to several publicly available implementations of other biclustering methods. The methods we consider include 'BiMax', 'Cheng-and-Church', 'Plaid', 'Quest', 'Spectral' and 'Xmotif', as implemented in the 'biclust' package in R[5], with references in [9, 10, 11, 12] and [$\mathcal{M}$27] and [$\mathcal{M}$40]. We also consider the 'QuBic' and 'UniBic' algorithms [13, 14], as implemented by the authors [6]. We also consider the 'BicMix' algorithm [$\mathcal{M}$30], as implemented by the authors [7].

It is important to note that comparing these various biclustering methods is not entirely straightforward; by and large, each of these methods was designed to solve a different kind of biclustering problem. To briefly summarize, some of these other methods try and group the rows/columns of the data-matrix into disjoint (or overlapping) biclusters, others try and find large biclusters containing mostly 'large' values, others try and decompose the data-matrix into various 'factors', and others try and find more subtle patterns involving gene-expression. To make matters more complicated, the performance of many of these methods can be implementation dependent, relying on internal parameters and search-strategies that might be hard-coded into the software.

[5] available at `https://r-forge.r-project.org/`

[6] see `https://github.com/maqin2001/qubic` and `https://github.com/zhenjiawang157/UniBic`

[7] see `http://beehive.cs.princeton.edu/sw/BicMix-Code-for-distribution.zip`

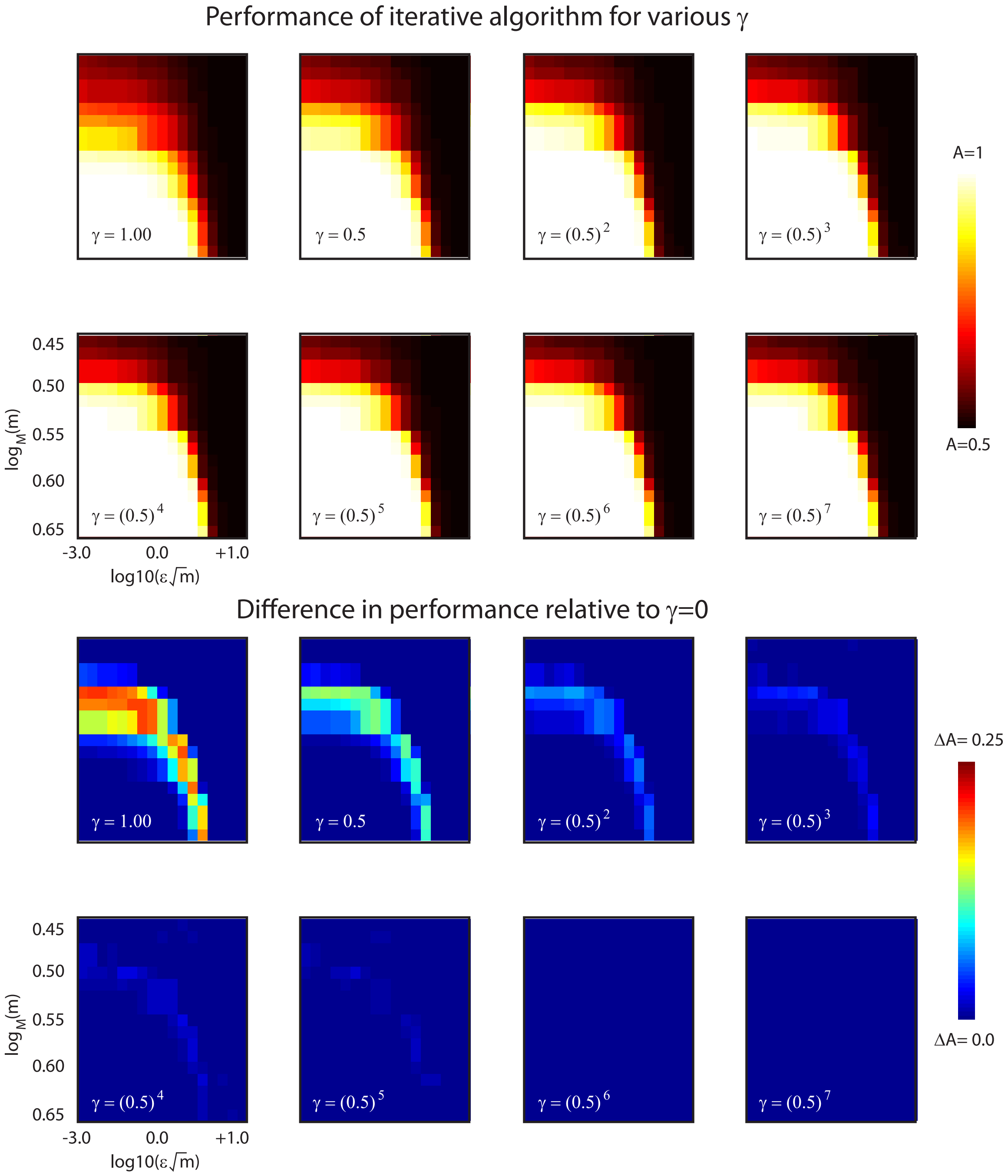

Figure 32: Performance of our algorithm on the planted-bicluster problem with $M = N = 1024$ for various choices of the iteration-parameter γ. The parameter γ is the fraction of rows and columns eliminated at each step of the algorithm. When $\gamma = 1$ the loop-scores are only calculated once and then used to order all the rows and columns. When $\gamma \to 0$ we eliminate only a single row or column with each iteration. Note that the performance of our algorithm improves as γ decreases, and for values of $\gamma \lesssim 0.5^5$ the algorithm seems to 'converge', in the sense that the performance of the algorithm approaches the $\gamma = 0$ case. In practice we notice little to no difference between our algorithm's performance when $\gamma \lesssim 5\%$.

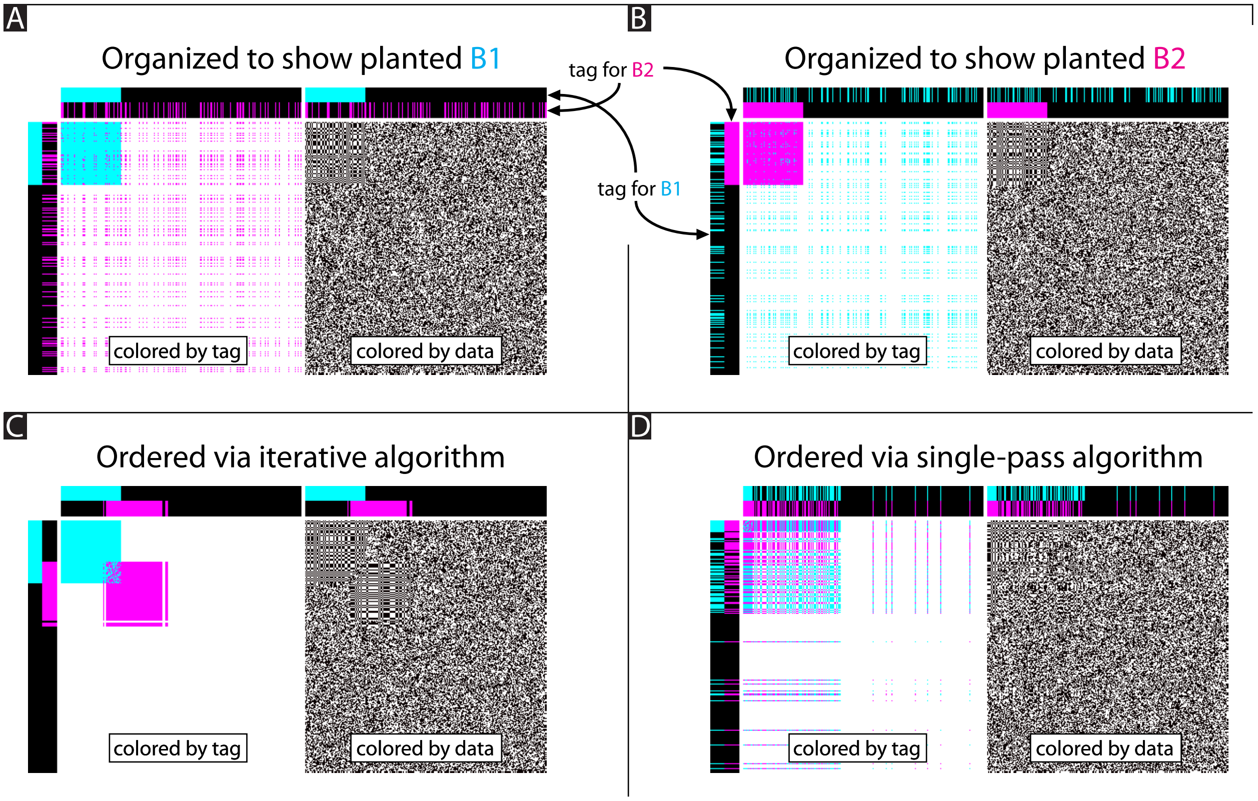

Figure 33: Demonstration of the focusing effects of an iterative algorithm. For this numerical experiment we generate a 256×256 data-matrix, within which we implant two biclusters $B1$ and $B2$. Both of these biclusters are square in shape and both are rank-1. The biclusters themselves overlap, both in terms of rows and columns. For illustration, each index (j, k) of the data-matrix is 'tagged' by $B1$ if the index is part of $B1$, and 'tagged' by $B2$ if the index is part of $B2$. We also calculate cumulative 'row-tags' for $B1$ and $B2$; these row-tags are 1 if the row or column is part of $B1$ or $B2$ respectively, and 0 otherwise. We similarly calculate column-tags for $B1$ and $B2$. In the figure shown we illustrate these tags in four different subplots. Subplots A and B organize the rows and columns of the data-matrix to reveal the planted biclusters $B1$ and $B2$, respectively. Subplots C and D organize the rows and columns based on the output of our iterative loop-score algorithm (bottom left) and the single-pass spectral-biclustering algorithm described in section 22 (bottom right). In each of the four subplots we show two copies of the data-matrix side by side; one colored by index-tag (cyan vs magenta for $B1$ vs $B2$) and the other colored by the actual data (black vs white for $+1$ vs -1). Along the sides and top of each subplot we draw vertical and horizontal strips indicating the row- and column-tags for $B1$ and $B2$. Note that, for the iterative algorithm in subplot C, rows and columns that are eliminated first show up in the bottom-right of the data-matrix, whereas the rows and columns retained the longest are placed in the upper-left. For the single-pass algorithm in subplot D, the rows and columns with the lowest scores are placed in the bottom-right, whereas those with the highest scores are placed in the upper-left. Note that the iterative algorithm not only detects both $B1$ and $B2$, but naturally separates them. This separation occurs because of the iteration; if a row or column of $B1$ is eliminated before any rows or columns of $B2$, the overall strength of the signal associated with $B1$ is reduced relative to $B2$, increasing the chance that another row or column of $B1$ will be eliminated before any rows or columns of $B2$. The same holds if a row or column of $B2$ were to be eliminated first; in this case the biclusters are symmetrically distributed, and the symmetry is broken randomly. Note also that the single-pass algorithm does not separate $B1$ and $B2$.

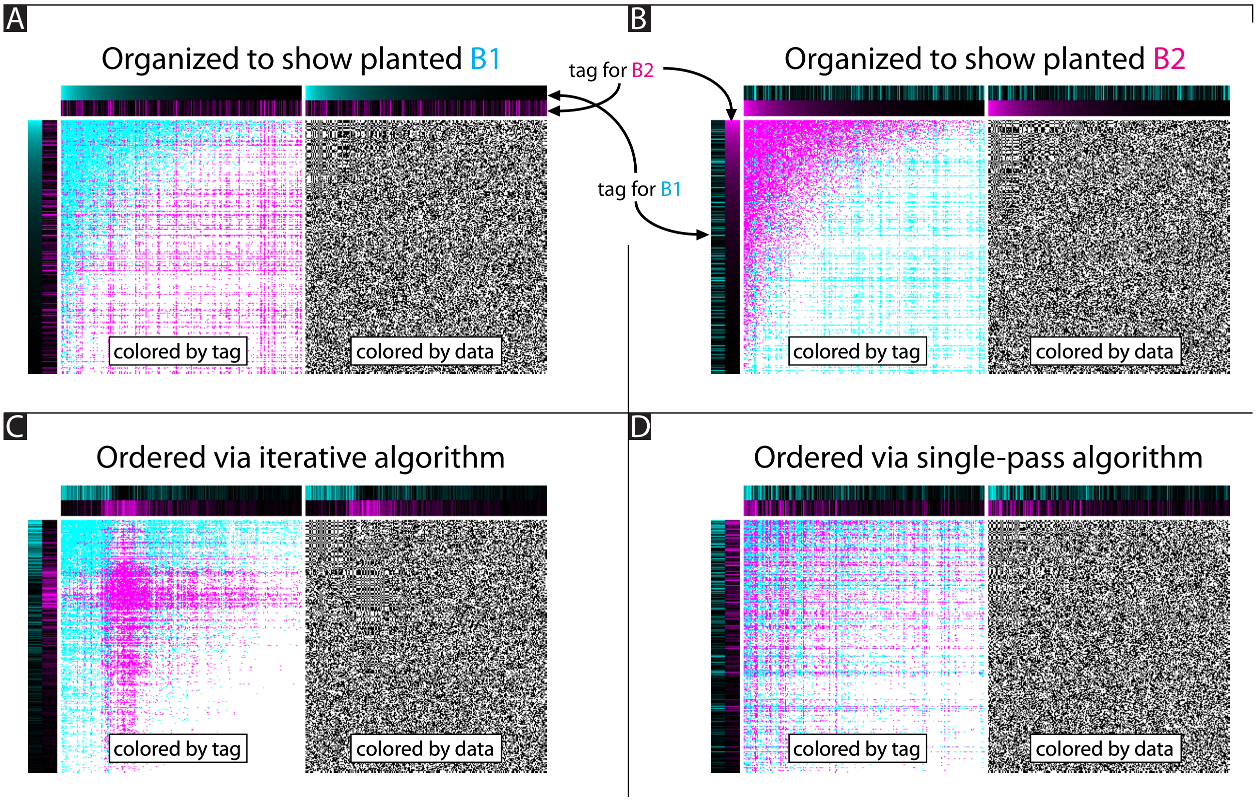

Figure 34: This figure is similar to Fig 33, except that the planted biclusters $B1$ and $B2$ are no longer perfect crisp squares. Instead, each bicluster is formed by constructing a loose collection of rows and columns, a few of which are tightly correlated with each other (forming the basis of a soft 'core'), while others are less and less correlated. In this case we calculate the row- and column-tags for $B1$ and $B2$ so that they are proportional to the number of entries within that row or column that participate in $B1$ or $B2$ respectively. As shown in subplots A and B, the biclusters are quite noisy, and the boundaries of their cores are not clearly delineated. In these kinds of situations our iterative algorithm (subplot C) can still often separate the cores of the embedded structures, whereas a single-pass algorithm (subplot D) often intermingles them.

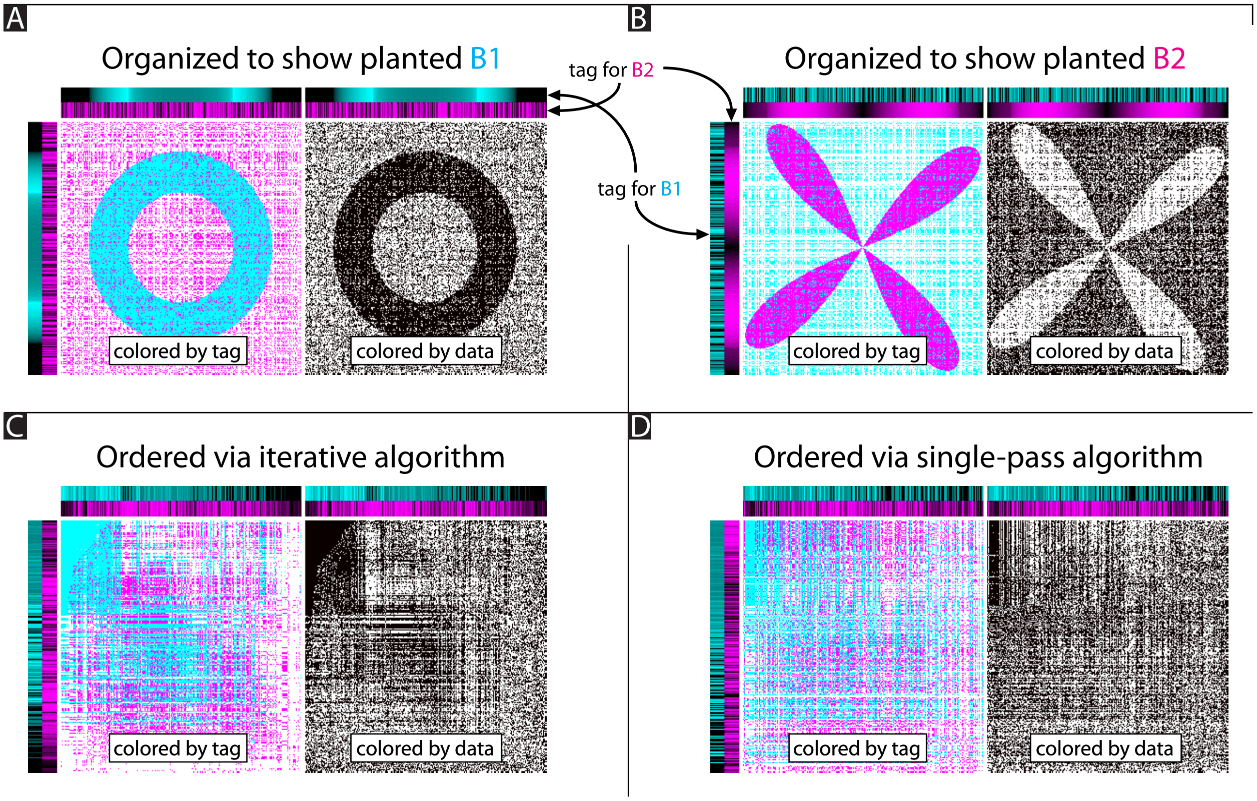

Figure 35: This figure is also similar to Fig 33, except that the planted structures $B1$ and $B2$ are no longer biclusters. These structures cannot easily be associated with a single list of row- and column-indices. Neither the iterative algorithm nor the single-pass algorithm can separate these structures from one another easily.

None of these other algorithms was designed with the same goals as our loop-counting algorithm; namely, to detect small correlated structures from within a large and noisy data-matrix. Consequently, we shouldn't expect these other algorithms to perform as well as our loop-counting algorithm when applied to the planted-bicluster problem described in section 6. We emphasize that this doesn't mean that these other methods are 'worse', or that our loop-counting method is 'better'. Indeed, the overarching goal of 'detecting structure' is so broad that no single algorithm (or implementation) will function well for every problem. There are almost surely problems out there for which these other methods outperform our loop-counting method.

Because of this discrepancy in design, we do not immediately try and directly apply these other biclustering methods to our planted-bicluster problem described in Fig 24. Instead, we first make one important change: Rather than implanting a low-rank bicluster into a random matrix D , we implant a rank-1 bicluster B that has been conditioned to have mostly positive entries (i.e., a 'rank-0' bicluster, as shown in Fig 1A of the main text). This modified 'rank-0' planted-bicluster problem is simpler than the original problem, and is more similar to some of the problems for which these other methods have been designed.

In terms of details, we start by constructing our planted-bicluster B to be numerical-rank $l = 1$ with noise ε (using the same methodology as before, described in section 5.1). Because B is close to rank-1, we can approximate $B \sim \vec{u}\vec{v}^{\intercal}$ for some vectors $\vec{u}$,$\vec{v}$. Given this approximation, we then multiply B on the left by diag(sign($\vec{u}$)), which flips the signs of each negative element of $\vec{u}$ (as well as flipping the signs of the associated entries in the other left-principal-vectors of B). We also multiply B on the right by diag(sign($\vec{v}$)), which flips the signs of each negative element of $\vec{v}$ (and also flips the signs of the associated entries in the other right-principal-vectors of B). These two operations ensure that most of the entries of B are positive, but don't change the spectrum of B. The result is a bicluster B that has the same noise-level ε as before, but is almost entirely composed of positive entries when ε is small. We then proceed as before, implanting B into D (where the entries of D are chosen from a distribution with median 0) and then binarizing the result. An illustration of the kinds of matrices this procedure generates is shown in Fig 36.

Note that our loop-counting algorithm is completely insensitive to sign-changes applied to the rows (or columns) of D; the performance of our loop-counting algorithm on this 'rank-0' planted-bicluster problem is identical to the results for the rank-1 planted-bicluster problem shown in Figs 26 and 29.

Results for the 'biclust' package:

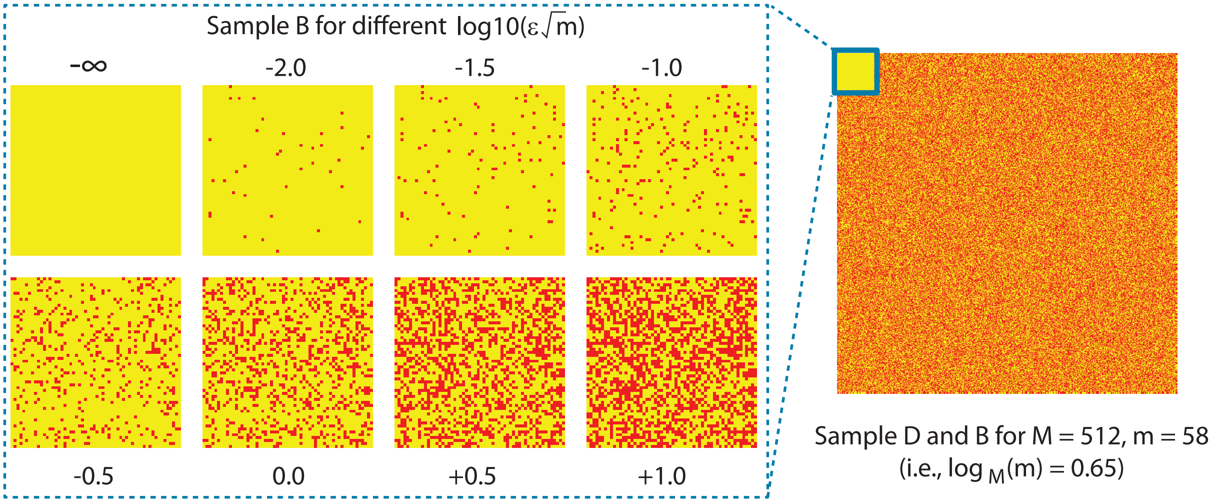

Figure 36: This figure illustrates the sample D and B matrices used for the numerical experiments shown in Fig 37. The format of this figure is similar to that of Fig 25. On the right we show a sample D of size $M = 512$, along with an implanted B of size $m = 58$, corresponding to $\log_M(m) = 0.65$. For illustration, we have rearranged the rows and columns of D to reveal B in the upper-left corner (see cyan box). This bicluster is large and noiseless; finding such a bicluster should be easy. Sample biclusters with varying noise-level are shown on the right.

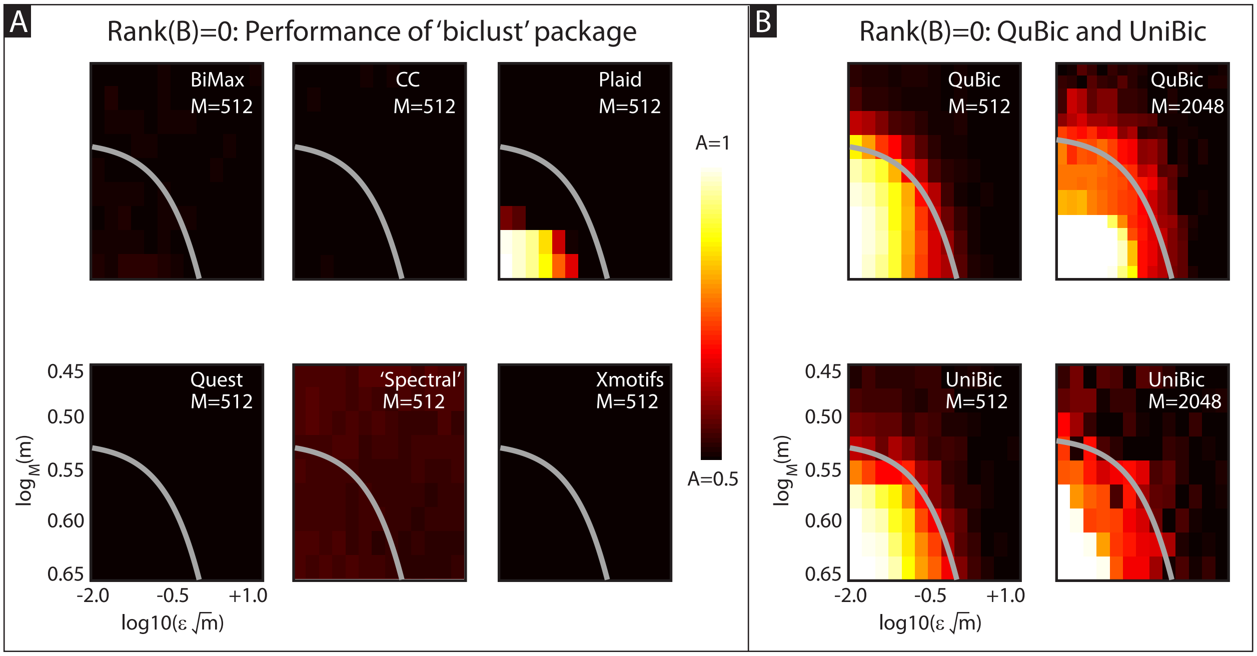

Figure 37: This figure illustrates the performance of several implementations of various biclustering algorithms (see text) on the 'rank-0' planted-bicluster problem described in section 7.4 and illustrated in Fig 36. In Panel-A we show the results associated with the 'biclust' package. The format of this figure is analogous to that of Fig 29A, with the exception that we fix $M = 512$, and we average over 64 trials for each choice of m and ε (i.e., for each pixel in the parameter-array). The thin grey line is the detection-threshold of our loop-counting algorithm (taken from Fig 26 and 29). Note that the results for the 'Spectral' method (from the biclust package) are artificially inflated (see text). In Panel-B we show the results for the 'QuBic' and 'UniBic' algorithms. For the $M = 512$ panel we average over 64 trials per pixel. Due to runtime constraints for the QuBic implementation, we only average over 8 trials per pixel when $M = 2048$. Similarly, due to runtime constraints for the UniBic implementation, we only show results for a single trial per pixel when $M = 2048$.

Shown in Fig 37A are the results for each of the publicly available algorithms listed above from the 'biclust' package; the format of this figure is analogous to that of Fig 29A, with the exception that we fix $M = 512$, and we average over 64 trials for each choice of m and ε.

In terms of details, we used the following parameter settings for each of the algorithms (with other parameters set to their default values). We tried to give each method a good chance of finding the planted-bicluster, and so we increased any available parameters associated with the number of internally generated random samples, number of iterations, etc:

BiMax: We set the 'number' parameter to 1.

Cheng and Church: We set the 'number' parameter to 1.

Plaid: We increase the 'iter.startup' parameter from the default of 5 to 200 and we increase the 'iter.layer' parameter from the default of 10 to 100.

Quest: We set the 'number' parameter to 1.

Spectral: We set the 'normalization' parameter to bistochastization (choosing the 'irrc' option instead for independent-rescaling of rows and columns did not seem to affect the performance).

Xmotifs: We increase the 'nd' parameter from the default of 10 to 100, and increase the 'sd' parameter from the default of 5 to 50. We also set the 'number' parameter to 1.

For all the algorithms aside from the 'Plaid' and 'Spectral' methods we were able to set the 'number' parameter to 1. Setting this 'number' parameter to 1 informed each method that only 1 bicluster was in the data, and asked each method to output only a single bicluster for each trial. For each trial, we used this single output-bicluster to order the rows of the original matrix D; rows within the output-bicluster were ranked higher than the others. This row-ranking then allowed us to calculate the auc A_R, as described in Fig 24. We defined A_C analogously (using the columns), and then used $A = (A_R + A_C)/2$ as a metric of success for that trial. If the output-bicluster overlapped strongly with the planted-bicluster B, then A would be high for that trial (close to 1). On the other hand, if the output-bicluster did not overlap with B, then A would be low for that trial (close to 0.5).

Even though we could not set a 'number' parameter for the 'Plaid' method, this method only ever returned a single output-bicluster, and so we calculated 'A' for the 'Plaid' method in the same fashion.

On the other hand, the implementation of the 'Spectral' method returned a varying number of biclusters for each trial, typically ranging from 10 to 25 or more. To give this 'Spectral' method the benefit of the doubt, we calculated the A value for each of the biclusters it returned on any given trial, and used the maximum as the result for that trial. As a result of this procedure, our results for the 'Spectral' method are artificially inflated (i.e., typically, one of the 20 or so output biclusters for each trial would have a small overlap with the planted-bicluster B based purely on chance).

Note that, of all the algorithms in the 'biclust' package, only the implementation of the 'Plaid' algorithm was able to reliably detect the planted-bicluster across a subset of our numerical experiments. Nevertheless, the 'biclust' implementation of the 'Plaid' algorithm does not appear to succeed all that often near the detection-threshold of the loop-counting algorithm.

Results for 'QuBic' and 'UniBic':

Shown in Fig 37B are the results for the 'QuBic' and 'UniBic' algorithms. For each implementation of the 'QuBic' and 'UniBic' algorithms we used the default values, requesting a single output bicluster (i.e., the '`-o1`' option). For the $M = 512$ panel we average over 64 trials per pixel.

Note that the implementations of these two algorithms tend to perform rather well when $M = 512$. Unfortunately, as shown in the $M = 2048$ panels to the far right, their performance appears to degrade as M increases. Due to runtime constraints for the QuBic implementation, we only average over 8 trials per pixel when $M = 2048$. Similarly, due to runtime constraints for the UniBic implementation, we only show results for a single trial per pixel when $M = 2048$.

Results for 'BicMix':

We also ran the same set of numerical experiments using the implementation of the 'BicMix' algorithm mentioned above. We used the default parameters suggested in the documentation file for this implementation (e.g., the number of factors was initialized to 50 via the '`--nf 50`' option). We were not capable of finding any biclusters using this implementation across all our numerical experiments.

Results for the rank-l planted-bicluster problem:

Shown in Figs 38 and 39 are analogous results for the rank-1 and rank-2 planted-bicluster problems. Unsurprisingly, none of these other methods perform well on these more difficult planted-bicluster problems; as alluded to above, none of these other methods were designed with the goal of finding small correlated structures from within a large and noisy data-matrix.

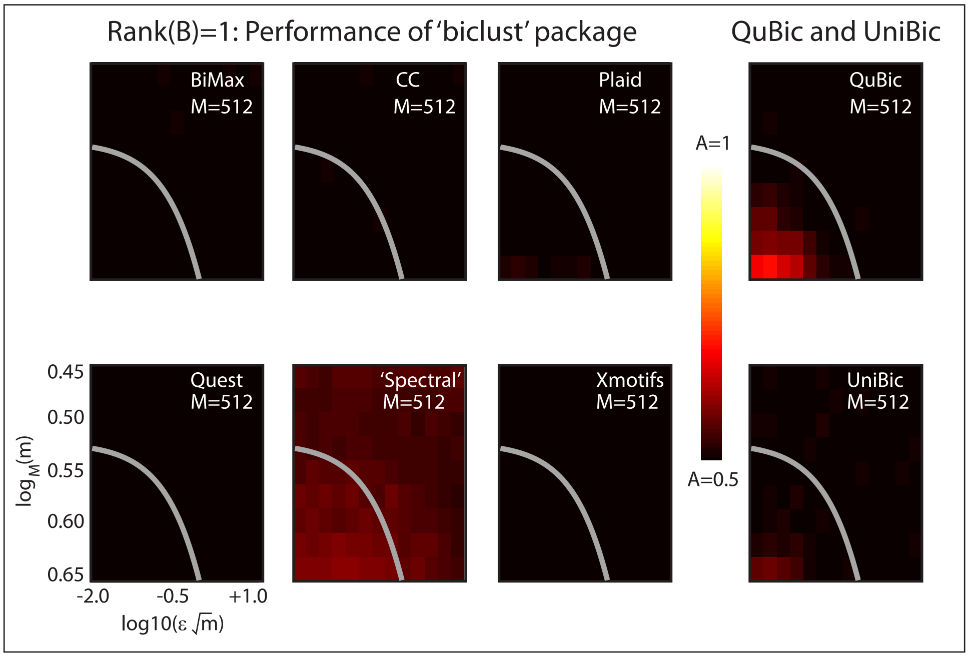

Figure 38: This figure illustrates the performance of several implementations of various biclustering algorithms on the rank-1 planted-bicluster problem. The format of this figure is analogous to that of Fig 37. The thin grey line is the detection-threshold of our loop-counting algorithm (taken from Fig 29). Note that the results for the 'Spectral' method (from the biclust package) are artificially inflated (see text).

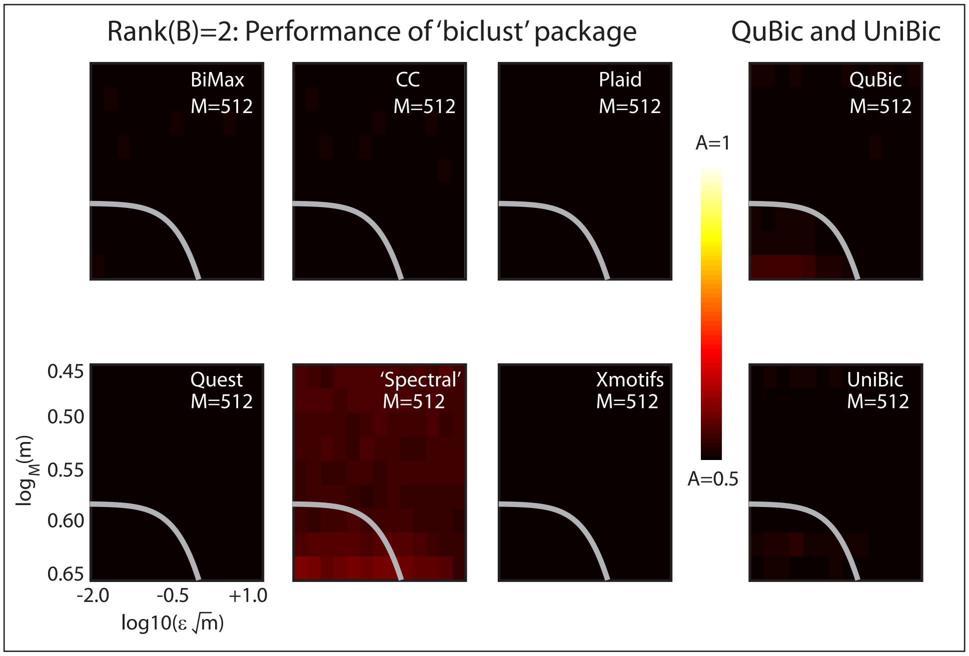

Figure 39: This figure illustrates the performance of several implementations of various biclustering algorithms on the rank-2 planted-bicluster problem. The format of this figure is analogous to that of Fig 37. The thin grey line is the detection-threshold of our loop-counting algorithm (taken from Fig 30). Note that the results for the 'Spectral' method (from the biclust package) are artificially inflated (see text).

8 Correcting for Controls: D and X

When analyzing genetic data, there often exist large subsets of genes which are correlated across large subsets of the population (e.g., genes related to development). Because these common genetic signatures correspond to massive biclusters within the data, the algorithm presented in section 2 is not typically very informative; the largest biclusters include large swathes of the patient population, and are not usually of interest. More typically, one is interested in finding biclusters which are restricted to a subset of the case-patient-population – say patients with a given disease – and which exhibit correlations which are *not* found in the control-population (e.g., patients without that disease). Such a bicluster might pinpoint genes that are linked to disease mechanisms and are useful for diagnosis.

In terms of notation, we will refer to:

- The $M_D \times N$ case-matrix D: The entry $D(j,k)$ is a real-number recording the gene-expression value for the kth-gene of the jth-case-patient.
- The $M_X \times N$ control-matrix X: The entry $X(j,k)$ is a real-number recording the gene-expression value for the kth-gene of the jth-control-patient.

Ideally, we would like to find a subset of n_B genes, as well as a subset of m_D case-patients such that the $m_D \times n_B$ bicluster lies entirely within D, and exhibits a low-rank structure which is *not* significantly exhibited within the M_X controls (i.e., the bicluster is case-specific, and does not extend into X). In a more realistic scenario, we are willing to consider a subset of n_B genes which is correlated across m_B case-patients and m_X control-patients, as long as m_D/M_D is significantly greater than m_X/M_X. In both scenarios we would like to ignore any non-specific biclusters which include a comparable fraction of cases and controls (e.g., for which $m_D/M_D = m_X/M_X$) or which are over-represented amongst the controls (e.g., $m_D/M_D < m_X/M_X$). These goals can be achieved by slightly modifying 'Step-0' and 'Step-1' of the D-only algorithm above. In Step-0 we now need to binarize both D and X. In Step-1 we need to calculate the scores in a slightly different way, which we'll explain now.

As before, we look at all loops within the data, focusing on a given row j for the moment. There will be two types of loops that involve row-j: (i) loops that are contained within D, and (ii) loops that travel through X. If row-j were part of a bicluster which was restricted to D, then we would expect row-j to participate in an abundance of rank-1 loops of type-(i), but not of type-(ii). Based on this intuition, we score the first type of loop positively if it is rank-1 and negatively if it is rank-2. In addition, we score the second type of loop the other way; negatively if it is rank-1 and positively if it is rank-2 (see Fig 40).

Collectively, this amounts to defining subscores Z_{ROW}^{DD} and Z_{ROW}^{DX}, and then combining the results to form Z_{ROW}:

$$\begin{aligned}
\left[Z_{\text{ROW}}^{DD}\right]_j &= \frac{1}{(M_D-1)\,N\,(N-1)} \sum_{\substack{j \text{ fixed},\ j'\in D,\ j'\neq j;\\ k'\neq k}} D_{jk}D_{j'k}D_{j'k'}D_{jk'}, \\
\left[Z_{\text{ROW}}^{DX}\right]_j &= \frac{1}{M_X N\,(N-1)} \sum_{\substack{j \text{ fixed},\ j'\in X;\\ k'\neq k}} D_{jk}X_{j'k}X_{j'k'}D_{jk'}, \\
\left[Z_{\text{ROW}}\right]_j &= \left[Z_{\text{ROW}}^{DD}\right]_j - \left[Z_{\text{ROW}}^{DX}\right]_j .
\end{aligned}$$

The scaling factors ensure that the collection of loops within D and the collection of loops passing through X are weighted equally (e.g., the Z_{ROW} does not change if M_X is doubled simply by duplicating each control-patient).

Note that, in the equations above, we write our sums so that each loop involves two distinct row- and column-indices (i.e., we specifically exclude collapsed-loops). We have also chosen the normalization factors to equal the number of terms in each sum (also excluding collapsed-loops). We have adopted this convention so that the row-scores $\left[Z_{\text{ROW}}^{DD}\right]_j$ and $\left[Z_{\text{ROW}}^{DX}\right]_j$ will each depend monomially on the size of each bicluster they are a part of. If we were to include collapsed-loops in our sum, then this dependence would no longer be monomial, with $\left[Z_{\text{ROW}}^{DD}\right]_j$ and $\left[Z_{\text{ROW}}^{DX}\right]_j$ each containing a small constant term. While the inclusion or exclusion of a constant term won't affect the control-correction discussed in this section, it will be convenient for the covariate-correction described later on. Thus, to maintain uniformity throughout our methodology, we exclude collapsed-loops here as well.

Because we have excluded collapsed-loops and normalized appropriately, the row-scores above have the following properties. First, when considering large random matrices the row-scores $\left[Z_{\text{ROW}}^{DD}\right]_j$ and $\left[Z_{\text{ROW}}^{DX}\right]_j$ will have mean 0 and variances $\sim 2/\left(M_D N^2\right)$ and $\sim 2/\left(M_X N^2\right)$, respectively. Now let's imagine that D and X contain a bicluster B that is a ε-error rank-l bicluster of size $(m_D + m_X) \times n_B$, with m_D rows in D (corresponding to the row-set J_B), m_X rows in X, and n_B columns from D (corresponding to the column-set K_B). The final row-score $\left[Z_{\text{ROW}}\right]_j$ is designed so that, if $j \notin J_B$ then its average row-score will be 0. However, if $j \in J_B$, then its average row-score will be $\sim (m_D/M_D - m_X/M_X)\,(n_B/N)^2\,(2g-1)$, where g is the fraction of loops in B that are rank-1. We refer to this as the

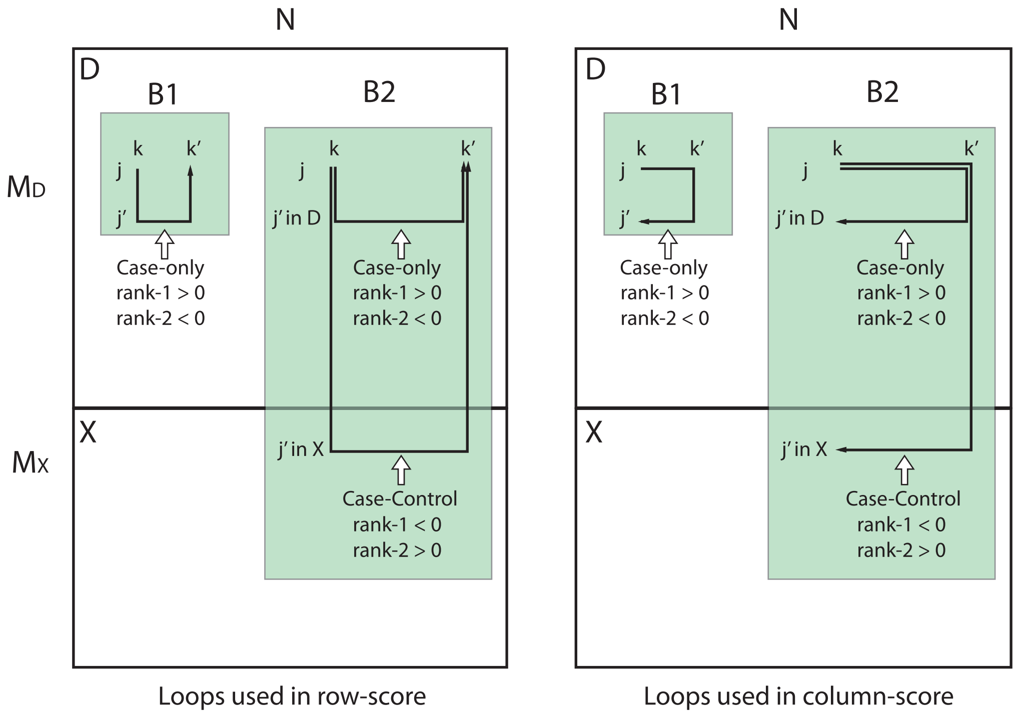

Figure 40: This figure illustrates the considerations associated with correcting for controls. In both panels we illustrate a case-matrix D (top) as well as a control-matrix X (bottom). Some loops associated with the row-scores are shown on the left panel, whereas some loops associated with the column-scores are shown on the right-panel. To aid in the discussion, we imagine that our data has within it two low-rank biclusters; $B1$, which is restricted to the cases, and $B2$, which straddles both the cases and controls. We would like to design scores that focus on case-specific biclusters such as $B1$, while ignoring non-specific biclusters such as $B2$. One way to do this is to divide the loops within our data-matrix into case-only loops and case-control loops. The former remain within D, whereas the latter extend from D to X and back again. Both biclusters $B1$ and $B2$ contain an abundance of rank-1 case-only loops (some of which are indicated in the figure). However, $B2$ also contains an abundance of rank-1 case-control loops, whereas only half the case-control loops starting from within $B1$ are rank-1. We take advantage of this observation when designing our row-score. For any given row j within D we accumulate all loops passing through that row; case-only loops contribute positively if they are rank-1, and negatively if they are rank-2; case-control loops have their sign switched, and contribute negatively if they are rank-1 and positively if they are rank-2. We adopt the same convention when designing our column-score. For any given column k within D we accumulate all loops passing through that column; case-only loops contribute positively only if they are rank-1, and case-control loops contribute positively only if they are rank-2.

scaled row-signal of B. Note that, if B is constructed as described in section 5.1, then g will equal $g_{l,\varepsilon,m_D+m_X}$. Note also that, if j participates in multiple independent and disjoint biclusters, then its average row-score will be the sum of the scaled row-signals of each of those biclusters.

The column-scores are calculated similarly:

$$\begin{aligned}
\left[Z_{\text{COL}}^{DD}\right]_k &= \frac{1}{(N-1)\,M_D\,(M_D-1)} \sum_{\substack{j',j\in D,\ j'\neq j;\\ k \text{ fixed},\ k'\neq k}} D_{jk}D_{jk'}D_{j'k'}D_{j'k},\\
\left[Z_{\text{COL}}^{DX}\right]_k &= \frac{1}{(N-1)\,M_D M_X} \sum_{\substack{j\in D,\ j'\in X;\\ k \text{ fixed},\ k'\neq k}} D_{jk}D_{jk'}X_{j'k'}X_{j'k},\\
\left[Z_{\text{COL}}\right]_k &= \left[Z_{\text{COL}}^{DD}\right]_k - \left[Z_{\text{COL}}^{DX}\right]_k.
\end{aligned}$$

These scores are designed so that the following properties hold. First, when considering large random matrices, $\left[Z_{\text{COL}}^{DD}\right]_k$ and $\left[Z_{\text{COL}}^{DX}\right]_k$ will have mean 0 and variances $\sim 2/\left(M_D^2 N\right)$ and $\sim 1/\left(M_D M_X N\right)$, respectively. Second, when considering an embedded bicluster B like the one described above (i.e., spanning column-subset K_B), the average column-score of a column $k \notin K_B$ will be 0. On the other hand, the average column-score of a column $k \in K_B$ will be equal to the scaled column-signal of B, which is $\sim (m_D/M_D)(m_D/M_D - m_X/M_X)(n_B/N)(2g-1)$. Again, If k participates in multiple independent and disjoint biclusters, then its average column-score will be the sum of the scaled column-signals of each of those biclusters.

Note that the signal produced by a case-only bicluster will be the same (on average) as the signal produced in our original D-only algorithm, but the signal produced by a non-specific bicluster (i.e., including a comparable fraction of cases and controls) will be demoted to 0. Consequently, these scores allow our algorithm to ignore any non-specific biclusters while still focusing on the biclusters which include a larger relative fraction of cases than controls. Note also that, if a row or column participates in multiple non-independent biclusters (e.g., potentially overlapping), then the overall score for that row or column will depend on the relative strengths of the various contributing signals and whether or not those signals cancel one another out. A simple example along these lines is shown in Figs 41 and 42, where we consider a variation on the planted-bicluster problem involving both a planted case-specific bicluster and a planted non-specific bicluster.

In terms of notation, it is convenient for us to introduce the 'rescaled sum' $\tilde{\Sigma}$ to denote a sum which is scaled by a factor equal to the total number of summands. With this notation our row- and column-scores become:

$$\left[Z_{\text{ROW}}^{DD}\right]_j = \widetilde{\sum_{\substack{j \text{ fixed},\ j'\in D,\ j'\neq j;\\ k'\neq k}}} D_{jk}D_{j'k}D_{j'k'}D_{jk'}, \quad \left[Z_{\text{ROW}}^{DX}\right]_j = \widetilde{\sum_{\substack{j \text{ fixed},\ j'\in X;\\ k'\neq k}}} D_{jk}X_{j'k}X_{j'k'}D_{jk'}$$

$$\left[Z_{\text{COL}}^{DD}\right]_k = \widetilde{\sum_{\substack{j',j\in D,\ j'\neq j;\\ k \text{ fixed},\ k'\neq k}}} D_{jk}D_{jk'}D_{j'k'}D_{j'k}, \quad \left[Z_{\text{COL}}^{DX}\right]_k = \widetilde{\sum_{\substack{j\in D,\ j'\in X;\\ k \text{ fixed},\ k'\neq k}}} D_{jk}D_{jk'}X_{j'k'}X_{j'k}.$$

As in the D-only case, these scores involve only a few matrix-matrix multiplications, and can be easily updated using only matrix-vector multiplications after rows or columns of D are removed. For example, when actually computing these scores one can use:

$$\begin{aligned}
\left[Z_{\text{ROW}}^{DD}\right]_j &= \frac{1}{(M_D-1)\,N\,(N-1)} \left\{[DD^{\intercal}DD^{\intercal}]_{jj} - N\,(N+M_D-1)\right\},\\
\left[Z_{\text{ROW}}^{DX}\right]_j &= \frac{1}{M_X N\,(N-1)} \left\{[DX^{\intercal}XD^{\intercal}]_{jj} - M_X N\right\}, \text{ and}
\end{aligned}$$

$$\begin{aligned}
\left[Z_{\text{COL}}^{DD}\right]_k &= \frac{1}{(N-1)\,M_D\,(M_D-1)} \left\{[D^{\intercal}DD^{\intercal}D]_{kk} - M_D\,(M_D+N-1)\right\},\\
\left[Z_{\text{COL}}^{DX}\right]_k &= \frac{1}{(N-1)\,M_D M_X} \left\{[D^{\intercal}DX^{\intercal}X]_{kk} - M_D M_X\right\}.
\end{aligned}$$

In summary, this control-correction is designed to allow our loop-counting algorithm to focus on low-rank biclusters that include a disproportionately large number of cases, while ignoring low-rank biclusters which extend across a similar fraction of cases and controls. Consequently, we expect this control-correction to be particularly useful when searching for biclusters that comprise subsets of genes that are correlated within the cases, but uncorrelated across the controls.

We conclude by pointing out that this control-correction may not always be appropriate. For example, if one is interested in searching for 'differentially-expressed' biclusters (i.e., where each gene is either high across the cases and

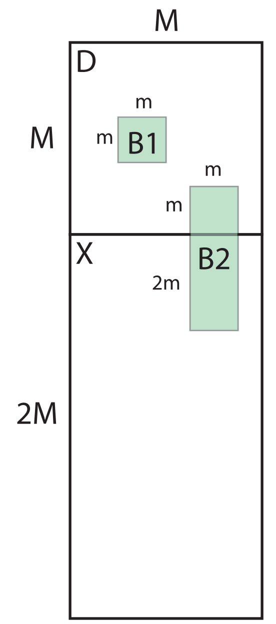

Figure 41: This figure illustrates the setup for the numerical experiments shown in Fig 42. These numerical experiments illustrate the performance of our algorithm on a simple planted-bicluster problem involving both case- and control-patients (i.e., D as well as X). For each numerical experiment we let D be a large $M \times M$ random matrix with entries chosen independently from a distribution with median 0. We construct X in a similar fashion, except that X is $2M \times M$ (to simulate the fact that real data sets often have more controls than cases). For each experiment we implant within the case-matrix D a small $m \times m$ bicluster $B1$ that is rank-1 with error ε. The rows and columns of $B1$ are chosen at random. We also implant a second rank-1 error-ε 'red-herring' bicluster $B2$ within both the case-matrix D as well as the control-matrix X. To implant $B2$ we first randomly choose m columns K and m cases J^D from D, as well as $2m$ controls J^X within X. We then construct a $3m \times m$ bicluster $B2$ that is rank-1 with error ε, and implant the top third of $B2$ into the chosen cases J^D and the bottom two-thirds of $B2$ into the chosen controls J^X (in each case using the chosen K columns). Our illustration is somewhat misleading, as $B1$ and $B2$ are not typically disjoint. Because the row- and column-subsets for these biclusters are chosen randomly, there is typically a small overlap between $B1$ and $B2$. For the purposes of this numerical experiment entries within this overlap are randomly assigned to one of the biclusters. For each numerical experiment we calculate the average auc A_1 and A_2 for biclusters $B1$ and $B2$, respectively. Our goal is to find the former, despite the masking effect of the latter.

low across the controls, or vice-versa), then one should not use the control-correction described in this section. This is because such a differentially-expressed structure is – by definition – a low-rank structure which extends across both the cases and the controls; the control-correction described above is deliberately designed to ignore such structures. Instead, if one wishes to search for 'differentially-expressed' biclusters, we recommend a modified (and simpler) version of our loop-counting algorithm – described below in section 15.2.

9 Correcting for Categorical Covariates:

Another important feature of real genetic data is that there are typically many important covariates to consider. For example, the different patients may be drawn from different categories (e.g., different studies performed at different locations or times). In these situations it is important to 'correct' for this categorial-covariate, and find biclusters that are in some sense independent of the covariate-category. In terms of notation, we'll separate our case- and control-matrices by category, using a post-script i. We'll refer to:

- The $M_{Di} \times N$ case-matrix Di: The entry $Di(j,k)$ is a real-number recording the gene-expression value for the kth-gene of the jth-case-patient from category-i.
- The $M_{Xi} \times N$ control-matrix Xi: The entry $Xi\,(j,k)$ is a real-number recording the gene-expression value for the kth-gene of the jth-control-patient from category-i.

For simplicity we'll discuss a situation with case-patients only, each belonging to one of $I_{\text{cat}} = 3$ covariate-categories. The number of case-patients in category $i \in \{1, 2, 3\}$ will be denoted by M_{D1}, M_{D2}, and M_{D3}, respectively. The data for the case-patients will be recorded in data-matrices $D1$, $D2$, and $D3$, respectively (i.,e., Di is $M_{Di} \times N$).

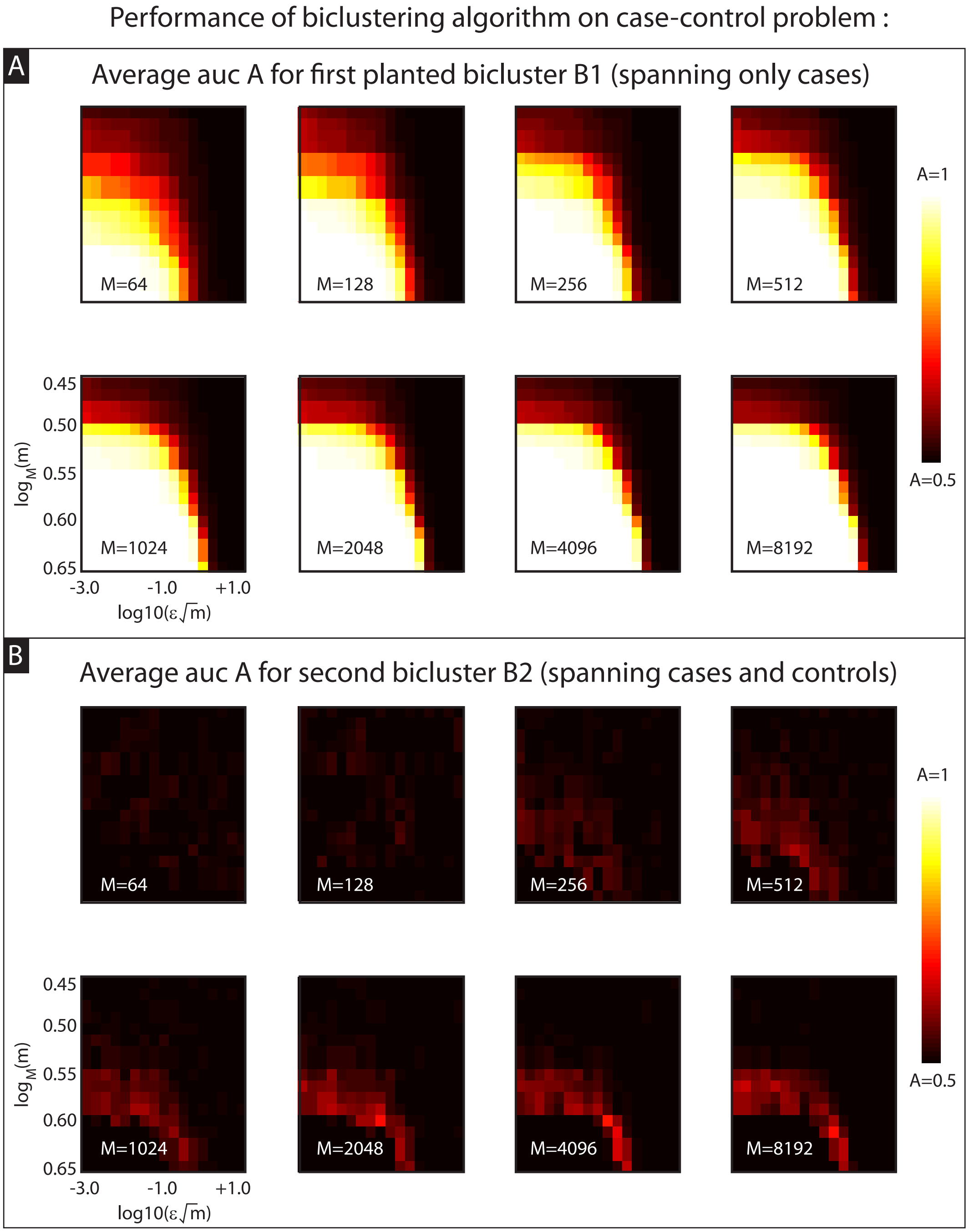

Figure 42: These numerical experiments illustrate the performance of our algorithm on a simple planted-bicluster problem involving both case- and control-patients (see Fig 41). In panel-A we show the trial-averaged value of A_1 as a function of $\varepsilon\sqrt{m}$ and $\log_M(m)$. In panel-B we show the trial-averaged value of A_2. While not quite as accurate as the D-only case (e.g., compare panel-A with Fig 26), our algorithm is still generally successful when $\log_{10}(\varepsilon\sqrt{m}) \lesssim 0$ and $\log_M(m) \gtrsim 0.5$.

Our goal when presented with this data will be to find any biclusters that extend to encompass many of the covariate-categories. In an ideal scenario, if a bicluster B involves m_{D1} patients from $D1$, m_{D2} from $D2$ and m_{D3} in $D3$, then we would like m_{D1}/M_{D1} to equal both m_{D2}/M_{D2} and m_{D3}/M_{D3} (i.e., m_{Di}/M_{Di} should be the same for all i). In a more realistic scenario we are willing to consider biclusters for which the m_{Di}/M_{Di} are different, so long as the bicluster is well represented in many of the categories. We typically want to ignore biclusters that are limited to a single category, or for which $m_{Di}/M_{Di} \sim 0$ for most of the i.

These considerations imply that there are several ways to correct for categorical-covariates, and the user needs to specify exactly what kind of correction is desired. In our case we expect a single number: an integer $I_{\text{req}} \in [1, \ldots, I_{\text{cat}}]$. The value I_{req} indicates the minimum number of covariate-categories a bicluster must encompass in order to produce a signal. In other words, $I_{\text{cat}} - I_{\text{req}}$ corresponds to the maximum number of covariate-categories a bicluster can avoid while still producing a signal. In the simple example above, a choice of $I_{\text{req}} = 2$ will allow our algorithm to ignore biclusters which are limited to only a single covariate-category, while focusing on biclusters that encompass 2 or 3 of the covariate-categories.

In terms of actual calculation, we modify the score-calculation within Step-1 of our algorithm. For each pair of categories i and i' we calculate sub-scores $Z^{i'}_{\text{ROW}}$ and $Z^{ii'}_{\text{COL}}$ as follows:

$$\left[Z^{i'}_{\text{ROW}}\right]_{j \text{ from category } i} = \widetilde{\sum_{\substack{j \text{ fixed, } j' \in \text{category } i', \\ \text{ensuring that } j' \neq j \text{ if } i'=i; \\ k' \neq k}}} Di_{jk} Di'_{j'k} Di'_{j'k'} Di_{jk'}$$

$$\left[Z^{ii'}_{\text{COL}}\right]_{k} = \widetilde{\sum_{\substack{j \in \text{category } i, \\ j' \in \text{category } i', \\ \text{ensuring that } j' \neq j \text{ if } i'=i; \\ k \text{ fixed, } k' \neq k}}} Di_{jk} Di_{jk'} Di'_{j'k'} Di'_{j'k}.$$

The row sub-scores $\left[Z^{i'}_{\text{ROW}}\right]_j$ measure the signal for row-j coming from loops traveling through category-i'. The column sub-scores $\left[Z^{ii'}_{\text{COL}}\right]_k$ measure the signal for column-k which comes from loops traveling through categories i and i'.

Note that, similar to section 8, we have excluded collapsed-loops from these row and column sub-scores. While not strictly necessary, this exclusion is convenient – ensuring that the subscores for any row or column depend monomially on the size of any biclusters that row or column participates in. If we were to include the contribution of collapsed-loops, then each subscore would also include a constant term depending on the size of the categories involved.

Once we have calculated the sub-scores above, we consider the collection $\left[Z^{i'}_{\text{ROW}}\right]_j$ for each j across all I_{cat} values of i', and sort this collection in descending order. We then choose $[Z_{\text{ROW}}]_j$ to be element number I_{req} on this sorted list (i.e., the I_{req}-order-statistic). We'll represent this by the notation:

$$[Z_{\text{ROW}}]_j = \left[Z^{\#I_{\text{req}}}_{\text{ROW}}\right]_j$$

In the case of our simple example, we would have $I_{\text{cat}} = 3$ values within each such collection (e.g., $\left[Z^1_{\text{ROW}}\right]_j$, $\left[Z^2_{\text{ROW}}\right]_j$ and $\left[Z^3_{\text{ROW}}\right]_j$), and we would choose $[Z_{\text{ROW}}]_j$ to be the second largest value. We also consider the collection $\left[Z^{ii'}_{\text{COL}}\right]_k$ for each k across all $(I_{\text{cat}})^2$ values of i, i', and sort this in descending order as well. We choose $[Z_{\text{COL}}]_k$ to be element number I^2_{req} on this sorted list, i.e.,

$$[Z_{\text{COL}}]_k = \left[Z^{\#I^2_{\text{req}}}_{\text{COL}}\right]_k.$$

In terms of our simple example this collection would contain the 9 elements $\{ \left[Z^{11}_{\text{COL}}\right]_k, \left[Z^{12}_{\text{COL}}\right]_k, \ldots, \left[Z^{33}_{\text{COL}}\right]_k \}$, and we would choose $[Z_{\text{COL}}]_k$ to be the 4th-largest element in this collection.

This procedure ensures that, in the limit as $M_{Di} \gg m_{Di} \gg 1$, any bicluster which extends to encompass at least I_{req} categories will contribute a signal to the row- or column-scores, whereas a bicluster which encompasses fewer than I_{req} categories will not. To elaborate, let's imagine an ε-error rank-l bicluster B comprising components Bi involving m_{Di} patients from Di (each associated with row-set J_{Bi}) and n columns assocated with column-set K_B. Now the row sub-score $\left[Z^{i'}_{\text{ROW}}\right]_j$ for a row j in category i will, on average, be 0 if $j \notin J_{Bi}$. On the other hand, if $j \in J_{Bi}$ then the average row sub-score will be equal to the scaled row-signal coming from Bi'; namely, $\left\{ \left(m_{D_{i'}}/M_{Di'}\right) (n_B/N)^2 (2g-1) \right\}$, (with g equal to $g_{l,\varepsilon,m}$ where $m = \sum_i m_{Di}$ is the total number of rows in B).

Note that these average row sub-scores depend monomially on the ratios $m_{Di'}/M_{Di'}$ and n_B/N, scaling linearly with $m_{Di'}/M_{Di'}$. As long as $j \in J_{Bi}$, larger values of $\left[Z^{i'}_{\text{ROW}}\right]_j$ necessarily imply larger values of $m_{Di'}/M_{Di'}$ (i.e., a larger

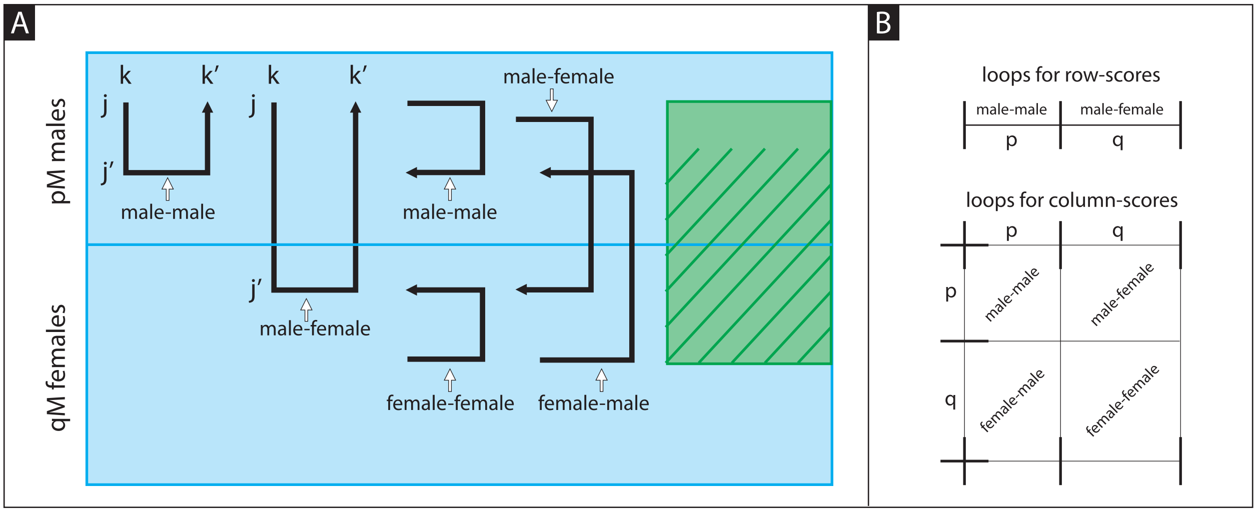

Figure 43: Correcting for a binary covariate (e.g., gender). In Panel-A we illustrate an $M \times N$ case-matrix D with pM males and qM females. Within this matrix, from left to right, we illustrate the two kinds of loops involved in the row-score for a male (row-j), and the four kinds of loops involved in the column-score for any given column. On the right side of D we illustrate an embedded bicluster B (green solid). This bicluster is not balanced across the covariates, having an abundance of males and a paucity of females (relative to the ratios p and q, respectively). The scores we devise in section 9 demote the signal produced by B to the signal that would be produced by the largest balanced bicluster within B. The size of this balanced cluster is indicated by one such representative, indicted via green hashed lines. Note that even though the signal produced by B has a strength demoted to that of its balanced component, this demoted signal affects the scores of all rows and columns of B (including those that are not included in the hashed bicluster we chose to draw). In the top of Panel-B we show the rough proportion of male-male and male-female loops expected to contribute to the row-score of any male within a balanced bicluster. In the bottom of Panel-B we show the rough proportion of male-male, male-female, female-male and female-female loops expected to contribute to the column-score of any column within a balanced bicluster.

relative-size for the bicluster within category i'). By sorting the set of $\left[Z^{i'}_{\text{ROW}}\right]_j$ (across i') and choosing the I_{req}-largest, we ensure that the final row-score for j is large only if *both* j participates in the bicluster Bi' *and* the bicluster spans at least I_{req} categories.

A similar story holds for the column sub-scores. Specifically, the column sub-score $\left[Z^{ii'}_{\text{COL}}\right]_k$ will, on average, be 0 for any column $k \notin K_B$. On the other hand, if $k \in K_B$ then the average column sub-score will be equal to the scaled column-signal involving Bi and Bi': namely, $\{(m_{Di}/M_{Di})\,(m_{Di'}/M_{Di'})\,(n_B/N)\,(2g-1)\}$. Similar to the row sub-scores, these column sub-scores depend monomially on the ratios m_{Di}/M_{Di}, $m_{Di'}/M_{Di'}$ and n_B/N, scaling linearly with each. By sorting the set of $\left[Z^{ii'}_{\text{COL}}\right]_k$ (across i, i') and choosing the I^2_{req}-largest, we ensure that the final col-score for k is large only if *both* k participates in the bicluster B *and* the bicluster spans at least I_{req} categories.

One important caveat to keep in mind when correcting for categorical-covariates is that choices of I_{req} close to I_{cat} are rather restrictive. If, for example, one were to choose $I_{\text{req}} = I_{\text{cat}}$, then our algorithm would search only for biclusters that span every single category. In this situation a bicluster will only be found if it passes the size-detection thresholds for all the categories (i.e., if $m_{Di} \gtrsim \sqrt{M_{Di}}$ for each i). This requirement is unlikely to be met – even by large biclusters – if some of the covariate-categories do not have many representatives (i.e., if M_{Di} were small for some i). Put another way, choosing $I_{\text{req}} \approx I_{\text{cat}}$ diminishes the power of our algorithm down to that of the smallest category (i.e., as if M were the smallest of the M_{Di}). Choices of I_{req} in between 1 and I_{cat} are less restrictive, and allow biclusters to be reliably detected even if some of the M_{Di} are small.

9.1 Interpretation when $I_{\text{cat}} = 2$ and $I_{\text{req}} = 2$

If we consider a binary covariate (e.g., gender), then it is natural to choose $I_{\text{req}} = 2$: a bicluster must extend acoss both covariate-categories to be considered. If we analyze this situation we see that our algorithm above can be interpreted in a slightly simpler way: each bicluster produces a signal in accordance with the largest balanced bicluster contained within it. This scenario is illustrated in Fig 43.

Carrying forward the notation used above, let's imagine that the first category corresponds to, say, males and the second to females. Let's define $M = M_{D1} + M_{D2}$ to be the total number of patients. A bicluster B involving m_{D1} males

and m_{D2} females will have $m = m_{D1} + m_{D2}$ patients in total. When considering the row-score for male-j in this bicluster, there are two possible kinds of loops which could add a signal: male-male loops and male-female loops (the former involves another row j' from the m_{D1} males in B, whereas the latter involves a row j' from the m_{D2} females in B). If the bicluster B were balanced across gender, then these two kinds of loops should occur with fractions p and q, respectively, where $p = (M_{D1} - 1) / (M - 1)$, and $q = M_{D2} / (M - 1)$ are the fraction of other males and females, respectively (note that we are excluding row-j from the males). However, if B is not balanced then one type of loop will be more abundant than expected, and the other type of loop will be less abundant. In this situation it is natural to construct a row-score which limits the signal of B to the signal produced by the largest balanced bicluster that fits within B – that is, to find out which is the lower of $(m_{D1} - 1) / (M_{D1} - 1)$ or m_{D2}/M_{D2}, and to ignore the surplus of abundant loops coming from the other gender. This can be accomplished by taking, e.g.,

$$\left[Z^1_{\text{ROW}}\right]_j = \sum_{\substack{j \text{ fixed, } j' \text{ male, } j' \neq j \\ k' \neq k}} D1_{jk} D1_{j'k} D1_{j'k'} D1_{jk'}, \text{ and } \left[Z^2_{\text{ROW}}\right]_j = \sum_{\substack{j \text{ fixed, } j' \text{ female,} \\ k' \neq k}} D1_{jk} D2_{j'k} D2_{j'k'} D1_{jk'}$$

$$\left[Z_{\text{ROW}}\right]_j = \min\left\{ \left[Z^1_{\text{ROW}}\right]_j \frac{1}{p},\ \left[Z^2_{\text{ROW}}\right]_j \frac{1}{q} \right\}.$$

Apart from scaling, this is equivalent to our row-score constructed using $I_{\text{req}} = 2$. The row-scores for any given female are similar, and involve female-female and female-male loops. Note that, while our method demotes the signal of B to that of its largest balanced component, this demoted signal affects the row-scores of all rows of B (regardless of gender).

Regarding the column-scores: for any column k there are four kinds of loops which could add a signal: male-male, male-female, female-male and female-female. Ideally, these four different kinds of loops should occur with fractions α_{11}, α_{12}, α_{21}, α_{22}, respectively, where $\alpha_{11} = M_{D1}(M_{D1} - 1) / \bar{M}^2$, $\alpha_{12} = \alpha_{21} = M_{D1} M_{D2} / \bar{M}^2$, and $\alpha_{22} = M_{D2}(M_{D2} - 1) / \bar{M}^2$, with $\bar{M}^2 = M^2 - M_{D1} - M_{D2}$. If B is not balanced we might find that the different kinds of loops will be more or less abundant than expected. We can construct a column-score which limits the signal of B to that produced by the largest balanced bicluster within B via:

$$\left[Z^{11}_{\text{COL}}\right]_k = \sum_{\substack{j', j \text{ male, } j' \neq j \\ k \text{ fixed, } k' \neq k}} D1_{jk} D1_{jk'} D1_{j'k'} D1_{j'k}, \text{ and } \left[Z^{12}_{\text{COL}}\right]_k = \sum_{\substack{j \text{ male, } j' \text{female,} \\ k \text{ fixed, } k' \neq k}} D1_{jk} D1_{jk'} D2_{j'k'} D2_{j'k}$$

$$\left[Z^{21}_{\text{COL}}\right]_k = \sum_{\substack{j \text{ female, } j' \text{male,} \\ k \text{ fixed, } k' \neq k}} D2_{jk} D2_{jk'} D1_{j'k'} D1_{j'k}, \text{ and } \left[Z^{22}_{\text{COL}}\right]_k = \sum_{\substack{j', j \text{ female, } j' \neq j \\ k \text{ fixed, } k' \neq k}} D2_{jk} D2_{jk'} D2_{j'k'} D2_{j'k}$$

$$\left[Z_{\text{COL}}\right]_k = \min\left\{ \left[Z^{11}_{\text{COL}}\right]_k \frac{1}{\alpha_{11}},\ \left[Z^{12}_{\text{COL}}\right]_k \frac{1}{\alpha_{12}},\ \left[Z^{21}_{\text{COL}}\right]_k \frac{1}{\alpha_{21}},\ \left[Z^{22}_{\text{COL}}\right]_k \frac{1}{\alpha_{22}} \right\}.$$

Again, apart from scaling, this is equivalent to the column-score we constructed above using $I_{\text{req}} = 2$. An example of this simple correction is given in Figs 44 and 45.

10 Correcting for Continuous Covariates:

For certain applications each patient is associated with a high-dimensional continuous-covariate. For example, in genome-wide association studies, each patient is often equipped with an N_T-dimensional vector of 'mds-components'. These mds-components are usually formed by applying dimension-reduction techniques (i.e., multi-dimensional-scaling) to the genetic data of the patients. The euclidean distance between patients in this N_T-dimensional mds-space is often used as a proxy for the genetic similarity of those patients' ancestors [$\mathcal{M}$37,$\mathcal{M}$38]. Consequently, when attempting to control for genetic-ancestry, we are not interested in biclusters involving patients that are concentrated together in mds-space. Instead, we would like to ignore these mds-specific biclusters and focus on the biclusters which involve patients that are widely dispersed across mds-space.

For simplicity we'll discuss a situation with case-patients only, and we'll focus on what we'll later refer to as a '2-sided' covariate correction. In terms of notation, we'll let $M = M_D$, and refer to the continuous-covariate via:

- The $M \times N_T$ case-covariate matrix T: The entry $T(j,t)$ is the tth-coefficient of the continuous-covariate-vector for the jth-case-patient.

Each row of T contains the $1 \times N_T$ vector of covariate-coefficients for that patient. In Step-0 we'll binarize both D and T. After binarization, we define the base row- and column-scores in Step-1 as though we had no covariates:

$$\left[Z^{\text{base}}_{\text{ROW}}\right]_j = \widetilde{\sum_{\substack{j \text{ fixed, } j' \in D,\ j' \neq j; \\ k' \neq k}}} D_{jk} D_{j'k} D_{j'k'} D_{jk'},$$

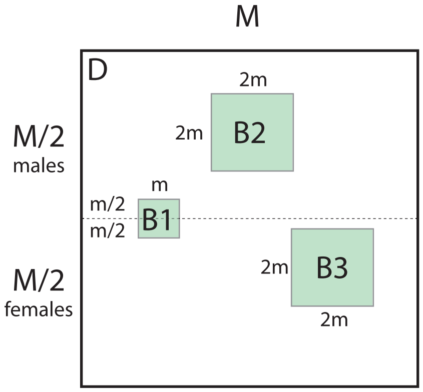

Figure 44: This figure illustrates the setup for the numerical experiments shown in Fig 45. These numerical experiments illustrate the performance of our algorithm on a simple planted-bicluster problem involving two categorical covariates (i.e. male and female). For each numerical experiment we let D be a large $M \times M$ random matrix with entries chosen independently from a distribution with median 0. Half the patients are male and half are female. For each experiment we implant a small balanced $m \times m$ bicluster $B1$ that is rank-1 with error ε and contains $m/2$ males and $m/2$ females. The rows and columns of $B1$ are chosen at random. We also randomly implant two larger rank-1 error-ε 'red-herring' biclusters $B2$ and $B3$ of size $2m \times 2m$, except that we restrict the rows of $B2$ to the males and the rows of $B3$ to the females. The illustration here is slightly misleading; the biclusters $B1, B2$ and $B3$ are not typically disjoint. Because the row- and column-subsets for each bicluster are drawn randomly, there is typically a small overlap between each pair of biclusters. For this simple numerical experiment we assign entries within any overlap to one of the overlapping biclusters at random. For each numerical experiment we calculate the average auc A_1, A_2 and A_3 for biclusters $B1$, $B2$ and $B3$, respectively. Our goal is to find the first, despite the masking effect of the latter two.

$$\left[Z^{\text{base}}_{\text{COL}}\right]_k = \widetilde{\sum_{\substack{j',j\in D,\ j'\neq j;\\ k \text{ fixed},\ k'\neq k}}} D_{jk}D_{jk'}D_{j'k'}D_{j'k}.$$

We then define additional subscores for each covariate coefficient:

$$\left[Z^{[t]}_{\text{ROW}}\right]_j = \widetilde{\sum_{\substack{j \text{ fixed},\ j'\in D,\ j'\neq j;\\ k'\neq k}}} D_{jk}D_{j'k}T_{j't}D_{j'k'}D_{jk'}T_{jt}, \text{ for each } t \in \{1,\ldots . N_T\},$$

$$\left[Z^{[t]}_{\text{COL}}\right]_k = \widetilde{\sum_{\substack{j',j\in D,\ j'\neq j;\\ k \text{ fixed},\ k'\neq k}}} D_{jk}T_{jt}D_{jk'}D_{j'k'}T_{j't}D_{j'k}, \text{ for each } t \in \{1,\ldots . N_T\}.$$

Note that, as in sections 8 and 9, we exclude collapsed-loops for these subscores. As before, this exclusion is convenient – ensuring that the subscores have a mean of 0 and depend monomially on the size of any biclusters they participate in.

We then combine these subscores to form the row- and column-scores as follows:

$$\left[Z^{[T]}_{\text{ROW}}\right]^2_j = \frac{1}{\kappa^2}\widetilde{\sum_t}\left\lfloor Z^{[t]}_{\text{ROW}}\right\rfloor^2_j, \quad \left[Z^{[T]}_{\text{COL}}\right]_k = \frac{1}{\kappa^2}\widetilde{\sum_t}\left[Z^{[t]}_{\text{COL}}\right]_k,$$

$$\left[Z_{\text{ROW}}\right]^2_j = \left\lfloor Z^{\text{base}}_{\text{ROW}}\right\rfloor^2_j - \left[Z^{[T]}_{\text{ROW}}\right]^2_j, \quad \left[Z_{\text{COL}}\right]_k = \left[Z^{\text{base}}_{\text{COL}}\right]_k - \left[Z^{[T]}_{\text{COL}}\right]_k,$$

where $\lfloor x \rfloor = \max(0, x)$, and κ^2 is a parameter that depends on N_T (we'll define κ^2 more carefully below).

The main idea behind this construction is to ensure that (i) a bicluster B with rows drawn uniformly from covariate-space produces a signal close to the original signal it would have produced without any covariate-correction, and (ii) a bicluster B with rows drawn from only one side of covariate-space will produce no signal on average. This is accomplished through the covariate-corrected scores $[Z^{[T]}_{\text{ROW}}]^2_j$ and $[Z^{[T]}_{\text{COL}}]_k$. These covariate-corrected scores will be proportional to the norm-squared of the average continuous-covariate vector accumulated across the rows of any biclusters that intersect row-j or column-k, respectively (see Fig 46). These covariate-corrected scores will typically have a small magnitude if the biclusters within the data are balanced with respect to the continuous-covariates, and a large magnitude otherwise.

To see why this might be the case, let's imagine a situation where B is an $m \times n$ bicluster within D involving row-subset J_B and column-subset K_B, and let's imagine that the covariates associated with J_B are drawn isotropically from $\mathbb{R}^{N_T}$. With this construction B is a balanced bicluster; it should have a high score.

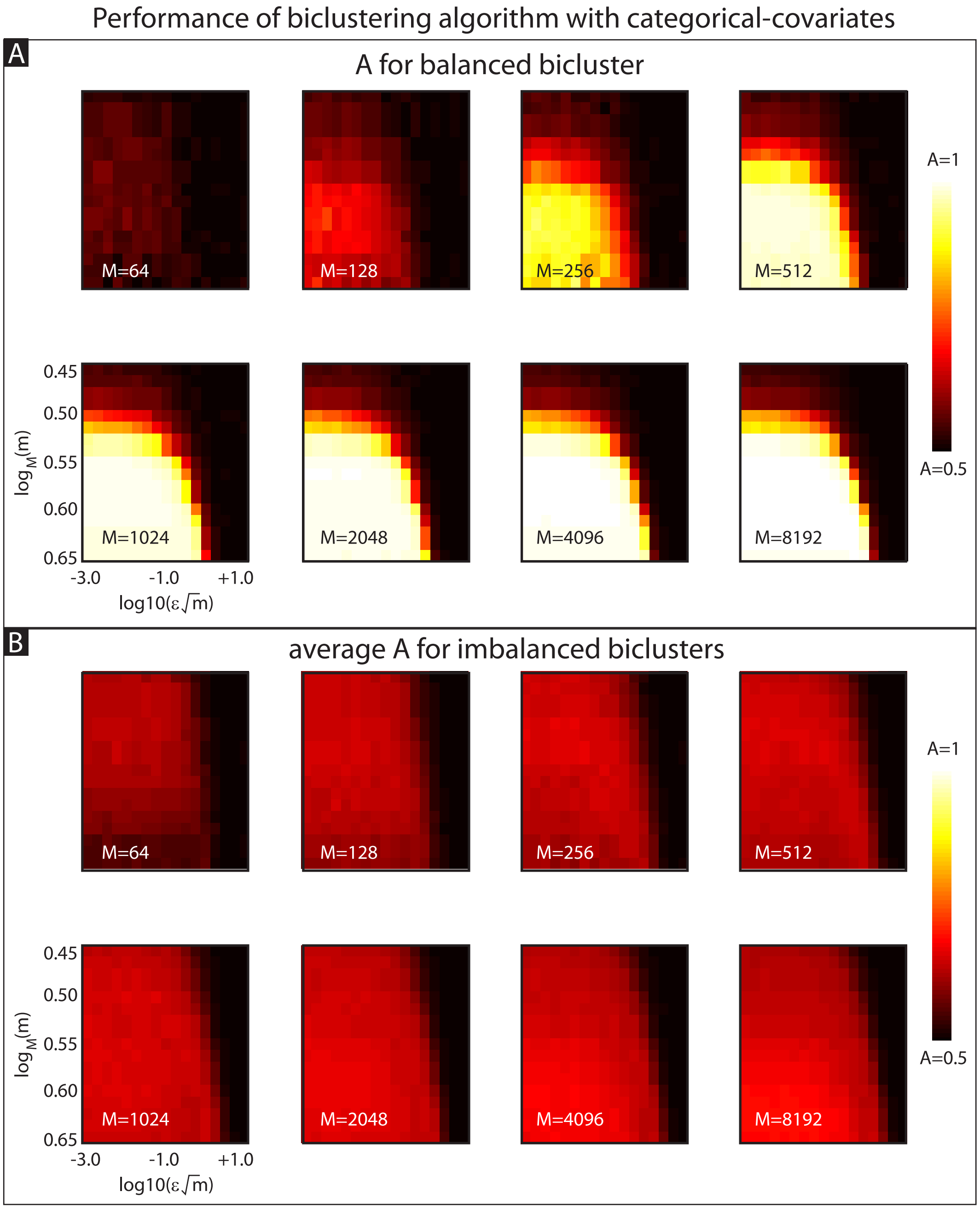

Figure 45: These numerical experiments illustrate the performance of our algorithm on a simple planted-bicluster problem involving two categorical covariates (i.e. male and female). See Fig 44. In panel-A we show the trial-averaged value of A_1 as a function of $\varepsilon\sqrt{m}$ and $\log_M(m)$. In panel-B we show the trial-averaged value of $(A_2 + A_3)/2$. While not quite as accurate as the D-only case (e.g., compare panel-A with Fig 26), our algorithm is still generally successful when $\log_{10}(\varepsilon\sqrt{m}) \lesssim 0$ and $\log_M(m) \gtrsim 0.5$, provided M is sufficiently large.

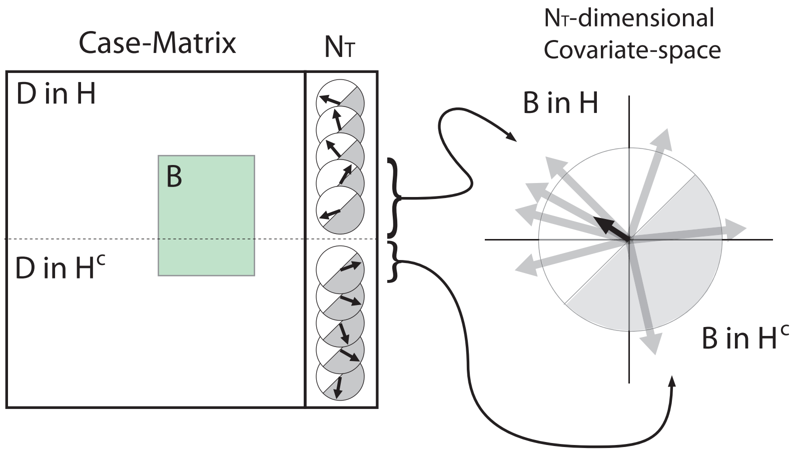

Figure 46: This figure illustrates the main idea behind our continuous-covariate correction described in section 10. For this scenario we assume that we are given a case-matrix D (shown on the left). Moreover, we'll assume that each patient (i.e., row of D) is associated with a continuous-covariate-vector of dimension $N_T = 2$. For illustration, a fraction of the rows of T are represented as unit-vectors in $\mathbb{R}^2$. For presentation purposes, we'll imagine that the continuous-covariate-space is divided into two half-spaces, H (indicated by the white semicircle) and H^c (grey semicircle) The matrix D has been organized so that the rows with continuous-covariates drawn from H are placed on top, whereas the rows with continuous-covariates drawn from H^c are placed on the bottom. Within D we've planted a bicluster B. Note that this bicluster isn't perfectly balanced with respect to the continuous-covariate; a disproportionate number of rows of B are drawn from H, with only a minority drawn from H^c. We can measure this imbalance by collecting all the continuous-covariate-vectors associated with the rows of B. A fraction of the covariate-vectors associated with B are illustrated on the right-side of the data-matrix using large grey arrows. By averaging all these covariate-vectors, we obtain the smaller dark black vector: the covariate-average. If B were balanced with respect to the continuous-covariates, we would expect this covariate-average to be close to 0 in magnitude. However, because B is imbalanced, the magnitude of the covariate-average is not quite 0. If B were more imbalanced (e.g., entirely drawn from H or entirely drawn from H^c), then the magnitude of the covariate-average would be even larger. As one may guess, the expected magnitude of this average depends both on the level of imbalance within B, as well as the (fixed) dimension N_T of covariate-space. Our covariate-correction scheme described in section 10 modifies the loop-score to account for the magnitude of this covariate-average, demoting the score of any biclusters that are imbalanced in covariate-space.

We expect the base row-score for each row-$j \in J_B$ to have a signal proportional to $(m/M)(n/N)^2(2g-1)$, where g is the fraction of rank-1 loops in B. Because the covariates associated with J_B are isotropic, the row subscores $\left[Z_{\text{ROW}}^{[t]}\right]_j$ will be roughly proportional to the vector

$$\vec{\zeta}_t = \sum_{j' \in J_B} T_{j't},$$

which has coefficients drawn from $\mathcal{N}(0, m) = \rho_{\sqrt{m}}$ (i.e., the number of positive and negative entries $T_{j't}$ should roughly cancel out). Note that for each index t,

$$\vec{\zeta}_t^2 = \sum_{j,j' \in J_B} T_{jt} T_{j't},$$

which will be drawn from $N\left(m, 2m^2\right) = m + \rho_{\sqrt{2}m}$, implying that $\left\|\vec{\zeta}\right\|^2$ will be close to a small multiple of $N_T m$. Because the value of $\left\|\vec{\zeta}\right\|^2$ is $O(N_T m)$, rather than $O\left(N_T m^2\right)$, the overall row-score for $j \in J_B$ will be on the same order as the base-row-score. Indeed, if the base-score produced by B is sufficiently large then, for $j \in J_B$, we can ignore the contribution of loops not contained within B, and we should expect:

$$\left[Z_{\text{ROW}}^{\text{base}}\right]_j^2 \sim \frac{m^2 n^4}{M^2 N^4}(2g-1)^2, \text{ and}$$

$$\left[Z_{\text{ROW}}^{[T]}\right]_j^2 \sim \frac{1}{\kappa^2} \widetilde{\sum_t} \left[\vec{\zeta}_t \cdot (1/M)(n/N)^2 (2g-1)\right]^2 \sim \frac{1}{\kappa^2} \frac{O(m) \cdot n^4}{M^2 N^4}(2g-1)^2, \text{ implying}$$

$$\left[Z_{\text{ROW}}\right]_j^2 \sim \left[Z_{\text{ROW}}^{\text{base}}\right]_j^2 \text{ for each } j \in J_B.$$

A similar story holds for the column-scores. The base column-score for each $k \in K_B$ is proportional to the signal $(m/M)^2(n/N)(2g-1)$. Because the covariates associated with B are drawn isotropically from $\mathbb{R}^{N_T}$, the column-subscores will be roughly proportional to the vector

$$\vec{\xi}_t = \sum_{j,j' \in J_B,\ j \neq j'} T_{jt} T_{j't} \sim \left(\vec{\zeta}_t\right)^2 - m,$$

which has coefficients drawn from $N\left(0, 2m^2\right) = \rho_{\sqrt{2}m}$. Note that $\sum \vec{\xi}_t \sim \left\|\vec{\zeta}^2\right\| - N_T m = O(N_T m)$, rather than $O\left(N_T m^2\right)$. Thus if the base-score produced by B is sufficiently large then, for $k \in K_B$, we can ignore the contribution of loops not contained within B, and we should expect:

$$\left[Z_{\text{COL}}^{\text{base}}\right]_k \sim \frac{m^2 n}{M^2 N}(2g-1), \text{ and}$$

$$\left[Z_{\text{COL}}^{[T]}\right]_k \sim \frac{1}{\kappa^2} \widetilde{\sum_t} \vec{\xi}_t \cdot \left(1/M^2\right)(n/N)(2g-1) \sim \frac{1}{\kappa^2} \frac{O(m) \cdot n}{M^2 N}(2g-1), \text{ again implying}$$

$$\left[Z_{\text{COL}}\right]_k \sim \left[Z_{\text{COL}}^{\text{base}}\right]_k \text{ for each } k \in K_B.$$

Now let's consider a situation where the covariates associated with B are not drawn isotropically from $\mathbb{R}^{N_T}$, but are instead drawn from a fixed (but otherwise randomly-oriented) half-space in $\mathbb{R}^{N_T}$. With this construction B will be an imbalanced bicluster; its scores should be low.

To be more precise, let us define the hemisphere in $\mathbb{R}^{N_T}$ as $H = \left\{\vec{x} \in \mathbb{R}^{N_T} \,|\, \|\vec{x}\| = 1 \text{ and } x_1 > 0\right\}$. Letting $Q \in \mathbb{R}^{N_T \times N_T}$ be a rotation of $\mathbb{R}^{N_T}$, define $\mu(Q)$ to be the uniform measure on Q. For any given Q, let T be an $m_B \times N_T$ random binary-matrix where – prior to binarization – each row is drawn independently from QH. Denote this measure on T by $\mu(T|Q)$. With this construction of T, the base row- and column-scores for B will stay the same, but the vector $\vec{\zeta}$ will be different. Because the covariates of B are now drawn from a half-space, the value $\left\|\vec{\zeta}\right\|^2$ will be $O\left(N_T m^2\right)$, rather than $O(N_T m)$. Indeed, there exists a constant of proportionality κ^2 such that the expected-value of $\left\|\vec{\zeta}\right\|^2$ will equal $\kappa^2 N_T m^2$:

$$\kappa^2(N_T, m) = \frac{1}{N_T m^2} \int \left(\int \left\|\vec{\zeta}\right\|^2 d\mu(T|Q) \right) dQ.$$

As shown in Fig 47, this constant κ^2 is only weakly dependent on m, but strongly dependent on N_T; we can approximate κ^2 via $\kappa^2(N_T) = \kappa^2(N_T, \infty)$, using the values shown in Fig 47.
