# Supplementary material for: A loop-counting method for covariate-corrected low-rank biclustering of gene-expression and genome-wide association study data
Source: PLoS Comput Biol. 2018 May 14;14(5):e1006105. doi: 10.1371/journal.pcbi.1006105 (PMC5997363; doi:10.1371/journal.pcbi.1006105)
Supplement: S2 Text — The second part of this 2-part document describes in more detail the examples shown in the main text. This document also contains proofs of certain inequalities, and a comparison with a simple spectral method. (PDF) [file pcbi.1006105.s006.pdf]

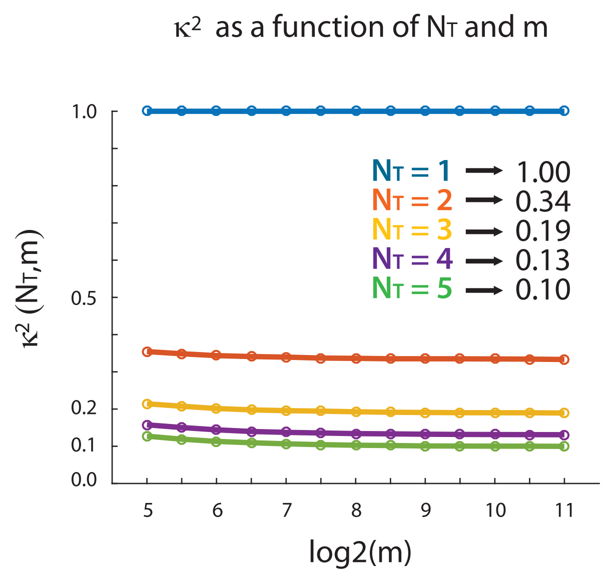

Figure 47: $\kappa^2(N_T, m)$ for the 2-sided continuous-covariate correction, as shown for $m = 32, \ldots, 2048$, with different colors corresponding to different values of N_T (higher values of N_T correspond to lower values of κ^2). The large-m values of κ^2 are listed within the legend.

Given N_T we choose $\kappa^2 = \kappa^2(N_T)$ (e.g., if there were 2 continuous-covariate-components per patient we would choose $\kappa^2 \approx 0.34$). This then implies that the sub-scores for $j \in J_B$ or $k \in K_B$ will be:

$$\left[Z_{\text{ROW}}^{[T]}\right]_j^2 \sim \frac{m^2 n^4}{M^2 N^4}(2g-1)^2, \text{ and } \left[Z_{\text{COL}}^{[T]}\right]_k \sim \frac{m^2 n}{M^2 N}(2g-1).$$

Consequently, when the covariates of B are drawn from $\mu(T|Q)$, we expect that on average with respect to $\mu(Q)$ the signal produced by B will be.

$$[Z_{\text{ROW}}]_j^2 \sim 0 \text{ and } [Z_{\text{COL}}]_k \sim 0.$$

To summarize so far: we've introduced a method for calculating row- and column-scores which corrects for multi-dimensional continuous-covariates in the following sense. A bicluster B with its covariates drawn isotropically from the covariate-space $\mathbb{R}^{N_T}$ will produce a signal comparable to what that bicluster would have produced in our original algorithm (i.e., a row-signal of $(m/M)(n/N)^2(2g-1)$ and a column-signal of $(m/M)^2(n/N)(2g-1)$). If the covariates associated with B are not isotropically distributed in $\mathbb{R}^{N_T}$, but are instead concentrated with respect to angle in $\mathbb{R}^{N_T}$, then the signal produced by B will be demoted. A bicluster B with its covariates drawn from only a half-space of $\mathbb{R}^{N_T}$ will, on average, produce a signal of 0. A bicluster B with its covariates drawn from an even more narrow cone of $\mathbb{R}^{N_T}$ will produce a negative signal. And finally, in the extreme scenario where each covariate of B is identical, the row-signal of B will be $-\left(1/\kappa^2 - 1\right)(m/M)(n/N)^2(2g-1)$ and the column-signal $-\left(1/\kappa^2 - 1\right)(m/M)^2(n/N)(2g-1)$.

In order for this kind of covariate-correction to be useful, the number of patients m_B in any planted bicluster B must be sufficiently large. Specifically, m_B must be large enough that the averages $\left[Z_{\text{ROW}}^{[T]}\right]_j^2$ and $\left[Z_{\text{COL}}^{[T]}\right]_k$ correctly reflect the covariate-distribution of B. This typically happens when $m_B \gtrsim O\left(2^{N_T}\right)$ or so. If $m_B \lesssim 2^{N_T}$ then there are unlikely to be very many patients in each orthant of $\mathbb{R}^{N_T}$; the averages $\left[Z_{\text{ROW}}^{[T]}\right]_j^2$ and $\left[Z_{\text{COL}}^{[T]}\right]_k$ are unlikely to be accurate estimates of the covariate-distribution of B, and are likely to be large even if B's covariates are drawn from an isotropic distribution. Thus, if m_B is insufficiently large, even the signals produced by covariate-balanced biclusters will be demoted, and our algorithm will not perform well. Based on this observation, we see that the m_B required for covariate-estimation grows exponentially with N_T, but is not too large for small N_T; for $N_T = 2$ the required m_B is roughly a few dozen or so (see Figs 48, 49 and 50).

Before we move on, we mention another kind of continuous-covariate correction; one that we'll refer to later as a '1-sided' correction.

$$\left[Z_{\text{ROW}}^{[t]\ 1\text{s}}\right]_j = \widetilde{\sum_{\substack{j \text{ fixed},\ j' \in D,\ j' \neq j; \\ k' \neq k}}} D_{jk} D_{j'k} T_{j't} D_{j'k'} D_{jk'}, \text{ for each } t \in \{1, \ldots N_T\},$$

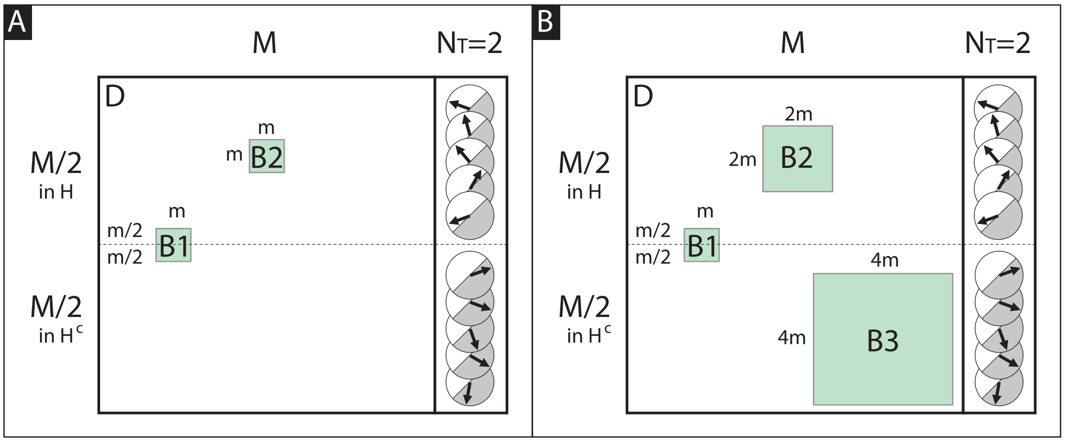

Figure 48: Panels A and B illustrate the setup for the numerical experiments shown in Figs 49 and 50, respectively. These numerical experiments illustrate the performance of our algorithm on a simple planted-bicluster problem involving continuous covariates with dimension $N_T = 2$. For each numerical experiment we let D be a large $M \times M$ random matrix with entries chosen independently from a distribution with median 0. We let T be an $M \times 2$ matrix of covariates with each row chosen randomly from an isotropic distribution on $\mathbb{R}^2$. For illustration, the rows of T are represented as unit-vectors in $\mathbb{R}^2$. Once we have chosen T, we select a randomly-oriented half-space H of $\mathbb{R}^2$ and then determine the subset J_H of rows of D which have covariates-vectors lying in H. In our illustration we depict one such randomly-oriented H with a white semicircle, and the complement H^c with a grey semicircle. We've organized the rows of D so that the rows in J_H are on top and the rows of J_{H^c} are on the bottom. In Panel-A we implant within D a small $m \times m$ bicluster $B1$ that is rank-1 with error-ε. To implant $B1$ we choose the m rows and columns at random. Note that $B1$ will typically be balanced with respect to the covariates; roughly half the rows of $B1$ will come from J_H, and half will come from J_{H^c}. We also implant within D a second rank-1 error-ε 'red-herring' bicluster $B2$ of size $m \times m$. We choose m columns at random from D, and m rows from J_H to implant $B2$. Note that bicluster $B2$ is not balanced with respect to the covariates. Note that our illustration is slightly misleading; $B1$ and $B2$ will not necessarily be disjoint. Rather, because they are randomly chosen, the row- and column-subsets of $B1$ and $B2$ will typically have a small overlap. For the purposes of this simple numerical experiment the entries in this overlap are randomly assigned to either $B1$ or $B2$. In Panel-B we implant $B1$ and $B2$ as before, except that $B2$ is of size $2m \times 2m$. We also implant a rank-1 error-ε $B3$ of size $4m \times 4m$ with rows restricted to J_{H^c}. For each numerical experiment we calculate the average auc A_1 for the bicluster $B1$, as well as the average auc A_2 for bicluster $B2$ (as well as A_3 for $B3$ if it exists). Our goal is to find the first, despite the masking effect of the latter. As shown in Fig 49, an $m \times m$ $B2$ does not compromise the effectiveness of our algorithm all that much. However, as shown in Fig 50, the presence of a larger $B2$ and $B3$ interfere with our algorithm's ability to successfully detect $B1$ when M is insufficiently large.

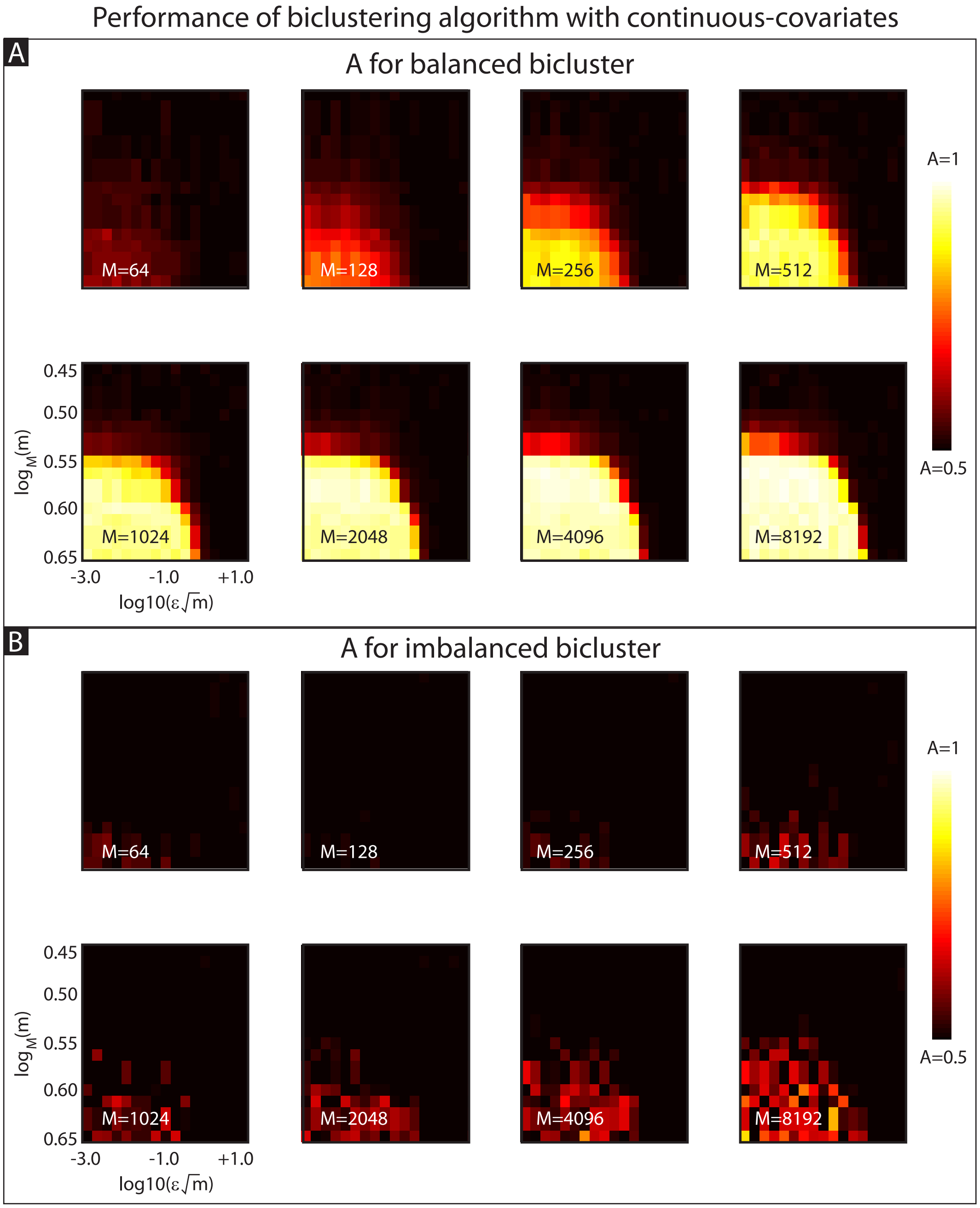

Figure 49: These numerical experiments illustrate the performance of our algorithm on a simple planted-bicluster problem involving continuous covariates with dimension $N_T = 2$ (see Fig 48A). In panel-A we show the trial-averaged value of A_1 as a function of $\varepsilon\sqrt{m}$ and $\log_M(m)$. In panel-B we show the trial-averaged value of A_2. The presence of $B2$ slightly impacts our algorithm's performance (compare with Fig 26). Note that, when M is insufficiently large (in this case $\lesssim 64$), our algorithm focuses on $B2$ rather than $B1$.

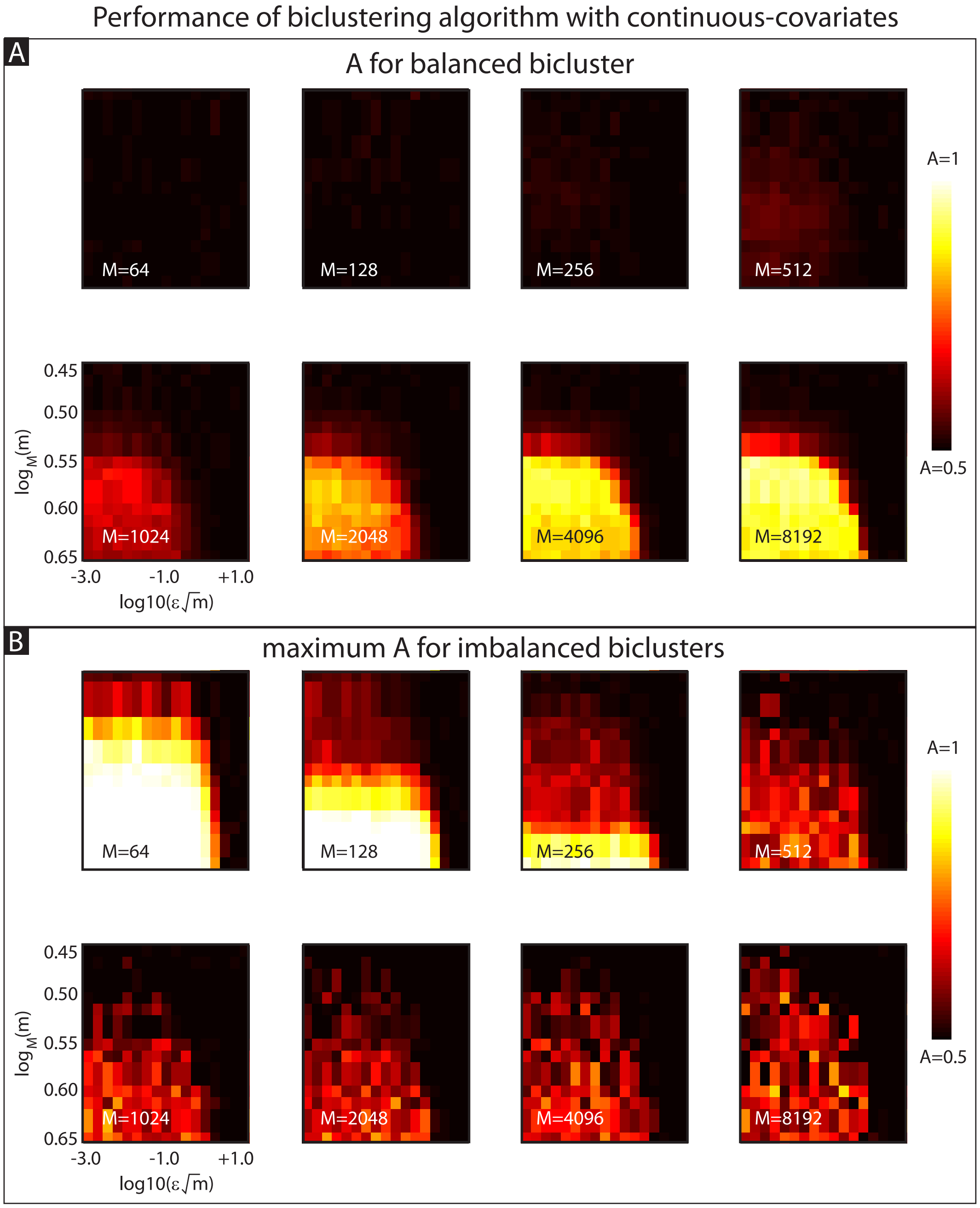

Figure 50: These numerical experiments illustrate the performance of our algorithm on a simple planted-bicluster problem involving continuous covariates with dimension $N_T = 2$ (see Fig 48B). In panel-A we show the trial-averaged value of A_1 as a function of $\varepsilon\sqrt{m}$ and $\log_M(m)$. In panel-B we show the trial-averaged value of $\max(A_2, A_3)$ which is usually A_3. The presence of $B2$ and $B3$ make it difficult for our bicluster to detect the smaller $B1$ when the M is low. Note that, when $M \lesssim 1024$, our algorithm focuses on $B2$ or $B3$ rather than $B1$.

$$\left[Z^{[t]\ 1s}_{\mathrm{COL}}\right]_k = \widetilde{\sum_{\substack{j',j\in D,\ j'\neq j;\\ k \text{ fixed},\ k'\neq k}}} D_{jk}D_{jk'}D_{j'k'}T_{j't}D_{j'k}, \text{ for each } t \in \{1,\ldots N_T\}\,.$$

This 1-sided correction only counts the continuous-covariates associated with the j' row-index, ignoring the contribution from $T_{j,t}$. The associated row- and column-scores are:

$$\left[Z^{[T]\ 1s}_{\mathrm{ROW}}\right]^2_j = \frac{1}{\kappa^2}\widetilde{\sum_t}\left[Z^{[t]\ 1s}_{\mathrm{ROW}}\right]^2_j, \quad \left[Z^{[T]\ 1s}_{\mathrm{COL}}\right]^2_k = \frac{1}{\kappa^2}\widetilde{\sum_t}\left[Z^{[t]\ 1s}_{\mathrm{COL}}\right]^2_k,$$

$$\left[Z_{\mathrm{ROW}}\right]_j = \left[Z^{\mathrm{base}}_{\mathrm{ROW}}\right]_j - \left[Z^{[T]\ 1s}_{\mathrm{ROW}}\right]_j, \quad \left[Z_{\mathrm{COL}}\right]_k = \left[Z^{\mathrm{base}}_{\mathrm{COL}}\right]_k - \left[Z^{[T]\ 1s}_{\mathrm{COL}}\right]_k.$$

The 1-sided correction is essentially the same as the 2-sided correction with regards to the row-subscores. However, the 1-sided correction is rather different for the column-subscores. Under the 1-sided correction the column-subscores only capture the norm (rather than the norm-squared) of the averaged continuous-covariate vector accumulated across biclusters that include column-k. This difference won't affect the D-only situation very much, but will have ramifications when we include the X-matrix of controls in section 12.

11 Correcting for Sparsity

Up to this point we've been assuming that the entries of the original data-matrix take on a range of values, both positive and negative. Our notion of low-rank was predicated on this assumption: Indeed, we've spent our time analyzing how our loop-scores function when the underlying data-matrix was drawn from an isotropic gaussian distribution, assuming that any planted-biclusters were drawn from an eccentric (nonisotropic) gaussian distribution. Because binarization is built into our algorithm, our analysis immediately carries forward to the situation where the original data is binary rather than continuous, provided that we assume the underlying data-matrix was drawn from a binarized version of an isotropic gaussian, and that any planted-biclusters were drawn from binarized versions of eccentric gaussians.

All of this analysis depends on one thing: our algorithm assumes that the fraction of negative entries within the data-matrix is comparable to the fraction of positive entries. If the original data-matrix has a surplus of, say, negative entries, then the binarized version of the data-matrix will be 'sparse' (i.e., the fraction of positive entries will be much less than 0.5, and the fraction of negative entries will be much greater than 0.5). Such a sparse matrix is likely to contain large submatrices consisting of mostly negative entries, simply due to their abundance. As we originally described above, our algorithm will typically focus on these large submatrices, even though they are not statistically significant. The reason for this behavior is that the abundance of negative entries gives rise to an abundance of 'all-negative loops' (i.e., rank-1 loops involving only negative entries). The loop-score we describe in section 2 treats all rank-1 loops the same way – each adds one to the total score – regardless of whether or not those loops consist of positive or negative entries (or a mixture of the two). Therefore, the large number of all-negative loops drowns out the other (more significant) rank-1 loops, obscuring the presence of any significant biclusters.

To rectify this behavior we can correct for sparsity. The main idea is to rescore the loops so that rank-1 loops consisting of entries that are otherwise abundant do not add much to the score, whereas rank-1 loops containing entries that are otherwise rare add more to the score. To describe this correction let's assume that our $M \times N$ data-matrix D is binary, with sparsity p (i.e., a fraction p of entries equal to $+1$, and a fraction $q = 1 - p$ of the entries equal to -1). We set $\alpha = p - q$ to be the column mean of each column of D, and $1/\delta = 4pq$ to be the inverse of the variance of each column. Using this notation we define:

$$[Z_{\mathrm{ROW}}]_j = \widetilde{\sum_{\substack{j \text{ fixed},\ j'\neq j;\\ k'\neq k}}} [D_{jk}-\alpha]\cdot\delta\cdot[D_{j'k}-\alpha][D_{j'k'}-\alpha]\cdot\delta\cdot[D_{jk'}-\alpha]\,,$$

$$[Z_{\mathrm{COL}}]_k = \widetilde{\sum_{\substack{j'\neq j;\\ k \text{ fixed},\ k'\neq k}}} [D_{jk}-\alpha][D_{jk'}-\alpha]\cdot\delta\cdot[D_{j'k'}-\alpha][D_{j'k}-\alpha]\cdot\delta.$$

These scores 'normalize' D by subtracting the mean from each entry and then dividing by the standard-deviation. This normalization ensures that, when considering a large random matrix in the large M, N limit, the mean row- and column-score will remain close to 0 and the variance of the row- and column-scores will remain close to $2/\left(MN^2\right)$ and $2/\left(M^2N\right)$, respectively (regardless of the fixed sparsity-coefficient p). Any rank-1 loop composed of common entries (e.g., the abundant negative entries in our earlier example) will only add a small amount to the total score, whereas a rank-1 loop composed of rare entries (e.g., positive entries) will add a larger amount to the total score.

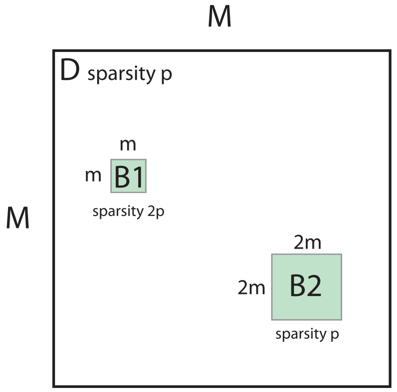

Figure 51: This figure illustrates the setup for the numerical experiments shown in Fig 52. These numerical experiments illustrate the performance of our algorithm on a simple planted-bicluster problem involving a sparse matrix with sparsity-coefficient $p = 0.0625$. For each numerical experiment we let D be a large $M \times M$ random matrix with entries chosen independently and randomly to be $+1$ with probability $p = 0.0625$, and -1 with probability $q = 1 - p$. Within D we implant an ε-error $m \times m$ bicluster $B1$ with sparsity-coefficient $2p$, and another ε-error $2m \times 2m$ bicluster $B2$ with sparsity-coefficient p. These biclusters are both approximately rank-1, and are generated using the method described in section 5.2, with m, ε and the sparsity-coefficient given as parameters. Both biclusters are implanted randomly. Note that our illustration is slightly misleading; $B1$ and $B2$ will not necessarily be disjoint. Rather, because they are randomly chosen, the row- and column-subsets of $B1$ and $B2$ will typically have a small overlap. For the purposes of this simple numerical experiment the entries in this overlap are randomly assigned to either $B1$ or $B2$. For each numerical experiment we calculate the average auc A_1 for the bicluster $B1$, as well as the average auc A_2 for bicluster $B2$. Our goal is to find both $B1$ and $B2$, even in the presence of the abundant negative entries within D. Because of the tradeoff between size, noise and sparsity, there will be combinations of m, ε and p for which $B2$ is prioritized over $B1$, and vice-versa.

To be more specific, of the 16 different possible loops within a binary-matrix, 8 will be rank-1 and 8 will be rank-2 (recall Fig 20). The 8 rank-1 loops fall into three categories: (a) four negative entries, (b) two negative and two positive entries, and (c) four positive entries. These categories contribute, respectively, p^2/q^2, 1, and q^2/p^2 to the summands above. The 8 rank-2 loops fall into two categories: (d) three negative entries and one positive entry, and (e) three positive entries and one negative entry. These categories contribute, respectively, $-p/q$ and $-q/p$ to the summands above.

To relate this to the planted-bicluster problem, let's consider an $M \times N$ data-matrix D with sparsity-coefficient $p < 0.5$. Let's implant within D a rank-1 error-ε $m \times n$ bicluster B with a potentially different sparsity-coefficient $\tilde{p}$ with $p < \tilde{p} < 0.5$. We'll generate B using the algorithm described in section 5.2. If $\varepsilon\sqrt{m}$ is not too large, the bicluster B will produce a row-sigal of approximately $(m/M)(n/N)^2\,\eta$ and a column-signal of approximately $(m/M)^2(n/N)\,\eta$, where η is given by:

$$\eta \sim g\left\{\xi_{4,0}\frac{p^2}{q^2} + \xi_{2,2} + \xi_{0,4}\frac{q^2}{p^2}\right\} - (1-g)\left\{\xi_{4,0}\frac{p}{q} + \xi_{2,2}\frac{1}{2}\left(\frac{p}{q}+\frac{q}{p}\right) + \xi_{0,4}\frac{q}{p}\right\}, \text{ with}$$

$$\xi_{4,0} = \left(\hat{p}^4 + \hat{q}^4\right),\ \xi_{2,2} = 4\left(\hat{p}^3\hat{q} + \hat{p}^2\hat{q}^2 + \hat{p}\hat{q}^3\right),\ \text{and}\ \xi_{0,4} = 2\hat{p}^2\hat{q}^2,$$

and $\hat{p}$ and $\hat{q} = 1 - \hat{p}$ given by the construction in section 5.2 (i.e., using sparsity-coefficient $\tilde{p}$). The terms $\xi_{4,0}$, $\xi_{2,2}$ and $\xi_{0,4}$ represent the distribution of loops from categories (a), (b) and (c), respectively, from within the outer product $uv^{\intercal}$ used in the construction of B. In the limit $\varepsilon\sqrt{m} \to 0$ and $p \ll 0.5$, the signal-factor η can be approximated by:

$$\eta \sim \left\{\xi_{4,0}\frac{p^2}{q^2} + \xi_{2,2} + \xi_{0,4}\frac{q^2}{p^2}\right\}, \text{ with } \xi_{4,0} \sim 1 - 2\tilde{p} + \frac{1}{2}\tilde{p}^2,\ \xi_{2,2} \sim 2\tilde{p} - \tilde{p}^2, \text{ and } \xi_{0,4} \sim \frac{1}{2}\tilde{p}^2, \text{ implying}$$

$$\eta \sim \left(1 - 2\tilde{p} + \frac{1}{2}\tilde{p}^2\right)\frac{p^2}{q^2} + 2\tilde{p} - \tilde{p}^2 + \frac{1}{2}\tilde{p}^2\frac{q^2}{p^2} \sim \frac{1}{2}\left(\frac{\tilde{p}}{p}\right)^2.$$

The overall signal produced by B depends on the size of B and the noise (as before), but also on the sparsity coefficient $\tilde{p}$ of B, which influences $\hat{p}$ and η. The signal-factor η typically grows with $\tilde{p}$ and, ultimately, there is a tradeoff: a bicluster $B1$ which is small but has a higher $\tilde{p}$ may produce a signal that is just as great as another bicluster $B2$ that is large but has a lower $\tilde{p}$. For example, the bicluster $B1$ may have more rank-1 loops containing rare positive-entries; their presence causes the loops of $B1$ to count for more than the typical loops of $B2$. An example along these lines is provided in Figs 51 and 52.

Sparsity arises in practice when attempting to analyze genotyped data; single-nucleotide-polymorphisms (SNPs) can involve minor-allele-frequencies (MAFs) that are quite small (e.g., 0.1 or smaller), giving rise to sparsity-coefficients that

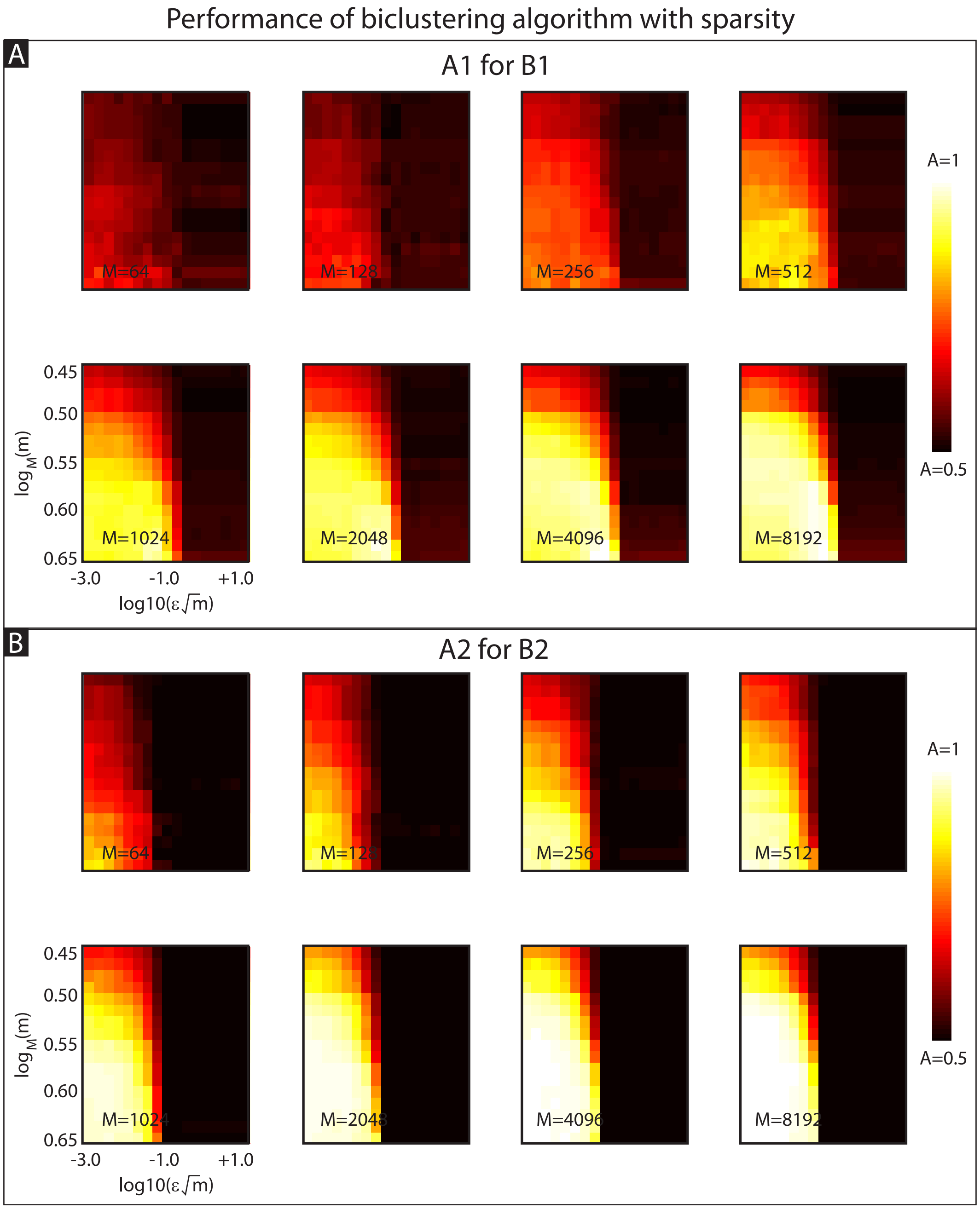

Figure 52: These numerical experiments illustrate the performance of our algorithm on a simple planted-bicluster problem involving a sparse matrix (see Fig 51). In panel-A we show the trial-averaged value of A_1 as a function of $\varepsilon\sqrt{m}$ and $\log_M(m)$. In panel-B we show the trial-averaged value of A_2. Note that our algorithm typically finds both $B1$ and $B2$, despite the abundance of negative entries in D. Depending on the parameters, either $B1$ or $B2$ can be prioritized.

are very far from 0.5 (e.g., ≤ 0.01 or ≥ 0.99). Moreover, the different columns k of the data-matrix often correspond to different SNPs with different MAFs and hence different sparsity-coefficients p_k. We can generalize our sparsity-correction to account for this scenario by defining:

$$[Z_{\text{ROW}}]_j = \widetilde{\sum_{\substack{j \text{ fixed},\, j' \neq j;\\ k' \neq k}}} [D - 1\alpha^{\intercal}]_{jk}\, \Delta_{kk}\, [D^{\intercal} - \alpha 1^{\intercal}]_{kj'}\, [D - 1\alpha^{\intercal}]_{j'k'}\, \Delta_{k'k'}\, [D^{\intercal} - \alpha 1^{\intercal}]_{k'j}\,,$$

$$[Z_{\text{COL}}]_k = \widetilde{\sum_{\substack{j' \neq j;\\ k \text{ fixed},\, k' \neq k}}} [D^{\intercal} - \alpha 1^{\intercal}]_{kj}\, [D - 1\alpha^{\intercal}]_{jk'}\, \Delta_{k'k'}\, [D^{\intercal} - \alpha 1^{\intercal}]_{k'j'}\, [D - 1\alpha^{\intercal}]_{j'k}\, \Delta_{kk},$$

where $\vec{\alpha} \in \mathbb{R}^N$ is the $N \times 1$ vector of means $\alpha_k = p_k - q_k$, $\Delta \in \mathbb{R}^{N \times N}$ is the diagonal matrix with entries $\Delta_{kk}^{-1} = 4p_k q_k$, and $\vec{1} \in \mathbb{R}^M$ is the $M \times 1$ vector of all ones. Both these row- and column-scores can be computed using two binary matrix-matrix multiplications as well as $O(1)$ matrix-vector multiplications and $O(\max(M, N))$ vector-vector dot-products (see section 12 for details).

12 Putting it all together

So far we've discussed modifications to our algorithm that account for cases-vs-controls, categorical- and continuous-covariates, as well as sparsity. One of our goals was to ensure that these modifications were not mutually exclusive; they can all be combined into one algorithm which accounts for all of the above. When combining these modifications we end up calculating a variety of sub-scores which contribute in various ways to our final row- and column-scores. Within the context of our methodology, we have chosen to perform the following steps in order:

1. Correct for sparsity.

2. Correct for continuous covariates.

3. Given I_{req}, correct for categorical covariates.

4. Correct for cases versus controls.

Based on our experience, we believe that the sparsity correction should be performed first, because the sparsity correction alters the weight of each loop. After that, the correction for continuous-covariates should come before the correction for categorical-covariates; this ensures that each category refers to its own distribution of continuous-covariates. Finally, the correction for categorical-covariates should come before the correction for cases-vs-controls; this ensures that biclusters which are balanced across case-categories are not unfairly penalized by an imbalance across control-categories. Two examples which have motivated our choices are given in Fig 53.

The main user-specified input-parameter is I_{req}, which is used in Step-3 to dictate the number of covariate-categories a bicluster must extend across in order to be considered by the algorithm. Technically speaking, Step-2 also involves a parameter κ^2, which dictates how broadly a bicluster must be distributed in continuous-covariate-space in order to be considered. While κ^2 could in principle be modified by the user, we typically set κ^2 so that, on average, biclusters produce no signal when they are drawn from only half of covariate-space (see Fig 47).

Before we describe our general procedure, we briefly review our notation. We'll consider a situation where the patients are segregated into I_{cat} different covariate-categories (e.g., study, batch-number, recording location, etc). We'll use the post-script i to track the category-number. We'll denote by M_{Di} and M_{Xi} the number of case- and control-patients in category-i. Each patient will also be associated with N different genetic-measurements. Each of these genetic-measurements will, in general, give rise to its own sparsity-coefficient p_k (for $k = 1, \ldots, N$) calculated across the entire patient population. The genetic-data for the case- and control-patients in category-i is contained in the data-matrices Di and Xi, of size $M_{Di} \times N$ and $M_{Xi} \times N$, respectively. The matrix-entry $Di\,(j, k)$ contains the kth-genetic-measurement for the jth-case-patient from category-i. The matrix-entry $Xi\,(j, k)$ contains the same for the jth-control-patient. In addition to the genetic-data, each patient will also be associated with an N_T-dimensional vector of continuous-covariates. These continuous-covariates are contained in covariate-matrices T_{Di} and T_{Xi}, of size $M_{Di} \times N_T$ and $M_{Xi} \times N_T$, respectively. The row-vector $T_{Di}\,(j, t)$ for $t = 1, \ldots, N_T$ contains the continuous-covariates for the jth case-patient in category-i. The row-vector $T_{Xi}\,(j, t)$ for $t = 1, \ldots, N_T$ contains the same for the jth-control-patient. In the expressions below we'll use the dummy-variable 'Q' below to denote either D or X; e.g., M_{Qi} could be either M_{Di} or M_{Xi}. We'll also use the notation 1_{Qi} to refer to the $M_{Qi} \times 1$ vector of all ones.

After binarizing all the matrices involved we calculate the sparsity coefficients:

$$p_k = \widetilde{\sum_{\substack{i,j\;;\\ Q = \{D, X\}}}} \lfloor Qi\,(j, k) \rfloor\,, \text{ where } \lfloor x \rfloor = \max(0, x)\,.$$

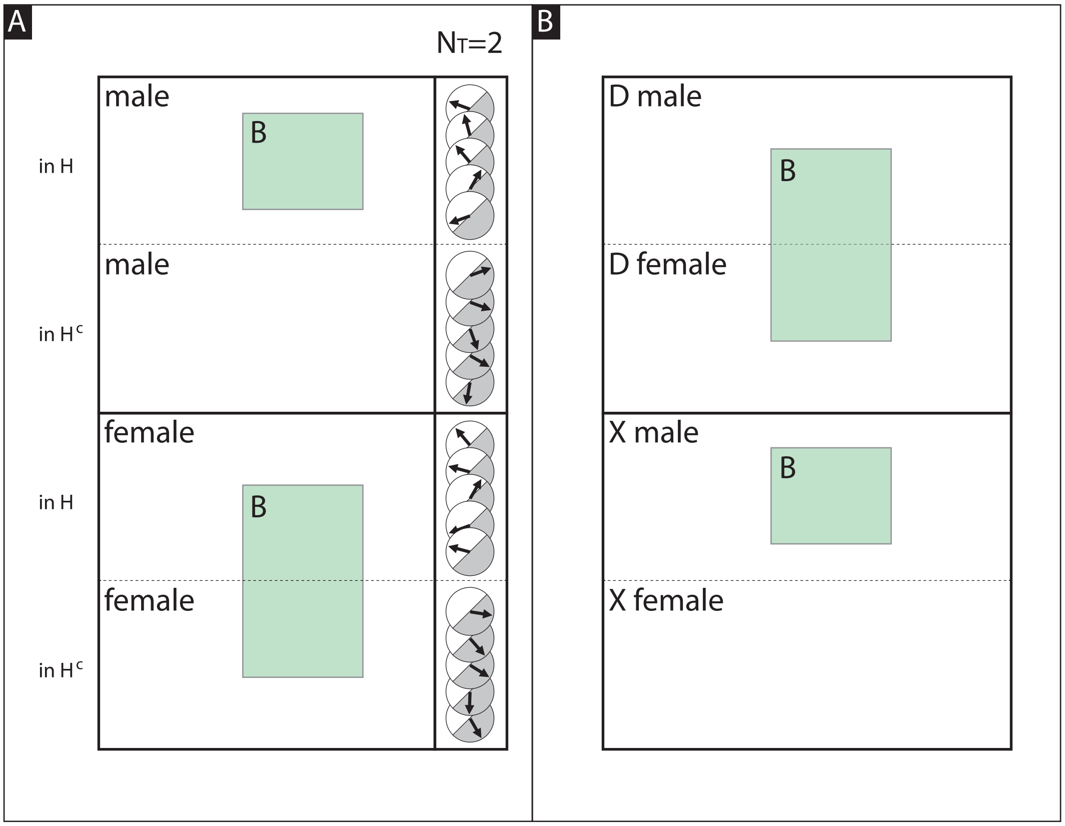

Figure 53: Examples illustrating the order of corrections for our full algorithm. In Panel-A we show a situation with both continuous- and categorical-covariates. The continuous-covariates are drawn from an $N_T = 2$ dimensional space (right side). There are two covariate-categories, male and female. The bicluster B shown extends across many males as well as many females. While the females in B have continuous-covariates drawn isotropically from $\mathbb{R}^{N_T}$, the males in B all have continuous-covariates drawn from the same half-space $H \in \mathbb{R}^{N_T}$ (indicated by the white semicircle). If we were to correct for categorical-covariates before correcting for continous-covariates, the signal produced by B would be high, which is incorrect in this case. If, instead, we correct for continuous-covariates before correcting for categorical-covariates, the signal produced by B will be correctly reduced to zero. In Panel-B we show a situation with both categorical-covariates as well as cases-vs-controls. The bicluster B extends across both male-cases and female-cases, but only across male-controls. If we were to correct for cases-vs-controls before correcting for categorical-covariates, the score for B would be reduced to zero, even though B is balanced across the cases in a manner not exhibited across the controls. If, instead, we correct for categorical-covariates before correcting for cases-vs-controls, the signal produced by B will be high, as it should be.

Using these sparsity coefficients we define the probabilities $q_k = 1 - p_k$, the means $\alpha_k = p_k - q_k$, and the variances $\delta_k^{-1} = 4p_kq_k$. These means and variances allow us to define the $N \times 1$ vector $\vec{\alpha}$ and the $N \times N$ diagonal matrix Δ with diagonal-entries δ_k. We then compute the base sub-scores for each combination of $Q = D$ and $Q' \in \{D, X\}$, for each pair of categories i, i':

$$\left[Z_{\text{ROW}}^{Q,Q';\,i,i';\,\text{base}}\right]_j = \widetilde{\sum_{\substack{Q,\ i,\ Q',\ i' \text{ fixed };\\ j \text{ fixed in } Qi,\ j' \text{ in } Q'i';\\ j'\neq j \text{ if } Q=Q' \text{ and } i=i'\\ k'\neq k}}} \left[Qi - 1_{Qi}\alpha^{\intercal}\right]_{j,k} \Delta_{kk} \left[Q'i' - 1_{Q'i'}\alpha^{\intercal}\right]_{j',k} \times \left[Q'i' - 1_{Q'i'}\alpha^{\intercal}\right]_{j',k'} \Delta_{k'k'} \left[Qi - 1_{Qi}\alpha^{\intercal}\right]_{j,k'} ,$$

$$\left[Z_{\text{COL}}^{Q,Q';\,i,i';\,\text{base}}\right]_k = \widetilde{\sum_{\substack{Q,\ i,\ Q',\ i' \text{ fixed };\\ j \text{ in } Qi,\ j' \text{ in } Q'i';\\ j'\neq j \text{ if } Q=Q' \text{ and } i=i'\\ k \text{ fixed},\ k'\neq k}}} \left[Qi - 1_{Qi}\alpha^{\intercal}\right]_{j,k} \left[Qi - 1_{Qi}\alpha^{\intercal}\right]_{j,k'} \Delta_{k'k'} \times \left[Q'i' - 1_{Q'i'}\alpha^{\intercal}\right]_{j',k'} \left[Q'i' - 1_{Q'i'}\alpha^{\intercal}\right]_{j',k} \Delta_{kk} ,$$

as well as the covariate-dependent sub-scores for each $t = 1, \ldots, N_T$:

$$\left[Z_{\text{ROW}}^{Q,Q';\,i,i';\,[t]}\right]_j = \widetilde{\sum_{\substack{Q,\ i,\ Q',\ i' \text{ fixed };\\ j \text{ fixed in } Qi,\ j' \text{ in } Q'i';\\ j'\neq j \text{ if } Q=Q' \text{ and } i=i'\\ k'\neq k}}} \left[Qi - 1_{Qi}\alpha^{\intercal}\right]_{j,k} \Delta_{kk} \left[Q'i' - 1_{Q'i'}\alpha^{\intercal}\right]_{j',k} \left[T_{Q'i'}\right]_{j',t} \times \left[Q'i' - 1_{Q'i'}\alpha^{\intercal}\right]_{j',k'} \Delta_{k'k'} \left[Qi - 1_{Qi}\alpha^{\intercal}\right]_{j,k'} \left[T_{Qi}\right]_{j,t} ,$$

$$\left[Z_{\text{COL}}^{Q,Q';\,i,i';\,[t]}\right]_k = \widetilde{\sum_{\substack{Q,\ i,\ Q',\ i' \text{ fixed };\\ j \text{ in } Qi,\ j' \text{ in } Q'i';\\ j'\neq j \text{ if } Q=Q' \text{ and } i=i'\\ k \text{ fixed},\ k'\neq k}}} \left[Qi - 1_{Qi}\alpha^{\intercal}\right]_{j,k} \left[T_{Qi}\right]_{j,t} \left[Qi - 1_{Qi}\alpha^{\intercal}\right]_{j,k'} \Delta_{k'k'} \times \left[Q'i' - 1_{Q'i'}\alpha^{\intercal}\right]_{j',k'} \left[T_{Q'i'}\right]_{j',t} \left[Q'i' - 1_{Q'i'}\alpha^{\intercal}\right]_{j',k} \Delta_{kk} .$$

Combining these sub-scores allows us to correct for continuous-covariates within each combination of Q, Q', i, i':

$$\left[Z_{\text{ROW}}^{Q,Q';\,i,i';\,[T]}\right]_j^2 = \frac{1}{\kappa^2} \widetilde{\sum_t} \left\lfloor Z_{\text{ROW}}^{Q,Q';\,i,i';\,[t]}\right\rfloor_j^2 , \text{ and } \left[Z_{\text{COL}}^{Q,Q';\,i,i';\,[T]}\right]_k = \frac{1}{\kappa^2} \widetilde{\sum_t} \left[Z_{\text{COL}}^{Q,Q';\,i,i';\,[t]}\right]_k ,$$

where $\kappa^2 = \kappa^2 (N_T)$ as defined in section 10. We then define sub-scores for each Q, Q', i, i'.

$$\left[Z_{\text{ROW}}^{Q,Q';\,i,i'}\right]_j^2 = \left\lfloor Z_{\text{ROW}}^{Q,Q';\,i,i';\,\text{base}}\right\rfloor_j^2 - \left[Z_{\text{ROW}}^{Q,Q';\,i,i';\,[T]}\right]_j^2 , \text{ and } \left[Z_{\text{COL}}^{Q,Q';\,i,i'}\right]_k = \left[Z_{\text{COL}}^{Q,Q';\,i,i';\,\text{base}}\right]_k - \left[Z_{\text{COL}}^{Q,Q';\,i,i';\,[T]}\right]_k .$$

We correct for categorical covariates by choosing the I_{req}-largest element across i' for each row-sub-score, and the I_{req}^2-largest element across i, i' for each column-sub-score (see section 9):

$$\left[Z_{\text{ROW}}^{Q,Q';\,i}\right]_j = \left[Z_{\text{ROW}}^{Q,Q';\,i,\#I_{\text{req}}}\right]_j , \text{ and } \left[Z_{\text{COL}}^{Q,Q'}\right]_k = \left[Z_{\text{COL}}^{Q,Q';\,\#I_{\text{req}}^2}\right]_k .$$

Finally, we correct for cases-vs-controls by subtracting the control-sub-scores from the case-sub-scores (see section 8):

$$\left[Z_{\text{ROW}}\right]_j = \left[Z_{\text{ROW}}^{D,D;\,i}\right]_j - \left[Z_{\text{ROW}}^{D,X;\,i}\right]_j \text{ for } j \in Di, \text{ and } \left[Z_{\text{COL}}\right]_k = \left[Z_{\text{COL}}^{D,D}\right]_k - \left[Z_{\text{COL}}^{D,X}\right]_k .$$

An example illustrating this combination of corrections is shown in Figs 54 and 55. This example illustrates the performance of our algorithm on a planted-bicluster problem involving both an $M \times N$ case-matrix D as well as a $2M \times N$ control-matrix X, where $N = 3M$. Within D and X, the case- and control-patients (i.e., rows) are randomly assigned to one of $I_{\text{cat}} = 3$ categories, with $M_{Di} = M/3$ and $M_{Xi} = 2M/3$ respectively. In addition, we endow each patient with a randomly generated continuous-covariate vector of dimension $N_T = 2$. The genes (i.e., columns) have varying degrees of sparsity; one-third of the columns have a sparsity-coefficient $p = 0.125$, one-third have $p = 0.25$, and one-third have $p = 0.5$.

Within these case- and control-matrices we implant several rank-1 ε-error biclusters, each with sparsity $p = 0.25$. The size of these implanted biclusters will depend on m and n, where n is related to m by ensuring that $\log_N(n) = \log_M(m)$ (i.e., if $m = \sqrt{M}$, then $n = \sqrt{N}$).

Case-specific and balanced: The first bicluster we implant is an $m \times n$ bicluster $B0$ restricted to the cases. The bicluster $B0$ is implanted within D by choosing m rows and columns at random; consequently, $B0$ is a case-specific bicluster that is typically balanced across all 3 covariate-categories and continuous-covariates.

Case-specific and somewhat balanced: The next three biclusters we implant, $B1$ $B2$ and $B3$, are each of size $3m/2 \times 3n/2$ and are restricted to the cases. Each of these bicluster has its rows and columns chosen at random from D, with the restriction that $B1$ excludes category-1, $B2$ excludes category-2, and $B3$ excludes category-3; each of these three case-specific biclusters is balanced with respect to the continuous-covariates, but only extends across 2 of the 3 covariate-categories.

Case-nonspecific: The next bicluster we implant, $B4$, is of size $9m/2 \times 3n/2$, and extends across both the cases and controls. This bicluster has 1/3 of its rows drawn randomly from D and 2/3 drawn from X; $B4$ will be balanced with respect to the continuous- and categorical-covariates, but will not be case-specific (extending into the controls).

Case-specific but imbalanced: The final two biclusters we implant, $B5$ and $B6$, are each of size $3m/2 \times 3n/2$, and are restricted to the cases. Each of these biclusters has its rows and columns chosen at random from D, with the restriction that $B5$ be restricted to rows that have continuous-covariates drawn from a randomly oriented half-space $H \in \mathbb{R}^{N_T}$, and $B6$ be restricted to rows that have continuous-covariates drawn from the complement H^c. Consequently, each of these two case-specific biclusters will be balanced with respect to the categorical-covariates, but not the continuous-covariates.

All of our biclusters have columns drawn randomly; each will typically overlap all of the different sparsity-domains in D and/or X. Note that the illustration in Fig 54 is misleading; the biclusters will not necessarily have disjoint columns (nor strongly overlapping rows). Rather, because they are randomly chosen, the row- and column-subsets of each bicluster will typically have a small overlap. For the purposes of this numerical experiment we implant each bicluster in the order described above: any overlapping entries will be overwritten by the next bicluster in the list (with the imbalanced biclusters taking precedence).

For each numerical experiment we calculate the average auc A_0 for the bicluster $B0$, as well as the average auc A_1 for bicluster $B1$ and so forth. In our first numerical experiment shown on the left of Fig 55 we set $I_{\text{req}} = 3$; We are looking for biclusters that span *all* of the covariate-categories, and we are not interested in finding $B1$ $B2$ or $B3$. With this choice of $I_{\text{req}} = 3$, our goal is to find $B0$ despite the masking effects of the other biclusters. In our second numerical experiment shown on the right of Fig 55 we set $I_{\text{req}} = 2$; We are looking for biclusters that span at least 2 of the 3 covariate-categories. With $I_{\text{req}} = 2$ we are satisfied with either $B0$ $B1$ $B2$ or $B3$, but we still don't want to find $B4$ $B5$ or $B6$, since these are either case-nonspecific or imbalanced with respect to the continuous-covariates.

2-sided versus 1-Sided continuous-covariate correction: As we alluded to earlier, it is possible to correct for continuous-covariates in step-2 by using a '1-sided'-correction, rather than the 2-sided correction described above. These 1-sided corrections involve altering the covariate-dependent sub-scores:

$$\left[Z_{\text{ROW}}^{Q,Q';\ i,i';\ [t]\ 1\text{s}}\right]_j = \widetilde{\sum_{\substack{Q,\ i,\ Q',\ i' \text{ fixed };\\ j \text{ fixed in } Qi,\ j' \text{ in } Q'i';\\ j'\neq j \text{ if } Q=Q' \text{ and } i=i'\\ k'\neq k}}} \left[Qi - 1_{Qi}\alpha^{\mathsf{T}}\right]_{j,k} \Delta_{kk} \left[Q'i' - 1_{Q'i'}\alpha^{\mathsf{T}}\right]_{j',k} \left[T_{Q'i'}\right]_{j',t} \times \left[Q'i' - 1_{Q'i'}\alpha^{\mathsf{T}}\right]_{j',k'} \Delta_{k'k'} \left[Qi - 1_{Qi}\alpha^{\mathsf{T}}\right]_{j,k'} ,$$

$$\left[Z_{\text{COL}}^{Q,Q';\ i,i';\ [t]\ 1\text{s}}\right]_k = \widetilde{\sum_{\substack{Q,\ i,\ Q',\ i' \text{ fixed };\\ j \text{ in } Qi,\ j' \text{ in } Q'i';\\ j'\neq j \text{ if } Q=Q' \text{ and } i=i'\\ k \text{ fixed},\ k'\neq k}}} \left[Qi - 1_{Qi}\alpha^{\mathsf{T}}\right]_{j,k} \left[Qi - 1_{Qi}\alpha^{\mathsf{T}}\right]_{j,k'} \Delta_{k'k'} \times \left[Q'i' - 1_{Q'i'}\alpha^{\mathsf{T}}\right]_{j',k'} \left[T_{Q'i'}\right]_{j',t} \left[Q'i' - 1_{Q'i'}\alpha^{\mathsf{T}}\right]_{j',k} \Delta_{kk} .$$

Note that these covariant-dependent subscores depend on $[T_{Q'i'}]_{j',t}$, but not $[T_{Qi}]_{j,t}$. These 1-sided-subscores are then combined:

$$\left[Z_{\text{ROW}}^{Q,Q';\ i,i';\ [T]\ 1\text{s}}\right]_j^2 = \frac{1}{\kappa^2} \widetilde{\sum_t} \left[Z_{\text{ROW}}^{Q,Q';\ i,i';\ [t]\ 1\text{s}}\right]_j^2$$

$$\left[Z_{\text{COL}}^{Q,Q';\ i,i';\ [T]\ 1\text{s}}\right]_k^2 = \frac{1}{\kappa^2} \widetilde{\sum_t} \left[Z_{\text{COL}}^{Q,Q';\ i,i';\ [t]\ 1\text{s}}\right]_k^2 ,$$

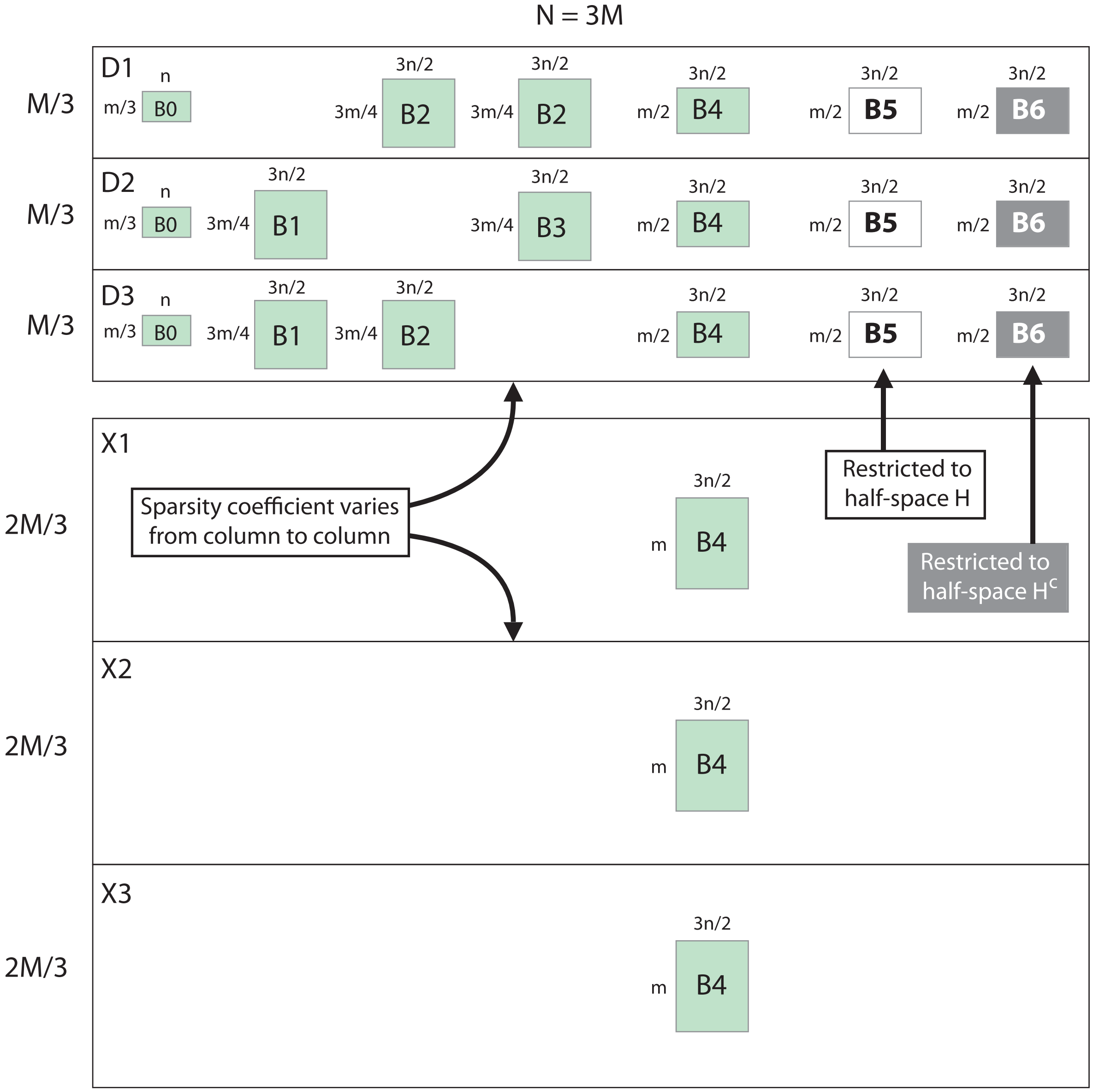

Figure 54: This figure illustrates the setup for the numerical experiments shown in Fig 55. See text for description.

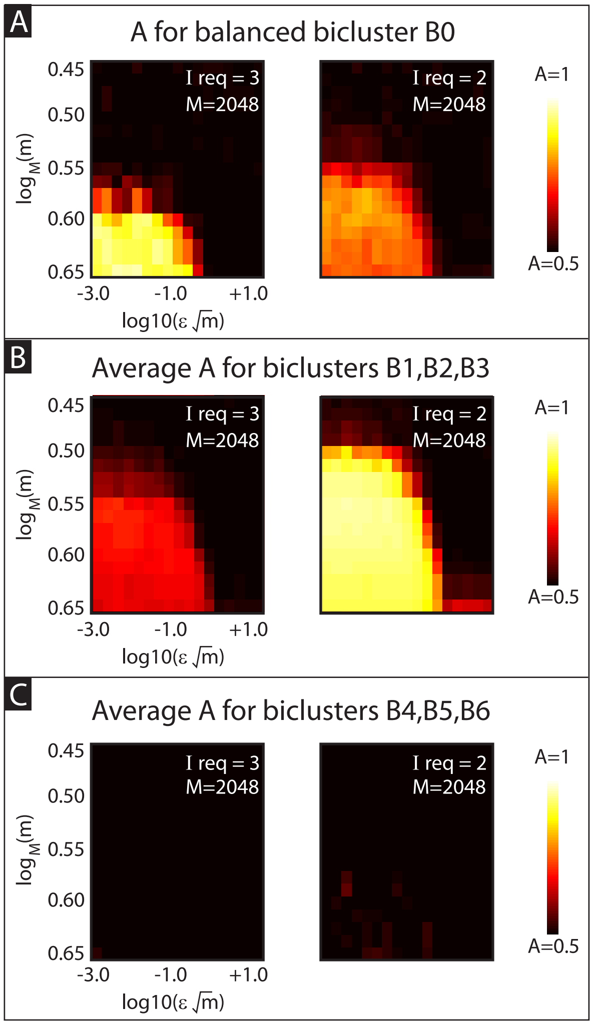

Figure 55: These numerical experiments illustrate the performance of our algorithm on the planted-bicluster problem described in Fig 54. In panel-A we show the trial-averaged value of A_0 as a function of $\varepsilon\sqrt{m}$ and $\log_M(m)$. In panel-B we show the trial-averaged value of A_1, A_2 and A_3. In panel-C we show the trial-averaged value of A_4, A_5 and A_6. The left side of each panel displays results with $I_{\text{req}} = 3$, the right side with $I_{\text{req}} = 2$. Note that, when $I_{\text{req}} = 3$ the goal was to find $B0$, as this was the only case-specific bicluster that was balanced across all the continuous-covariates and covariate-categories. On the other hand, when $I_{\text{req}} = 2$ the goal was to find the most dominant of $B0$ $B1$ $B2$ and $B3$, as each of these case-specific biclusters were balanced across all the continuous-covariates while extending across at least 2 covariate-categories. Note that our algorithm typically achieves these goal, despite the presence of the other biclusters $B4$ $B5$ and $B6$.

and contribute to the sub-scores for each Q, Q', i, i' as follows:

$$\left[Z_{\text{ROW}}^{Q,Q';\,i,i'}\right]_j = \left[Z_{\text{ROW}}^{Q,Q';\,i,i';\,\text{base}}\right]_j - \left[Z_{\text{ROW}}^{Q,Q';\,i,i';\,[T]\ \text{1s}}\right]_j$$

$$\left[Z_{\text{COL}}^{Q,Q';\,i,i'}\right]_k = \left[Z_{\text{COL}}^{Q,Q';\,i,i';\,\text{base}}\right]_k - \left[Z_{\text{COL}}^{Q,Q';\,i,i';\,[T]\ \text{1s}}\right]_k.$$

As mentioned earlier in section 10, these 1-sided corrections don't change the row-scores much, but do alter the column-scores. Under the 1-sided correction the column-subscores $Z_{\text{COL}}^{Q,Q';\,i,i';\,[T]\ \text{1s}}$ now capture the norm (rather than the norm-squared) of the averaged continuous-covarate-vector accumulated across any biclusters intersecting column-k.

With regards to case-specific biclusters, both the 2-sided and 1-sided continuous-covariate corrections accomplish the same general goal. That is to say, both correction schemes reduce the score of case-specific biclusters that are imbalanced with respect to the space of continuous-covariates, while leaving unaffected the scores of case-specific biclusters that are balanced in covariate-space (at least to to first order). However, these two correction-schemes differ in how they treat certain kinds of biclusters that straddle both cases and controls. The 2-sided correction penalizes biclusters that include a comparable number of cases and controls, regardless of how those cases and controls are distributed in covariate-space. The 1-sided correction also penalizes biclusters that include a comparable number of cases and controls, but behaves differently in one important situation: The 1-sided correction does not penalize biclusters that include both cases and controls as long as (i) the cases are balanced in covariate-space, and (ii) the controls are imbalanced in covariate-space. An illustration of this difference is given in Fig 56.

These two different strategies for continuous-covariate correction are useful in different applications. For example, the 1-sided correction is useful if we want to discover a covariate-independent case-specific signature from amongst a heterogenous group of controls. This kind of correction may be appropriate if we expect many different regions in covariate-space to each give rise to their own biclusters (which we should then need to correct for). On the other hand, the 2-sided correction is more conservative, and is more useful for highlighting case-specific biclusters that are robust to changes in the control population. This kind of correction may be appropriate if we want to isolate biclusters that remain significant when compared with arbitrary groups of controls (rather than merely covariate-balanced groups of controls); this kind of goal can be relevant for developing robust risk-prediction models.

Role of binarization: In the prescription above we choose to binarize T. This binarization allows for a more efficient calculation (see next section), but somewhat compromises the accuracy of the covariate-correction. More specifically, after binarization, the covariate-dependent subscores $[Z_{\text{ROW}}^{Q,Q';\,i,i';\,[T]}]_j$ (resp. $[Z_{\text{COL}}^{Q,Q';\,i,i';\,[T]}]_k$) only approximate the magnitude (resp. magnitude-squared) of the averaged-covariate-vector associated with any biclusters including row-j (resp. column-k). Once we binarize T, then this averaged-covariate-vector will depend not only on whether or not the relevant biclusters are imbalanced with respect to any half-space H, but also on the orientation of that half-space. For example, assuming the existence of a bicluster B that is fully imbalanced with respect to H, it is possible for H to be oriented in such a way that the covariate-correction does not fully demote the scores associated with B (e.g., the normal vector defining H can lie along a coordinate axis in covariate-space).

To avoid this scenario, and to ensure that all imbalanced biclusters are appropriately penalized, we could have included multiple versions of T, each randomly rotated (in covariate-space) before binarization. With this redundancy, we could calculate the covariate-dependent sub-scores (referred to in the previous paragraph) for each version of T, taking e.g., the highest magnitude across the versions. One issue with this approach is that there is a trade-off between speed and accuracy; the more versions of T we include, the more accurate an estimate we can produce, but the longer it takes to produce this estimate. At some point it becomes more efficient to simply skip the T-binarization step altogether, and just include the full covariate-vector for T.

For our GWAS example at the beginning (see section 1.4), as well as for our numerical-examples throughout, we've seen that these more sophisticated correction techniques are unnecessary; the simplest covariate-correction has sufficed. That is to say, we've binarized T, and only used a single version (with no additional random rotations).

13 Notes regarding computation

As we touched on earlier, the binarization step in our algorithm sets the stage for the efficient storage and manipulation of matrices and vectors. Many of the laborious computations involved in calculating the row- and column-scores can be accomplished without the use of floating-point operations. As an example, consider the row-sub-score $\left[Z_{\text{ROW}}^{Q,Q';\,i,i';\,[t]}\right]_j$, with $i = i'$, and $Q = Q' = D$. In the following equations we'll refer to the $M_{Di} \times 1$ vector 1_{Di} as simply 1, and suppress the post-scripts i and i' to ease the notation. We'll use matlab conventions for referring to indices: $D_{j,:}$ and $D_{:,k}$ refer to the jth-row and k^{th}-column of D, respectively. We'll also denote the entry-wise product as '.∗', and the entry-wise power as '.^'. I.e., $[Q. * Q']_{ij} = Q_{ij}Q'_{ij}$, and $\left[Q^{\cdot 2}\right]_{ij} = Q_{ij}^2$.

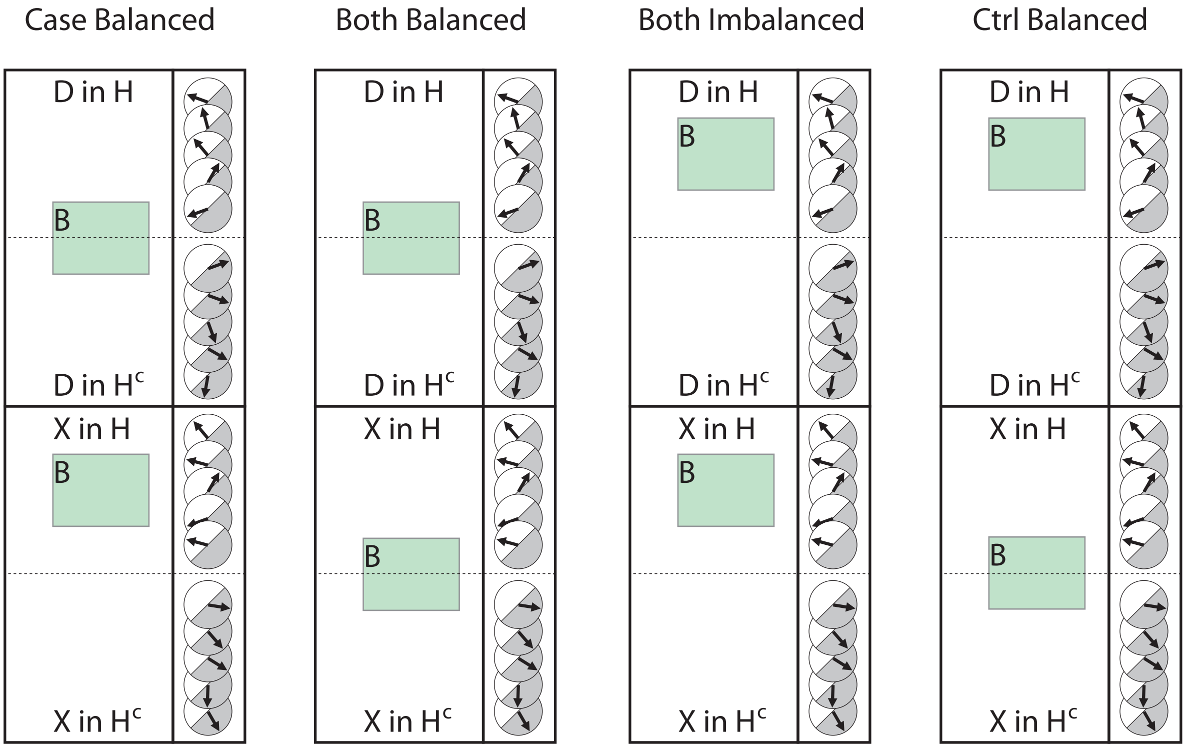

Figure 56: This figure illustrates some of the differences between the 2-sided and 1-sided continuous-covariate correction. In each situation we illustrate a data-matrix containing cases D and controls X. We assume that this data-matrix contains a bicluster B which includes both a case-specific and control-specific component of comparable size. We'll focus on the differences that result when these components of B occupy different regions in covariate-space. For ease of illustration, we've divided covariate-space into a half-space H (white), and its complement H^c (grey). Each of the four panels illustrates a different arrangement; the case- and control-specific components of B are either balanced or imbalanced with regards to the continuous-covariate. Panel 1 (Case Balanced): When the case-component is balanced but the control-component is imbalanced we see the greatest difference between the 2-sided and 1-sided scheme; the former fully demotes the column-score of B (reducing it to 0), whereas the latter does not penalize B at all. Panel 2 (Both Balanced): When both components of B are balanced, both the 2-sided and the 1-sided schemes fully demote the column-score (reducing it to 0). Panel 3 (Both Imbalanced): When both components of B are imbalanced, both the 2-sided and the 1-sided schemes fully demote the column-score (reducing it to 0). Panel 4 (Control Imbalanced): When the control-component is balanced but the case-component is imbalanced, both the 2-sided and the 1-sided schemes greatly lower the column-score, reducing it to a negative value.

The sub-score $\left[Z_{\text{ROW}}^{D,D;\ i,i';\ [t]}\right]_j$ can be calculated by first computing the $M_D \times M_D$ correlation-matrix 'C', defined via:

$$C = \left(D - 1\alpha^\intercal\right)\Delta\left(D^\intercal - \alpha 1^\intercal\right) = D\Delta D^\intercal - 1\alpha^\intercal\Delta D^\intercal - D\Delta\alpha 1^\intercal + \left(\alpha^\intercal\Delta\alpha\right)11^\intercal,$$

then computing

$$\left[Z_{\text{ROW}}^{\text{full sum};\ [t]}\right]_j = C_{j,:}\cdot\text{diag}\left(T_{:,t}\right)\cdot\ C_{:,j} = C_{j,:}^{\cdot 2}\cdot T_{:,t},$$

$$\left[Z_{\text{ROW}}^{j'=j;\ [t]}\right]_j = T_{j,t}\cdot C_{jj}^{\cdot 2},$$

$$\begin{aligned}\left[Z_{\text{ROW}}^{k'=k;\ [t]}\right]_j &= \left(D - 1\alpha^\intercal\right)_{j,:}^{\cdot 2}\Delta^{\cdot 2}\left(D^\intercal - \alpha 1^\intercal\right)^{\cdot 2}T_{:,t} \\ &= \left(11^\intercal - 2\left[D.*1\alpha^\intercal\right] + 1\alpha^{\cdot 2\intercal}\right)_{j,:}\Delta^{\cdot 2}\left(11^\intercal - 2\left[D^\intercal.*\alpha 1^\intercal\right] + \alpha^{\cdot 2}1^\intercal\right)T_{:,t},\end{aligned}$$

$$\left[Z_{\text{ROW}}^{j'=j\&\ k\prime=k;\ [t]}\right]_j = T_{j,:}\cdot\left[1_j 1_:^\intercal\Delta^{\cdot 2}1_: - 4\cdot D_{j,:}\text{diag}\left(\alpha\right)\Delta^{\cdot 2}1_: + 6\cdot 1_j\alpha_:^{\cdot 2\intercal}\Delta^{\cdot 2}1_: - 4\cdot D_{j,:}\text{diag}\left(\alpha^{\cdot 3}\right)\Delta^{\cdot 2}1_: + 1_j\alpha_:^{\cdot 4\intercal}\Delta^{\cdot 2}1_:\right],$$

and forming the combination:

$$\left[Z_{\text{ROW}}^{D,D;\ i,i';\ [t]}\right]_j = \frac{T_{j,t}}{(M-1)\,N\,(N-1)}\left\{\left[Z_{\text{ROW}}^{\text{full sum};\ [t]}\right]_j - \left[Z_{\text{ROW}}^{j=j';\ [t]}\right]_j - \left[Z_{\text{ROW}}^{k'=k;\ [t]}\right]_j + \left[Z_{\text{ROW}}^{j'=j\&\ k'=k;\ [t]}\right]_j\right\}.$$

All the terms in these expressions can be rearranged to only involve matrix-vector products, with the exception of $D\Delta D^\intercal$ in the calculation of C.

The matrix D is binary, and therefore a matrix-matrix product such as $DD^\intercal$ can be calculated using a binary matrix-matrix product. This binary matrix-matrix product does take $O\left(M^2N\right)$ operations, but can be computed rapidly by appealing to bitwise operations rather than floating-point operations. These bitwise operations typically allow for multiple matrix entries to be processed simultaneously. For example, if $\vec{u} = D_{j,:}$ and $\vec{v} = D_{j',:}$ are two binary row-vectors from D, then the dot-product $\vec{u}^\intercal\cdot\vec{v}$ can be calculated by first breaking up $\vec{u}$ and $\vec{v}$ into disjoint chunks, each comprising R adjacent bits:

$$\vec{u}_r = \left[\vec{u}_{rR-R+1},\ldots,\vec{u}_{rR}\right],\ \vec{v}_r = \left[\vec{v}_{rR-R+1},\ldots,\vec{v}_{rR}\right]\ \text{for}\ r = 1,\ldots,N/R,$$

and then using bitwise operations on each pair of chunks $\vec{u}_r, \vec{v}_r$:

$$\vec{u}^\intercal\vec{v} = N - 2\sum_{r=1,\ldots,N/R}\text{popcount}\left(\text{xor}\left(\vec{u}_r,\vec{v}_r\right)\right).$$

In the above calculation we assume that R is the number of bits that can be conveniently loaded at once (e.g., $R = 128*(16-1) = 1920$), and that N is a multiple of R (or that $\vec{u}$ and $\vec{v}$ have been extended in length and appropriately masked or padded). The xor operation refers to a bitwise 'exclusive-or', and the popcount operation sums the number of nonzero bits.

Our situation is slightly more complicated than the simple example above because the diagonal matrix Δ is not binary; rather than simply forming $\vec{u}^\intercal\vec{v}$, we have to form $\vec{u}^\intercal\Delta\vec{v}$, which cannot be easily computed using only bitwise operations. Fortunately, in practice the size of Δ is usually very large (e.g., $N \gtrsim 10^5$), and the variances $\delta_k^{-1} = 4p_kq_k$ are bounded above by 1 and usually bounded below by a constant greater than 0. We take advantage of this observation by sorting the columns of D so that the diagonal entries of Δ are monotonically ordered. We then approximate each block of R entries of Δ by a single constant equal to their average:

$$\bar{\delta}_r = \frac{1}{R}\left(\delta_{rR-R+1} + \cdots + \delta_{rR}\right),$$

and then calculate:

$$\vec{u}^\intercal\Delta\vec{v} = \sum_r\bar{\delta}_r\left\{N_r - 2\cdot\text{popcount}\left(\text{xor}\left(\vec{u}_r,\vec{v}_r\right)\right)\right\},$$

where N_r is the number of column-indices in chunk r. This procedure allows us to generate $D\Delta D^\intercal$, and ultimately C, by using $O\left(M^2N/R\right)$ bitwise-operations, rather than $O\left(M^2N\right)$ floating-point multiplications.

The column-sub-score $\left[Z_{\text{COL}}^{D,D;\ i,i';\ [t]}\right]_k$ has a similar structure, and can be calculated by first computing

$$\left[Z_{\text{COL}}^{\text{full sum};\ [t]}\right]_k = \left(D_{k,:}^\intercal - \alpha_k 1_:^\intercal\right)\text{diag}\left(T_{:,t}\right)C\text{diag}\left(T_{:,t}\right)\left(D_{:,k} - 1_:\alpha_k^\intercal\right),$$

$$\left[Z_{\text{COL}}^{k'=k;\ [t]}\right]_k = \left[\left(1_k1^\intercal - 2D_{k,:}^\intercal.*\alpha_k1^\intercal + \alpha_k^{\cdot 2}1^\intercal\right)T_{:,t}\right]\cdot\left[\left(1_k1^\intercal - 2D_{k,;}^\intercal.*\alpha_k1^\intercal + \alpha_k^{\cdot 2}1^\intercal\right)T_{:,t}\right],$$

$$\left[Z_{\text{COL}}^{j'=j;\ [t]}\right]_k = \left(1_k 1^\intercal - 2D^\intercal_{k,:} .* \alpha_k 1^\intercal + \alpha_k^{.2} 1^\intercal\right) \cdot \text{diag}\left(T_{:,t}^{.2}\right) \cdot \left(11^\intercal - 2D .* 1\alpha^\intercal + 1\alpha^{.2\intercal}\right) \Delta 1_:$$

$$\left[Z_{\text{COL}}^{j'=j\&\ k'=k;\ [t]}\right]_k = \Delta_{k,k} \cdot \left[1_k 1_:^\intercal - 4 \cdot D^\intercal_{k,:} .* \alpha_k 1^\intercal + 6 \cdot \alpha_k^{.2} 1^\intercal - 4 \cdot D^\intercal_{k,:} .* \alpha_k^{.3} 1^\intercal + \alpha_k^{.4\intercal} 1^\intercal\right] \cdot T_{:,t}^{.2},$$

and then forming the combination:

$$\left[Z_{\text{COL}}^{D,D;\ i,i';\ [t]}\right]_k = \frac{\Delta_{k,k}}{(N-1)\, M\, (M-1)} \left\{ \left[Z_{\text{COL}}^{\text{full sum};\ [t]}\right]_k - \left[Z_{\text{COL}}^{k'=k;\ [t]}\right]_k - \left[Z_{\text{COL}}^{j'=j;\ [t]}\right]_k + \left[Z_{\text{COL}}^{j'=j\&\ k'=k;\ [t]}\right]_k \right\}.$$

As in the case with the row-sub-score, all the expressions within this column-sub-score involve matrix-vector products, with the exception of $D\Delta D^\intercal$ in C, which requires a matrix-matrix product.

Re-Binarization of C: We pause to remark on the calculation of

$$\left[Z_{\text{COL}}^{\text{temp};\ [t]}\right]_k := D^\intercal_{k,:} \text{diag}\left(T_{:,t}\right) C \text{diag}\left(T_{:,t}\right) D_{:,k} \text{ within } \left[Z_{\text{COL}}^{\text{full sum};\ [t]}\right]_k.$$

In practice N is typically much larger than any M_{Qi}; i.e., for genome-wide-association-studies (GWAS) the total number of genetic-measurements (i.e., SNPs) can be on the order of 10^{5-6} or more, while the total number of patients is only on the order of 10^{3-4}. This implies that, even though calculating $D^\intercal_{k,:}\text{diag}(T)\, C\text{diag}(T_{:,t})\, D_{:,k}$ for each k is technically $O\left(NM\right)$ floating-point operations, this calculation can often be the bottleneck for our entire algorithm. In these cases it is advantageous to take advantage of the fact that both D and T are binary-matrices. Although C is not a binary-matrix itself, we can represent C using a collection of b binary-matrices (one such binary-matrix for each significant bit of C we wish to preserve). This kind of representation allows us to calculate $Z_{\text{COL}}^{\text{temp};\ [t]}$ using b binary matrix-matrix calculations, each requiring $O\left(NM^2/R\right)$ bitwise-operations. We find that this procedure is often more efficient than the direct calculation of $Z_{\text{COL}}^{\text{temp};\ [t]}$, and is sufficiently precise so long as we preserve sufficiently many bits of C (i.e., if b is sufficiently high). When the sparsity-coefficients p_k are all close to 0.5, such as in gene-expression-analysis, then 8 bits of C are typically sufficient (i.e., we choose $b \gtrsim 8$). In cases where the sparsity coefficients p_k extend from close to 0 up to 0.5, such as in GWAS, more bits of C are necessary, and we typically choose $b \gtrsim 24$. This re-binarization can be applied not only to the matrix C that we introduced at the beginning of section 13, but also to the other correlation-matrices involved in the calculation of $\left[Z_{\text{ROW}}^{Q,Q';\ i,i';\ [t]}\right]_j$ and $\left[Z_{\text{COL}}^{Q,Q';\ i,i';\ [t]}\right]_k$ which have a similar structure to C.

More rows than columns: Some applications involve data-matrices D and X with more rows than columns (e.g., $M_Q > N$). In these situations the calculations above are not efficient; they require $O(M_Q^2 N)$ work, when $O(M_Q N^2)$ work would be preferable. To arrange the calculation so that only $O(M_Q N^2)$ work is required, we can first calculate the $N \times N$ matrix E:

$$E = \left(D^\intercal - \alpha 1^\intercal\right) \text{diag}\left(T_{:,t}\right) \left(D - 1\alpha^\intercal\right),$$

and then compute

$$\left[Z_{\text{ROW}}^{\text{full sum};\ [t]}\right]_j = \left(D - 1\alpha^\intercal\right) \cdot \Delta \cdot E \cdot \Delta \cdot \left(D^\intercal - \alpha 1^\intercal\right), \text{ and}$$

$$\left[Z_{\text{COL}}^{\text{full sum};\ [t]}\right]_k = E_{k,:} \cdot \Delta \cdot E_{:,k} = E_{k,:}^{.2} \cdot \text{diag}\left(\Delta\right).$$

Parallelization and Timing: The full algorithm described in section 12 involves both case- and control-matrices, as well as I_{cat} covariate categories and N_T continuous-covariate coefficients. There are many different ways to organize this calculation, with different considerations pertaining to different computing architectures. Our current implementation at the time of this writing uses different nodes to process the different trials (e.g., the original data as well as label-shuffled-trials from H0x). For each trial running on a given node, we use different processors within the node to calculate the scores associated with different combinations of Q', i, i', and t. This implementation is certainly not always optimal (e.g., in the D-only case), but is reasonably efficient for many applications. For example, the GWAS data-set shown in Example-3 was processed over a weekend; the computation used 65 nodes (one for the original-data, and 64 others for the label-shuffled-trials from H0x), each with 16 processors (Intel Xeon Processor E5-2650 v2). The total computation-time per-node was $\lesssim 12$ hours, with γ set to 0.05 and $b = 32$ bits retained for each correlation-matrix. If, instead, we had only retained $b = 8$ bits (which is sufficient for many problems) and not corrected for the $N_T = 2$ continuous covariates, then the total computation-time per-node would have been reduced by a factor of ~ 12. If necessary, a further reduction in computation-time could be obtained by increasing γ.

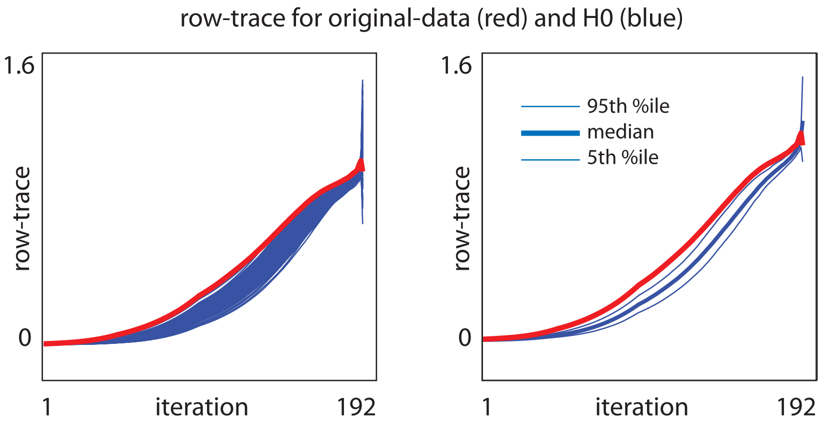

Figure 57: Row-traces for the bicluster shown in Example-1b from section 1.2. This bicluster was found by running our loop-counting algorithm on the data shown in Fig 5. For this example we corrected for cases-vs-controls; we compare our original-data to the distribution we obtain under the null-hypothesis H0. On the left we show the row-trace as a function of iteration for the original-data (red) as well as each of the 256 random shuffles (blue). On the right we replot this same trace data, showing the 5th, 50th and 95th percentile (across iterations) of the H0 distribution. Because we are not correcting for any covariates, the column-traces are identical to the row-traces.

14 Notes regarding application

Our biclustering algorithm produces three lists as output. The first lists the rows and columns of the data-matrix in the order in which they were eliminated. The second lists the average row-score taken across the remaining data-matrix at each iteration. The third lists the average column-score taken across the remaining data-matrix at each iteration. We refer to these average row- and column-scores as row- and column-traces; they are both equal to the trace of $DD^{\intercal}DD^{\intercal}$ in the D-only situation, but will in general be different when correcting for cases-vs-controls and covariates. In practice it is often critical to determine

- How to interpret the algorithm's output,
- How significant this output is,
- Whether or not a bicluster was found, and what the boundaries of the bicluster are.
- How to find the second, third, and subsequent biclusters.

The answers to these questions are, in general, application dependent. We comment on some of our experiences below.

14.1 Interpreting the output:

We have run our algorithm in the context of gene-expression analysis and genome-wide-association-studies (i.e., GWAS). In both cases we were comparing case-patients to control-patients, at times correcting for various covariates. In the context of gene-expression-analysis we had the luxury of normalizing the data so that the sparsity-coefficient for each column was $p_k = 0.5$. This choice of normalization meant that we did not need to correct for sparsity; each $\alpha_k = 0$ and $\delta_k = 1$. Because each loop contributed either a $+1$ or a -1 to our total score, the row- and column-traces produced by loops within D were bounded between -1 and $+1$. Similarly, the traces produced by loops entering X were also bounded between ± 1. The full trace produced by our algorithm was thus bounded by ± 2, and was usually in the interval $[0, 1]$. We could interpret the output in this situation relatively easily: If, after sufficiently many iterations, the row- or column-traces grew large (i.e., came close to $+1$), then the remaining rows and columns should form a highly correlated low-rank bicluster. An illustration along these lines is shown in Figs 57 and 58, which corresponds to our Example-1b for gene-expression-analysis. A similar illustration is given in Figs 59 and 60, which corresponds to our Example-2 for gene-expression-analysis (which involves categorial covariates). Similarly, Figs 61 and 62 correspond to Example-1a.

For GWAS the same interpretation is not valid; the GWAS data is itself binary because each allele is either dominant or recessive. This means that we do not have the luxury of normalizing the data to alter the sparsity-coefficients; typically the sparsity-coefficients for genotyped-data range from close to 0 all the way up to 0.5. This range of sparsity-coefficients makes interpreting the output of our algorithm more difficult. It is now possible for any loop to contribute a large magnitude to the total scores. The row- and column-traces are no longer bounded by a small number like ± 1 or ± 2, and often rise to be much higher towards the end of the iterations when the few remaining columns have extreme sparsity-coefficients. In

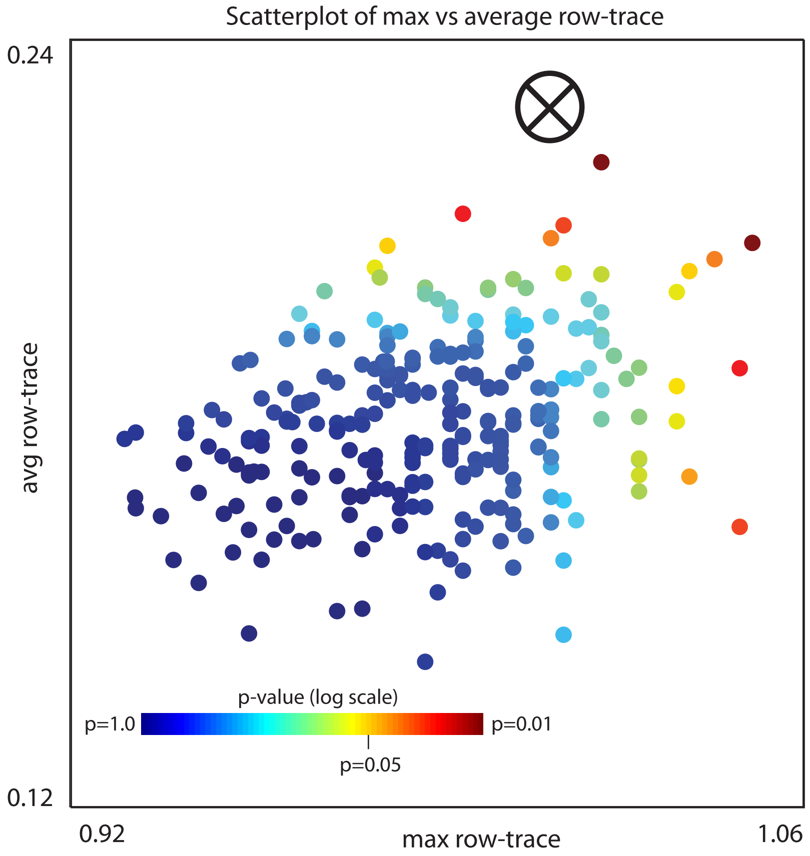

Figure 58: We show a scatterplot of the data shown in Fig 57. Each row-trace shown on the left in Fig 57 is plotted as a single point in 2-dimensional space; the horizontal-axis corresponds to the maximum row-trace and the vertical-axis corresponds to the average row-trace (taken across the iterations). The original-data is indicated with a '$\otimes$', and each of the random shuffles with a colored '$\bullet$'. The p-value for any point $\vec{w}$ in this plane is equal to the fraction of label-shuffled-traces that have either an x-position larger than x_w or a y-position larger than y_w, where x_w and y_w are the x- and y-percentiles associated with the most extreme coordinate of $\vec{w}$ (details given in section 14.2). Each random shuffle is colored by its p-value determined by the label-shuffled-distribution. By comparing the original-trace with the shuffled-distribution we can read off a p-value for the original-data of $\lesssim 0.008$.

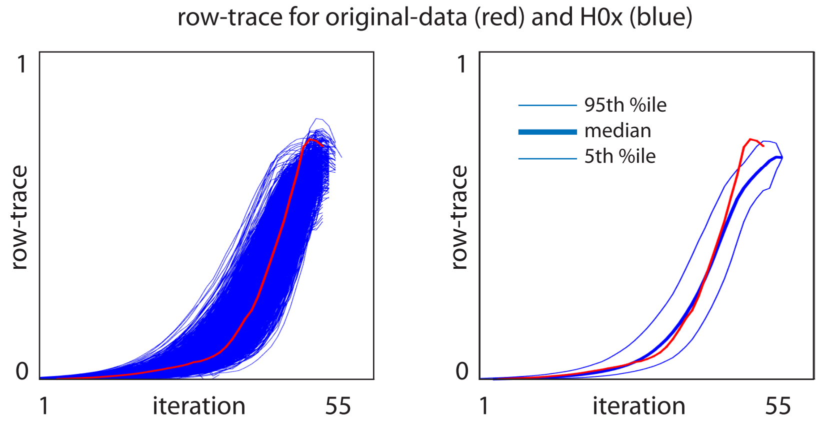

Figure 59: Row-traces for the bicluster shown in Example 2 in section 1.3. This bicluster was found by running our loop-counting algorithm on the data shown in Fig 8. For this example we corrected for cases-vs-controls as well as the categorical-covariates of gender and ethnicity. Because we corrected for categorical-covariates, we compare our original-data to the distribution we obtain under the null-hypothesis H0x. On the left we show the row-trace as a function of iteration for the original-data (red) as well as each of the 2048 random shuffles (blue). Note that the different traces terminate at different iteration-numbers. This is due to the fact that different trials eliminate the various covariate-categories at different iteration-numbers; for each trace we only include iterations for which all covariate-categories remain. On the right we replot this same trace data, showing the 5th, 50th and 95th percentile (across iterations) of the H0x distribution. While not exactly identical to the row-traces, the column-traces are very similar (not shown).

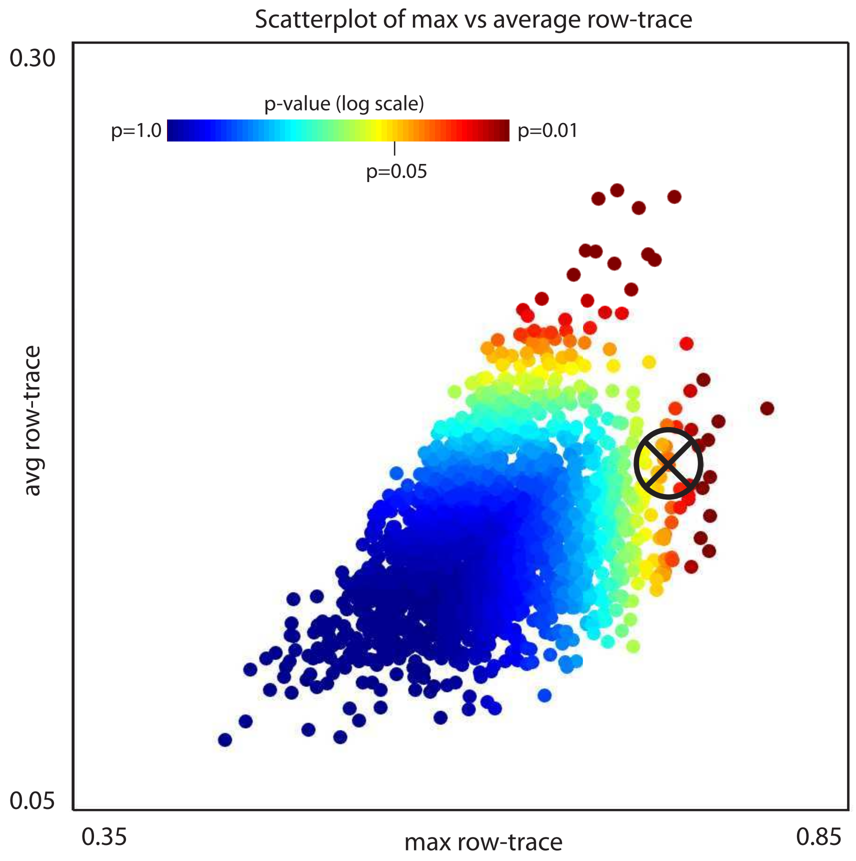

Figure 60: We show a scatterplot of the data shown in Fig 59. The format of this figure is similar to that of Fig 58. By comparing the original-trace with the shuffled-distribution we can read off a p-value for the original-data of ~ 0.027.

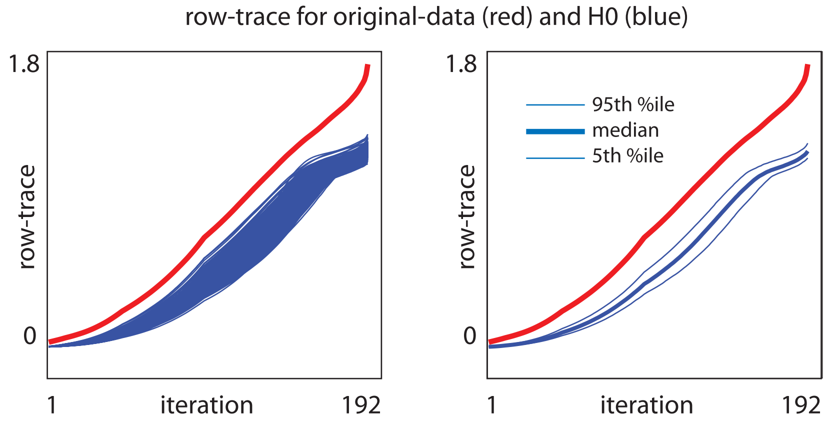

Figure 61: Row-traces for the bicluster shown in Example-1a from section 1.2. This bicluster was found by running our half-loop algorithm on the data shown in Fig 2. For this example we corrected for cases-vs-controls; we compare our original-data to the distribution we obtain under the null-hypothesis H0. On the left we show the row-trace as a function of iteration for the original-data (red) as well as each of the 512 random shuffles (blue). On the right we replot this same trace data, showing the 5th, 50th and 95th percentile (across iterations) of the H0 distribution. Because we are not correcting for any covariates, the column-traces are identical to the row-traces.

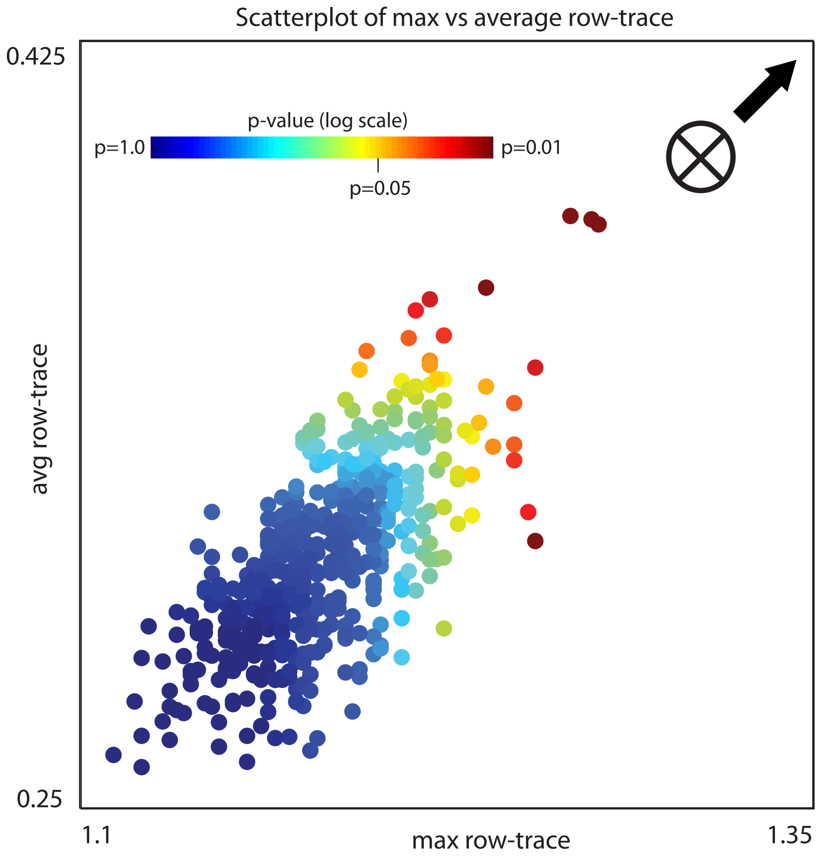

Figure 62: We show a scatterplot of the data shown in Fig 61. The format for this scatterplot is similar to that shown in Fig 58, except that the original-trace lies far to the upper-right of the distribution drawn from H0 (see arrowhead). By comparing the original-trace with the shuffled-distribution we can read off a p-value for the original-data of $\lesssim 1/512$ (i.e., bounded above by the reciprocal of the number of samples drawn from H0).

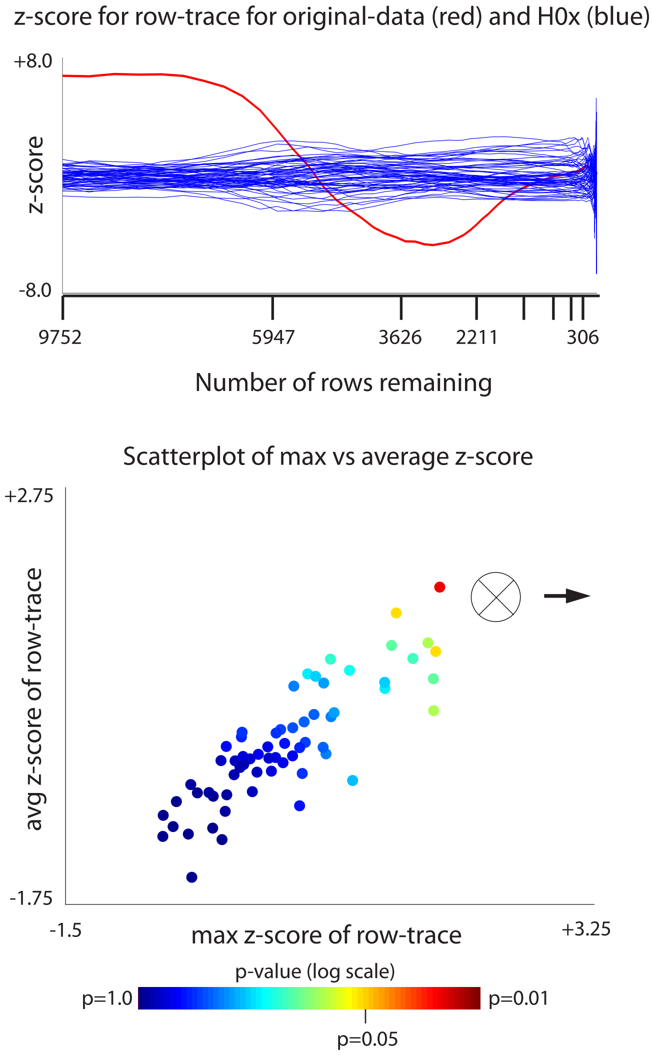

Figure 63: Z-scores for the bicluster shown in section 1.4. This bicluster was found by running our algorithm on the GWAS data-set referenced in 1.4. For this example we corrected for cases-vs-controls, as well as continuous-covariates and sparsity. Because we corrected for continuous-covariates, we compare our original-data to the distribution obtained under H0x. Because the data-set had many columns with extreme sparsity-coefficients, the magnitude of our row- and column-traces is not particularly meaningful by itself. A more meaningful measurement is the z-score of the row- and column-traces (calculated with respect to the label-shuffled distribution). On top we plot this z-score as a function of the number of rows remaining. The original-trace is shown in red, and the 64 random shuffles from H0x are shown in blue. On the bottom we show a scatterplot of the maximum z-score (horizontal) and average z-score (vertical) taken across the iterations. The format for this scatterplot is similar to that shown in Fig 58, except that the original-trace lies far to the right of the distribution drawn from H0x (see arrowhead). By comparing the original-trace to the shuffled-distribution, we can read off a p-value of $\lesssim 1/64$ (i.e., bounded above by the reciprocal of the number of samples drawn from H0x).

this situation the most natural way to determine whether a low-rank structure has been found is to compare the traces for the original data to traces obtained after label-shuffling the data (see the next subsection).

14.2 Determining significance:

Our typical hypothesis (say, H1) is that there exists some disease-related structure in the case-patients that is not exhibited by the control-patients. This is in contrast to the null-hypothesis (H0) in which the case- and control-labels are actually arranged randomly and have no disease-related structure. Under this null-hypothesis the traces we observe after running our algorithm on the original data should be similar to the traces we would find if we were to shuffle the case-control labels randomly (across patients). To draw a sample from this null-hypothesis H0, we shuffle the case-control labels of the patients indiscriminantly while retaining the same number of cases and controls. If we are correcting for covariates, then we restrict our null-hypothesis slightly (H0x) so that the random-shuffles respect the covariates. For example, if we were correcting for gender as a categorical-covariate, we would shuffle the labels of case-males only with control-males, and shuffle the labels of case-females only with control-females. If we were correcting for a continuous-covariate, we would shuffle the case-control labels of patients in each orthant of covariate-space.

For each label-shuffled trial we rerun our algorithm, and collect the output. Each trial does not depend on any of the other trials, and they can all be processed in parallel. This library of label-shuffled traces produces a distribution associated with H0 (or H0x). We use this label-shuffled distribution to calculate a p-value for the traces produced by the original data.

Example-1 and Example-2:

The row- and column-traces each have one value per iteration. Two of the most straightforward measurements for each trace are (i) the maximum value, and (ii) the average value, with the latter computed using a measure where each trace-value contributes a weight proportional to the number of rows or columns eliminated during that iteration. When considering gene-expression-data these two measurements are useful and have a natural interpretation. On one hand, (i) traces that reach a high maximum during a late iteration correspond to small but tightly-correlated biclusters. On the other hand, (ii) traces that may not have an exceptionally high maximum value but are consistently above the median for many consecutive iterations correspond to larger but more loosely-correlated biclusters. The biclusters shown in Example-1a, Example-1b and Example-2 illustrate the first phenomenon (i), as shown in Figs 61, 57 and 59.

We can compare our original-data with the label-shuffled-distribution to obtain a p-value for our original-trace. We have done this using the 2-dimensional scatterplots shown in Figs 62, 58 and 60. For illustration, consider Fig 60. Each of the $L = 2048$ label-shuffled-traces has an x- and a y-position on this scatterplot. We'll denote the position of trace-l by (x_l, y_l). Let's also denote by $F(x)$ the fraction of label-shuffled traces with an $x_l < x$, and by $G(y)$ the fraction of label-shuffled traces with a $y_l < y$. Let's further denote by $x(F)$ the x-position of the Fth-percentile of the x_l samples (i.e., $x(F)$ is the inverse of $F(x)$). Let's also denote by $y(G)$ the y-position of the Gth-percentile of the y_l samples (i.e., $y(G)$ is the inverse of $G(y)$). Now we can calculate the p-value of any location $\vec{w} = (w_x, w_y)$ in the x, y-plane as follows.

1. Calculate $F(w_x)$ and $G(w_y)$,
2. Let $H = \max(F(w_x), G(w_y))$ be the more extreme of the two.
3. Calculate $x(H)$ and $y(H)$,
4. find the set $\Omega = \{l \text{ such that either } \; x_l \geq x(H) \; \text{ or } \; y_l \geq y(H)\}$.
5. Set the p-value for $\vec{w}$ to be the fraction of label-shuffled-traces in Ω: i.e., p-value $= p(w_x, w_y) = |\Omega| / L$.

We remark that this method of calculating p-values is 'self-consistent'; the distribution of p-values across the label-shuffled traces will be uniform by construction. Specifically, the collection of $p(x_l, w_l)$ taken across l will be uniformly distributed in $[0, 1]$ and the empirical cdf of these p-values will be a diagonal line with slope 1. The more standard bonferroni-correction $p = \max(F(w_x), G(w_y))$ is an overestimate and will not produce a uniform distribution of p-values across the label-shuffled traces when F and G are correlated.

Note that the label-shuffled null-hypothesis H0x is 'nested-within' (and more restrictive than) the null-hypothesis H0. Specifically, the collection of label-permutations allowed under H0x is a subset of the label-permutations allowed under H0. The label-shuffles which mix patients in different covariate categories (allowed under H0) are typically less likely to exhibit the same structures as the label-shuffles allowed under H0x. As a result, a smaller fraction of the trials drawn from H0 are likely to 'look like the real data', as compared with H0x. Consequently, the p-value obtained from the H0-distribution will typically be smaller (i.e., indicating a more significant result) than the more conservative p-value obtained using the more reasonable H0x-distribution.

Example-3:

When considering GWAS data the maximum and average of the trace are not useful measurements because of the varying sparsity-coefficients in the data. Because of these varying sparsity-coefficients, the trace can take on values far outside the interval $[-1, 1]$, as is typical when there are only a few iterations remaining. Standard measurements such as the max- and average-trace are strongly influenced by the values of the trace during the final few iterations. To account for this bias, we transform each trace by replacing its value at each iteration with its z-score calculated with respect to the distribution of the label-shuffled-traces at that iteration. The max and average of the transformed-traces have an analogous interpretation to the original measures (i) and (ii) described earlier, except that the correlations revealed should be interpreted relative to the label-shuffled distribution, rather than in an absolute sense. Traces that reach a high z-score during a late iteration correspond to biclusters which, although small, are more tightly-correlated than the typical small bicluster seen at that iteration. Traces that have a high average z-score correspond to biclusters that are larger, but somewhat less correlated. The bicluster shown in Example-3 illustrates this latter phenomenon, as shown in Fig 63.

14.3 Delineating a bicluster:

Example-1 and Example-2:

One way to visualize the structure highlighted by the biclustering algorithm is to calculate a 'shape-matrix' S. To create S we first order the rows and columns of the original data-matrix in terms of their importance (i.e., the longest retained rows and columns come first). With this ordering we define S_{jk} as the average trace of the 'corner-submatrix' obtained by considering the j most important rows and the k most important columns. This calculation can be performed for each j and k, revealing the subsets of rows and columns for which the trace is high. The decay of S_{jk} as j and k increase will indicate the shape of the structure found. We llustrate some shape-matrices in Figs 64 and Figs 65, which correspond to our Example-1a and Example-1b. The shape-matrix in Fig 66 corresponds to our Example-2, while the shape-matrices in Fig 67 correspond to some other biclusters we have found in other gene-expression data-sets.

We note that, towards the end of our iteration, there are not always sufficiently many patients remaining to represent all the covariate-categories (e.g., in Fig 66 $I_{\text{cat}} = 4$). When the number of remaining covariate-categories drops (e.g., when the last noncaucasian female is eliminated), our algorithm reduces the number of covariate-categories I_{cat} involved in the score-calculation, and reduces I_{req} as necessary. This abrupt change in I_{cat} and I_{req} results in the discontinuities of S in the j-direction seen in Fig 66B. When defining p-values and delineating the bicluster we only consider iterations where all the initial covariate-categories are represented.

To actually delineate a bicluster we invoke a user-specified threshold $\tau \in (0, 1)$; in this case $\tau = 0.7$. Given τ, We find the j, k for which S_{jk} is equal to the fraction τ of its maximum, and jk is as large as possible. In other words, we find the largest corner-submatrix with average-trace τS_{max}. As an example, the biclusters shown in Figs 64, 65 and 66 were delineated using this technique.

This procedure (i.e., picking out a corner-submatrix) does indeed do a reasonable job of delineating rectangular biclusters when they exist, and is not very sensitive to τ when the rectangular biclusters are sharply defined. Nevertheless, this kind of delineation does not always tell the whole story; corner-submatrices are not always the best indicator of the various sorts of structure that can be revealed by S in practice. For example, as seen in Figs Figs 64 and 67, our algorithm can often reveal patterns within gene-expression data that are not quite rectangular. These shape-matrices may indicate some of the more complicated underlying structures discussed in section 7.3. Thus, in summary, it is important to note that bicluster-delineation will always be somewhat arbitrary; in practice there will rarely be a clear-cut boundary to any bicluster. Because of this arbitrariness, we have structured our methodology so that the details regarding bicluster-delineation do not affect the bicluster's p-value (which only depends on the traces, and not the threshold τ).

Example-3:

The S-matrix described above is useful when the goal is to pick out a small sharply-correlated bicluster – i.e., when the traces output by our algorithm are significantly high towards the end of the iterations. However, if the output-traces are not high towards the end of the iterations, then this S-matrix may not be particularly meaningful. This situation arises in Example-3, where the output-traces are significantly higher than the H0x-distribution for the first several iterations, but not towards the end (see Fig 63). If we were to try and use the S-matrix to to delineate this bicluster, then we would need to compare the S_{jk} produced by the original-data with the S_{jk} produced by the random-shuffles from H0x; the former would only be significantly greater than the latter when j is in the thousands and k is in the hundreds-of-thousands – clearly not a sharp delineation of any kind. Thus, for Example-3, we choose to delineate the bicluster by using the last peak of the output-trace relative to the H0x-distribution (i.e., the last peak of the z-score). This final peak occurs with 115 case-patients and 706 alleles remaining (i.e., the submatrix shown in Fig 11).

14.4 Finding secondary biclusters:

So far we have been concerned with finding a single bicluster hidden within a large data-matrix. In practice of course, many large data-sets are home to multiple biclusters, some of which are distinct and some of which overlap to varying degrees.

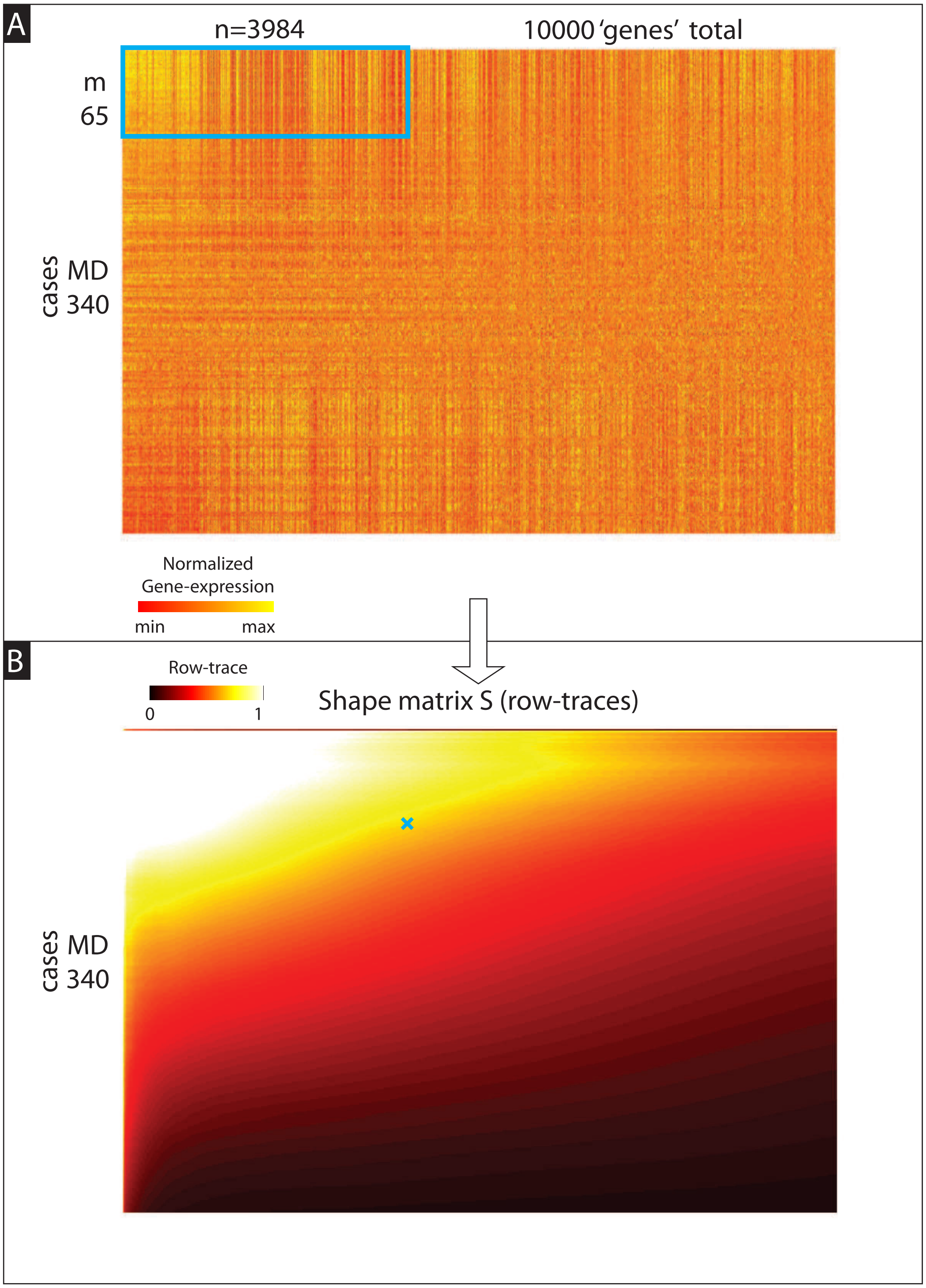

Figure 64: Illustration of 'shape-matrix' S for Example-1a shown in Fig 3. In Panel-A we show a subset of the D-matrix rearranged according to the output of our algorithm, with the most important rows and columns listed first. We show all $M_D = 340$ patients, but only the 10000 most important of the $N = 11711$ genes. The upper left corner holds the bicluster shown in Fig 3A, as indicated by the cyan box. In Panel-B we show the shape-matrix S, which records the row-trace for each corner-submatrix of the output shown in Panel-A. Specifically, given the rearranged version of D shown in Panel-A, we calculate S_{jk} to be the row-trace associated with rows $\{1, \ldots, j\}$ and columns $\{1, \ldots, k\}$. The entry of S for $j = 65$ and $k = 3984$ corresponds to the submatrix within the cyan box, and is indicated with a cyan '$\times$'. This shape-matrix reveals a rather smeared-out bicluster; while there are many rows and columns that clearly participate, the shape of the bicluster is not obviously rectangular. Consequently, the delineation of a 'corner-submatrix' will depend somewhat sensitively on the methodology used (e.g., the particular value of τ we use to choose the corner).

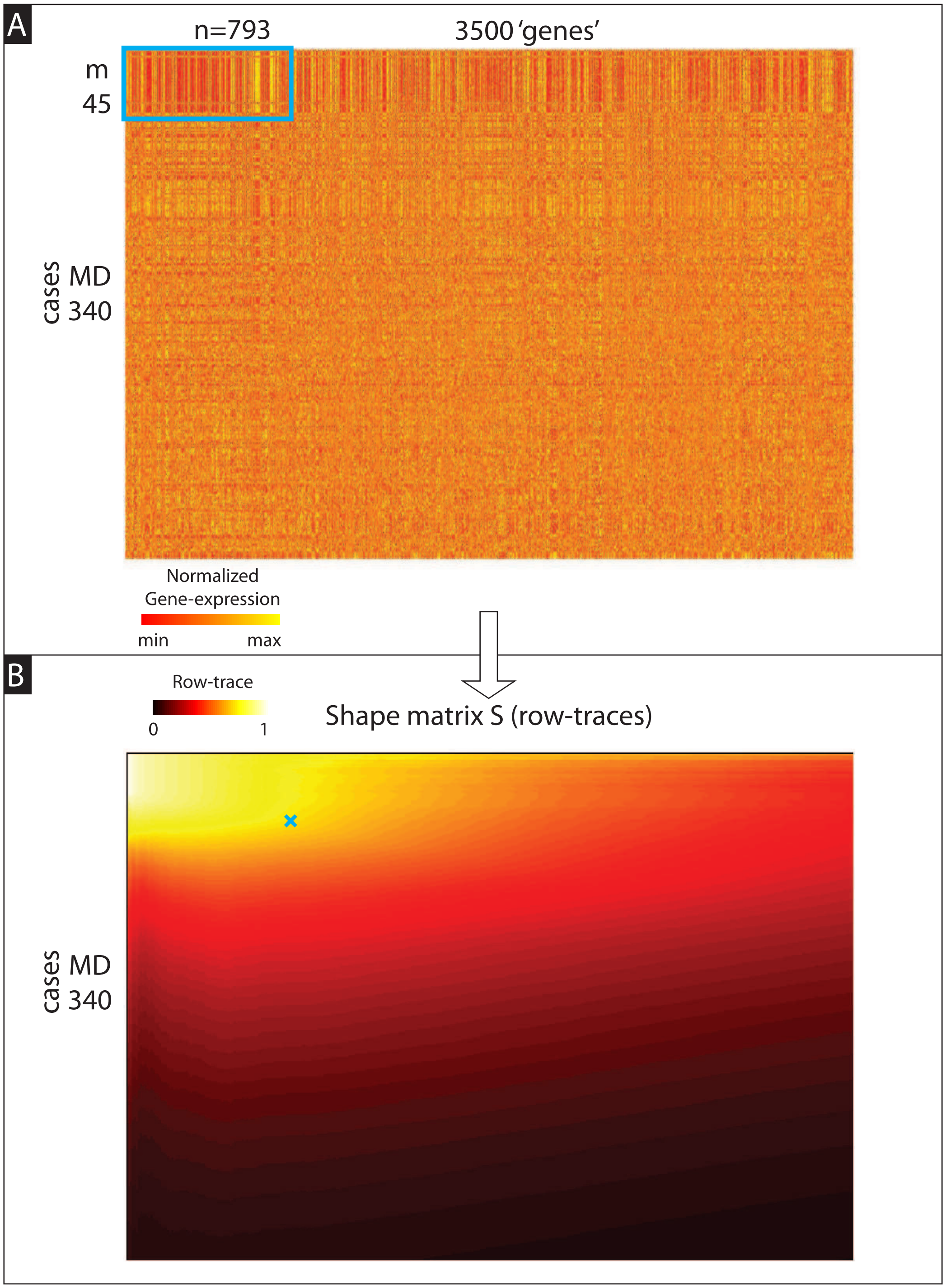

Figure 65: Illustration of 'shape-matrix' S for Example-1b shown in Fig 6. The format of this figure is similar to that of Fig 64. We show all $M_D = 340$ patients, but only the 10000 most important of the $N = 16738$ genes. The entry of S for $j = 45$ and $k = 793$ corresponds to the submatrix within the cyan box, and is indicated with a cyan '×'. This shape-matrix reveals a rather rectangular bicluster; the delineation of this bicluster is not very sensitive to our threshold of $\tau = 0.7$.

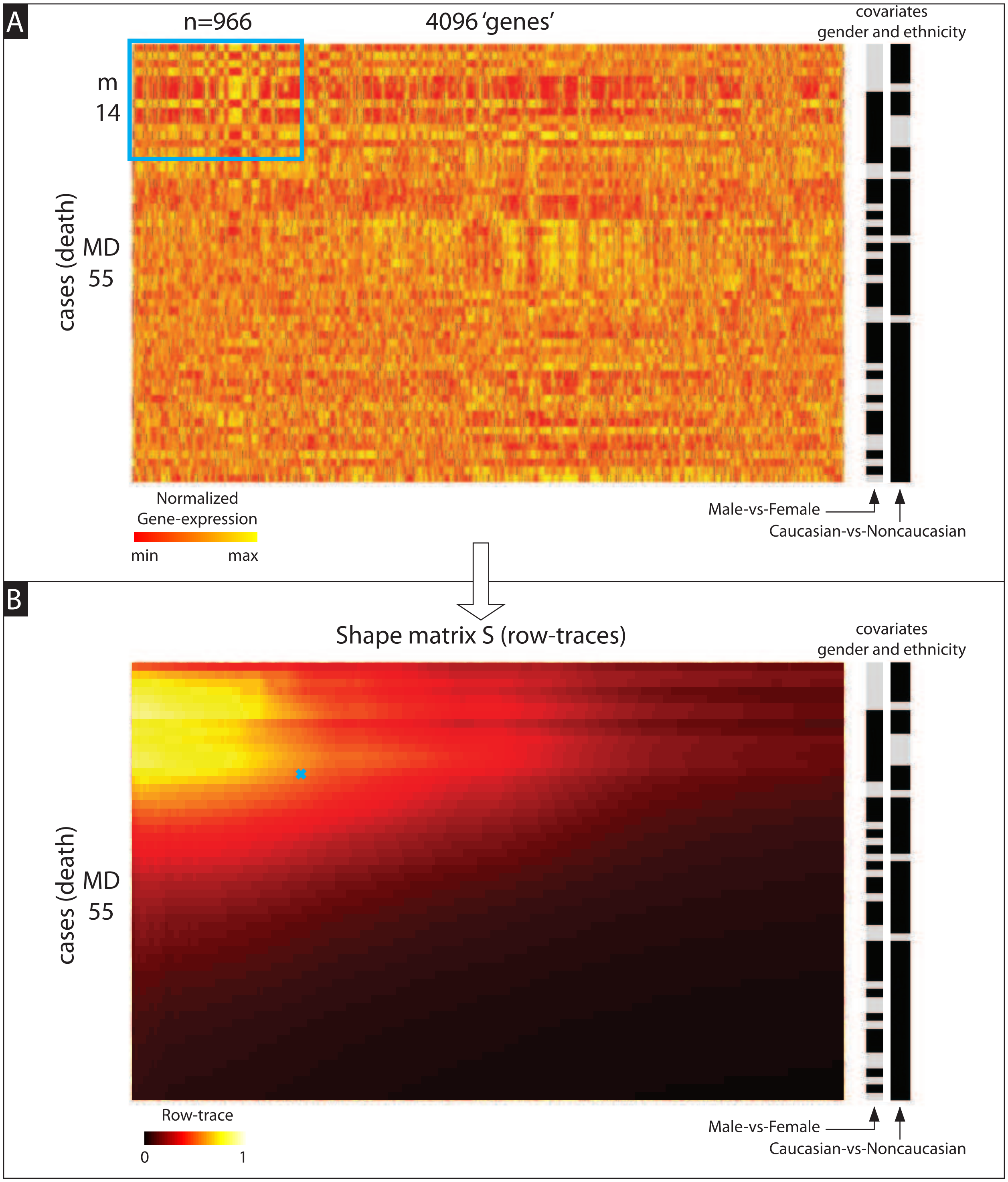

Figure 66: Illustration of 'shape-matrix' S for Example-2 shown in Fig 9. The format of this figure is similar to that of Fig 64. We show all $M_D = 55$ patients, but only the 4096 most important of the $N = 17942$ genes. The entry of S for $j = 14$ and $k = 966$ corresponds to the submatrix within the cyan box, and is indicated with a cyan '×'. This shape-matrix reveals a rather rectangular bicluster; the delineation of this bicluster is not very sensitive to our threshold of $\tau = 0.7$.

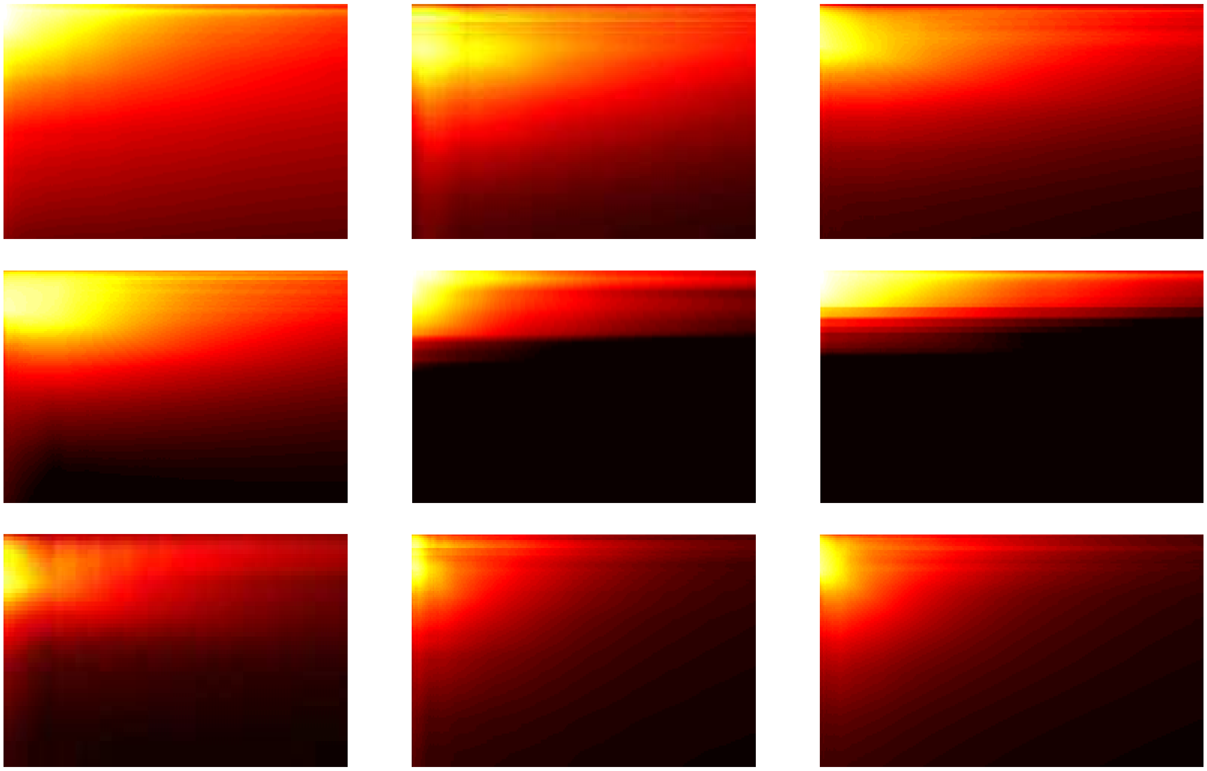

Figure 67: Illustration of some other shape-matrices we obtained when running our algorithm on various gene-expression data-sets. Some of these shape-matrices suggest rectangular biclusters, but others suggest more 'smeared-out' structures, some similar to the kinds of structures discussed in section 7.3.

What does our algorithm do when the data-set has multiple biclusters, and how do we find these multiple biclusters?

As we've discussed above in section 4, our algorithm usually finds the most 'dominant' bicluster within the data; that is, the bicluster that produces the greatest signal (i.e., the largest and/or most tightly correlated bicluster). This first most dominant bicluster will often be highlighted by the corners of the shape matrix 'S' described in section 14.3. In some cases secondary biclusters will also be highlighted by the shape matrix, trailing the first (see Fig 33).

Situations as clean as Fig 33 don't usually arise in practice, however, and we've found it useful to search for secondary biclusters using repeated passes of our algorithm. More specifically, after running our algorithm for the first time (and delineating the most dominant bicluster), we then rerun our algorithm from scratch, scrambling the data within the first detected bicluster to destroy any signal it might create. This second pass of our algorithm will then locate the second most dominant bicluster. After this we can perform a third pass, scrambling the first two biclusters and so forth.

Note that when we scramble the data within the biclusters we have found so far, we should respect the constraints compatible with our null hypothesis (e.g., H0, H0x, etc.). For example, if we were considering categorical-covariates we should scramble the data within a bicluster by randomly interchanging/replacing matrix-entries within (but not across) covariate-categories. This procedure ensures that we destroy (most of) the signal associated with the previously discovered biclusters without introducing any large new spurious signals.

We remark that secondary passes of our algorithm only rarely produce traces that are as high as those produced by the first pass (after all, the secondary biclusters typically have a weaker signal than the first). Nevertheless, we currently believe that it is best to assess the p-value of secondary biclusters by comparing their traces with the original shuffled-distribution of traces used to assess the most dominant bicluster (rather than with a new shuffled-distribution associated with second passes of our algorithm). We adopt this methodology because it is conservative: we use the same standard to assess both secondary and primary biclusters, even though this standard may underestimate the significance of secondary biclusters.

The results of this approach for Example-2 are shown in Fig 68. Note that the first trace is the most pronounced. This phenomenon is typical for the gene-expression data sets we have analyzed; while certainly not impossible, it is rare for a secondary bicluster to be more significant than a previously found bicluster.

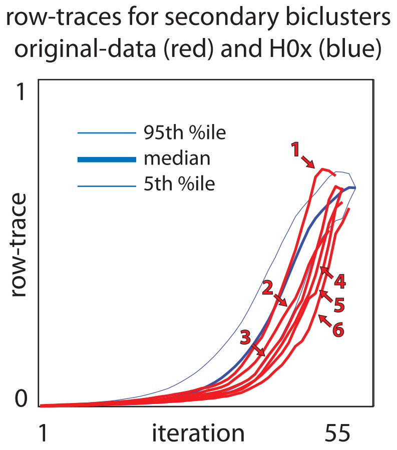

Figure 68: Here we show the row-traces for the secondary biclusters associated with Example-2 from section 1.3. These biclusters were found by successively rerunning our algorithm on the data shown in Fig 8 after scrambling any previously found biclusters. In the background we indicate the distribution of traces drawn from the shuffled-distribution (blue lines). These are identical to those shown in Fig 59. In the foreground we indicate the row-traces for the various passes of our algorithm (red numbers and lines). The first pass (corresponding to the most dominant bicluster) was shown previously in Fig 59).

15 Additional Applications

We have focused so far on the detection of low-rank biclusters. The simplest situation involves locating a 'rank-1' bicluster B hidden within an $M \times N$ data-matrix D. Because the bicluster B can be approximated with a single outer-product $u \cdot v^{\intercal}$, we have chosen to consider loops (i.e., 2×2 submatrices) within D. The low-rank structure of B ensures that loops within B are likely to be rank-1 (rather than rank-2). Moreover, the rows and columns of D that intersect B will contribute to many more rank-1 loops than the rows and columns that don't. Using these observations, our algorithms accumulates the loops within D; exposing B in the process. We've also discussed how to search for low-rank biclusters within a data-matrix involving cases and controls, considering along the way how to account for sparsity as well as categorical- and continuous-covariates.

Our methods can be easily extended to tackle many related problem. For example, we can treat 'genetic controls', look for 'rank-0' biclusters (i.e., differentially-expressed biclusters), and even look for 'triclusters'. We discuss these topics briefly below.

15.1 Accounting for genetic-controls:

In some applications the goal is to find biclusters that are not merely specific to a subset of case-rows, but also to a subset of case-columns. Such a situation may arise in gene-expression analysis when the goal is to find biclusters restricted to one set of genes that do not extend to encompass the gene-expression measurements taken from another set of genes. As an example, let's return to the Example-2 in section 1.3. The particular bicluster we already found in section 1.3 involved many genes and pathways that are already known to affect colon-cancer; these genes are involved in many pathways related to DNA-replication, DNA-repair, mitosis, etc. Instead of focusing on these well-known pathways, we might want to deliberately ignore them and search for signals within other genes; hopefully discovering genes that participate in less well understood pathways.

One way to go about this is to classify some of the genes in our data-set as 'control-genes'; i.e., genes that we deliberately want to avoid when searching for low-rank structure. For this example we'll use as genetic-controls a list of genes that are already well-recognized to affect colon-cancer (see the discussion towards the end of sec 1.3). To recap: we generate our set of control-genes by first choosing three of the most well documented genes which influence colon-cancer: MLH1, MSH2 and MSH6. Our second step involves using 'Seek' (see [$\mathcal{M}$42]) to generate a list of genes that are commonly co-expressed alongside these three cancer-related genes. We then define our control-genes to be all the genes in this list which have a combined co-expression value (with MLH1, MSH2 and MSH6) of at least 0.75. The control-gene set generated this way comprises 1659 of our original 17941 genes. As we alluded to above, this control-gene set is *strongly* enriched for DNA-replication, DNA-repair, mitosis, etc. (e.g., p-values on the order of 1e-20 or below); these are the pathways we want to ignore. We'll classify the remaining 16282 genes as 'case-genes', and search for a bicluster within these case-genes that does not exhibit a low-rank structure that is shared by a significant fraction of the control-genes.

We can divide our measurements into the following data-matrices:

- the $M_D \times N_D$ case-matrix D: The entry $D_{j,k}$ is the kth case-gene measurement from the jth-case patient.
- the $M_X \times N_D$ control-matrix X: The entry $X_{j,k}$ is the kth case-gene measurement from the jth-control patient.
- the $M_D \times N_Y$ control-matrix Y: The entry $Y_{j,k}$ is the kth control-gene measurement from the jth-case patient.
- the $M_X \times N_Y$ control-matrix W: The entry $W_{j,k}$ is the kth control-gene measurement from the jth-control patient.

Our goal will be to find co-expressed subsets of genes within the case-population; we would like to find a low-rank bicluster within D. As before, we are most interested in biclusters which are case-patient-specific and don't extend to include many control-patients from X. In addition, we would also like to focus on biclusters which are case-gene-specific, and don't extend to include many gene-expression measurements from the control-genes that are commonly co-expressed with MLH1, MSH2 and MSH6 (i.e,. Y). Put another way, we are interested in finding co-expressed subsets of genes that are (as best as possible) isolated to the case-patient-population and restricted to the 'undiscovered' subset of case-genes.

In order to accomplish this goal, we can extend the techniques used above, counting loops as before. This time, however, we'll also count loops that go through D and Y, as well as loops that go through all four data-matrices D, X, W and Y. Similar to the situation with both cases and controls (see section 8), we'll flip the sign on loops passing through X, and also flip the sign of loops passing through Y. Consequently, we'll end up positively counting loops passing through W. One way of understanding this choice of signs is that (i) we want to reward low-rank loops that are fully contained in D, and (ii) penalize low-rank loops that extend from D to X or from D to Y. The reason we want to reward low-rank loops that pass through all four data-matrices (i.e., through W) is that we want to ensure that biclusters are not unfairly penalized when the data-set has ambient correlations. Put another way, we would like the average row- and column-scores of random data (with no planted biclusters) to be 0. We choose the sign associated with the W-loops so that this average score remains 0 even when the random data is drawn from a non-isotropic gaussian (rather than an isotropic gaussian).

The row-scores built using this intuition take the following form (see Fig 69):

$$\left[Z_{\text{ROW}}^{DDDD}\right]_j = \widetilde{\sum_{\substack{j \text{ fixed},\ j'\in D,\ j'\neq j;\\ k'\neq k}}} D_{jk}D_{j'k}D_{j'k'}D_{jk'}, \quad \left[Z_{\text{ROW}}^{DXXD}\right]_j = \widetilde{\sum_{\substack{j \text{ fixed},\ j'\in X;\\ k'\neq k}}} D_{jk}X_{j'k}X_{j'k'}D_{jk'}$$

$$\left[Z_{\text{ROW}}^{DDYY}\right]_j = \widetilde{\sum_{\substack{j \text{ fixed},\ j'\in D,\ j'\neq j;\\ k\in D,\ k'\in Y}}} D_{jk}D_{j'k}Y_{j'k'}Y_{jk'}, \quad \left[Z_{\text{ROW}}^{DXWY}\right]_j = \widetilde{\sum_{\substack{j \text{ fixed},\ j'\in X;\\ k\in D,\ k'\in Y}}} D_{jk}X_{j'k}W_{j'k'}Y_{jk'},$$

and are combined as follows:

$$\left[Z_{\text{ROW}}\right]_j = \left[Z_{\text{ROW}}^{DDDD}\right]_j - \left[Z_{\text{ROW}}^{DXXD}\right]_j - \left[Z_{\text{ROW}}^{DDYY}\right]_j + \left[Z_{\text{ROW}}^{DXWY}\right]_j.$$

The column-scores, on the other hand, take the following form:

$$\left[Z_{\text{COL}}^{DDDD}\right]_k = \widetilde{\sum_{\substack{j',j\in D,\ j'\neq j;\\ k \text{ fixed},\ k'\neq k}}} D_{jk}D_{jk'}D_{j'k'}D_{j'k}, \quad \left[Z_{\text{COL}}^{DDXX}\right]_k = \widetilde{\sum_{\substack{j\in D,\ j'\in X;\\ k \text{ fixed},\ k'\neq k}}} D_{jk}D_{jk'}X_{j'k'}X_{j'k}.$$

$$\left[Z_{\text{COL}}^{DYYD}\right]_k = \widetilde{\sum_{\substack{j',j\in D,\ j'\neq j;\\ k\in D \text{ fixed},\ k'\in Y}}} D_{jk}Y_{jk'}Y_{j'k'}D_{j'k}, \quad \left[Z_{\text{COL}}^{DYWX}\right]_k = \widetilde{\sum_{\substack{j\in D,\ j'\in X;\\ k\in D \text{ fixed},\ k'\in Y}}} D_{jk}Y_{jk'}W_{j'k'}X_{j'k}.$$

and are combined as follows:

$$\left[Z_{\text{COL}}\right]_k = \left[Z_{\text{COL}}^{DDDD}\right]_k - \left[Z_{\text{COL}}^{DDXX}\right]_k - \left[Z_{\text{COL}}^{DYYD}\right]_k + \left[Z_{\text{COL}}^{DYWX}\right]_k.$$

A simple example illustrating this scenario is shown in Figs 70 and 71, where we consider a variation on the planted-bicluster problem involving both a planted case-specific bicluster and various planted non-specific biclusters involving both the patient- and genetic-controls.

When we actually carry out this method on the data from section 1.3 we do indeed find a significant bicluster, shown in Figs 72-75. Not only is this bicluster significant with respect to the label-shuffled hypothesis, but it is also enriched strongly for many different pathways. The most strongly enriched pathways include substrate-specific channel-activity (p=3e-8), passive-transmembrane-transporter-activity (p=1e-7) and cation-transport (p=1.5e-6); all of which are distinct from the most prominent pathways affiliated with the control-genes (as desired).

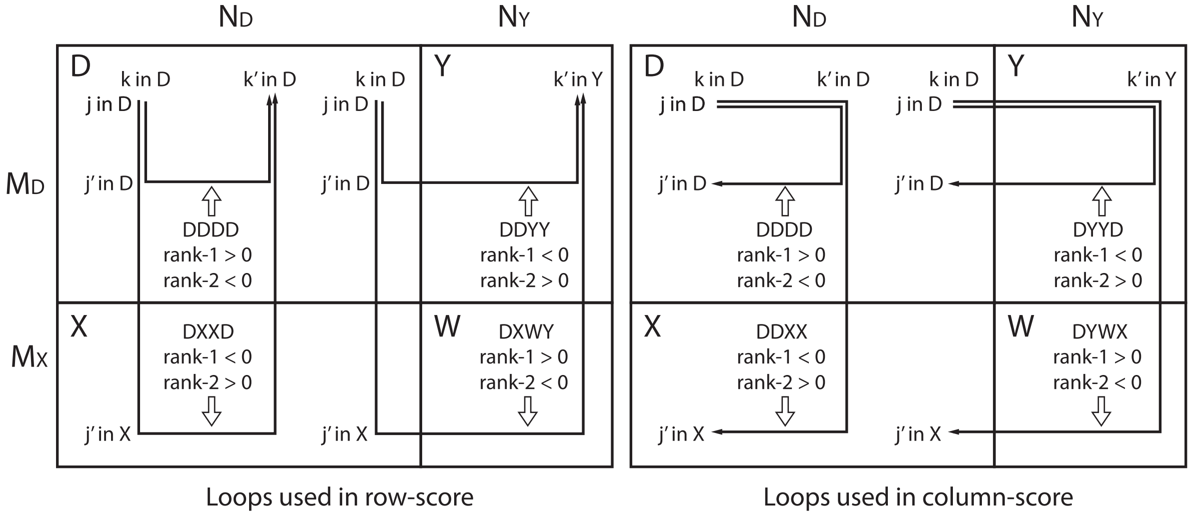

Figure 69: Illustration of the different kinds of loops that contribute to the row-score in the presence of genetic-controls. In the left panel we show some loops which contribute to the row-score; on the right we show some loops which contribute to the column-score. In each panel we illustrate an $M_D \times N_D$ case-matrix D (upper left) and an $M_X \times N_D$ control-matrix X (bottom left). In addition, we illustrate an $M_D \times N_Y$ matrix of genetic-controls Y (upper right) and an $M_X \times N_Y$ matrix of genetic-controls for the control-patients W (bottom right). Some of the loops are contained entirely within D, some extend from D to X, some extend from D to Y, and some extend to include D, X, W and Y. We count the first and last type of loop positively if they are rank-1, and negatively if they are rank-2. We count the second and third type of loop positively if they are rank-2, and negatively if they are rank-1.

15.2 Searching for 'rank-0' (i.e., differentially-expressed) biclusters:

As mentioned above, our algorithms accumulate the loops within D. While conceptually straightforward, the accumulation of these loops requires a matrix-matrix multiplication, limiting the efficiency of our approach (i.e., requiring at least $O(\max(M,N)MN)$ work).

If, instead of attempting to find generic rank-1 biclusters, we restrict our search to a particular kind of rank-1 bicluster, we can overcome this limitation and make our algorithms even more efficient (e.g., requiring only $O(MN)$ work). Specifically, we can greatly reduce the run-time of our algorithm if we decide to search only for special rank-1 biclusters $B \sim 1 \cdot v^{\intercal}$; i.e., where the column-vector 'u' has been replaced by a vector of all-ones. These biclusters are characterized as follows: each column of the bicluster is either high (i.e., mostly above the median for that column) or low (i.e., mostly below the median for that column). This means that, after binarization, the 'high' columns of the bicluster are mostly '+1', and the 'low' columns are mostly '−1'. Put another way, the rows/columns of the bicluster clump together near a single point in high-dimensional space; the bicluster is effectively rank-0. These biclusters can also be thought of as 'differentially-expressed': the genes in the biclusters are each differentially-expressed when comparing the patients in the bicluster to the rest of the population.

To detect these rank-0 biclusters we don't actually need to consider full loops; simpler half-loops will suffice (see Fig 76). The accumulation of half-loops is substantially easier than the accumulation of full loops, and does not require a matrix-matrix multiplication. Consequently, our methodology can be applied to the case of rank-0 biclusters with a reduced computational cost of only $O(MN\log(\max(M,N)))$ work.

All of our corrections involving sparsity, categorical- and continuous-covariates work just as well here. The formulae in section 12 can be carried forward, replacing all the loops with the associated half-loops. Specifically, we redefine our base-scores as follows:

$$\left[Z_{\text{ROW}}^{Q,Q';\ i,i';\ \text{base}}\right]_j = \widetilde{\sum_{\substack{Q,\ i,\ Q',\ i' \text{ fixed };\\ j \text{ fixed in } Qi,\ j' \text{ in } Q'i';\\ j'\neq j \text{ if } Q=Q' \text{ and } i=i'\\ k\in Q}}} \left[Qi - 1_{Qi}\alpha^{\intercal}\right]_{j,k} \Delta_{kk} \left[Q'i' - 1_{Q'i'}\alpha^{\intercal}\right]_{j',k},$$

$$\left[Z_{\text{COL}}^{Q,Q';\ i,i';\ \text{base}}\right]_k = \widetilde{\sum_{\substack{Q,\ i,\ Q',\ i' \text{ fixed };\\ j \text{ in } Qi,\ j' \text{ in } Q'i';\\ j'\neq j \text{ if } Q=Q' \text{ and } i=i'\\ k \text{ fixed},}}} \left[Qi - 1_{Qi}\alpha^{\intercal}\right]_{j,k} \Delta_{kk} \left[Q'i' - 1_{Q'i'}\alpha^{\intercal}\right]_{j',k},$$

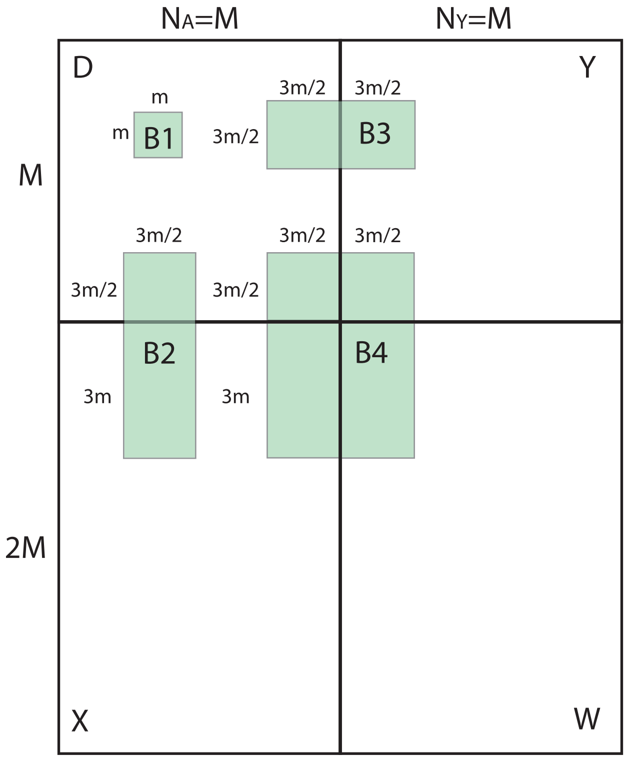

Figure 70: This figure illustrates the setup for the numerical experiments shown in Fig 71. These numerical experiments illustrate the performance of our algorithm on a simple planted-bicluster problem involving both case- and control-patients, along with case- and control-genes. The case-genes associated with the case- and control-patients are stored in matrices D and X, respectively. The control-genes associated with the case- and control-patients are stored in matrices Y and W, respectively. For each numerical experiment we let D be a large $M \times M$ random matrix with entries chosen independently from a distribution with median 0. We construct X, Y, and W in a similar fashion, except that X, Y and W are $2M \times M$, $M \times M$ and $2M \times M$, respectively. For each experiment we implant within the case-matrix D a small $m \times m$ bicluster $B1$ that is rank-1 with error ε. The rows and columns of $B1$ are chosen at random. We also implant a second rank-1 error-ε 'red-herring' bicluster $B2$ within both the case-matrix D as well as the control-matrix X. To implant $B2$ we first randomly choose $3m/2$ case-genes K and $3m/2$ case-patients J^D from D, as well as $3m$ control-patients J^X within X. We then construct a $3 \cdot 3m/2 \times 3m/2$ bicluster $B2$ that is rank-1 with error ε, and implant the top third of $B2$ into the chosen case-patients J^D and the bottom two-thirds of $B2$ into the chosen controls J^X (in each case using the chosen case-genes K). We implant another bicluster $B3$ in a similar fashion, except that $B3$ extends across both D and Y, and the total size of $B3$ is $3m/2 \times 2 \cdot 3m/2$. To implant $B3$ we first randomly choose $3m/2$ case-genes K_D from D and another $3m/2$ control-genes K_Y from Y, as well as $3m/2$ case-patients J from D. After constructing the rank-1 error ε matrix $B3$, we implant the left half of $B3$ into the chosen case-patients J and case-genes K_D, while implanting the right half of $B3$ into the chosen case-patients J and control-genes K_Y. Finally, we implant a fourth bicluster $B4$ which extends across D, X, W and Y. The bicluster $B4$ is of total size $3 \cdot 3m/2 \times 2 \cdot 3m/2$, and we create and implant it in a manner analogous to biclusters $B2$ and $B3$ above. We remark that our illustration is somewhat misleading, as the biclusters $B1$, $B2$, $B3$, and $B4$ are not typically disjoint. Because the row- and column-subsets for these biclusters are chosen randomly, there is typically a small overlap between them. For the purposes of this numerical experiment we implant each bicluster in the order described above: any overlapping entries will be overwritten by the next bicluster in the list (with biclusters $B2$, $B3$, $B4$ taking precedence). For each numerical experiment we calculate the average auc A_1 for $B1$, A_2 for $B2$, and so forth. Our goal is to find $B1$, despite the masking effect of the larger biclusters.

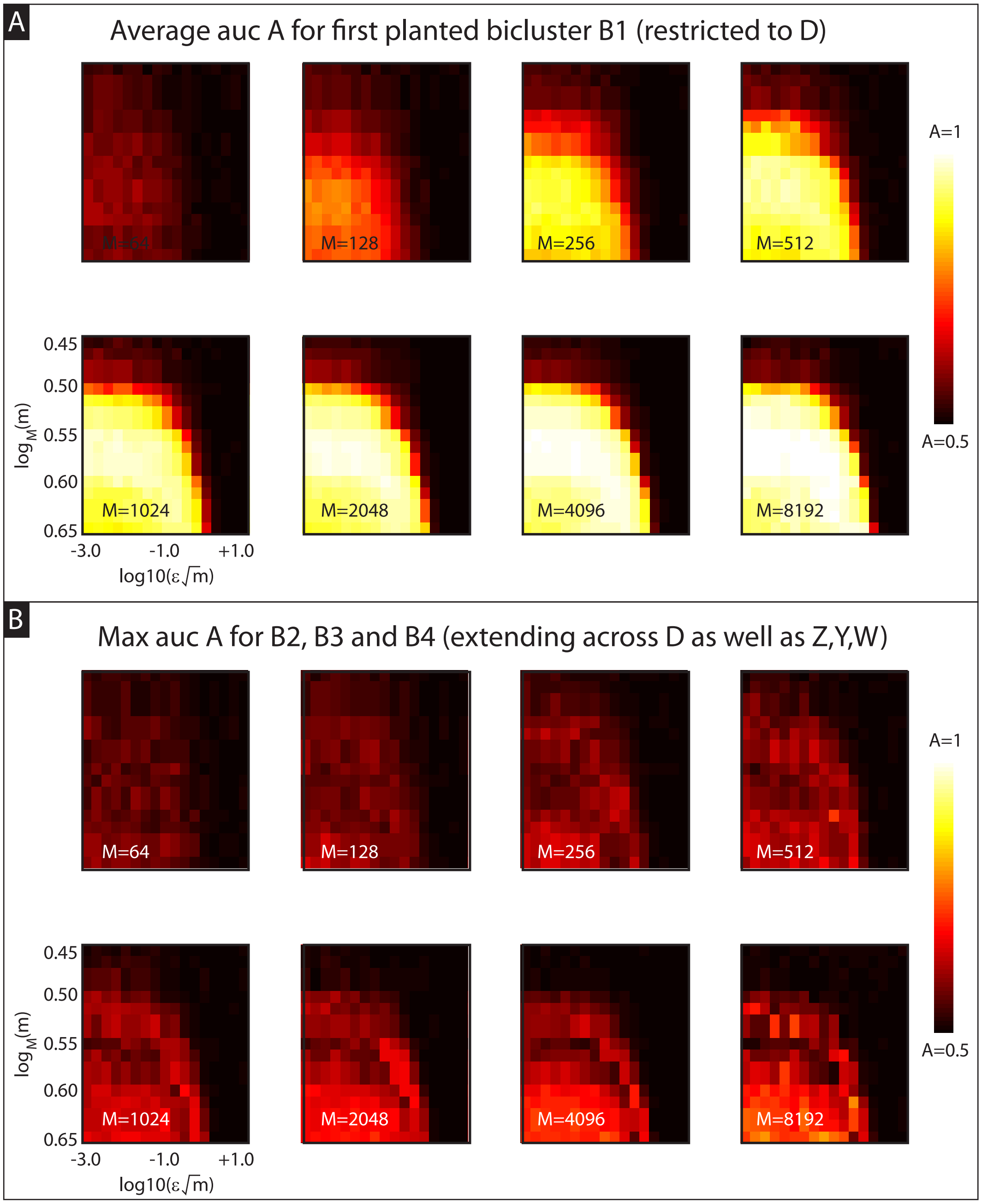

Figure 71: These numerical experiments illustrate the performance of our algorithm on a simple planted-bicluster problem involving both case- and control-patients as well as case- and control-genes (see Fig 70). In panel-A we show the trial-averaged value of A_1 associated with the case-specific bicluster $B1$ as a function of $\varepsilon\sqrt{m}$ and $\log_M(m)$. In panel-B we show the maximum of the trial-averaged values of A_2, A_3 and A_4 associated with the other biclusters. While the presence of genetic-controls makes it more difficult for our algorithm to detect $B1$ (e.g., compare panel-A with Fig 42), our algorithm is still generally successful when $\log_{10}(\varepsilon\sqrt{m}) \lesssim 0$ and $\log_M(m) \gtrsim 0.5$.

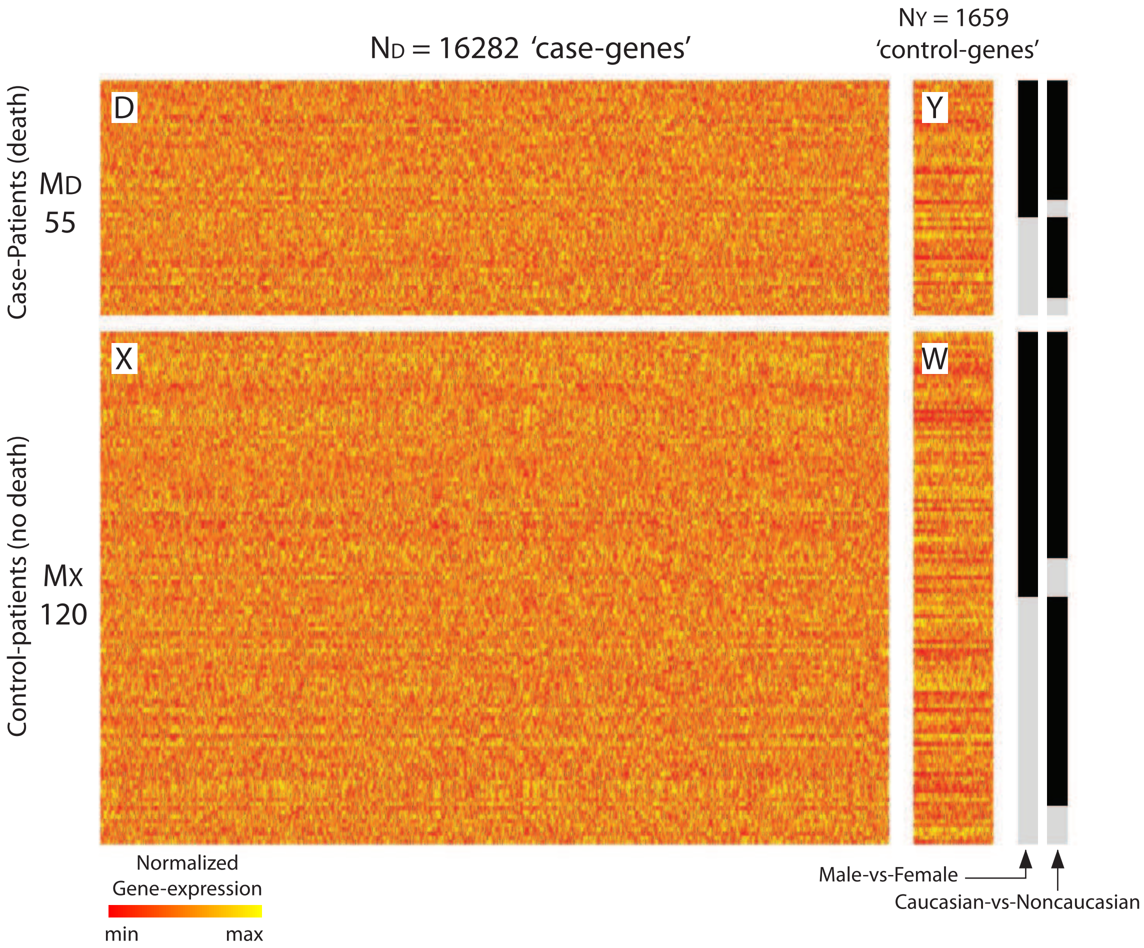

Figure 72: This figure illustrates the GSE17536 gene-expression data-set used in section 15.1. Each row corresponds to a patient, and each column to a 'gene' (i.e., gene-expression measurement): the color of each pixel codes for the intensity of a particular measurement of a particular patient (see colorbar to the bottom). We divide the 175 patients into $M_D = 55$ case-patients and $M_X = 120$ control-patients; colorectal cancer was determined to be the significant cause of death for the former, but not the latter. We also divide the 17941 genes into $N_D = 16286$ case-genes and $N_Y = 1659$ control-genes; the control-genes are those that are significantly co-expressed alongside genes MLH1 MSH2 and MSH6, whereas the case-genes are the remainder. We use these patient- and gene-classifications to delineate the case-matrix D, as well as the control-matrices X, Y and W. The covariates (gender and ethnicity) for each patient are shown to the far right, (i.e., grey vs black).

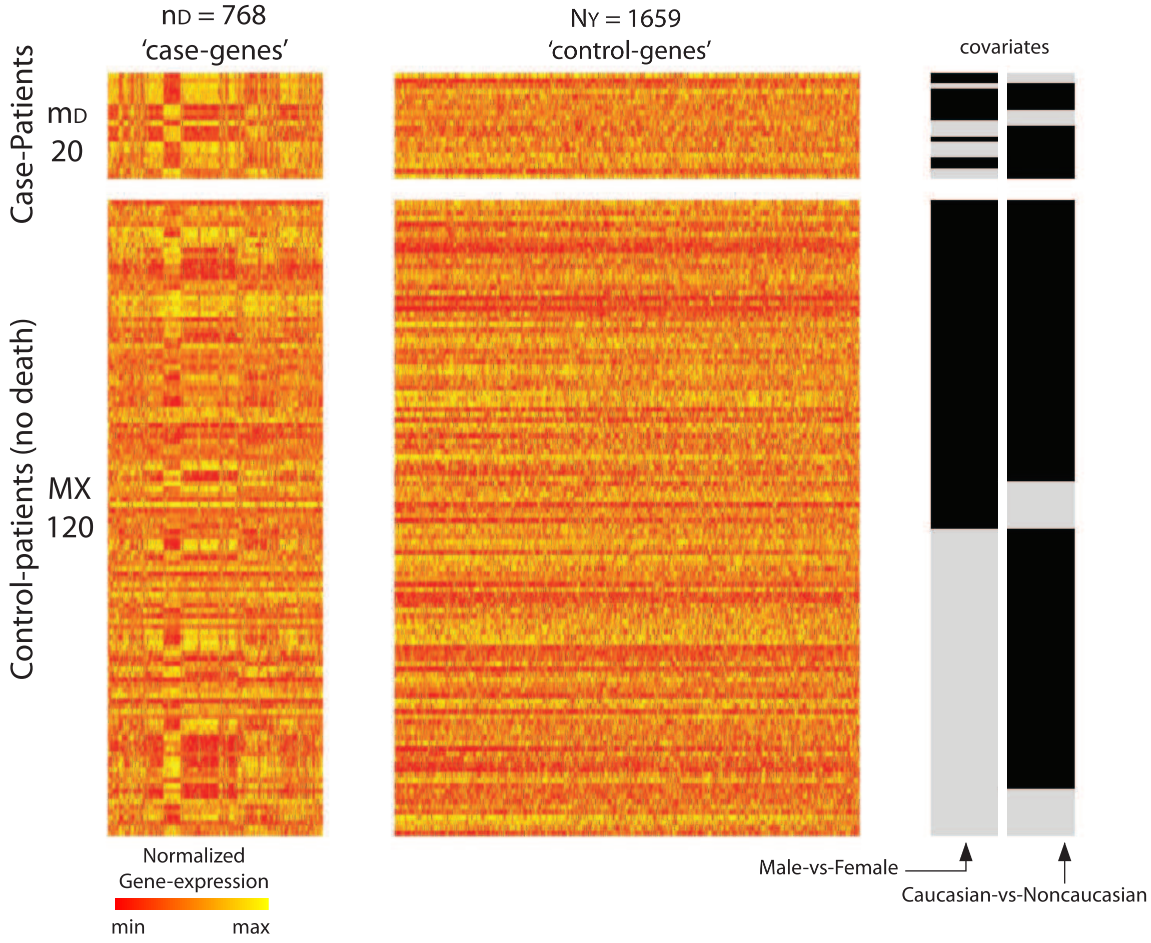

Figure 73: This figure illustrates the bicluster discovered after applying the loop-counting algorithm described in section 15.1 to the data-set shown in Fig 72. We've shown only the m_D case-patients and n_D genes involved in the bicluster, listed in the order given by the output of the algorithm (the patients and genes placed in the upper-left corner are those that have been retained the longest).

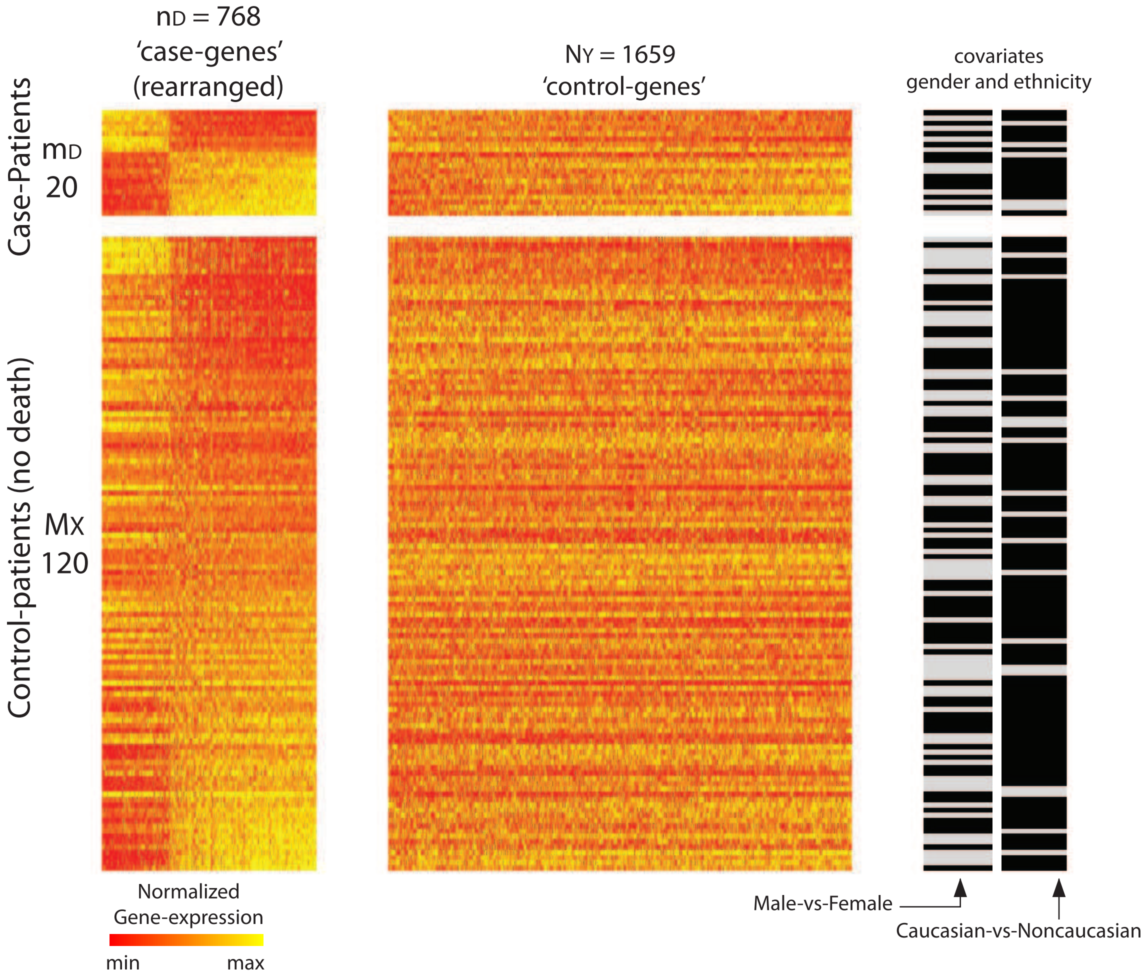

Figure 74: This figure illustrates the same bicluster shown in Fig 73, except that we've rearranged the rows and columns to highlight the co-expression pattern more clearly. The control-patients (and control-genes) are also rearranged in accordance with the dominant right (and left, respectively) singular vectors of the bicluster. As can be seen, there is a striking pattern of correlation across the 20 case-patients and 768 case-genes within the bicluster. Moreover, this co-expression pattern is not exhibited across a comparable fraction of the control-patients or control-genes.

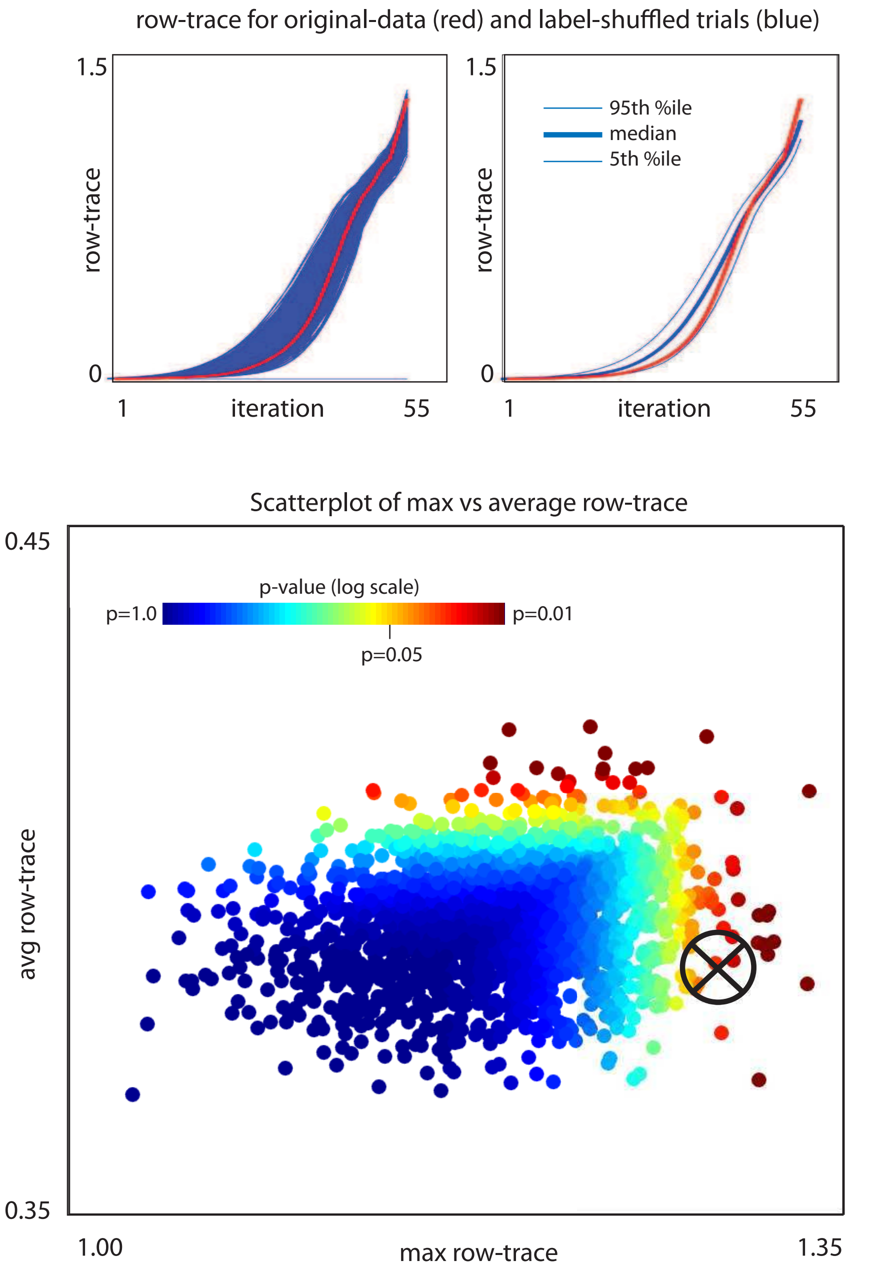

Figure 75: On top we show row-traces associated with the bicluster shown in Fig 74. On the left we show the row-trace as a function of iteration for the original-data (red) as well as each of the 2048 random shuffles (blue) generated under the null hypothesis. On the right we replot this same trace data, showing the 5th, 50th and 95th percentile (across iterations) of the shuffled-distribution. On the bottom we show a scatterplot of maximum- and average-row-traces (taken across the iterations) for both the original data and the shuffled-trials. Each row-trace from the graph above is plotted as a single point in 2-dimensional space; the horizontal-axis corresponds to the maximum row-trace and the vertical-axis corresponds to the average row-trace (taken across the iterations). The original-data is indicated with a '$\otimes$', and each of the random shuffles with a colored '$\bullet$'. The color of each '$\bullet$' indicates the p-value associated with that position in x,y-space. By comparing the original-trace with the shuffled-distribution we can read off a p-value of 0.021 for the bicluster drawn from the original-data.

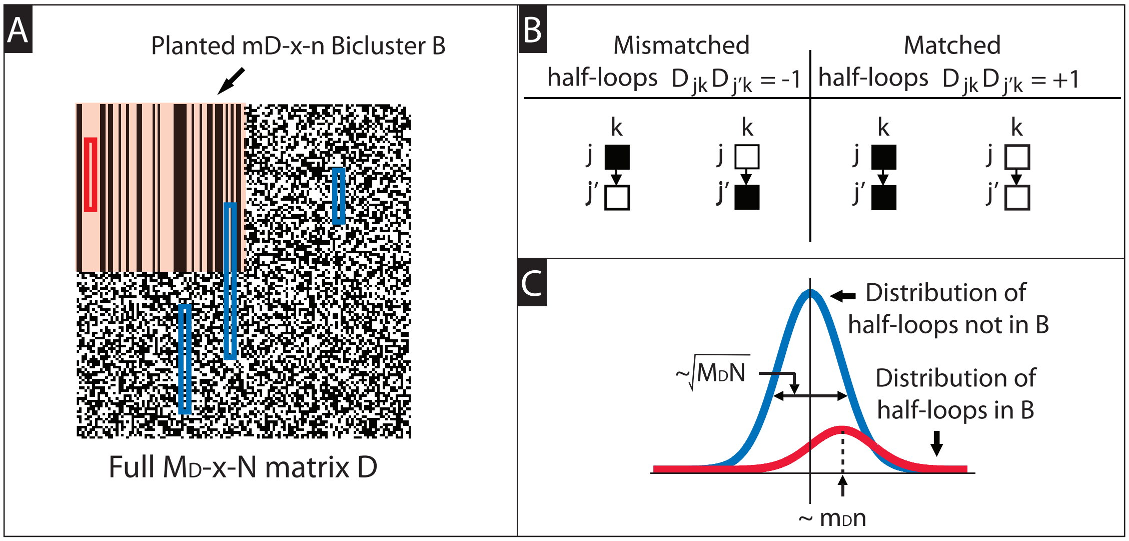

Figure 76: Detecting a rank-0 bicluster within a case-matrix alone. In Panel-A we show a large $M \times N$ binarized matrix D (black and white pixels correspond to values of ± 1, respectively). In the upper left corner of D we've inserted a large rank-0 bicluster B (shaded in pink). Our algorithm considers all 2×1 submatrices (i.e., 'half-loops') within D. Several such half-loops are highlighted via the thin blue rectangles (the top and bottom of each rectangle pick out a 2×1 submatrix). Generally speaking, the entries of a half-loop are equally likely be matched or mismatched. Some half-loops, such as the half-loop shown in red, are entirely contained within B. These half-loops are more likely to be matched than mismatched. In Panel-B we show some examples of mismatched and matched half-loops. Given a half-loop with row-indices j, j' and column-index k, the parity of the half-loop is determined by the sign of $D_{jk} D^{\intercal}_{kj'}$. Our algorithm accumulates a 'half-loop-score' for each row j and each column k. The half-loop-score for a particular row j is given by $\sum_{j',k} D_{jk} D^{\intercal}_{kj'} = [DD^{\intercal}1]_j$. The half-loop-score for a column k is given by $[1^{\intercal}D]_k \cdot [D^{\intercal}1]_k$. In Panel-C we show the distribution of half-loop-scores we might expect from the rows within D. The blue-curve corresponds to the distribution of scores expected from the rows/cols of D that are not in B, whereas the red-curve corresponds to the distribution of scores expected from the rows/cols of B.

and redefine the (2-sided) covariate-dependent sub-scores for each $t = 1, \ldots, N_T$ as follows:

$$\left[Z_{\text{ROW}}^{Q,Q';\, i,i';\, [t]}\right]_j = \widetilde{\sum_{\substack{Q,\ i,\ Q',\ i' \text{ fixed };\\ j \text{ fixed in } Qi,\ j' \text{ in } Q'i';\\ j' \neq j \text{ if } Q=Q' \text{ and } i=i'\\ k\in Q}}} [T_{Qi}]_{j,t} \left[Qi - 1_{Qi}\alpha^{\mathsf{T}}\right]_{j,k} \Delta_{kk} \left[Q'i' - 1_{Q'i'}\alpha^{\mathsf{T}}\right]_{j',k} [T_{Q'i'}]_{j',t} \,,$$

$$\left[Z_{\text{COL}}^{Q,Q';\, i,i';\, [t]}\right]_k = \widetilde{\sum_{\substack{Q,\ i,\ Q',\ i' \text{ fixed };\\ j \text{ in } Qi,\ j' \text{ in } Q'i';\\ j' \neq j \text{ if } Q=Q' \text{ and } i=i'\\ k \text{ fixed,}}}} [T_{Qi}]_{j,t} \left[Qi - 1_{Qi}\alpha^{\mathsf{T}}\right]_{j,k} \Delta_{kk} \left[Q'i' - 1_{Q'i'}\alpha^{\mathsf{T}}\right]_{j',k} [T_{Q'i'}]_{j',t} \,.$$

After substituting these new definitions, the subsequent combination of sub-scores remains unchanged.

We pause to remark that we can also redefine our 1-sided covariate-dependent sub-scores as follows:

$$\left[Z_{\text{ROW}}^{Q,Q';\, i,i';\, [t]\ 1\text{s}}\right]_j = \widetilde{\sum_{\substack{Q,\ i,\ Q',\ i' \text{ fixed };\\ j \text{ fixed in } Qi,\ j' \text{ in } Q'i';\\ j' \neq j \text{ if } Q=Q' \text{ and } i=i'\\ k\in Q}}} \left[Qi - 1_{Qi}\alpha^{\mathsf{T}}\right]_{j,k} \Delta_{kk} \left[Q'i' - 1_{Q'i'}\alpha^{\mathsf{T}}\right]_{j',k} [T_{Q'i'}]_{j',t} \,,$$

$$\left[Z_{\text{COL}}^{Q,Q';\, i,i';\, [t]\ 1\text{s}}\right]_k = \widetilde{\sum_{\substack{Q,\ i,\ Q',\ i' \text{ fixed };\\ j \text{ in } Qi,\ j' \text{ in } Q'i';\\ j' \neq j \text{ if } Q=Q' \text{ and } i=i'\\ k \text{ fixed,}}}} \left[Qi - 1_{Qi}\alpha^{\mathsf{T}}\right]_{j,k} \Delta_{kk} \left[Q'i' - 1_{Q'i'}\alpha^{\mathsf{T}}\right]_{j',k} [T_{Q'i'}]_{j',t} \,.$$

After this redefinition, we use the same subsequent combination of sub-scores as discussed in section 12 (i.e., the combination appropriate for a 1-sided covariate-correction).

One advantage to the half-loop algorithms described above is that they are very easy to analyze. In fact, much of our previous analysis remains unchanged; the main difference being that we replace the probability $g_{l,\varepsilon,m}$ with the new probability $\hat{g}_{\varepsilon}$. Recall that we previously defined the former to be the probability that a loop drawn from a low-rank submatrix is rank-l, whereas we now define the latter to be the probability that a half-loop drawn from a rank-0 submatrix is of matched sign. While $g_{l,\varepsilon,m}$ depends on both the noise-level ε as well as the submatrix size m, the probability $\hat{g}_{\varepsilon}$ only depends on the level of noise, and not on the size m. To generate a rank-0 submatrix with noise level ε, we draw each entry independently and randomly to be either ± 1 with a probability chosen so that $g_{1,\varepsilon,m}$ equals $\hat{g}_{\varepsilon}$.

A second advantage to the half-loop algorithms is that they can be used to search for differentially-expressed biclusters where each gene is either high across the cases and low across the controls, or vice-versa. These structures are – by definition – rank-1 structures that extend across both the case- and control-populations; they were deliberately ignored by the control-correction described in section 8, but can easily be found using the half-loop algorithms above.

A simple example illustrating this approach is shown in Figs 77 and 78. A more complicated example is shown in Figs 79 and 80. These two examples are structured almost identically to the numerical experiments described in Figs 24 and 54, the only difference being that we implant rank-0 biclusters, rather than merely low-rank biclusters. As these examples illustrate, our half-loop algorithm is not quite as good as our full loop-counting algorithm. Nevertheless, it is an order of magnitude faster, a feature that may be of interest when analyzing large data-sets.

When we actually carry out our half-loop method on the data from section 1.4, we do indeed find a rank-0 bicluster, shown in Figs 81-85. This rank-0 bicluster is strongly enriched for many different pathways, the most prominent of which include: neurogenesis (p=1.6e-9), neuron-differentiation (p=5.2e-9), axon-guidance (p=3.4e-6), enzyme-linked receptor-protein signaling pathway (p=6e-5), ion-channel-binding (1.6e-4), peptidyl-tyrosine physphorylation (5e-4) and ephrin-receptor-activity (8e-4). As in the example shown in section 1.4, this bicluster was found within the neurotypical patients, and may highlight genes that play a protective role with regards to the psychiatric disorder associated with this data-set.

15.3 Searching for triclusters:

There are often situations where the data-paradigm we've assumed – involving N measurements taken across M patients – doesn't suffice. For example, in a clinical study there may be M patients, each of which are subjected to P different kinds of treatments (e.g., therapy regimes, medications, etc). For each of these P treatments, N variables may be measured for each patient. Within this paradigm, the data isn't best represented as a 2-dimensional array (e.g., a matrix), but rather as a 3-dimensional array (i.e., a box or a cube) comprising both rows and columns, as well as 'layers'. In formal terms, we

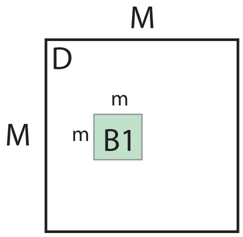

Figure 77: This figure illustrates the setup for the numerical experiments shown in Fig 78. The setup for these experiments is almost identical to the setup shown in Fig 24, with the exception that the planted bicluster is not merely low-rank, but rather rank-0. A rank-0 bicluster is characterized by its size, as well as by the probability $\hat{g}_\varepsilon$ that a half-loop drawn from the bicluster will be of matched sign. We generate our rank-0 biclusters by choosing each entry independently and randomly so that the probability $\hat{g}_\varepsilon$ is equal to the $g_{1,\varepsilon,m}$ we would associate with a rank-1 biclster.

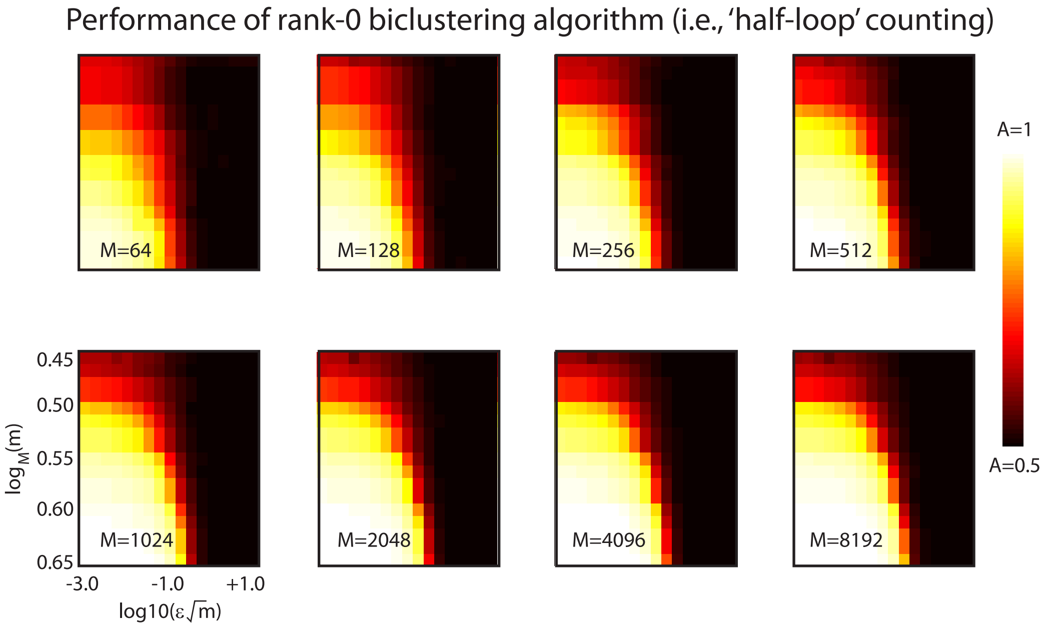

Figure 78: This figure illustrates the numerical experiments described in Fig 77. We show the trial-averaged value of A as a function of $\varepsilon\sqrt{m}$ and $\log_M(m)$. Note that, while our half-loop algorithm does indeed detect the bicluster when $\varepsilon\sqrt{m} \lesssim 1/10$ and $m \gtrsim \sqrt{M}$, it does not do as good a job as our full loop-counting algorithm (compare with Fig 26).

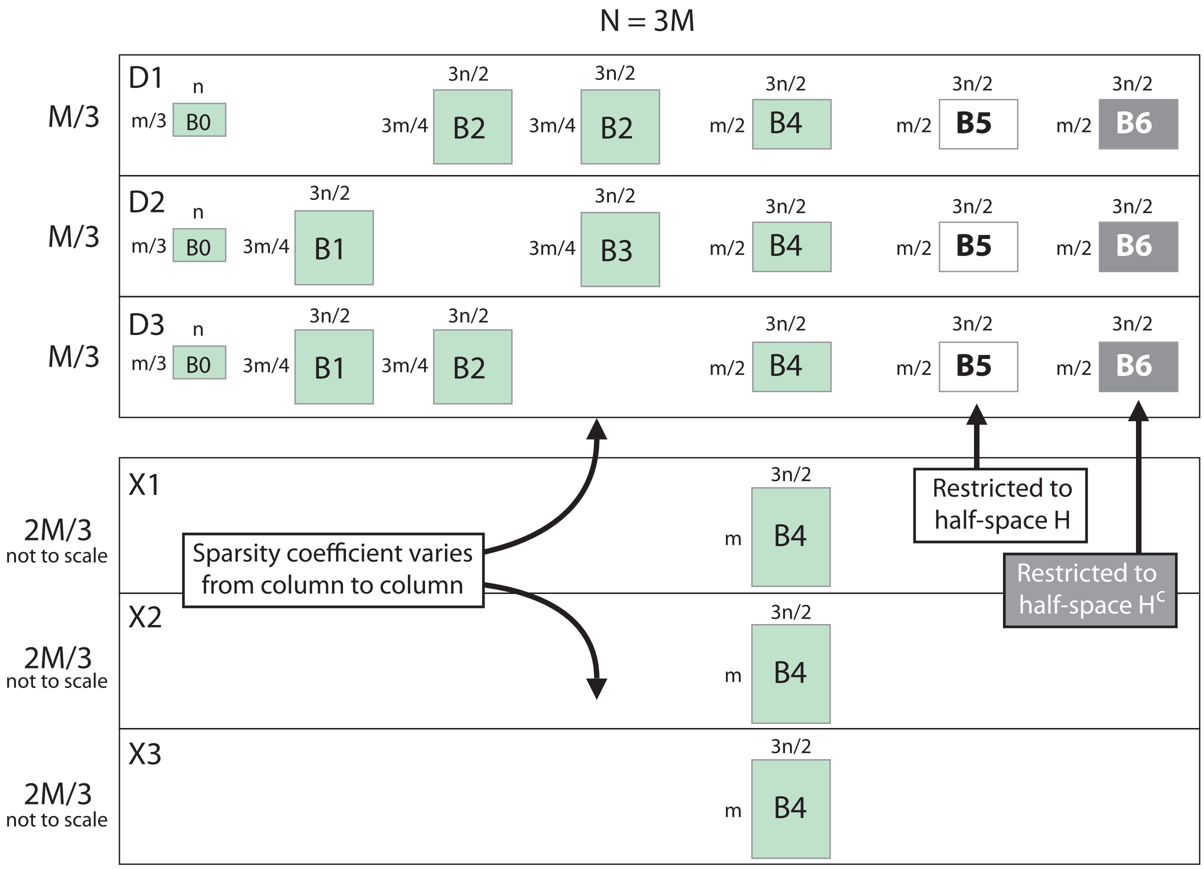

Figure 79: This figure illustrates the setup for the numerical experiments shown in Fig 80. The setup for these experiments is almost identical to the setup shown in Fig 54, with the exception that all the planted biclusters are not merely low-rank, but rather rank-0. As in Fig 78, we generate our rank-0 biclusters by choosing each entry independently and randomly so that the probability $\hat{g}_{\varepsilon}$ is equal to the $g_{1,\varepsilon,m}$ we would associate with a rank-1 biclster.

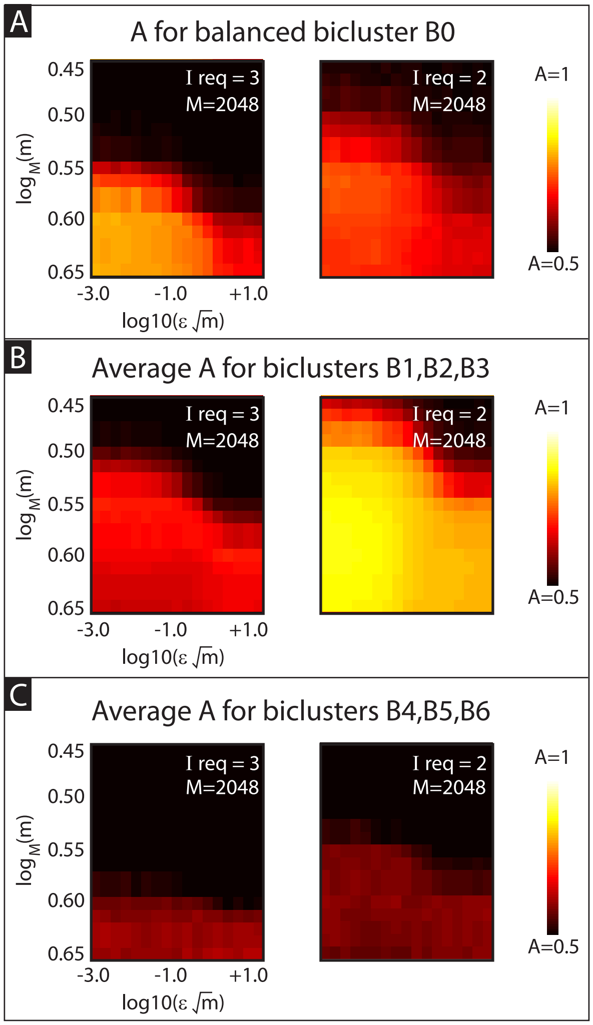

Figure 80: This figure illustrates the numerical experiments described in Fig 79. In panel-A we show the trial-averaged value of A_0 as a function of $\varepsilon\sqrt{m}$ and $\log_M(m)$. In panel-B we show the trial-averaged value of A_1, A_2, A_3. In panel-C we show the trial-averaged value of A_4, A_5, A_6. The left side of each panel displays results with $I_{\text{req}} = 3$, the right side with $I_{\text{req}} = 2$. Note that, when $I_{\text{req}} = 3$ the goal was to find $B0$, as this was the only case-specific bicluster that was balanced across all the continuous-covariates and covariate-categories. On the other hand, when $I_{\text{req}} = 2$ the goal was to find the most dominant of $B0$ $B1$ $B2$ and $B3$, as each of these case-specific biclusters were balanced across all the continuous-covariates while extending across at least 2 covariate-categories. Note that our half-loop algorithm does a modest job of achieving these goal, even in the presence of the other biclusters $B4$ $B5$ and $B6$. Note also that this half-loop algorithm is not quite as good as our original loop-counting algorithm (compare with Fig 55).

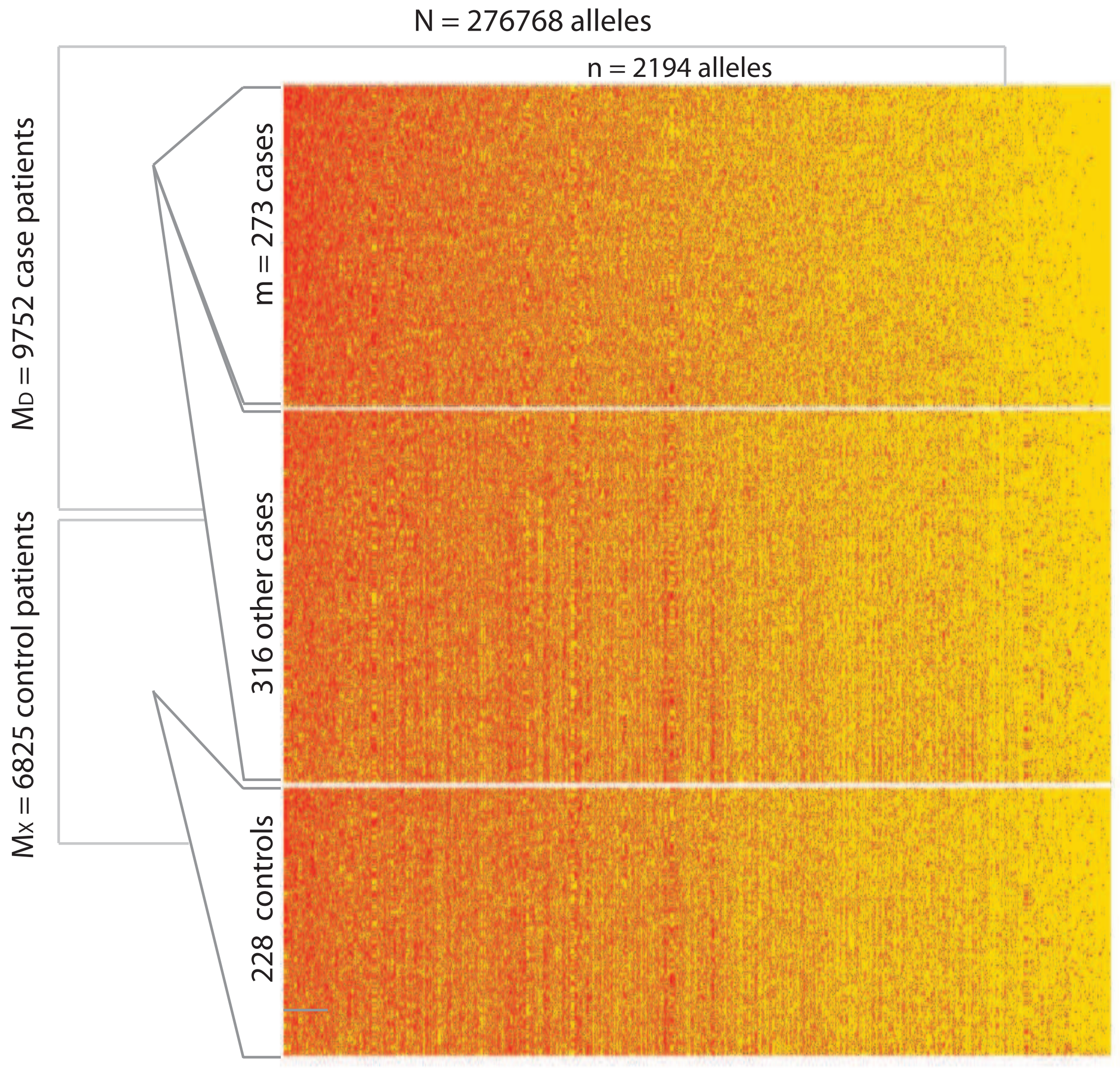

Figure 81: In this figure we illustrate the rank-0 bicluster found within the GWAS data-set discussed in Example-3 (see sections 1.4 and 15.2). This figure has the same format as Fig 11; the bicluster is shown on in the topmost portion of the inset, while a random selection of other case-patients and control-patients are shown below. While the distribution of genetic-data within this bicluster does not appear to be all that different from the other patients, the bicluster is indeed quite different from the remaining patients. See Fig 82 for more details.

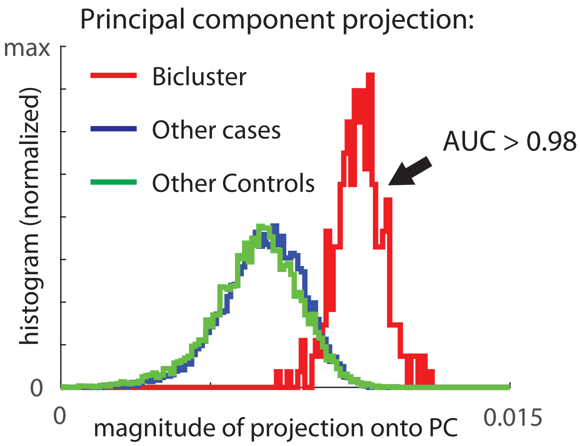

Figure 82: The bicluster shown in the top of Fig 81 was not obviously distinct from the remaining cases and controls. However, the structure within this bicluster can be revealed by first calculating the dominant right principal component of the bicluster, and then projecting each patient's associated genetic information onto that principal component. In this figure we show histograms collecting the magnitude of this projection. While the absolute magnitude of the projection is not very large for any of the patients (which is why the bicluster was not visually distinct from the remaining patients), the actual values of the projection are quite distinct – the patients within the bicluster are indeed well-separated from the other patients. We can quantify this separation by calculating the Auc associated with the projected values, yielding in this case an Auc of $A > 0.98$.

imagine our data arranged into an array D of dimension $M \times N \times P$, where $D_{j,k,l}$ corresponds to the kth measurement of the jth-patient as they undergo therapy l.

Within this 3-dimensional array, it is often prudent to search for subsets of patients that exhibit some kind of simple structure across a subset of measurements as well as a subset of treatments. Such a 'tricluster' would correspond to a 'sub-cube' of the data, rather than simply a submatrix. The techniques we discussed earlier can readily be extended to search for these kinds of objects as well.

For exposition, let's consider the 'planted-tricluster' problem. We'll assume that the data-array D contains a hidden $m \times n \times p$ sub-array B involving patient-subset J_B, measurement-subset K_B, and treatment-subset L_B (i.e., J_B, K_B and L_B are unknown). The sub-array B will exhibit structure not exhibited by the rest of D, and our goal will be to locate the tricluster B (i.e,. to find the row- column- and layer-subsets J_B, K_B and L_B).

We'll assume that each entry of D that is not in B is drawn independently and randomly from, say, a median-0 distribution, but that the entries of B are drawn from a more structured distribution. For simplicity let's assume B is formed from a collection of outer-products; one for each pair of array-dimensions:

$$B_{j,k,l} = u_j^1 v_k^1 + u_j^2 w_l^2 + v_k^3 w_l^3,$$

where $\vec{u}^1$, $\vec{u}^2 \in \mathbb{R}^m$, and $\vec{v}^1$, $\vec{v}^3 \in \mathbb{R}^n$, and $\vec{w}^2$, $\vec{w}^3 \in \mathbb{R}^p$. are each randomly chosen (but unknown) vectors that describe the low-rank structure within B.

As before, we'll binarize D, sending each entry to either $+1$ or -1, depending on its sign. Once we binarize D, we can calculate the following scores:

$$\begin{aligned}
[Z_{\text{ROW}}]_j &= \widetilde{\sum_{\substack{j \text{ fixed},\ j' \neq j; \\ k' \neq k\ ;\ l}}} D_{jkl} D_{j'kl} D_{j'k'l} D_{jk'l} + \widetilde{\sum_{\substack{j \text{ fixed},\ j' \neq j; \\ k\ ;\ l' \neq l}}} D_{jkl} D_{j'kl} D_{j'kl'} D_{jkl'}, \\
[Z_{\text{COL}}]_k &= \widetilde{\sum_{\substack{k \text{ fixed},\ k' \neq k; \\ j' \neq j\ ;\ l}}} D_{jkl} D_{jk'l} D_{j'k'l} D_{j'kl} + \widetilde{\sum_{\substack{k \text{ fixed},\ k' \neq k; \\ j\ ;\ l' \neq l}}} D_{jkl} D_{jk'l} D_{jk'l'} D_{jkl'}, \\
[Z_{\text{LYR}}]_l &= \widetilde{\sum_{\substack{l \text{ fixed},\ l' \neq l; \\ j' \neq j\ ;\ k}}} D_{jkl} D_{jkl'} D_{j'kl'} D_{j'kl} + \widetilde{\sum_{\substack{l \text{ fixed},\ l' \neq l; \\ j\ ;\ k' \neq k}}} D_{jkl} D_{jkl'} D_{jk'l'} D_{jk'l},
\end{aligned}$$

corresponding to the row-, column- and layer-scores, respectively. Once we've calculated these scores, we can remove the rows, columns and layers with low scores, and repeat the entire process. As before, this process will focus on the rows, columns and layers of B with high probability as long as $m \gtrsim \sqrt{M}$, $n \gtrsim \sqrt{N}$ and $p \gtrsim \sqrt{P}$.

The reason this process works is that – as before – the scores accumulate the ranks of the various loops within D. However, unlike the simpler situation discussed in section 2, D is a 3-dimensional array (and not merely a matrix).

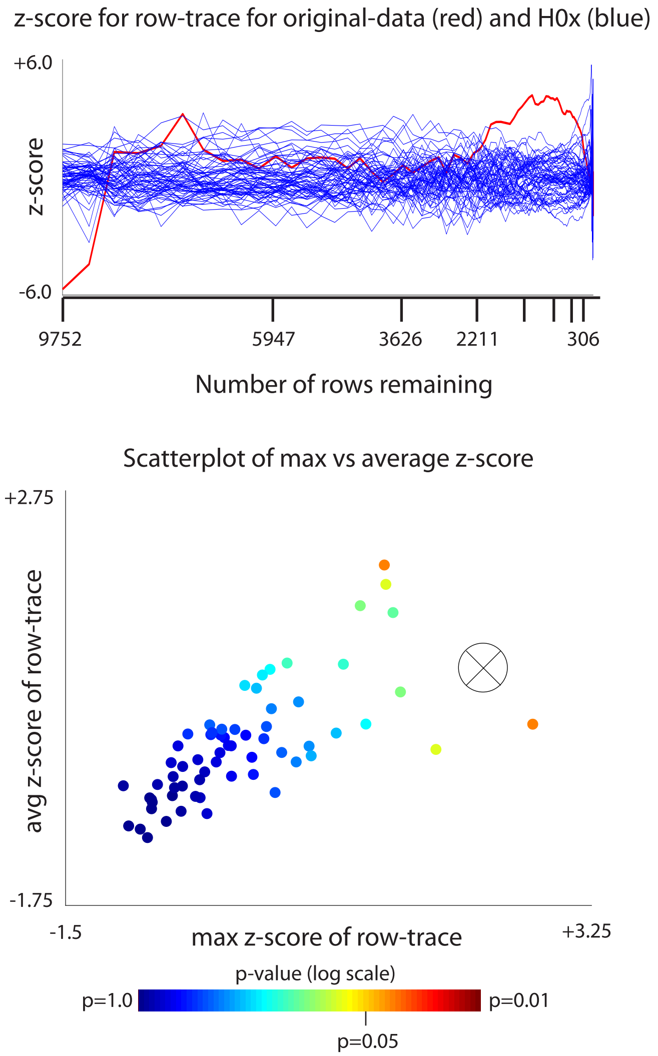

Figure 83: The bicluster shown in Fig 81 is significant with respect to our label-shuffled hypothesis H0x. This figure has the same format as Fig 63, and shows Z-scores for the bicluster shown in Fig 81. This bicluster is significant with respect to our label-shuffled hypothesis H0x, yielding a p-value of $\lesssim 0.03$.

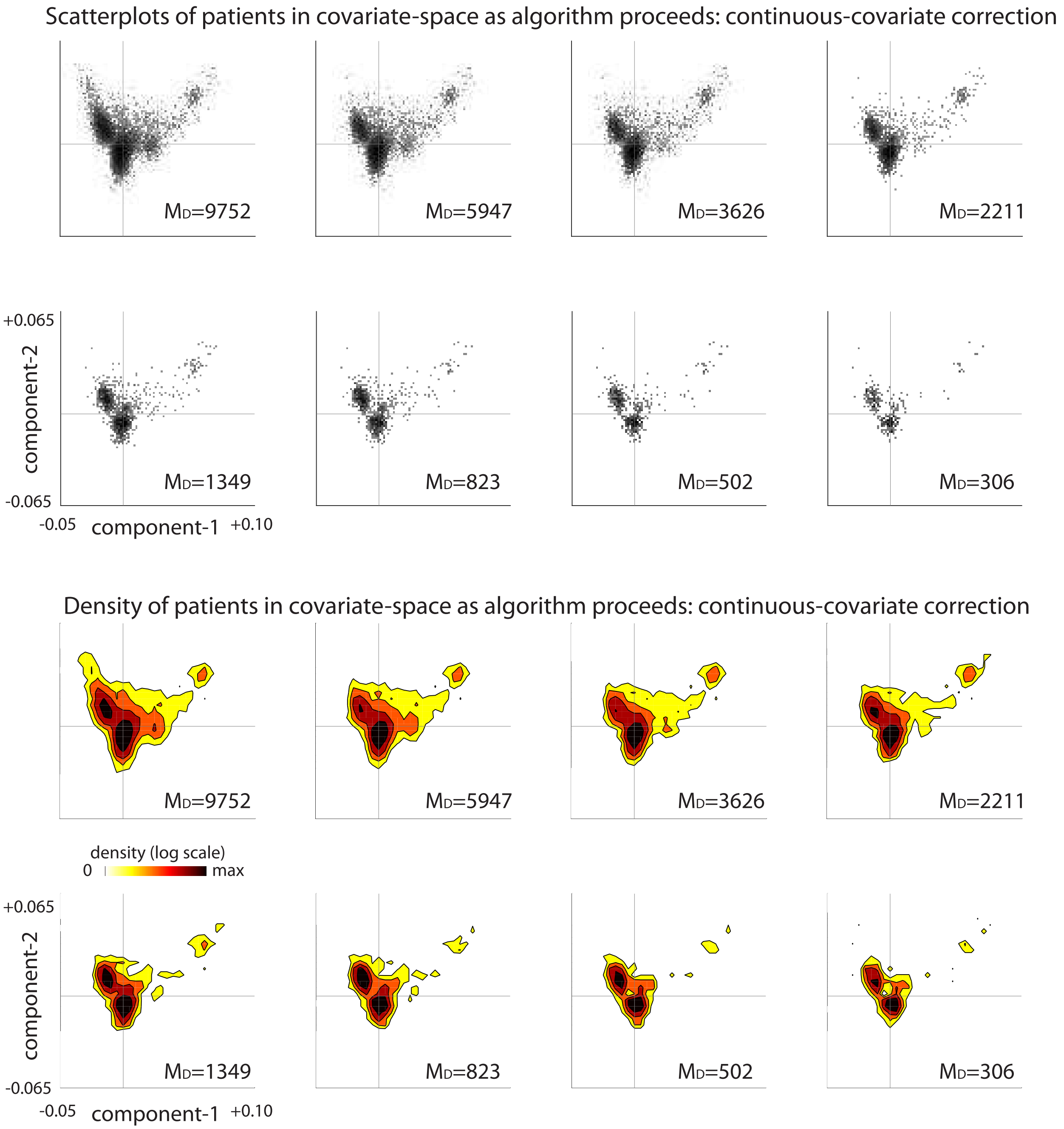

Figure 84: This figure has the same format as Fig 14, and shows the distribution of continuous-covariate components across the remaining case-patients as our algorithm proceeds. As before, our algorithm ensures that the covariate-distribution remains relatively well-balanced.

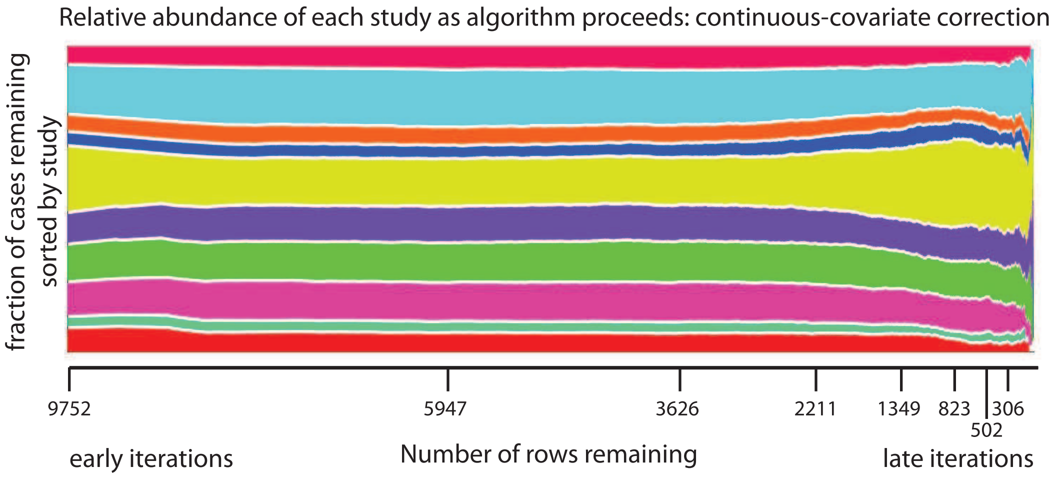

Figure 85: This figure has the same format as Fig 18, and shows the relative abundance of each study as our algorithm proceeds. As before, our algorithm is able to ensure that the remaining case-patients are drawn from a reasonable mixture of the studies.

Consequently, there are 3 different kinds of loops within D, each traversing a different pair of array-dimensions (see Fig 86). Any loop within D that does not lie entirely within B is just as likely to be rank-1 as it is to be rank-2. On the other hand, loops within D that are entirely contained within B are more likely to be rank-1 than rank-2. This probability is not 100%, but it is still significantly greater than 50%, with the exact value dependent on how the $\vec{u}^1, \vec{u}^2, \ldots, \vec{w}^3$ are drawn. Moreover, this probability is still significantly greater than 50% even in the presence of a moderate amount of noise (e.g., if B were not exactly a sum of outer products).

A numerical experiment corroborating this intuition is shown in Fig 87. Techniques along these lines have been used to find triclusters in clinical data involving several patients, measurements and therapies. See [$\mathcal{M}$44] for some preliminary results.

16 Various properties of multivariate gaussians

This section serves as somewhat of an appendix to section 2, collecting various observations about gaussian distributions.

16.1 one-dimensional gaussian:

We use $\rho_\sigma(x)$ to refer to the normal distribution $N\left(0,\sigma^2\right)$ on x:

$$\rho_\sigma(x) = \frac{1}{\sqrt{2\pi}\sigma}\exp\left(\frac{-x^2}{2\sigma^2}\right), \text{ with mean 0 and variance } \sigma^2.$$

Note that the gaussian distribution ρ_σ satisfies:

$$\frac{d}{dx}\rho_\sigma(x) = -\frac{x}{\sigma^2}\rho_\sigma(x), \text{ implying}$$

$$\int_{-\infty}^{+\infty} x^{k+2}\rho_\sigma(x)\,dx = -\sigma^2\int_{-\infty}^{+\infty} x^{k+1}\left(\frac{d}{dx}\rho_\sigma\right)dx = (k+1)\,\sigma^2\int_{-\infty}^{+\infty} x^k\rho_\sigma(x)\,dx.$$

Consequently, the first few moments of ρ_σ are given by:

$$\int d\rho_\sigma = 1,\ \int x d\rho_\sigma = 0,\ \int x^2 d\rho_\sigma = \sigma^2,\ \int x^3 d\rho_\sigma = 0,\ \int x^4 d\rho_\sigma = 3\sigma^4,$$

implying that, if x is drawn from $\rho_\sigma(x)$, then the mean of x^2 will be σ^2 and the variance of x^2 will be $\left(3\sigma^4 - \sigma^4\right) = 2\sigma^4$.

One can also show that the 1-dimensional fourier-transform F of a 1-d gaussian is another 1-d gaussian:

$$F\{\rho_\sigma\}(k) = \frac{1}{\sqrt{2\pi}}\int_{-\infty}^{+\infty} e^{-ikx}\rho_\sigma(x)\,dx = \frac{1}{\sigma}\rho_{1/\sigma}(k).$$

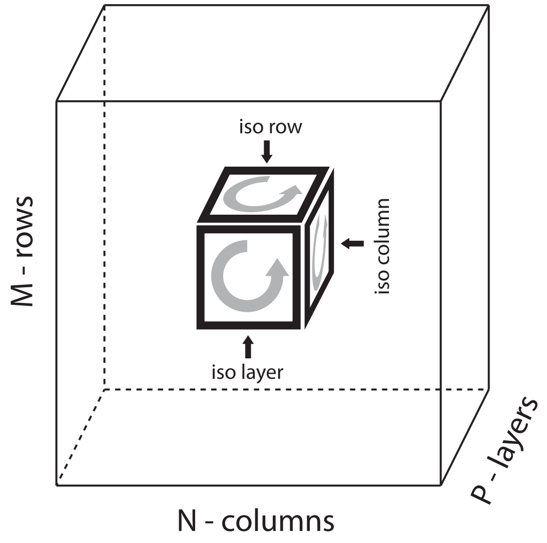

Figure 86: Illustration of the loops within a 3-dimensional array. We sketch the structure of a 3-dimensional data-array D, with J rows, K columns and P 'layers'. Each entry $D_{j,k,l}$ will lie in the cube shown. The loops within D can be divided into 3-categories: (a) iso-layer loops that stretch across 2 rows and 2 columns, (b) iso-column loops that stretch across 2 rows and 2 layers, and (c) iso-row loops that stretch across 2 columns and 2 layers. The row-score $[Z_{\text{ROW}}]_j$ aggregates all the iso-column and iso-layer loops associated with row-j. The column-score $[Z_{\text{COL}}]_k$ aggregates all the iso-row and iso-layer loops associated with column-k. The layer-score $[Z_{\text{LYR}}]_l$ aggregates all the iso-row and iso-column loops associated with layer-l.

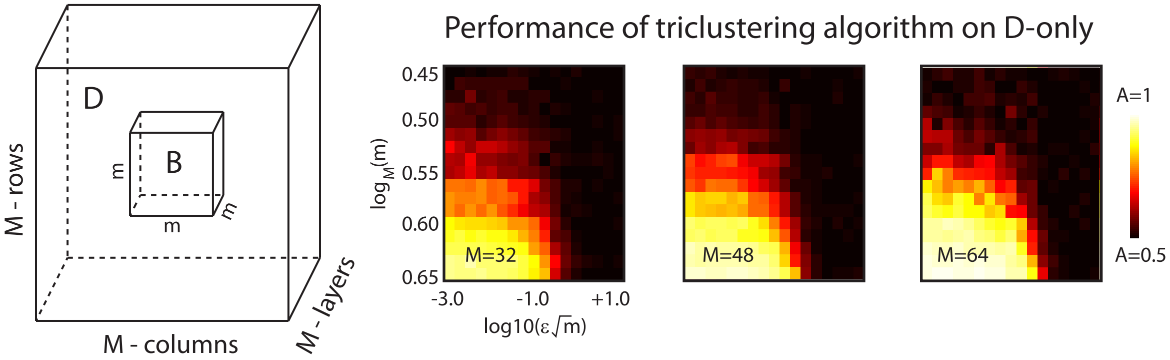

Figure 87: This numerical experiment involves a 'planted-tricluster' problem, where we implant an $m \times m \times m$ tricluster B within an $M \times M \times M$ array D. The setup of this experiment is shown to the far left. Each entry of D is chosen independently and randomly from a distribution with median 0. The tricluster B is constructed by first forming a sum of three outer products: $B_{j,k,l} = u_j^1 v_k^1 + u_j^2 w_l^2 + v_k^3 w_l^3$, and then flipping the sign of a randomly-chosen fraction f of the entries of B. We choose the sign-flipping probability f as described in section 5.1 (i.e., f depends on $\varepsilon\sqrt{m}$). Our loop-counting algorithm produces a list of row-, column- and layer-indices of D in the order in which they are eliminated; those indices retained the longest are expected to be members of B. For each numerical experiment we calculate the auc A_R (i.e., area under the receiver operator characteristic curve) associated with the row-indices of B with respect to the output list from our algorithm. We calculate the auc A_C and A_L for the columns and layers similarly. Finally, we use $A = (A_R + A_C + A_L)/3$ as a metric of success; values of A near 1 mean that the rows, columns and layers of B were retained the longest by our algorithm. Values of A near 0.5 mean that our algorithm failed to detect B. On the right-hand side we show the trial-averaged value of A as a function of $\varepsilon\sqrt{m}$ and $\log_M(m)$ for a few values of M. Note that our loop-counting algorithm is generally successful when $\varepsilon\sqrt{m}$ is sufficiently small and m is sufficiently large.

16.2 convolution of two one-dimensional gaussians:

It is well known that the convolution of two one-dimensional gaussians is yet another gaussian:

$$[\rho_\alpha \star \rho_\beta](x) = \int_{-\infty}^{\infty} \rho_\alpha(x-y)\,\rho_\beta(y)\,dy = \rho_\gamma(x)\,,\ \text{where } \alpha^2+\beta^2=\gamma^2.$$

This can be understood by noting the fourier-transform above and appealing to the convolution theorem (i.e., $F\{\rho_\alpha \star \rho_\beta\} = F\{\rho_\alpha\} F\{\rho_\beta\}$). This can also be understood by considering a random vector x^N with N independently chosen entries, each either -1 or $+1$. Obviously, any particular entry of x^N has mean 0 and variance 1. Therefore the sum $S_N = \sum_{n=1}^{n=N} x_n^N$ is a random variable with mean 0 and variance N; when N is large S_N will be drawn from $\rho_{\sqrt{N}}$. Now, considering a situation where $\alpha^2+\beta^2=\gamma^2$, we immediately see that S_{γ^2} must be drawn from ρ_γ, whereas S_{α^2} and S_{β^2} are drawn from ρ_α and ρ_β respectively. Given that there is a one-to-one mapping between vectors x^{γ^2} and vectors $x^{\alpha^2} \oplus x^{\beta^2}$, the random variables S_{γ^2} and $S_{\alpha^2}+S_{\beta^2}$ must have the same distribution. Consequently, ρ_γ must be equal to $\rho_\alpha \star \rho_\beta$.

16.3 two-dimensional gaussian:

We also use $\rho_{a,b,\theta}(\vec{x})$ to refer to the mean 0 anisotropic multivariate gaussian distribution in 2-dimensions with variances a^2 and b^2, and first principal-component oriented at angle θ. That is to say:

$$\rho_{a,b,\theta}(\vec{x}) = \frac{1}{2\pi ab}\exp\left(-\frac{1}{2}\vec{x}^\intercal R_\theta \begin{bmatrix} \frac{1}{a^2} & \\ & \frac{1}{b^2} \end{bmatrix} R_\theta^\intercal \vec{x}\right),$$

$$\text{where } R_\theta = \begin{bmatrix} \cos\theta & -\sin\theta \\ +\sin\theta & \cos\theta \end{bmatrix}.$$

In terms of visualization, the contours of $\rho_{a,b,\theta}$ are ellipses with one principal axis pointing in the θ-direction, and the other principal axis perpendicular to the θ-direction; these elliptical contours will have principal radii proportional to a and b, respectively.

Using our notation, we see that $\rho_{a,b,0}(\vec{x})$ is separable: $\rho_{a,b,0}(\vec{x}) = \rho_a(x_1)\,\rho_b(x_2)$. Thus, we can think of $\rho_{a,b,\theta}(\vec{x})$ as:

$$\rho_{a,b,\theta}(\vec{x}) = \rho_{a,b,0}(R_{-\theta}\cdot x) = \rho_a(x_1\cos\theta + x_2\sin\theta)\cdot\rho_b(-x_1\sin\theta + x_2\cos\theta)\,.$$

These observations can be easily used to show that the 2-dimensional fourier-transform F of a 2-d gaussian is yet another 2-d gaussian:

$$\begin{aligned} F[\rho_{a,b,\theta}(\vec{x})]\left(\vec{k}\right) &= \frac{1}{2\pi}\int \rho_{a,b,\theta}(\vec{x})\exp\left(-i\vec{k}^\intercal\cdot\vec{x}\right)dx_1dx_2 = F[\rho_{a,b,0}(\vec{x})]\left(R_{-\theta}\cdot\vec{k}\right) \\ &= \left[\frac{1}{a}\rho_{1/a}\otimes\frac{1}{b}\rho_{1/b}\right]\left(R_{-\theta}\cdot\vec{k}\right) = \frac{1}{ab}\rho_{1/a,1/b,0}\left(R_{-\theta}\cdot\vec{k}\right) \\ &= \frac{1}{ab}\rho_{1/a,1/b,\theta}\left(\vec{k}\right). \end{aligned}$$

Note that $\rho_{1/a,1/b,\theta}$ has the same orientation as $\rho_{a,b,\theta}$, but with inverted principal-values.

16.4 isotropic two-dimensional gaussian:

Note that $\rho_{\sigma,\sigma,\theta}$ is an isotropic gaussian distribution in 2-dimensions with variance σ, and does not depend on θ; the contours of $\rho_{\sigma,\sigma,\theta}$ are circles. A vector $[x_1,x_2]$ drawn from $\rho_{\sigma,\sigma,0}$ can be constructed via $\rho_{\sigma,\sigma,0}(\vec{x}) = \rho_\sigma(x_1)\,\rho_\sigma(x_2)$. Because $\rho_{\sigma,\sigma,0}$ is isotropic, the vector $[x_1,x_2]$ will have an orientation $\omega = \arctan(x_2/x_1)$ drawn uniformly from $[0,2\pi]$, and a magnitude $r=\sqrt{x_1^2+x_2^2}$ drawn from the Rayleigh distribution

$$\frac{r}{\sigma^2}\exp\left(\frac{-r^2}{2\sigma^2}\right) = \frac{\sqrt{2\pi}r}{\sigma}\rho_\sigma(r)\ \text{ on }\ r\in|0,\infty)\,,\text{ with mean } \sigma\sqrt{\pi/2} \text{ and variance } \sigma^2(2-\pi/2)\,.$$

16.5 restriction of two-dimensional gaussian to quadrants:

In general, given a gaussian $\rho_{a,b,\theta}$, the fraction p of this gaussian restricted to the first and third quadrants is

$$p(a,b,\theta) = \frac{1}{\pi}\,\text{arccot}\left(\left(\frac{b}{a}-\frac{a}{b}\right)\frac{1}{2}\sin 2\theta\right).$$

This can be determined by first observing that p is the integral of $\rho_{\alpha,b,\theta}$ over the domain Ω with boundaries given by the x and y axes, subtending an angle of π:

$$\Omega = \{(r,\theta) \,\|\theta \in [0,\pi/2] \text{ or } [\pi,3\pi/2]\}.$$

With this definition of Ω, we can write:

$$p(a,b,\theta) = \int_\Omega \rho_{a,b,\theta}(\vec{x})\, d\vec{x}.$$

Given this formulation, we can first rotate space (and Ω) by $-\theta$:

$$p(a,b,\theta) = \int_{R_\theta^\intercal \Omega} \rho_{a,b,\theta}(R_\theta \vec{x})\, d\vec{x} = \int_{R_\theta^\intercal \Omega} \rho_{a,b,0}(\vec{x})\, d\vec{x},$$

and then dilate space (and $R_\theta^\intercal \Omega$) by $\Sigma = \text{diag}(1/a, 1/b)$:

$$p(a,b,\theta) = \int_{\Sigma R_\theta^\intercal \Omega} \rho_{a,b,0}\left(\Sigma^{-1}\vec{x}\right) d\Sigma^{-1}\vec{x} = \int_{\Sigma R_\theta^\intercal \Omega} \rho_{1,1,0}(\vec{x})\, d\vec{x}.$$

Within this final formulation the integrand is a uniform isotropic gaussian $\rho_{1,1,0}$, and so $p(a,b,\theta)$ is simply $1/2\pi$ times the angle subtended by the transformed domain $\Omega' = \Sigma R_\theta^\intercal \Omega$. This angle in turn can be determined by applying $\Sigma R_\theta^\intercal$ to the boundaries of Ω (i.e., to the x and y axes), yielding the formula above.

16.6 approximation of orthonormal $\hat{u}, \hat{v}$:

If we randomly draw the numbers $u_1, \ldots, u_m$ and $v_1, \ldots, v_m$ independently from $\rho_{\sqrt{1/m}}$, then each u_j^2 and v_j^2 will be drawn from a distribution with mean $(1/m)$ and variance $\left(2/m^2\right)$. Thus, when m is large, the random variables $|u|^2$ and $|v|^2$ will be drawn from a gaussian-distribution with mean 1 and variance $2/m$. Each term $u_j v_j$, on the other hand, will be drawn from a gaussian distribution with mean 0 and variance $1/m^2$, implying that the random variable $u \cdot v$ is drawn from $\rho_{1/m}$. Thus, the unit vector $\hat{u} = u/|u|$ is likely within $O(1/\sqrt{m})$ of u. Because $\hat{u}$ and v are independent, the unit-vector $\tilde{v} = v - \hat{u}\hat{u}^\intercal v$ is likely within $O(1/\sqrt{m})$ of v, and will be orthogonal to $\hat{u}$. Similarly, the unit-vector $\hat{v} = \tilde{v}/|\tilde{v}|$ will also be orthogonal to $\hat{u}$ and within $O(1/\sqrt{m})$ of v. Given this construction, the unit vectors $\hat{u}$ and $\hat{v}$ will be orthonormal and uniformly distributed (conditioned on the fact that $\hat{u} \cdot \hat{v} = 0$). In other words, the original vectors u and v were close to orthonormal to begin with.

16.7 convolution of two two-dimensional gaussians:

Given random variables $\vec{x}$ and $\vec{y}$ drawn from $\rho_{a,b,\theta}$ and $\rho_{c,d,\phi}$, the sum $\vec{z} = \vec{x} + \vec{y}$ will be drawn from $\rho_{a,b,\theta} \star \rho_{c,d,\phi}$ (i.e., the distribution obtained by convolving $\rho_{a,b,\theta}$ and $\rho_{c,d,\theta}$) This distribution will also be a gaussian, of the form $\rho_{f,g,\omega}$, for some f, g, ω. This latter distribution can be understood via the fourier-transform. That is to say:

$$F\{\rho_{f,g,\omega}\}\left(\vec{k}\right) = F\{\rho_{a,b,\theta}\}\left(\vec{k}\right) \cdot F\{\rho_{c,d,\phi}\}\left(\vec{k}\right), \text{ or}$$

$$\frac{1}{fg}\rho_{1/f,1/g,\omega}\left(\vec{k}\right) = \frac{1}{ab}\rho_{1/a,1/b,\theta}\left(\vec{k}\right) \cdot \frac{1}{cd}\rho_{1/c,1/d,\phi}\left(\vec{k}\right).$$

This distribution $\rho_{f,g,\omega}$ can be determined by observing:

$$\frac{1}{fg}\rho_{1/f,1/g,\omega}\left(\vec{k}\right) = \frac{1}{2\pi}\exp\left(\frac{1}{2}\vec{k}^\intercal R_\omega \begin{bmatrix} f^2 & \\ & g^2 \end{bmatrix} R_\omega^\intercal \vec{k}\right), \text{ and}$$

$$\frac{1}{ab}\rho_{1/a,1/b,\theta}\left(\vec{k}\right) \cdot \frac{1}{cd}\rho_{1/c,1/d,\phi}\left(\vec{k}\right) = \frac{1}{4\pi^2}\exp\left(\frac{1}{2}\vec{k}^\intercal R_\theta \begin{bmatrix} a^2 & \\ & b^2 \end{bmatrix} R_\theta^\intercal \vec{k} + \frac{1}{2}\vec{k}^\intercal R_\phi \begin{bmatrix} c^2 & \\ & d^2 \end{bmatrix} R_\phi^\intercal \vec{k}\right).$$

Thus, the distribution $\rho_{f,g,\omega}$ can be determined by finding the f, g, ω that satisfy the following equations (drawn from the exponents in the previous equalities):

$$R_\theta \begin{bmatrix} a^2 & \\ & b^2 \end{bmatrix} R_\theta^\intercal + R_\phi \begin{bmatrix} c^2 & \\ & d^2 \end{bmatrix} R_\phi^\intercal = R_\omega \begin{bmatrix} f^2 & \\ & g^2 \end{bmatrix} R_\omega^\intercal.$$

These equalities can be rearranged into the following:

$$\begin{aligned} \left(a^2 - b^2\right)\cos 2\theta + \left(c^2 - d^2\right)\cos 2\phi &= \left(f^2 - g^2\right)\cos 2\omega \\ \left(a^2 - b^2\right)\sin 2\theta + \left(c^2 - d^2\right)\sin 2\phi &= \left(f^2 - g^2\right)\sin 2\omega \\ \left[a^2 + b^2\right] + \left[c^2 + d^2\right] &= \left[f^2 + g^2\right]. \end{aligned} \tag{2}$$

Thus, given 2θ, $\left[a^2 - b^2\right]$, 2ϕ and $\left[c^2 - d^2\right]$, we can use the first two equalities to find 2ω and $\left[f^2 - g^2\right]$ (see, e.g., Fig 88). Once this is accomplished we can use the third equality to find $\left[f^2 + g^2\right]$.

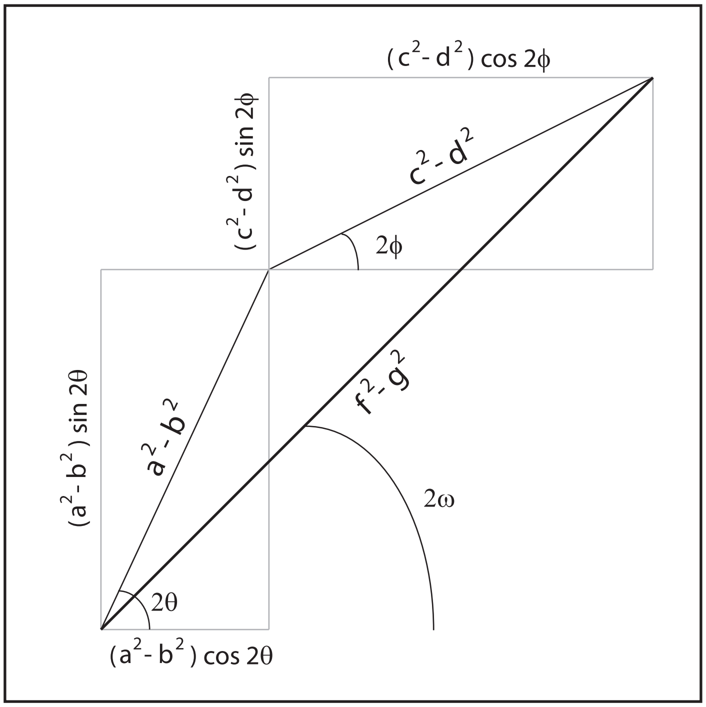

Figure 88: In this figure we show the relationships between $(f^2 - g^2)$, ω, $(a^2 - b^2)$, θ, $(c^2 - d^2)$, ϕ that are expressed in Eq. 2.

17 Asymptotic formula for $g_{1,\varepsilon,m}$:

When $\varepsilon\sqrt{m}$ is large (say, greater than 10), then Eq. 1 can be approximated via:

$$p\left(\omega, \frac{\varepsilon\sqrt{m}}{r}\right) \approx \frac{1}{2\pi}\left\{\pi + \sin(2\omega)\frac{r^2}{\varepsilon^2 m}\right\}, \text{ and } g_{1,\varepsilon,m} \approx \frac{1}{2} + \frac{2}{\pi^2\varepsilon^4 m^2}.$$

When $\varepsilon\sqrt{m}$ is small (say, less that 0.01), then Eq. 1 can be approximated via:

$$p\left(\omega, \frac{\varepsilon\sqrt{m}}{r}\right) \approx \frac{1}{\pi}\frac{\varepsilon\sqrt{m}}{r} \cdot \frac{1}{-\frac{1}{2}\sin 2\omega + \frac{2}{\pi}\frac{\varepsilon\sqrt{m}}{r}} = \frac{1}{2}\left[1 - \frac{\pi}{4}\frac{r}{\varepsilon\sqrt{m}}\sin 2\omega\right]^{-1} \quad \text{when } \sin 2\omega < 0, \text{ and}$$

$$p\left(\omega, \frac{\varepsilon\sqrt{m}}{r}\right) \approx 1 - \frac{1}{\pi}\frac{\varepsilon\sqrt{m}}{r} \cdot \frac{1}{+\frac{1}{2}\sin 2\omega + \frac{2}{\pi}\frac{\varepsilon\sqrt{m}}{r}} = 1 - \frac{1}{2}\left[1 + \frac{\pi}{4}\frac{r}{\varepsilon\sqrt{m}}\sin 2\omega\right]^{-1} \quad \text{when } \sin 2\omega > 0.$$

The integral associated with g is then given by:

$$\iint \frac{1}{2\pi}\left\{1 - 2p + 2p^2\right\} re^{-r^2/2} d\theta dr = \int_0^\infty \left\{r - \frac{4}{\pi}\varepsilon\sqrt{m}\left\{\frac{2}{\pi}\log\left(\frac{\pi}{2}\frac{r}{\varepsilon\sqrt{m}}\right) + \frac{1}{\pi}\right\}\right\} e^{-r^2/2} d\theta dr,$$

implying that:

$$g_{1,\varepsilon.m} \approx 1 - \frac{4}{\pi}\varepsilon\sqrt{m}\left[\frac{1}{\sqrt{2\pi}} + \frac{2}{\sqrt{2\pi}}\log\left(\frac{\pi}{2\varepsilon\sqrt{m}}\right) - \frac{2}{\pi}K\right],$$

$$\text{where } K = -\int_0^\infty \log(r)\exp\left(-r^2/2\right) dr \approx 0.7961.$$

18 Low-noise limit for $B^\intercal B$:

Let's consider a noiseless $m \times n$ rank-l bicluster B (i.e., with $\varepsilon = 0$). We can analytically solve for the expected value of the magnitude of the covariances $[B^\intercal B]_{kk'}$ in the limit $m, n \to \infty$.

Recall from section 5 that each column $B_{:,k}$ is obtained as follows: First we draw a sample vector $A_{:,k}$ of size $m \times 1$ from an eccentric gaussian-distribution on $\mathbb{R}^m$ with l principal-values equal to 1, and the remaining principal-values equal to 0. Then we define $B_{:,k}$ to be the binarization $B_{:,k} = \text{sign}(A_{:,k})$.

Because $\varepsilon = 0$, we construct the matrix A as follows:

$$A_{jk} = u_{j1}v_{k1} + \cdots + u_{jl}v_{kl} = \sum_{q=1}^{l} U_{jq}V^{\intercal}_{qk},$$

where $U \in \mathbb{R}^{m\times l}$ and $V \in \mathbb{R}^{n\times l}$, and $A = UV^{\intercal}$ is a singular-value-decomposition. Defining

$$\vec{u}_j = U_{j:} \text{ and } \vec{v}_k = V_{k:}$$

to be the jth-row of U and the kth-row of V, respectively, we can write A as:

$$A_{jk} = \vec{u}_j \cdot \vec{v}_k.$$

This allows us to write B as:

$$B_{jk} = \text{sign}\left(\vec{u}_j \cdot \vec{v}_k\right),$$

and $[B^{\intercal}B]$ as:

$$[B^{\intercal}B]_{kk'} = \sum_{j=1}^{m} \text{sign}\left(\vec{u}_j \cdot \vec{v}_k\right) \text{sign}\left(\vec{u}_j \cdot \vec{v}_{k'}\right).$$

For any particular $[B^{\intercal}B]_{kk'}$ the row-vectors $\vec{v}_k$ and $\vec{v}_{k'}$ are fixed. Any particular summand (corresponding to a particular j) will be $+1$ if that row-vector $\vec{u}_j$ makes an acute angle with both $\vec{v}_k$ and $\vec{v}_{k'}$. Similarly, a summand will be $+1$ if $\vec{u}_j$ makes an obtuse angle with both $\vec{v}_k$ and $\vec{v}_{k'}$. On the other hand, a summand will be -1 if and only if $\vec{u}_j$ makes an acute angle with one of the $\vec{v}_k, \vec{v}_{k'}$, and an obtuse angle with the other. If m is large, we can assume that each of the $\vec{u}_j$ are independent and randomly oriented. Thus, if we let $\theta \in [0, \pi]$ be the angle between $\vec{v}_k$ and $\vec{v}_{k'}$, then any particular $\vec{u}_j$ has probability θ/π of contributing a summand of -1 to $[B^{\intercal}B]_{kk'}$. Conversely, any particular $\vec{u}_j$ has probability $1-\theta/\pi$ of contributing a summand of $+1$. Thus, given a fixed set of $\vec{v}_k$ and $\vec{v}_{k'}$, the expected value of $[B^{\intercal}B]_{kk'}$ will be $m\left(1 - 2\theta/\pi\right)$.

To determine the expected value of $[B^{\intercal}B]_{kk'}$ for an arbitrary $\vec{v}_k$ and $\vec{v}_{k'}$, we simply integrate $m\left(1 - 2\theta/\pi\right)$ over the distribution $\mu(\theta)$ of θ. This distribution θ depends on the ambient dimension l:

$l = 1$: The $\vec{v}_k$ and $\vec{v}_{k'}$ lie on a line, and are either parallel or antiparallel: $\mu(\theta) = \delta(\theta - 0) + \delta(\theta - \pi)$.

$l = 2$: The $\vec{v}_k$ and $\vec{v}_{k'}$ lie on a circle: $\mu(\theta) = 1/\pi$

$l = 3$: The $\vec{v}_k$ and $\vec{v}_{k'}$ lie on a sphere: $\mu(\theta) = \sin(\theta)/2$

$l = 4$: The $\vec{v}_k$ and $\vec{v}_{k'}$ lie on a sphere in $l = 4$ dimensions: $\mu(\theta) = \sin^2(\theta)/(\pi/2)$

$l > 4$: The $\vec{v}_k$ and $\vec{v}_{k'}$ lie on a sphere in l dimensions: $\mu(\theta) = \sin^{l-2}(\theta)/Z_l$.

In the cases where $l \geq 2$, the normalization factor Z_l is equal to:

$$\begin{aligned} Z_l &= \int_0^{\pi} \sin^{l-2}(\theta)\, d\theta = 2Y_{l-2}, \text{ where} \\ Y_q &= \begin{cases} \frac{(q-1)(q-3)(q-5)\cdots(1)}{(q-0)(q-2)(q-4)\cdots(2)}\frac{\pi}{2} & \text{if } l \text{ is even} \\ \\ \frac{(q-1)(q-3)(q-5)\cdots(2)}{(q-0)(q-2)(q-4)\cdots(1)}1 & \text{if } l \text{ is odd} \end{cases} \end{aligned}$$

Note that the first few Y_q are given by:

$$Y_0 = \pi/2,\ Y_1 = 1, \text{ and } Y_q = \frac{q-1}{q}Y_{q-2} \text{ for } q \geq 2.$$

Using this construction we can calculate the expected value of magnitude of $[B^{\intercal}B]_{kk'}$, assuming that $k \neq k'$:

$$E\left(\left|[B^{\intercal}B]_{kk'}\right|\right) = m\int_0^{\pi} \left|1 - 2\theta/\pi\right| \mu(\theta)\, d\theta = 2m\int_0^{\pi/2} \left(1 - 2\theta/\pi\right)\mu(\theta)\, d\theta.$$

Noting that:

$$\int_0^{\pi/2} \sin^q(\theta)\, d\theta = Y_q, \text{ and } \int_0^{\pi/2} \theta \cdot \sin^q(\theta)\, d\theta = X_q, \text{ with}$$

$$X_0 = \frac{\pi^2}{8},\ X_1 = 1, \text{ and } X_q = \frac{1}{q^2} + \frac{q-1}{q}X_{q-2} \text{ for } q \geq 2,$$

we see that:

$$E\left(\left|[B^{\intercal}B]_{kk'}\right|\right) = m\left[1 - \frac{2}{\pi}\frac{X_{l-2}}{Y_{l-2}}\right].$$

Evaluating this expression for the first few l gives:

$l = 1$ **:** $E\left(\left|[B^{\intercal}B]_{kk'}\right|\right) = m \cdot 1$

$l = 2$ **:** $E\left(\left|[B^{\intercal}B]_{kk'}\right|\right) = m \cdot [1 - 1/2] = m/2$

$l = 3$ **:** $E\left(\left|[B^{\intercal}B]_{kk'}\right|\right) = m \cdot \left[1 - \frac{2}{\pi}\right] \approx m \cdot 0.3634$

$l = 4$ **:** $E\left(\left|[B^{\intercal}B]_{kk'}\right|\right) = m \cdot \left[1 - \frac{2}{\pi}\left(1/4 + \pi^2/16\right) / (\pi/4)\right] \approx m \cdot 0.2974$

While the following is not an exact formula, when $l \geq 2$ this expected value is quite close to:

$$E\left(\left|[B^{\intercal}B]_{kk'}\right|\right) \approx \frac{m}{2\sqrt{l-1}}.$$

19 Information-theoretic phase-transitions for rank-1 planted-bicluster problem

In this section we discuss in more detail the information-theoretic size- and noise-transitions for the rank-1 planted-bicluster problem. This problem involves sifting through an $M \times M$ binary data-matrix D, and distinguishing between the following two hypotheses.

H0: The null hypothesis. In this hypothesis the data-matrix D has no planted bicluster within it; each entry of D is chosen randomly and independently from $\{-1, +1\}$.

H1: The planted-bicluster hypothesis: In this hypothesis the data-matrix D is altered by planting within it a smaller $m \times m$ submatrix which corresponds to the binarization of a randomly-oriented rank-1 error-ε bicluster. The entries of D outside of this $m \times m$ submatrix will be chosen randomly and independently as before, but the entries of D within this $m \times m$ submatrix will depend on one another.

One step towards understanding the planted-bicluster problem is to determine when H1 is distinguishable from H0; i.e., how large m needs to be (and how small ε must be) before the planted-bicluster impacts the distribution of the elements of D. To address this question we assume that D is drawn from H0, and ask what kinds of structures might naturally exist within D, even without any deliberate attempt to plant any specific biclusters. As we'll see below, even under H0 we still expect to find within D a vast number of biclusters. These biclusters include (a) small noiseless biclusters of size $m \lesssim 2\log_2 M$ and (b) much larger and noisier biclusters of size $m \sim \sqrt{M}$ and $\varepsilon \gtrsim [\pi \log M]^{-1/2}$, as well as many structured elements in between these two extremes. Nevertheless, under H0 we are exponentially unlikely to find biclusters that are both large and low-noise.

Size phase-transition: Let's assume the noise $\varepsilon = 0$ for the moment, and consider the limit $M \gg m \gg 1$. Let's take a particular $m \times m$ submatrix B within D. The requirement that B be rank-1 places constraints on the signs of $(m-1)^2$ elements of B. That is, B will be rank-1 with probability $p_0 = 2\hat{}\left(-(m-1)^2\right)$, or approximately -log $p_0 \approx \log 2 \cdot m^2$. The total number of such $m \times m$ submatrices within D is $Z = \binom{M}{m}^2$, which can be approximated as $Z \approx \exp\left(2m\log(M/m) + 2m + 2m^2/M\right)$, or approximately $\log Z \approx 2m \log M$. The product $P_0 = 1 - (1-p_0)^Z \approx Zp_0$ provides an upper bound on the probability that D contains at least one $m \times m$ rank-1 submatrix (this is technically an overestimate because the ranks of each of the Z submatrices are not independent of one another). Obviously, this upper bound P_0 tends to 0 when $-\log p_0 \gg \log Z$, which occurs when m is larger than $[2/\log 2]\log M = 2\log_2 M$. This means that, for $M \gg m \gg 1$, a random binary-matrix D is exponentially unlikely to contain any $m \times m$ rank-1 matrix, as long as $m \gtrsim 2\log_2 M$. One consequence of these observations is that, if we were to embed within D a submatrix B of size m in the range $2\log_2(M) < m < \sqrt{M}$, say $m \sim \sqrt[3]{M}$, then B would be very significant but still undetectable to our algorithm.

Noise phase-transition: Now let's consider the second phase-transition, this time assuming $M \gg m \gg \log M$. As before, we'll assume that D is an $M \times M$ binary-matrix with each entry chosen independently from $\{-1, +1\}$, and we'll consider $m \times m$ submatrices B within D. This time we are interested in the probability $p_{\varepsilon\sqrt{m}}$ that a particular B is rank-1 with noise at most $\varepsilon\sqrt{m}$. As we pointed out above, when $\varepsilon\sqrt{m} = 0$ we impose constraints on the signs of $\approx m^2$ elements of B; any particular B will be exactly rank-1 with probability $p_0 \approx 2\hat{}\left(-m^2\right)$. If instead we require B to be rank-1 with noise $\varepsilon\sqrt{m}$, we actually only impose constraints on $\approx \left(1 - 2f_{\varepsilon\sqrt{m}}\right)m^2$ randomly chosen elements of B

(where $f_{\varepsilon\sqrt{m}}$ is the 'flip-probability' discussed in section 5.1). Assuming that $\varepsilon\sqrt{m} \gtrsim 10$, we can approximate $f_{\varepsilon\sqrt{m}}$ with $f_{\varepsilon\sqrt{m}} \approx 1/2 - \left[\sqrt{2\pi}\cdot\varepsilon\sqrt{m}\right]^{-1}$. An asymptotic expansion in terms of $\varepsilon\sqrt{m}$ reveals that, within the large-$\varepsilon\sqrt{m}$ regime:

$$p_{\varepsilon\sqrt{m}} \approx \sum_{fm^2=0}^{fm^2=f_{\varepsilon\sqrt{m}}\cdot m^2} \binom{m^2}{fm^2}\frac{1}{2^{m^2}} \approx \frac{1}{2}\frac{\varepsilon\sqrt{m}}{m}\cdot\exp\left(-\frac{m^2}{\pi\varepsilon^2 m}\right).$$

Thus, we see that:

$$-\log p_{\varepsilon\sqrt{m}} \approx \frac{m^2}{\pi\left(\varepsilon\sqrt{m}\right)^2} + \log m + \log 2 - \log\left(\varepsilon\sqrt{m}\right).$$

Comparing this with $\log Z = 2m\log M - 2m(\log m - 1)$, we see that $P_{\varepsilon\sqrt{m}} = 1 - \left(1 - p_{\varepsilon\sqrt{m}}\right)^Z \approx Zp_{\varepsilon\sqrt{m}}$ vanishes when

$$\frac{m^2}{\pi\left(\varepsilon\sqrt{m}\right)^2} + \log m + \log 2 - \log\left(\varepsilon\sqrt{m}\right) \gg 2m\log M - 2m(\log m - 1).$$

If we assume that $m \sim \sqrt{M}$, then $P_{\varepsilon\sqrt{m}}$ will vanish when

$$\frac{1 + 1/M\cdot\log 2 - 1/M\cdot\log\left(\varepsilon\sqrt{m}\right)}{\log M + 2} \gg \pi\varepsilon^2.$$

Consequently, when $\varepsilon\sqrt{m} \gtrsim 10$ and $m \sim \sqrt{M}$, we can expect that $P_{\varepsilon\sqrt{m}}$ will be essentially 0 whenever $\pi\varepsilon^2 \ll 1/\log M$, or when $\varepsilon\sqrt{m} \ll M^{1/4}/\sqrt{\pi\log M}$. This implies that a large random $M\times M$ matrix D is exponentially unlikely to contain any $\sqrt{M}\times\sqrt{M}$ rank-1 biclusters B which have a first-principal-component accounting for $\gtrsim \pi\log M/\sqrt{M}$ of their total variance. Put another way, if we were to embed within D an ε-error rank-1 $\sqrt{M}\times\sqrt{M}$ bicluster B which had, say, $\varepsilon \sim 1/\sqrt[3]{M}$, then B would be very statistically significant but still undetectable to our algorithm.

20 Does binarization destroy angular information?

This section describes the details of Fig 28. The scenario we investigate involves loops drawn from an $m\times m$ ε-error rank-1 bicluster B. If we select 2 columns of B at random, any 1×2 row-vector from these two columns will be drawn from the 2-dimensional distribution $\tilde{\rho}$ described in section 5. The distribution $\tilde{\rho} \sim \rho_{r_1,0,\theta_1} \star \rho_{\varepsilon,\varepsilon,0}$, where r_1 is drawn from $r\sqrt{2\pi m}\rho_{\sqrt{1/m}}(r)$ and θ_1 is uniformly distributed on $[0, 2\pi]$. Given r_1 and $\theta_1 = 0$, a 1×2 vector randomly drawn from $\tilde{\rho}$ gives rise to an angle ω drawn from:

$$\rho_B^{\text{original}}(\omega) \sim \frac{1}{2\pi}\frac{ab}{b^2\cos^2(\omega-\theta_1) + a^2\sin^2(\omega-\theta_1)}, \text{ with } a = \sqrt{r_1^2+\varepsilon^2}, \text{ and } b = \sqrt{0^2+\varepsilon^2} = \varepsilon.$$

By contrast, the angle ω of a 1×2 vector drawn from outside B is uniformly distributed (i.e., $\rho_D^{\text{original}}(\omega) = 1/2\pi$). These two distributions can be used to calculate the relative-entropy $\mathcal{D}_{KL}^{\text{original}}\left(\varepsilon\sqrt{m}\right)$ associated with the original bicluster. After binarization, the distributions $\rho_B^{\text{binarized}}(\omega)$ and $\rho_D^{\text{binarized}}(\omega)$ are given by:

$$\rho_B^{\text{binarized}}(\omega) = \begin{cases} \frac{1}{\pi}\operatorname{arccot}\left(\left(\left|\frac{b}{a}\right| - \left|\frac{a}{b}\right|\right)\frac{1}{2}\sin 2\theta_1\right) & \text{for } \omega = \pi/4 \text{ or } 5\pi/4 \\ \frac{1}{\pi}\operatorname{arccot}\left(\left(\left|\frac{a}{b}\right| - \left|\frac{b}{a}\right|\right)\frac{1}{2}\sin 2\theta_1\right) & \text{for } \omega = 3\pi/4 \text{ or } 7\pi/4 \end{cases}$$

$$\rho_D^{\text{binarized}}(\omega) = \begin{cases} 1/2 & \text{for } \omega = \pi/4 \text{ or } 5\pi/4 \\ 1/2 & \text{for } \omega = 3\pi/4 \text{ or } 7\pi/4 \end{cases}$$

These two distributions can be used to calculate the relative-entropy $\mathcal{D}_{KL}^{\text{binarized}}\left(\varepsilon\sqrt{m}\right)$ associated with the binarized version of the bicluster. Shown in Fig 28A are several randomly drawn distributions of this type (each drawn with a different r_1 and θ_1). As illustrated in Fig 28B, there is little difference between $\mathcal{D}_{KL}^{\text{original}}$ and $\mathcal{D}_{KL}^{\text{binarized}}$ when $\varepsilon\sqrt{m} \gtrsim 0.01$.

21 Analysis of loop-scores

In this section we analyze our loop-scores and show that they are, in a certain sense, close to optimal within the context of our methodology. In this section we'll use the notation $l[J;K]$ to refer to a $c\times c$-submatrix involving rows $J = \{j_1,\ldots,j_c\}$ and columns $K = \{k_1,\ldots,k_c\}$. To ease the discussion, we'll refer to these $c\times c$ submatrices as 'nets'. Our analysis begins by noting that our original loop-score was just one of a large class of 'c-scores' which are constructed using the following general template:

Step (i) Given a row j, consider the set S_j of all $c \times c$-nets $l\,[j_1, \ldots, j_c; k_1, \ldots, k_c]$ with $j \in \{j_1, \ldots, j_c\}$.

Step (ii) Measure some scalar quantity $\mu\,(l)$ for each $l \in S_j$;

Step (iii) Aggregate the sum $[Z_{\text{ROW}}]_j = \sum_{l \in S_j} \mu\,(l)$.

Our original loop-scores fall within the above template using $c = 2$ and $\mu\,(l)$ related to the rank of l (i.e., $\mu\,(l) = 3 - 2{\cdot}\text{rank}(l)$). It is natural to ask: Could we gain an advantage if we allowed for the more general steps (i) and (ii) above? Specifically, instead of merely looking at loops $l\,[j_1, j_2; k_1, k_2]$, we might consider larger $c \times c$ nets $l\,[j_1, \ldots, j_c; k_1, \ldots, k_c]$. Moreover, instead of simply measuring the rank $\mu\,(l)$, we could use some more informative measurement instead (e.g., any other scalar-valued function $\mu : \{-1, +1\}^{c \times c} \to \mathbb{R}$). It turns out that neither of these modifications can substantially improve the c-score; no matter which fixed c we choose, or how we construct μ, we won't be able to overcome the size-threshold of $\sqrt{M}$.

To see why this might be the case, let's consider a strictly easier problem: let's assume that the signs of u and v are known to be all positive; i.e., B is simply a rank-1 $m \times m$ matrix with each entry equal to $+1$. We'll show below that no algorithm built using the template above can overcome the size-threshold of $\sqrt{M}$ for this strictly easier problem. This then suggests that the same size-threshold applies to our original planted-bicluster problem.

In step (i) of the template, each net $l\,[J; K]$ is defined with the restriction that $j \in J$. Any net l may overlap B; there may be $x = |J \cap J_B|$ rows in common and $y = |K \cap K_B|$ columns in common. If $x = 0$ and $y = 0$, then $l\,[J; K]$ is drawn from outside B. While the joint-distribution across all such nets is highly structured, each individual net drawn from outside B looks 'fully-random'. That is to say, if a single net is drawn from outside B, the joint-distribution from which its own entries are drawn will be indistinguishable from that of a net whose entries are each drawn independently from $\{-1, +1\}$. Any individual net with $x = 0$ or $y = 0$ will also look fully-random. Only when both $x \geq 1$ and $y \geq 1$ will the structure of B impact the distribution from which the entries of $l\,[J; K]$ are drawn. When $x = 1$ and $y = 1$ the entries of $l\,[J; K]$ no longer look fully random; there will be some randomly-located entry that is guaranteed to be equal to $+1$, and the remaining entries of l will be drawn independently from $\{-1, +1\}$. As x and y increase, the structure of B impacts the entries of $l\,[J; K]$ more and more severely, restricting a larger and larger fraction of $l\,[J; K]$ to be positive until, when $x = m$ and $y = m$, the entire net $l\,[J; K]$ is forced to be $+1$.

It turns out that, in the limit as M and m go to infinity (with $m/M \to 0$), only the singly-overlapping nets with $x = 1$ and $y = 1$ contribute meaningfully to the sum in step (iii). That is to say, we can safely ignore all nets with multiple overlapping entries (i.e., with $x + y > 2$). There are two reasons which, together, ensure that this is the case.

1. The first reason is that, as m and M get larger, there will be $\sim m^{x+y} M^{2c-x-y}$ different nets with row- and column-overlaps of x and y, respectively. If the given row j lies inside B, then there will be $\sim m^1 M^{2c-2}$ nets with $x = 1, y = 1$, and only $\sim m^2 M^{2c-3}$ multiply-overlapping nets with $x + y > 2$. Similarly, if j lies outside B, then there will be $\sim m^2 M^{2c-3}$ nets with $x = 1, y = 1$, and only $\sim m^3 M^{2c-4}$ multiply-overlapping nets. In both cases the fraction of nets which are impacted by the presence of B is dominated by those nets with the smallest possible overlap – in this case $x = 1$ and $y = 1$. The fraction of multiply-overlapping nets will always be $\sim m/M$ smaller than the fraction of singly-overlapping nets.

2. The second reason is that, no matter the choice of c, the distributions of possible configurations allowed within the multiply-overlapping nets is always comparable to the distribution of possible configurations allowed within the singly-overlapping nets. In other words, the relative-entropy between these two configuration-distributions remains bounded by a constant depending only on c, regardless of the choice of x or y for the multiply-overlapping nets. Put another way, in order for us to safely ignore multiply-overlapping nets, it is imperative that these nets cannot adopt a configuration forbidden to singly-overlapping nets. This is the case here: there are only $2^{c \cdot c}$ possible configurations allowed for any net, and all of these configurations can be found (with probability bounded away from 0) amongst either the singly- or multiply-overlapping nets. We remark that a similar statement would not hold if, say, the data were not binarized and the planted bicluster were exactly rank-1 with unknown u and v. In such a scenario any multiply overlapping nets with $c = 2$, $x = 2$ and $y = 2$ would all have a second singular value of exactly 0. If we were to compare this configuration-distribution with that of the $c = 2$, $x + y < 4$ nets, we would find the relative-entropy unbounded: the multiply-overlapping nets contain infinitely more information than the singly-overlapping nets, and cannot be ignored (this is the same scenario we brought up earlier when discussing the information-loss associated with binarization).

Given the justification above, let's restrict our attention to $l\,[J; K]$ for which $x = 1$ and $y = 1$. These were the nets which contained a single randomly-located entry that was fixed to be $+1$ (with the other entries randomly chosen to be either $+1$ or -1). Because only a single entry within each net has a fixed sign, we don't need to worry about the joint-distribution of signs across entries. Therefore, the only relevant quantity for each of these nets is the number $\chi\,(l)$ of positive entries within l (i.e., $\chi\,(l)$ ranges from 0 to c^2). Thus, any μ that we construct must be be a function of $\chi\,(l)$ (i.e., $\mu = \mu\,(\chi\,(l))$). Consequently, given step (iii), our final c-score for row j must be of the form $[Z_{\text{ROW}}]_j = \mu\,(0)\, L_0^j + \cdots \mu\left(c^2\right) L_{c^2}^j$, where

L^j_χ is the total number of nets intersecting row j that contain exactly χ positive entries. In other words, the final c-score for row j takes the form $[Z_{\text{ROW}}]_j = \vec{\mu} \cdot \vec{L}^j$, where $\vec{\mu} \in \mathbb{R}^{1+c^2}$ is the vector formed from the various $\mu(\chi)$, and $\vec{L}^j \in \mathbb{N}^{1+c^2}$ is the vector formed from the L^j_χ. The difference between the c-score for some row j and the c-score for another row j' depends only on the difference between $\vec{L}^j$ and $\vec{L}^{j'}$.

To understand the structure of $\vec{L}^j$ more clearly, let's restrict our attention to a fixed column-subset $K = \{k_1, \ldots, k_c\}$. Given that column-subset K is fixed, we can consider various row-subsets $\bar{J} = \{j_1, \ldots, j_{c-1}\}$, each chosen such that $j \notin \bar{J}$ and $\bar{J} \cap J_B = \emptyset$. For each choice of $\bar{J}$, the $(c-1) \times c$ sub-net $l\left[\bar{J}; K\right]$ will have some number of positive entries $\chi\left(l\left[\bar{J}; K\right]\right)$. This collection of values for χ (taken across the various $\bar{J}$) can be encoded as a vector $\vec{P}^K \in \mathbb{N}^{1+(c-1)c}$ (i.e., $\vec{P}^K_\chi$ is the number of row-subsets $\bar{J}$ such that $\chi\left(l\left[\bar{J}; K\right]\right) = \chi$). Now the $(c-1) \times c$ sub-net $l\left[\bar{J}; K\right]$ can be can be expanded by adding the row $l[j; K]$ to form $l\left[j \cup \bar{J}; K\right]$. Obviously, $\chi\left(l\left[j \cup \bar{J}; K\right]\right)$ is equal to $\chi(l[j; K]) + \chi\left(l\left[\bar{J}; K\right]\right)$, implying that the collection of values for $\chi\left(l\left[j \cup \bar{J}; K\right]\right)$ (taken across the various $\bar{J}$) can be determined simply by embedding the vector $\vec{P}^K$ in $\mathbb{N}^{1+c^2}$ and shifting it forward by $\chi(l[j; K])$ indices. That is to say, the collection of values for $\chi\left(l\left[j \cup \bar{J}; K\right]\right)$ is simply $\vec{P}^K \star \vec{e}_{\chi(l[j;K])}$, where '$\star$' denotes a vector-convolution and $\vec{e}_\chi$ is a vector with entry χ equal to 1 and the other entries equal to 0.

With this notation the vector $\vec{L}^j$ can be written as:

$$\vec{L}^j = \sum_K \vec{P}^K \star \vec{e}_{\chi(l[j;K])},$$

where the vector $\vec{P}^K$ is calculated by summing over all row-subsets $\bar{J}$ that exclude j. At this point we are ready to compare the row j, which we'll assume lies in J_B, with another row j' taken from outside B. We can immediately see that:

$$\vec{L}^j - \vec{L}^{j'} = \sum_K \vec{P}^K \star \left[\vec{e}_{\chi(l[j;K])} - \vec{e}_{\chi(l[j';K])}\right],$$

where the vector $\vec{P}^K$ is calculated by summing over all row-subsets $\bar{J}$ that exclude both j and j' (i.e., nets which include both j and j' will contribute to the same χ-index within $\vec{L}^j$ and $\vec{L}^{j'}$). Recalling our discussion above, we need only consider nets $l[J; K]$ that have an overlap $x = |J \cap J_B|$, and $y = |K \cap K_B|$ of at most 1. This implies that we can calculate the vector $\vec{P}^K$ by summing over only those row sub-sets $\bar{J}$ that exclude J_B as well as j'. With this assumption each $l\left[\bar{J}; K\right]$ is disjoint from B, and each $\chi\left(l\left[\bar{J}; K\right]\right)$ will be drawn from the same binomial distribution

$$P\left(\chi\left(l\left[\bar{J}; K\right]\right) = \chi\right) = \binom{(c-1)c}{\chi} 2^{-(c-1)c}.$$

Thus the expected value of $\sum_K \vec{P}^K$ is merely the binomial distribution above multiplied by the number of possible $\bar{J}$:

$$E\left(\sum_K \vec{P}^K_\chi\right) = \binom{M-m-1}{c-2}\binom{(c-1)c}{\chi} 2^{-(c-1)c}.$$

Based on the linearity of expectation and the independence of $\vec{P}^K$ and $\chi(l[j, K])$ and $\chi(l[j'; K])$, we have that:

$$E\left(\vec{L}^j - \vec{L}^{j'}\right) = E\left(\sum_K \vec{P}^K_\chi\right) \star \left[\vec{E}^K_j - \vec{E}^K_{j'}\right], \text{ where}$$

$$\left[\vec{E}^K_j\right]_\chi = \left[E\left\{\sum_K \vec{e}_{\chi(l[j;K])}\right\}\right]_\chi \sim \binom{M}{c}\left\{\frac{M-m}{M}\binom{c}{\chi}2^{-c} + \frac{m}{M}\binom{c-1}{\chi-1}2^{-c+1}\right\}, \text{ and}$$

$$\left[\vec{E}^K_{j'}\right]_\chi = \left[E\left\{\sum_K \vec{e}_{\chi(l[j';K])}\right\}\right]_\chi \sim \binom{M}{c}\binom{c}{\chi}2^{-c}.$$

Thus, the ability for Z_{ROW} to discriminate between j and j' is limited by the difference between the distributions $\vec{E}^K_j$ and $\vec{E}^K_{j'}$. A calculation of their relative-entropy shows that these two distributions $\vec{E}^K_j$ and $\vec{E}^K_{j'}$ are maximally differentiable when $c = 1$. When $c = 1$ the distribution $E\left(\sum_K \vec{P}^K_\chi\right)$ is trivial and the choice of $\vec{\mu}$ is irrelevant (so long as $\mu(0) \neq \mu(1)$). In this case the best c-score for each row is simply the number of positive entries in that row. This is equivalent to simply using the 'degree' of each row (treating the matrix D as the adjacency matrix of a bigraph). A similar statement holds for the columns: The best c-score for each column is simply the number of positive entries in that column. This simple score – i.e., counting degree – is only informative when $m \gtrsim \sqrt{M}$.

Putting all this together, we see that – for the easier problem when B is a matrix of all '+1's – there is no advantage to be gained by considering larger $c \times c$-submatrices, or by considering a more general measurement $\mu(l)$. The choice of $c = 1$ is asymptotically optimal and, regardless of the choice of μ, the scores will only be indicative of J_B and K_B when $m \gtrsim \sqrt{M}$ (and won't be indicative of B when $m = o(\sqrt{M})$. In other words; algorithms built using the template above will always be subject to the same size-threshold of $m \gtrsim \sqrt{M}$. Therefore, we conclude that our original problem also suffers from the same size-threshold limitation; regardless of our choice of c or μ, the template above won't be able to construct scores which indicate the rows and columns of B when $m = o(\sqrt{M})$.

To summarize: we have argued that the size-transition exhibited by our loop-scores will also be exhibited by a rather large class of similar scores. To be more specific, we conclude that – for fixed $\varepsilon\sqrt{m}$ – all scores constructed using the template above will be informative only when $m \gtrsim \sqrt{M}$, and will not be informative when $m = o(\sqrt{M})$. This then implies that – when $\varepsilon\sqrt{m} = 0$ – our loop-scores are asymptotically optimal (within the constraints of the template above).

22 Comparison with a simple spectral-biclustering method

Our loop-counting algorithm is closely related to 'spectral-biclustering' [$\mathcal{M}$22,$\mathcal{M}$40]. Indeed, if we ignore the correction for collapsed-loops, we see that our loop-score $[Z_{\text{ROW}}]_j$ is the diagonal entry $[DD^\intercal DD^\intercal]_{jj}$, which is proportional to the rayleigh-quotient $\vec{x}^\intercal [D^\intercal D] \vec{x}$, with $\vec{x}^\intercal = D_{j,:}$ representing the jth-row of D. Similarly, $[Z_{\text{COL}}]_k$ is the rayleigh-quotient $\vec{y}^\intercal [DD^\intercal] \vec{y}$, with $\vec{y} = D_{:,k}$ representing the kth-column of D. Our algorithm attempts to focus on those rows and columns which correspond to large rayleigh-quotients; i.e., to those rows and columns which are most parallel to the dominant eigenvectors of $D^\intercal D$ and $DD^\intercal$, respectively. If we decompose $D = U\Sigma V^\intercal$ using the singular-value-decomposition, we see that the dominant eigenvectors of $D^\intercal D$ and $DD^\intercal$ are equal to the dominant right- and left-singular-vectors $\vec{v} = V_{:,1}$ and $\vec{u} = U_{:,1}$ of the data-matrix D. Therefore, one can imagine that the rows and columns which our algorithm focuses on will be those that are most highly correlated with $\vec{v}$ and $\vec{u}$, respectively. These rows and columns should thus correspond to high-magnitude components within the vectors $\vec{u}$ and $\vec{v}$, respectively.

Given these observations, it is natural to consider the following simple spectral-biclustering method:

Step 0 Binarize D, sending each entry to either $+1$ or -1 (i.e., $D = \text{sign}(D)$ or $D = 2(D > 0) - 1$).

Step 1 Calculate the singular-value-decomposition $D = U\Sigma V^\intercal$. Set $\vec{u}$ and $\vec{v}$ to be the first columns of U and V.

Step 2 Set $[Z_{\text{ROW}}]_j = \vec{u}_j^2$ and $[Z_{\text{COL}}]_k = \vec{v}_k^2$ to be the entrywise-squares of the singular-vectors $\vec{u}$ and $\vec{v}$.

Step 3 Use Z_{ROW} and Z_{COL} to produce a ranked list of rows and columns of D.

Another way to think about the connection between our loop-counting algorithm and this spectral-biclustering method is to first consider the bigraph corresponding to the binarized version of D. Within this context, we compute the row-loop-score by considering each $(1+1) \times 2 = 4$-step path $(j,k) \to (j',k) \to (j',k') \to (j,k')$ through the bigraph associated with D (see Fig 89). Let us denote such a path by $p[J;K]$, indexed by ordered row-subsets $J = \{j,j'\}$ and column-subsets $K = \{k,k'\}$ (here j is fixed, but j', k and k' are arbitrary). Note that this path begins and ends at the fixed row j (i.e., this path is a loop) . For each $p[J;K]$ we calculate the product of the matrix entries along that path:

$$\pi(p[\{j,j'\};\{k,k'\}]) = \prod_{s=0}^{1} D_{j^{[s]}k^{[s]}} D_{j^{[s+1]}k^{[s]}},$$

where $j^{[s]}$ corresponds to the j-index with s 'primes' atop it, adopting the convention that $j^{[2]} = j'' = j$. As expected from our loop-scores, these simple $(1+1) \times 2$-step paths produce a product of the form:

$$\pi(p[\{j,j'\};\{k,k'\}]) = \prod_{s=0}^{1} D_{j^{[s]}k^{[s]}} D_{j^{[s+1]}k^{[s]}} = D_{jk} D_{j'k} D_{j'k'} D_{jk'}.$$

Note that the path $p[J;K]$ steps on each row and each column an even number of times. Consequently, if $p[J;K]$ were fully contained within a bicluster that was exactly rank-1, then π would always equal $+1$. More generally, within the context of the planted-bicluster problem, the product $\pi(p[J;K])$ is more likely to be $+1$ if the path $p[J;K]$ is fully contained within a planted bicluster. On the other hand, we expect that $\pi(p)$ will be equally likely to be $+1$ or -1 when the path p is not fully contained within a planted bicluster. Once we have the product $\pi(p)$ for each path $p[J;K]$, we aggregate the results to form the score for row-j, which we'll denote by $\left[Z_{\text{ROW}}^1\right]_j := [Z_{\text{ROW}}]_j$:

$$\left[Z_{\text{ROW}}^1\right]_j = \sum_{J,K,\ j \text{ fixed}} \pi(p[J;K]), \text{ which is equal to } \left[(DD^\intercal)^{1+1}\right]_{jj}.$$

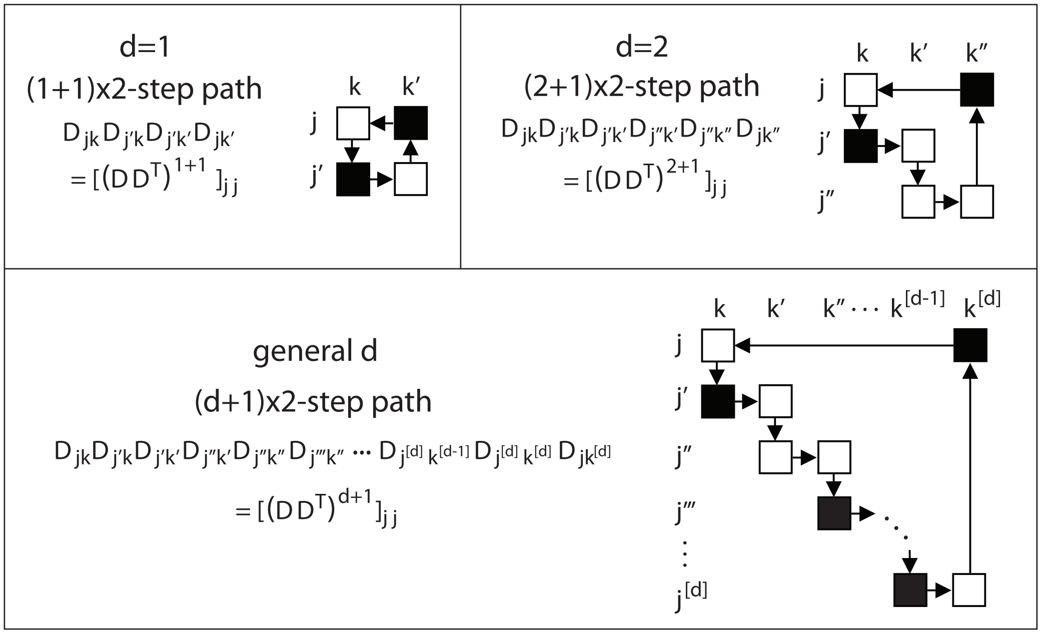

Figure 89: Illustration of paths through a bigraph. Our original row-scores $\left[Z^1_{\text{ROW}}\right]_j$ are computed by considering all $(1+1)\times 2 = 4$-step paths through the bigraph of D which both start and end at row-j (see top-left). If such a 4-step path lies entirely within a rank-1 bicluster it will satisfy the following property: the product of the matrix-entries along the path will be equal to $+1$. The accumulation of such a product over all possible paths is given by $\left[Z^1_{\text{ROW}}\right]_j = \left[(DD^\intercal)^{1+1}\right]_{jj}$. We may also consider other paths through the bigraph which start and end at row-j. On the top-right we show one such family of paths: namely $(2+1)\times 2 = 6$-step paths. One can easily see that these 6-step paths also have the same property as the 4-step paths above: namely, the product of the matrix-entries along the path will be equal to $+1$ if the path lies entirely within a rank-1 bicluster. The accumulation of these products is given by $\left[(DD^\intercal)^{2+1}\right]_{jj}$. On the bottom we generalize this notion to $(d+1)\times 2$-step paths. Again, if such a path is contained within a rank-1 bicluster, the product of its entries will be $+1$. The accumulation of these products is given by $\left[(DD^\intercal)^{d+1}\right]_{jj}$. As d grows, the accumulation $\left[(DD^\intercal)^{d+1}\right]_{jj}$ converges (after appropriate rescaling) to the jth-component of the dominant left-singular-vector of D. A similar statement can be made for paths which begin and end at column-k: as d increases the accumulation $\left[(D^\intercal D)^{d+1}\right]_{kk}$ converges to the kth-component of the dominant right-singular-vector of D.

We can generalize this approach by considering longer $(d+1)\times 2$-step paths, rather than simple $(1+1)\times 2$-step paths. Some examples are illustrated in Fig 89. These paths involve larger row-subsets $J = \left\{j, j', \ldots, j^{[d]}\right\}$ and $K = \left\{k, k', \ldots, k^{[d]}\right\}$. The product $\pi(p)$ is given by:

$$\pi\left(p\left[J;K\right]\right) = \prod_{s=0}^{d} D_{j^{[s]}k^{[s]}} D_{j^{[s+1]}k^{[s]}},$$

with the convention that $j^{[d+1]} = j$. As before, within the context of the planted-bicluster problem, $\pi(p)$ is more likely to be $+1$ whenever p is fully contained within a planted bicluster; if p is not contained within a planted bicluster then $\pi(p)$ is more likely to be drawn from the uniform distribution on $\{-1,+1\}$ (we'll quantify this more precisely below). The accumulation of such products, which we'll denote by $\left[Z^d_{\text{ROW}}\right]_j$ is now given by:

$$\left[Z^d_{\text{ROW}}\right]_j = \sum_{J,K,\ j \text{ fixed}} \pi\left(p\left[J;K\right]\right) = \left[(DD^\intercal)^{d+1}\right]_{jj}.$$

As d increases, the d-score $\left[Z^d_{\text{ROW}}\right]$ converges in direction to the entrywise-square of the dominant left-singular-vector: i.e., $\vec{u}^{\cdot 2}$. Similarly, the d-score $\left[Z^d_{\text{COL}}\right]$ converges in direction to the entrywise-square of the dominant right-singular vector: i.e., $\vec{v}^{\cdot 2}$. These scores are identical to the scores used in Step-2 of the simple spectral algorithm.

In the next two sections we argue that the size-threshold and noise-threshold associated with this simple spectral method can't be much better than the detection-thresholds associated with our loop-counting algorithm. Later, in section 22.3, we refine this argument to examine the behavior of the spectral method near the detection-threshold, explaining some of the results shown in Figs 29,30 and 31.

22.1 Size-threshold:

At this point we can use a simple counting argument to estimate how effective these scores $\left[Z^d_{\text{ROW}}\right]$ might be when it comes to detecting a planted bicluster. This counting argument is the first stage of the commonly-used 'moment-method' for analyzing the distribution of eigenvalues in a random matrix [15]. While we strongly encourage the interested reader to pursue this reference, we provide the basics of this argument here.

For now let's focus on the noiseless limit, and say that the $m \times m$ planted bicluster B is perfectly rank-1, and involves the row-subset J_B and column-subset K_B. Let's refer to the matrix-entries of D that are not in B as B^{C}. We'll also assume that d is fixed and that M and m are very large, with $M \gg m \gg d$; we'll pay attention to the change in behavior that occurs when $m \sim \sqrt{M}$. When we refer to quantity such as $O\left(M^{2d+1}\right)$ we'll ignore terms that do not grow like a power of m or M (e.g., we'll ignore terms of order d, e^d, and so forth). To ease the notation, let's define $\delta = (d-1)/2$; we'll later use δ to describe paths outside of B that contribute a $\pi(p)$ of $+1$.

Any $(d+1) \times 2$-step path $p[J;K]$ will step on up to $2(d+1)$ different matrix-entries within D. For a given path p, some of its matrix-entries may be traversed only once, but others may be stepped on multiple times (i.e., they may be traversed with multiplicity greater than 1). Paths p fall into one of several categories, depending on the unique matrix-entries in p and their multiplicities. The essential idea behind our argument will be that, in order for a path p to contribute positively to the row-score, its matrix-entries from B^{C} must be multiplicity-2, but its matrix-entries from B need only be multiplicity-1.

Category-D0: This category includes paths p that start at some $\hat{\jmath} \notin J_B$ and remain outside of B, while also having some matrix-entries with odd multiplicity. These paths will have a product $\pi(p[J;K])$ that is drawn from the uniform distribution on $\{-1,+1\}$. Given a fixed $\hat{\jmath} \notin J_B$, there are $O\left(M^d \cdot M^{d+1}\right)$ such paths. We arrive at this estimate by noting that there are $\sim M$ choices for each of the d rows $j^{[1]},\ldots,j^{[d]}$, and $\sim M$ choices for each of the $(d+1)$ columns $k^{[0]},k^{[1]},\ldots,k^{[d]}$. These Category-D0 paths each contribute a term of type $\pi(p) = \pm 1$ to the score $\left[Z^d_{\text{ROW}}\right]_{\hat{\jmath}}$. When averaging over all possible configurations of D, the total contribution of these Category-D0 paths will be 0. If these paths were all independent from one another, their total contribution would have a variance close to $M^d \cdot M^{d+1}$. Because the category-D0 paths are correlated, the variance of their total contribution is even larger, but is still is proportional to $M^d \cdot M^{d+1}$ multiplied by a prefactor of the form $[\text{constant}]^d$. The exact value of this prefactor is not as relevant as its magnitude, which grows with d but does not depend on M for fixed d. Thus, with respect to m and M, the variance of the total contribution of the Category-D0 paths is $O\left(M^d \cdot M^{d+1}\right)$.

Category-D: This category includes paths p that start at some $\hat{\jmath} \notin J_B$ and remain outside of B, while only having matrix-entries with even multiplicity. These paths will have a product $\pi(p) = +1$. For a given $\hat{\jmath} \notin J_B$ there will be $O\left(M^\delta \cdot M^{\delta+1}\right)$ paths available. We arrive at this estimate by noting that each matrix-entry must be traversed an even number of times (e.g., not once, but 0, 2, 4, ... times): there can be at most δ unique rows amongst $j^{[1]},\ldots,j^{[d]}$, and at most $\delta+1$ unique columns amongst $k^{[0]},k^{[1]},\ldots,k^{[d]}$. A path p with the maximum number of unique rows and columns thus has $d+1 = 2\delta+2$ unique matrix-entries, starting out at $\left(\hat{\jmath},k^{[0]}\right)$, including $\left(j^{[s+1]},k^{[s]}\right)$ and $\left(j^{[s+1]},k^{[s+1]}\right)$ for $s \in 0,\ldots,\delta$, and potentially terminating at $\left(\hat{\jmath},k^{[\delta]}\right)$. Such a path has multiplicity-2 for each of its matrix-entries. When averaging over all possible configurations of D, the total contribution of these Category-D paths will be $O\left(M^\delta \cdot M^{\delta+1}\right)$; because $\delta \approx d/2$, this is bounded above in magnitude by the standard-deviation associated with the Category-D0 paths. Note that in this estimate we need not worry about other Category-D paths that involve fewer than δ unique rows and $\delta+1$ unique columns, because the quantity of these other paths is an order of magnitude lower than the multiplicity-2-paths just discussed.

Category-B: This category includes paths p that start at some $j \in J_B$ and remain inside B. These paths will necessarily step on each row and each column an even number of times (by construction), and will have a product $\pi(p) = +1$. Given a fixed $j \in J_B$, there will be $O\left(m^d \cdot m^{d+1}\right)$ such paths. We arrive at this estimate by noting that there are $\sim m$ choices for each of the d rows $j^{[1]},\ldots,j^{[d]}$, and $\sim m$ choices for each of the $(d+1)$ columns $k^{[0]},k^{[1]},\ldots,k^{[d]}$. The total contribution of these Category-B paths will be $O\left(m^d \cdot m^{d+1}\right)$.

Category-C0: This category includes paths p that include some matrix-entries in B, as well as some matrix entries outside of B (i.e., in B^{C}). Additionally, the paths p must each have at least one of the following properties: either (i) some of the matrix-entries outside of B have an odd multiplicity, and/or (ii) the collection of matrix-entries inside of B does not include each row in J_B an even number of times, and/or (iii) the collection of matrix-entries inside of B does not include each column of K_B an even number of times. These paths produce a product $\pi(p)$ which is drawn uniformly from $\{-1,+1\}$. There are many such paths, but their total number is bounded by $O\left(m \cdot M^{2d}\right)$ if the path p starts at some given $j \in J_B$ and by $O\left(m^2 \cdot M^{2d-1}\right)$ if the path starts at some given $\hat{\jmath} \notin J_B$, both of which are bounded by the number of Category-D0 paths. The reason there are so few of these paths is that at least one of the matrix-entries $\left(j^{[s]},k^{[s]}\right)$ or $\left(j^{[s+1]},k^{[s]}\right)$ must have a row drawn from J_B and a column drawn from K_B. Consequently, after averaging over all possible configurations of D, the total contribution of these paths to

the row-score will be 0, and the variance of this contribution will be bounded by the variance contributed by the Category-D0 paths.

Category-C: This category includes paths p that include some matrix-entries in B, as well as some matrix entries outside of B (i.e., in $B^{\complement}$). In addition, paths in Category-C must have all of the following properties: both (i) all of the matrix-entries in $B^{\complement}$ must have an even multiplicity, and (ii) the collection of matrix-entries inside of B must include each row of J_B and each column of K_B an even number of times. These paths will produce a product $\pi(p) = +1$. If a Category-C path p has $y < d$ unique matrix-entries from $B^{\complement}$, then the maximum number of unique matrix-entries it can have in total is $y + x$, where $x = 2d + 2 - 2y \geq 4$ denotes the number of matrix-entries coming from B. This is because each of the y matrix-entries will have multiplicity at least 2. Assuming the path starts out inside B, the very first step of the path corresponds to one unique row-index from J_B and one unique column-index from K_B. Because the path itself can be thought of as a cycle, the final step of the path does not involve any unique row- or column-indices. Moreover, each step along the path corresponds to at most one additional unique row-index from J_B or $J_{B^{\complement}}$, or one additional unique column-index from K_B or $K_{B^{\complement}}$. Because $2y$ steps along the path must be associated with the multiplicity-2 matrix-entries from $B^{\complement}$, the entire path can include at most x unique indices from J_B and K_B, and y unique indices from $J_{B^{\complement}}$ and $K_{B^{\complement}}$. Combining these observations we see that, for fixed x, y such that $y < d$ and $x + 2y = 2d + 2 - 2y$, if we require that the path p begins at a given row-index $j \in J_B$, then there will be at most $O\left(m^{x-1}M^{y}\right)$ such paths available. On the other hand, if we require that the path p begins at a given row-index $\hat{\jmath} \notin J_B$, then there will be only $O\left(m^{x}M^{y-1}\right)$ such paths available. A Category-C path p with exactly $y = d$ unique matrix-entries from $B^{\complement}$ will only have $x = 1$ unique matrix-entry from B (since p cannot satisfy propery (ii) if $x = 2$). Such a path has at most 2 unique matrix-entries from J_B and K_B, and $y - 1$ unique matrix-entries from $J_{B^{\complement}}$ and $K_{B^{\complement}}$. Combining these observations we see that, for fixed $x = 1$ and $y = d$, if we insist that the path p begins at a given row-index $j \in J_B$, then there will be at most $O\left(m^{1}M^{d-1}\right)$ such paths available. On the other hand, if we insist that the path p begins at a given row-index $\hat{\jmath} \notin J_B$, then there will be only $O\left(m^{2}M^{d-2}\right)$ such paths available. Note that, similar to the Category-D case, we can safely ignore Category-C paths that involve fewer than the maximum number of unique matrix-entries, as their total quantity will be an order of magnitude lower than the paths just discussed.

Given the categories above, we can fix d and analyze the behavior of the typical row-score $\left[Z^{d}_{\text{ROW}}\right]_{j}$ when $j \in J_B$, and compare it against the typical row-score $\left[Z^{d}_{\text{ROW}}\right]_{\hat{\jmath}}$ when $\hat{\jmath} \notin J_B$. We have two cases:

If $m = o(\sqrt{M})$: The most numerous Category-C paths will correspond to $y = d$ and $x = 1$, implying an average Category-C contribution of $O\left(m^{1}M^{d-1}\right)$ when $j \in J_B$, and $O\left(m^{2}M^{d-2}\right)$ when $\hat{\jmath} \notin J_B$. The Category-C and Category-B contributions combined will be negligible compared to the standard-deviation of the contribution from the Category-D0 and Category-C0 paths. The row-score won't be significantly different for J_B than for $J_{B^{\complement}}$.

If $m \gtrsim \sqrt{M}$: The most numerous Category-C paths will correspond to $y = 1$ and $x = 2d$, implying an average Category-C contribution of $O\left(m^{2d-1}M\right)$ when $j \in J_B$, and $O\left(m^{2d}\right)$ when $\hat{\jmath} \notin J_B$. The sum of the average Category-C and Category-B contributions will be an order of magnitude higher for $j \in J_B$ than for $\hat{\jmath} \notin J_B$. Moreover, these contributions dwarf the standard-deviation of the contribution from Category-D0 and Category-C0. The row-score will be significantly greater for J_B than for $J_{B^{\complement}}$.

In summary, if we fix d and take the limit as M and m go to infinity with $M \gg m \gg d$, then the typical row-score $\left[Z^{d}_{\text{ROW}}\right]_{j}$ be greater than the typical row-score $\left[Z^{d}_{\text{ROW}}\right]_{\hat{\jmath}}$ only when m is on the same order as (or larger than) $\sqrt{M}$. This is the same size-threshold that pertains to our loop-score (i.e., $d = 1$), and suggests that this size-threshold applies for all d, as well as for the simple spectral method above.

22.2 Noise-threshold:

If we assume that B is not perfectly rank-1, but instead only approximately rank-1 with error ε, then the rough calculation above changes slightly. The Category-D0 and Category-C0 paths still contribute a variance on the order of $O\left(M^{2d+1}\right)$ to the row-score $\left[Z^{d}_{\text{ROW}}\right]_{\hat{\jmath}}$ when $\hat{\jmath} \notin J_B$. On the other hand, the Category-B and Category-C paths now contribute a weaker signal to the row-scores $\left[Z^{d}_{\text{ROW}}\right]_{j}$ when $j \in J_B$. Looking at the Category-B paths for now, we see that there are still $O\left(m^{2d+1}\right)$ paths within B that start and end at row-j. However, the noise within B will flip the sign of some of the entries of B, and not all these paths will contribute a product $\pi(p) = +1$. These paths now fall into two sub-categories:

Category-B+: This category includes paths p in B that step on any perturbed entries (i.e., those with sign flipped by the noise) an even number of times in total. These paths will produce a $\pi(p) = +1$.

Category-B-: This category includes paths p in B that step on any perturbed entries an odd number of times in total. These paths will produce a $\pi(p) = -1$.

The fraction of Category-B paths that are in Category-B+ is some number $G^d_{1,\varepsilon,m}$ in between 1/2 and 1 (note that $G^1_{1,\varepsilon,m}$ is the same as the $g_{1,\varepsilon,m}$ we calculated in section 5). The average contribution of all the Category-B paths is now $O\left(\left(2G^d_{1,\varepsilon,m} - 1\right) \cdot m^{2d+1}\right)$, and we expect the average contribution of all the Category-C paths to suffer from a similar reduction. Given this reduction, we now expect the score $\left[Z^d_{\text{ROW}}\right]_j$ to be distinguishable from $\left[Z^d_{\text{ROW}}\right]_{\hat{j}}$ only when $G^d_{1,\varepsilon,m}$ is significantly greater than 1/2.

Recall that B is generated by first drawing the columns of a matrix A from a randomly-oriented gaussian-distribution ρ and then binarizing the result. The distribution ρ has a dominant principal-value equal to 1, and subsequent singular-values equal to ε (i.e., A is rank-1 with error-ε). The distribution of singular-values of ρ implies that only a fraction ϕ of the total variance of ρ is associated with its first principal component. This fraction ϕ is $\sim 1/\left(1+\varepsilon^2 m\right)$, and is only significantly greater than 0 when $g_{1,\varepsilon,m} > 1/2$ (i.e., when $\varepsilon\sqrt{m} \lesssim 1$). When $\varepsilon\sqrt{m} \gg 1$ and $g_{1,\varepsilon,m} \sim 1/2$, then the fraction ϕ will be close to 0, and we expect $G^d_{1,\varepsilon,m}$ to be close to 1/2 as well. These observations suggest that $G^d_{1,\varepsilon,m}$ cannot be much higher than $g_{1,\varepsilon,m}$, which would imply that – at best – the row-scores Z^d_{ROW} obey the same noise-transition as our original loop-score (i.e., $\varepsilon\sqrt{m} \lesssim 1$). Consequently, we expect the simple spectral method to obey the same noise-transition at $\varepsilon\sqrt{m} \lesssim 1$.

22.3 Behavior near the detection-threshold

Comparisions between our loop-score method and the simple spectral method can be seen in Fig 29, as well as Figs 30 and 31. Note that our loop-score algorithm outperforms the spectral method in many circumstances, especially when the planted-bicluster has a rank $l > 1$. It turns out that this phenomenon is related to the behavior of these two methods *near* the detection-threshold. More specifically, all our analysis so far has focused on distinguishing the $m = o(\sqrt{M})$ situation from the $m \gtrsim \sqrt{M}$ situation. To understand why loop-counting outperforms the simple spectral method we need to focus on the situation where m is on the *same* order as $\sqrt{M}$. In other words, we should assume that $m/\sqrt{M}$ is some constant close to 1.

For illustration, let's consider a planted-bicluster problem where the matrix D is of size $M = 768$ and the embedded bicluster B is of rank $l = 2$ and size $m = 39$ with $\varepsilon = 0$. These parameters are close to the $M = 1024$, $\log_M(m) = 0.55$ position in Fig 30 (i.e., a parameter regime where loop-counting outperforms the spectral method). For this choice of parameters m is quite close to $\sqrt{M}$: i.e., $m/\sqrt{M} \approx 1.4$. Hence, the phenomenon we see for these parameters will be indicative of what happens near the detection-threshold. In this example we'll focus on the row-scores, although our argument applies equally well to the column-scores.

Given this planted-bicluster problem, we construct multiple trials; for each trial we calculate the d-scores $\left[Z^d_{\text{ROW}}\right]$ for various d. In this situation we have no controls; there is no convenience associated with correcting for collapsed-loops, and so we'll use the formulae for the d-scores in section 22; these scores will always be positive. Going forward, it will be more convenient to refer to these scores by their square-roots, which we'll denote by $\left[z^d_{\text{ROW}}\right]$. In general, each individual $\left[z^d_{\text{ROW}}\right]_j$ will be drawn from one of two distinct distributions, depending on whether or not j is in J_B or $J_{B^\complement}$. We'll denote these two distributions by ζ^d_B and $\zeta^d_{B^\complement}$, respectively. These two distributions will depend on d, in addition to the parameters M, m, l and ε. Ultimately, we'll be interested how these two distributions change as a function of d, for m close to $\sqrt{M}$.

For the particular parameters in this example, we illustrate the distributions of the square-roots of the d-scores in Fig 90A. Each of these subplots shows the distributions ζ^d_B and $\zeta^d_{B^\complement}$ for a particular d (obtained by trial-averaging). The distribution ζ^d_B is shown in red, while the distribution $\zeta^d_{B^\complement}$ is shown in blue.

Note that, when $d = 1$, we recover our original loop-scores. Our analysis from section 5 applies almost perfectly; the loop-scores $\left[Z^1_{\text{ROW}}\right]$ are distributed roughly like gaussians with means much greater than 0 (recall that these scores include the contribution of collapsed-loops), and hence so too are their square-roots $\left[z^1_{\text{ROW}}\right]$. Consequently, the distributions ζ^1_B and $\zeta^1_{B^\complement}$ are also roughly gaussian. Moreover, when $d = 1$ we can use our expression for $g_{2,\varepsilon,m}$ to calculate the shift in the means of these two gaussian distributions.

As d increases, the two square-root-score-distributions ζ^d_B and $\zeta^d_{B^\complement}$ change shape; for this choice of parameters they become more and more lopsided. When d is very large the scores $[Z^\infty_{\text{ROW}}]$ converge to the scores of the simple spectral method: namely, the entrywise-squares of the dominant left-singular-vector of D (i.e., $\vec{u}^{\cdot 2}$). Consequently, the square-roots of the scores (i.e., $[z^\infty_{\text{ROW}}]_j$) converge to the absolute-values $|\vec{u}_j|$. For this choice of parameters, both ζ^∞_B and $\zeta^\infty_{B^\complement}$ are rather lopsided (with the latter equivalent to half a gaussian-distribution). It is the lopsidedness of ζ^∞_B that compromises the performance of the spectral method, and we'll discuss this more below.

One way to quantify the difference between the square-root-score-distributions is to measure the trial-averaged Auc obtained by comparing ζ^d_B to $\zeta^d_{B^\complement}$ (shown in each panel). Note that the Auc is somewhat high for the original loop-scores

(i.e., $d = 1$), increases as a function of d up until a point, and then decreases as d gets larger and larger. The behavior of the trial-averaged Auc as a function of d is shown in Fig 90B (thick black line).

If we were to calculate our scores only once and use a linear threshold to distinguish rows inside B from rows outside B, then the Auc we just described would dictate the success of our methodology. However, our algorithm is iterative; we typically remove a small fraction of the rows and columns of D with each iteration. As mentioned in Fig 32, this fraction is usually $\lesssim 5\%$. Our hope is that – by removing the rows and columns associated with the lowest scores – we preferentially remove rows and columns from outside B, leaving the rows and columns of B mostly untouched. This strategy will be successful only if the lowest scores are more likely to come from outside B than from inside B. Thus, with regards to our iterative framework, a more revealing measure of the quality of the various d-score-distributions is the Auc obtained by comparing the bottom 5th-percentile of row-scores in B with the bottom 5th-percentile of row-scores outside B [8]. This trial-averaged metric – referred to as 'A05' – is shown in Fig 90C (thick black line).

Note that both the Auc and the A05 are markedly higher when d is small than when d is very large. This fact is a direct result of the lopsided nature of ζ_B^d for large d, and is the reason why our loop-counting algorithm outperforms the simple spectral method in this example. We now turn to a discussion of this lopsidedness.

Recall that the square-root-scores $[z_{\text{ROW}}^{\infty}]$ associated with the simple spectral method are drawn from ζ_B^{∞} and $\zeta_{B^{\complement}}^{\infty}$, which describe the distribution of the coefficients of the vector $|\vec{u}|$. The distribution of these coefficients can in turn be determined analytically by using the techniques of [$\mathcal{M}$32].

Before we engage in this analysis, we make the following observations:

1. The singular-vector $\vec{u}$ is equal to the dominant eigenvector of the covariance-matrix $DD^{\intercal}/M$.

2. The covariance-matrix $DD^{\intercal}/M$ can be approximated by the sum of (a) a random covariance matrix W and (b) a symmetric matrix C with the following block structure: C is mostly 0, except for an $m \times m$ block corresponding to J_B with off-diagonal entries roughly proportional to $\pm m/(2M\sqrt{\max(1/2, l-1)})$. See section 18 for a derivation. In this particular example, because $l = 2$, the nonzero off-diagonal entries of C are close to $\pm m/(2M)$.

3. The block matrix C is rank-1 with a dominant eigenvalue roughly equal to $\sim m^2/(2M\sqrt{\max(1/2, l-1)})$.

4. The dominant eigenvector $\vec{\psi}$ of C shares the block-structure of C: The m entries of ψ corresponding to J_B are each equal to $\pm 1/\sqrt{m}$, while the other $M - m$ entries of ψ are 0.

At this point we can adopt the approach of [$\mathcal{M}$32], which observes that the dominant eigenvector $\vec{u}$ of $DD^{\intercal}/M$ obeys an implicit equation involving the entries of $\vec{\psi}$ and $\vec{u}$ itself, as well as the empirical spectral distribution of W (which is, in this case, given by a Marchenko-Pastur law with aspect-ratio 1.0). Following this train of logic, one can show that – depending on m – the vector $\vec{u}$ may or may not point in the direction of $\vec{\psi}$. More specifically, if we define ω to be the angle between $\vec{u}$ and $\vec{\psi}$, one can show that $\cos(\omega)^2$ can be approximated by:

$$\cos(\omega)^2 \approx \max\left\{0\ ,\ 1 - \frac{M}{m^2}2\sqrt{\max(1/2, l-1)}\right\}, \tag{3}$$

with this approximation holding with high probability in the limit as $m, M \to \infty$ (with $m/\sqrt{M}$ fixed). Note also that, aside from the restriction that $\vec{u} \cdot \vec{\psi} = \cos(\omega)$, the vector $\vec{u}$ is drawn from a uniform distribution on the sphere in $\mathbb{R}^M$.

We illustrate this phenomenon with a numerical experiment in Fig 90D. For this experiment we fix $M = 768$, $l = 2$ and $\varepsilon = 0$. We then vary m; for each m we construct multiple trials and measure ω. The trial-averaged $\cos(\omega)^2$ is plotted in black, while the analytical approximation to $\cos(\omega)^2$ from Eq. 3 is shown in grey.

The value of ω can be used to determine the distribution of $\vec{u}$ on $\mathbb{R}^M$: $\vec{u}$ is drawn uniformly from the set of vectors that are of unit norm and have $\vec{\psi}$-component equal to $\cos(\omega)$ (i.e., $\vec{u}$ is drawn from a slightly smaller sphere of dimension $M - 1$ centered at $\cos(\omega) \cdot \vec{\psi}$ and normal to $\vec{\psi}$). This distribution on $\vec{u}$ in turn determines the two distributions ζ_B^{∞} and $\zeta_{B^{\complement}}^{\infty}$.

In general, the distribution $\zeta_{B^{\complement}}^{\infty}$ will be a half-gaussian; these square-root-scores are associated with the component of $\vec{u}$ that is randomly oriented. On the other hand, the distribution ζ_B^{∞} may or may not look like $\zeta_{B^{\complement}}^{\infty}$. If m is too low, then $\cos(\omega)$ will be essentially 0, and the distribution ζ_B^{∞} will look essentially like $\zeta_{B^{\complement}}^{\infty}$. But, if m is large then $\cos(\omega)$ will be large, and the distribution ζ_B^{∞} will be very different from $\zeta_{B^{\complement}}^{\infty}$.

Coming back to our example, we have $m = 39$ and $m/\sqrt{M} \approx 1.4$; the trial-averaged $\cos(\omega)$ is approximately 0.19. If we use this value of ω to determine ζ_B^{∞} and $\zeta_{B^{\complement}}^{\infty}$ as described above (i.e., drawing $\vec{u}$ from the appropriate sphere), we obtain the grey lines shown in the background of the spectral square-root-scores in Fig 90A. Note that this approximation is very close to the trial-averaged distributions shown in blue and red.

Taking a step back from our example we can immediately see that, if we were to increase m, then $\cos(\omega)$ would increase as well, and $\vec{u}$ would align more closely with $\vec{\psi}$. As $\vec{u}$ and $\vec{\psi}$ become more aligned, the distribution ζ_B^{∞} moves to the right,

[8] for this example, this A05 is obtained by comparing the bottom 2 scores in B with the bottom 36 scores outside B

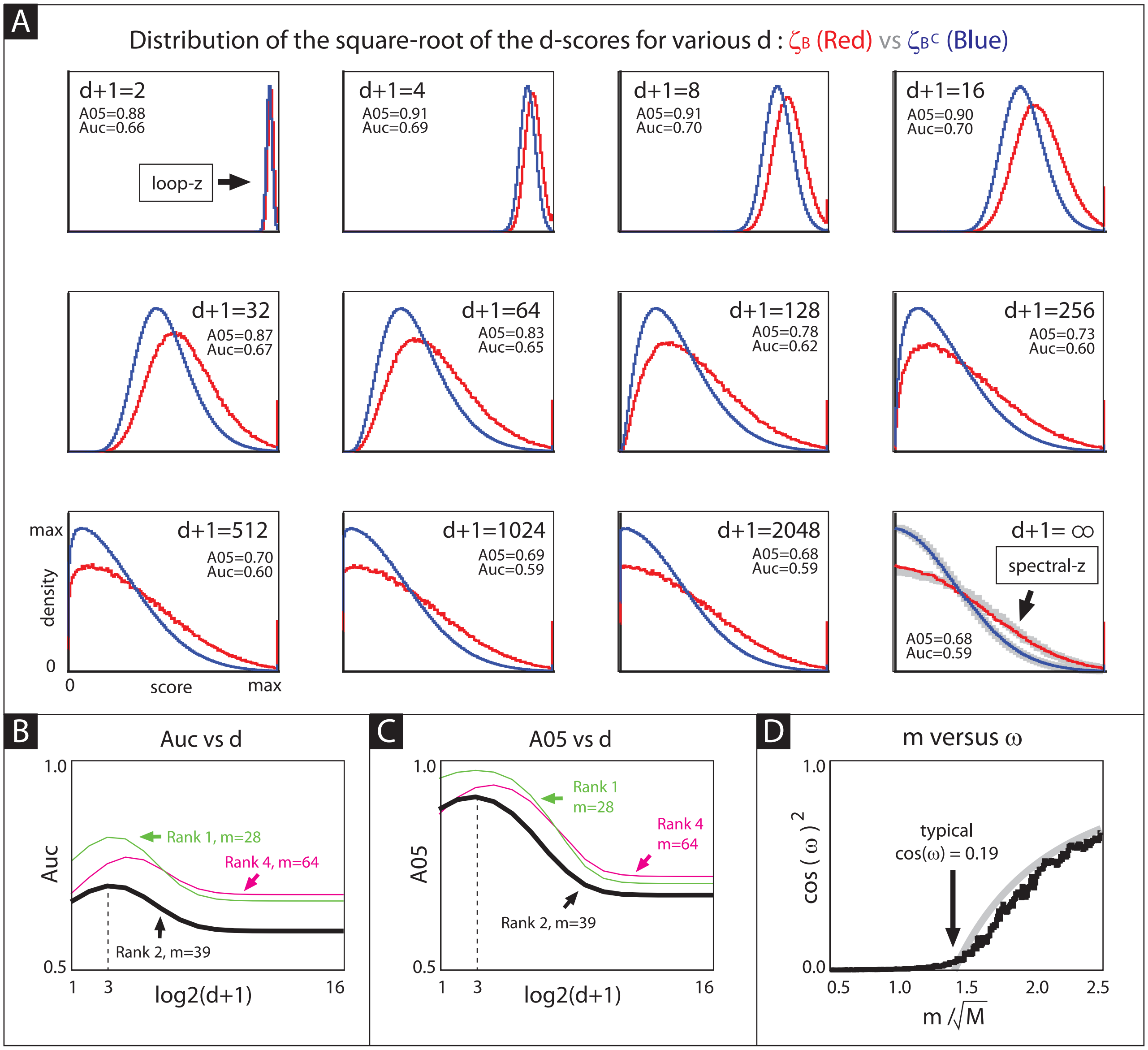

Figure 90: Illustration of square-roots of the d-scores for the planted-bicluster problem with $M = 768$, $l = 2$, $m = 39$, and $\varepsilon = 0$. Panel-A shows the trial-averaged distribution of the square-roots of the d-scores both within (red) and outside (blue) the planted bicluster B. Panel-B shows the trial-averaged Auc obtained by comparing all scores in B to all scores outside B (thick black line). Panel-C shows the trial-averaged Auc obtained by comparing the bottom 5th percentile of scores in B to the bottom 5th-percentile of scores outside B (thick black line). Panel-D shows the relationship between $m/\sqrt{M}$ and the typical angle ω made by the dominant left singular-vector of the data-matrix D and the 'true' singular-vector associated with B.

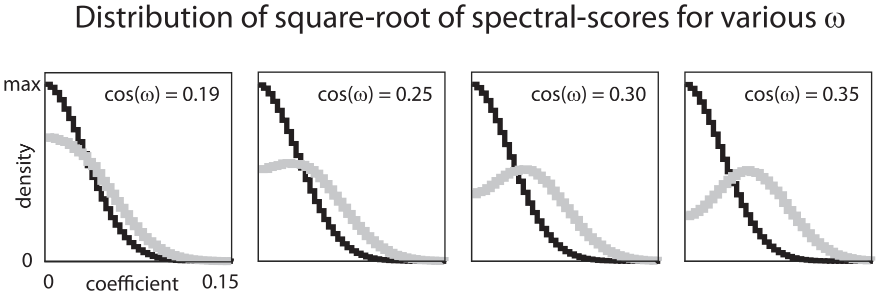

Figure 91: The spectral square-root-score-distributions associated with various ω, given $M = 768$, $l = 2$ and $\varepsilon = 0$. The distribution $\zeta^{\infty}_{B^{\complement}}$ is shown in black, and the distribution ζ^{∞}_{B} is shown in grey. See text for full description.

as shown in Fig 91. When $\cos(\omega)$ is sufficiently large, then ζ^{∞}_{B} is very well distinguished from $\zeta^{\infty}_{B^{\complement}}$, and the simple spectral method performs well.

At this point we can see that the value of $\cos(\omega)$, and hence the performance of the spectral method, is a function of the bicluster size m, as well as the bicluster rank l and error ε. For many choices of parameters, such as those shown in the example above, $\cos(\omega)$ will be small and the loop-scores will be more useful than the spectral-scores (at least within the context of our methodology). This phenomenon is particularly pronounced when the rank l of the planted-bicluster is greater than 1; in this situation $\cos(\omega)$ increases only gradually as a function of $m/\sqrt{M}$. On the other hand, when $l = 1$ then $\cos(\omega)$ increases rather quickly as a function of $m/\sqrt{M}$, and this phenomenon is not as pronounced. Indeed, for some choices of parameters (e.g., if $l = 1$ and m and ε are sufficiently large) then it is possible for the spectral-scores to be more useful than our loop-scores (see, e.g., label 'E' near the detection boundary in Fig 29C).

Overall, we believe that the loop-scores are a better choice than the spectral-scores. Not only are the loop-scores typically more useful with regards to the planted-bicluster problem (as discussed above), but the loop-scores are also less sensitive to certain kinds of spectral noise.

We close by highlighting two more planted-bicluster problems identical to the example discussed above, but with different values for m and l. The first involves $m = 28$, $l = 1$, and the second involves $m = 64$, $l = 4$. The behavior of the distributions ζ^{∞}_{B} and $\zeta^{\infty}_{B^{\complement}}$ for these problems is qualitatively similar to the behavior shown in Fig 90A, but the separation between the two distributions depends on the details. The trial-averaged Auc and A05 for these two problems is shown in Figs 90B,C using magenta and green lines in the background. Note that the general trend observed in the $m = 39$, $l = 2$ case still holds: the spectral scores are less useful than the loop-scores. Also note that the peak values for the Auc and the A05 occur at different values of d, depending on the problem parameters.

22.4 A remark regarding message-passing algorithms

As readily observed in the examples above, the difference between the two distributions ζ^{d}_{B} and $\zeta^{d}_{B^{\complement}}$ depends on d. We've quantified this difference using the metrics Auc and A05 (which are easy to measure in a numerical experiment), but one could imagine a more comprehensive measurement of the information $\mathcal{D}_{KL}$ (i.e., relative-entropy) between these two distributions. Given what we've seen so far, it seems likely that the 'best' d-score for any particular planted-bicluster problem is neither the loop-score (i.e., $d = 1$) nor the spectral-score (i.e., $d = \infty$), but rather somewhere in the middle.

Taking this reasoning a step further, it is tempting to try and construct some nonlinear function of the 'best' d-score which allows for the 'optimal' classification of J_B versus $J_{B^{\complement}}$. Going even further, one might imagine a score that is itself constructed nonlinearly; instead of building the d-score $\left[Z^{d}_{\text{ROW}}\right]$ by applying power-iteration to the covariance-matrix $DD^{\intercal}$, one might try and build an even better score by applying a nonlinearity in between each stage of the power-iteration.

The message-passing algorithms of [$\mathcal{M}$33], [$\mathcal{M}$19], and [$\mathcal{M}$41] proceed along these lines. Generally speaking, these algorithms choose an appropriate nonlinearity to apply between stages of a 'message-passing procedure' similar to power-iteration. By choosing this nonlinearity carefully, these methods can significantly reduce their detection-thresholds for a variety of problems very similar to our planted-bicluster problem (such as, e.g., the planted-clique problem). Given the success of the message-passing algorithms of [$\mathcal{M}$33], [$\mathcal{M}$19], and [$\mathcal{M}$41], it seems certain that there exists a message-passing algorithm that outperforms our loop-counting algorithm when applied to the planted-bicluster problem; we fully intend to pursue this line of research in the future.

We remark that, when developing a message-passing algorithm, it is critical to ensure that the algorithm performs robustly even outside the framework of the planted-bicluster problem. That is to say, it is important to ensure that the message-passing procedure (and the choice of nonlinearities) is not too specific to the assumptions of the planted-bicluster problem, and that the algorithm generalizes to the kinds of scenarios seen in real data sets.

For example, we've already seen that the 'best' d-score for the planted-bicluster problem depends strongly on the problem parameters. More specifically, the 'best' d depends not only on the size and spectrum of the planted bicluster

B, but also on the structure of the rest of D. For example, if the spectrum of D were to decay, or if D were to contain a heterogeneous collection of other biclusters, then the distributions ζ^d_B and $\zeta^d_{B^\complement}$ will reflect this structure. Simply put: the optimal choice of d for one instance of the planted-bicluster problem may no longer be optimal once the parameters change, or when things get a little messier. When analyzing real data-sets we often have no knowledge of the size or spectrum of any hidden structures, and it is usually difficult to characterize the noise accurately. A good message-passing algorithm should perform well even in the absence of this information (perhaps adaptively tailoring its message-passing procedure as it analyzes the data).

22.5 Accounting for controls:

Much like our loop-counting methods, the simple spectral method described above can also be modified to account for several aspects of experimental design. In this subsection we'll elaborate on these techniques, discussing the control-correction in detail. In fact, as we'll see below, there are several ways of correcting the spectral-method which are quite similar to the corrections we've used for our biclustering methods (e.g., in sections 8 and 15.2).

Recall that, within Step-1 of the simple spectral method defined in section 22, we used $\vec{u}$ and $\vec{v}$ to refer to the first columns of U and V (respectively), where $D = U\Sigma V^\intercal$ is a singular-value-decomposition. Note that $\vec{u}$ is the dominant eigenvector of $DD^\intercal$, and $\vec{v}$ is the dominant eigenvector of $D^\intercal D$. We can view $\vec{u} \in \mathbb{R}^{M_D}$ as the direction of greatest variance for the columns of D, and $\vec{v} \in \mathbb{R}^N$ as the direction of greatest variance for the rows of D. We'll use this perspective below to generalize the simple spectral method.

To begin with, let's assume that – in addition to the $M_D \times N$ case-matrix D – we also have an $M_X \times N$ control-matrix X (as in section 8). Our goal will now be to find a direction $\vec{v}$ in $\mathbb{R}^N$ that best serves to separate these two categories. Given this goal, it is reasonable to consider different measures of separation 'S'.

One of the simplest such measures tries to find a $\vec{v}$ such that the projections '$D\vec{v}$' of the case-patients onto $\vec{v}$ are, collectively, as large (in magnitude) as possible, whereas the projections '$X\vec{v}$' of the control-patients onto $\vec{v}$ are as small as possible. This measure is given by:

$$S_1^{\text{col}}(\vec{v}; D, X) := \frac{1}{2}\frac{1}{NM_D}\sum_{j\in D}(D_{j:}\cdot\vec{v})^2 - \frac{1}{2}\frac{1}{NM_X}\sum_{j'\in X}(X_{j':}\cdot\vec{v})^2,$$

which aggregates the squares of each projected variable, adding $|D\vec{v}|^2$ and subtracting $|X\vec{v}|^2$. In this expression the vectors $D_{j:}$ and $X_{j:}$ are the jth rows of the D and X matrices, respectively. If we constrain the norm of $\vec{v}$ and maximize $S_1^{\text{col}}(\vec{v})$ we find that the optimal $\vec{v}$ is given by the dominant eigenvector of:

$$M_1^{\text{col}} := \frac{1}{NM_D}D^\intercal D - \frac{1}{NM_X}X^\intercal X. \tag{4}$$

Using this vector as $\vec{v}$ in Step-1 of the simple spectral method (instead of merely using the dominant eigenvector of $D^\intercal D$) allows us to correct for certain features of the control submatrix. Specifically, such a $\vec{v}$ would highlight columns k that participated in biclusters within D, but do not participate in any biclusters within X.

While such a control-correction is similar in many ways to our control-correction in section 8, it is not equivalent! To explain the difference, consider two cases of the planted-bicluster problem involving D and X of equal size (i.e., $M_D = M_X$). In case-1, we'll implant a $2m \times n$ bicluster $B_{[D;X]}$, with m rows in D and m rows in X. In case-2 we'll implant one $m \times n$ bicluster B_D in D, and another $m \times n$ bicluster B_X in X, with B_D and B_X occupying the same columns. Note that both case-1 and case-2 involve two $m \times n$ biclusters; one in D and one in X, both occupying the same columns. In case-1, both biclusters have the same low-rank structure (i.e., both are part of $B_{[D;X]}$), On the other hand, in case-2, the two biclusters have different (arbitrary) low-rank structure.

The control-corrected loop-counting algorithm described in section 8 will (correctly) ignore the case-specific component of $B_{[D;X]}$ in case-1, because $B_{[D;X]}$ straddles both D and X; it will also (correctly) find the bicluster B_D in case-2, because this bicluster is case-specific and possesses a low-rank structure which is distinct from the corresponding columns of X. On the other hand, the control-corrected spectral method derived from S_1^{col} and Eq. 4 will ignore both the case-specific component of $B_{[D;X]}$ in case-1, as well as the case-specific bicluster B_D in case-2. This is because the term $-X^\intercal X$ in M_1^{col} will penalize any column k in $\vec{v}$ whenever there is *any* low-rank structure within X involving k, regardless of whether or not that low-rank structure is the *same* in D as it is in X.

To rectify this deficiency, we could instead try to find a $\vec{v}$ such that the projection $D\vec{v}$ correlates with each column of D only when the projection $X\vec{v}$ is uncorrelated with the corresponding column of X (or vice-versa). This measure is given by:

$$S_2^{\text{col}}(\vec{v}; D, X) := \frac{1}{2}\frac{1}{N}\sum_k\left(\frac{1}{M_D}D_{k:}^\intercal D\cdot\vec{v} - \frac{1}{M_X}X_{k:}^\intercal X\cdot\vec{v}\right)^2.$$

If, as before, we constrain the norm of $\vec{v}$ and maximize $S_2^{\text{col}}(\vec{v})$, we find that the optimal $\vec{v}$ is given by the dominant eigenvector of:

$$M_2^{\text{col}} := \frac{1}{N}\left[\frac{1}{M_D M_D}D^\intercal D D^\intercal D - \frac{1}{M_D M_X}D^\intercal D X^\intercal X - \frac{1}{M_X M_D}X^\intercal X D^\intercal D + \frac{1}{M_X M_X}X^\intercal X X^\intercal X\right]. \quad (5)$$

Each term of M_2^{col} can be interpreted as affecting $\vec{v}$ in a different way. The first term $D^\intercal D D^\intercal D$ rewards columns that participate in biclusters within D. The two middle-terms $D^\intercal D X^\intercal X$ and $X^\intercal X D^\intercal D$ penalize columns that participate in nonspecific biclusters that exhibit the same low-rank structure across both D and X. The fourth term $X^\intercal X X^\intercal X$ rewards columns that participate in biclusters within X.

In the above discussion we focused on finding $\vec{v}$; Now we turn to finding $\vec{u}$. Taking inspiration from S_1^{col} and S_2^{col}, we can devise a strategy for finding $\vec{u} \in \mathbb{R}^{M_D}$: we can try and ensure that the projection $D^\intercal \vec{u}$ correlates strongly (either positively or negatively) with each row of D, while not correlating as strongly with the rows of X. This measure is given by:

$$S_2^{\text{row}}(\vec{u}; D, X) := \frac{1}{2}\frac{1}{N^2 M_D}\sum_{j\in D}\left(D_{j:}D^\intercal \cdot \vec{u}\right)^2 - \frac{1}{2}\frac{1}{N^2 M_X}\sum_{j'\in X}\left(X_{j':}D^\intercal \cdot \vec{u}\right)^2.$$

As before, we constrain the norm of $\vec{u}$ and maximize $S_2^{\text{row}}(\vec{u})$; the optimal $\vec{u}$ is given by the dominant eigenvector of:

$$M_2^{\text{row}} := \frac{1}{N^2 M_D}DD^\intercal DD^\intercal - \frac{1}{N^2 M_X}DX^\intercal XD^\intercal. \quad (6)$$

Each term of M_2^{row} affects $\vec{u}$ in a different way; the first term $DD^\intercal DD^\intercal$ rewards rows that participate in biclusters within D, whereas the second term $DX^\intercal XD^\intercal$ penalizes rows that participate in nonspecific biclusters that exhibit the same low-rank structure across both D and X.

Note that the control-correction we use in our loop-counting algorithm (see section 8) is quite closely related to maximizing S_2^{row} and S_2^{col}. Indeed, our control-corrected row-score essentially involves calculating M_2^{row}, and our control-corrected column-score essentially involves calculating the first term of M_2^{col}, as well as one of the two middle terms. We don't use the fourth term of M_2^{col}, as our goal in section 8 is to find case-specific biclusters, not to avoid control-specific biclusters (unless, of course, they are part of non-specific biclusters, which will be penalized by either of the middle-terms).

Other measures of case-control separation: Note that S_1^{col}, S_2^{col} and S_2^{row} are only a few of the many possible measures of 'separation'. For example, we could try and ensure that the average value of $D\vec{v}$ was different than the average value of $X\vec{v}$, defining a separation:

$$S_3^{\text{col}} := \frac{1}{2}\left(\frac{1}{M_D}\sum_{j\in D}D_{j,:}\vec{v} - \frac{1}{M_X}\sum_{j'\in X}X_{j':,}\vec{v}\right)^2,$$

which has an optimal $\vec{v}$ given by the dominant eigenvector of:

$$M_3^{\text{col}} := \left(\frac{1}{M_D}D^\intercal 1_D - \frac{1}{M_X}X^\intercal 1_X\right)\left(\frac{1}{M_D}1_D^\intercal D - \frac{1}{M_X}1_X^\intercal X\right),$$

where 1_D is the $M_D \times 1$ vector of all ones, and 1_X is the $M_X \times 1$ vector of all ones.

We could also try and ensure a large magnitude difference between the projected case-rows $D_{j,:}\vec{v}$ and control-rows $X_{j':}\vec{v}$, defining:

$$S_4^{\text{col}} := \frac{1}{2}\frac{1}{M_D M_X}\sum_{j\in D,\ j'\in X}\left(D_{j,:}\vec{v} - X_{j',:}\vec{v}\right)^2,$$

which has an optimal $\vec{v}$ given by the dominant eigenvector of:

$$M_4^{\text{col}} := \frac{1}{M_D}D^\intercal\left(D - \frac{1}{M_X}1_D 1_X^\intercal X\right) + \frac{1}{M_X}X^\intercal\left(X - \frac{1}{M_D}1_X 1_D^\intercal D\right).$$

Each of these different strategies has its advantages and disadvantages; focusing on certain kinds of structures and ignoring others. While no particular strategy is 'best' overall, there will be certain applications where some strategies outperform others. Note that, within the context of biclustering, the control-correction we use in our 'half-loop' algorithm (see section 15.2) is somewhat related to maximizing S_4^{col} for $\vec{v}$ and its analog S_4^{row} for $\vec{u}$.

Continuous case-control status: Finally, we discuss how to extend the methodology above to deal with continuous case-control status. Such a situation may arise when the categorization of patients into 'cases' and 'controls' isn't all that

clear-cut. For example, we might imagine that our data includes N measurements taken across M total patients, each of which has a different blood-pressure $\kappa_j \in \mathbb{R}$ (i.e., an associated continous clinical variable).

If we are interested in finding a direction $\vec{v} \in \mathbb{R}^N$ which separates high-blood-pressure patients from low-blood-pressure patients, we could pick some threshold $\bar{\kappa}$ and divide the patients into case- and control-groups based on whether their blood-pressure κ_j was greater or less than $\bar{\kappa}$. This simple thresholding might be sufficient in some cases, but relies on an (arbitrary) threshold $\bar{\kappa}$.

Alternatively, we can treat κ_j as a fully continuous variable, and try to find a $\vec{v} \in \mathbb{R}^N$ that maximizes the following separation:

$$S_5^{\text{col}} := \frac{1}{2}\sum_{j,j'} \left(D_{j,:}\vec{v} - D_{j',:}\vec{v}\right)^2 \left(\kappa_j - \kappa_{j'}\right)^2 . \tag{7}$$

Note that if $\vec{\kappa}$ is indeed binary (e.g., each κ_j takes on values of 0 or 1), then this S_5^{col} is essentially the same thing as the S_4^{col} above. If we go about our usual business (constraining the norm of $\vec{v}$ and optimizing S_5^{col}), then we find that $\vec{v}$ is an eigenvector of:

$$M_5^{\text{col}} := MD^{\intercal}K^2D - \left(1^{\intercal}\vec{\kappa}\right)2D^{\intercal}KD + \left(1^{\intercal}\vec{\kappa}^2\right)D^{\intercal}D - D^{\intercal}K^2 11^{\intercal}D + 2D^{\intercal}K11^{\intercal}KD - D^{\intercal}11^{\intercal}K^2D, \tag{8}$$

where K is the diagonal matrix with $K_{jj} = \kappa_j$, and 1 is the $M \times 1$ vector of all ones.

It is also possible that each patient has a multi-dimensional continuous-category $\vec{\kappa}_j \in \mathbb{R}^t$ (e.g., a vector of t-values including blood-pressure, weight, etc). We can immediately extend the methodology above using a slightly modified separation:

$$S_6^{\text{col}} := \frac{1}{2}\sum_{j,j',t} \left(D_{j,:}\vec{v} - D_{j',:}\vec{v}\right)^2 \left(\kappa_{j,t} - \kappa_{j',t}\right)^2 , \tag{9}$$

where κ_{jt} is the t-component of $\vec{\kappa}_j$ for the jth-data-point. Optimizing this objective involves finding the dominant eigenvector of :

$$M_6^{\text{col}} = \sum_t MD^{\intercal}K_t^2D - 2\left(1^{\intercal}\vec{\kappa}_t\right)D^{\intercal}K_tD + \left(1^{\intercal}\vec{\kappa}_t^2\right)D^{\intercal}D - D^{\intercal}K_t^2 11^{\intercal}D + 2D^{\intercal}K_t11^{\intercal}K_tD - D^{\intercal}11^{\intercal}K_t^2D, \tag{10}$$

where $\kappa_t \in \mathbb{R}^M$ is the vector formed from the κ_{jt} for various j, and K_t is the diagonal matrix such that $[K_t]_{jj} = \kappa_{jt}$.

As we've seen earlier, there are control-corrected spectral methods that are quite similar to our control-corrected biclustering algorithm (compare, e.g., Eqs. 5,6 with the loop-score of section 8). By analogy, it seems likely that Eqs. 8,10 can be used to extend our biclustering methodology to account for continuous case-control status. We intend to pursue this line of research in the future.

References

[1] Cunha S.I., Bocci M., Lovrot J., Eleftheriou N., Roswall P., Cordero E., Lindstrom L., Bartoschek M., Haller B.K., Pearsall R.S., Mulivor A.W., Kumar R., Larsson C., Bergh J. and Pietras K. Endothelial ALK1 Is a Therapeutic Target to Block Metastatic Dissemination of Breast Cancer. *Cancer Res.* 75(12):2445-56 (2015).

[2] Smith J.J., Dean N.G., Wu F., Merchant N.B., Zhang B., Jiang A., Lu P., Johnson J.C., Schmidt C., Bailer C.E., Eschrich S., Kis C., Levy S., Washington M.K., Heslin M.J., Coffey R.J., Yeatman T.J., Shyr Y. and Beauchamp R.D. Experimentally Derived Metastasis Gene Expression Profile Predicts Recurrence and Death in Patients with Colon Cancer. *Gastroenterology.* 138:958-968 (2009).

[3] Eschrich S., Yang I., Bloom G., Kwong K.Y., Boulware D., Cantor A., Coppola D., Kruhoffer M., Aaltonen L., Orntoft T.F., Quackenbush J. and Yeatman T.J. Molecular staging for survival prediction of colorectal cancer patients. *Journal of Clinical Oncology*, 23:15. (2005)

[4] Mulcahy H.E. and O'Donoghue D.P. Duration of colorectal cancer symptoms and survival: the effect of confounding clinical and pathological variables. *Eur J Cancer.* 33(9):146177. (1997)

[5] `https:www.ncbi.nlm.nih.govbooksNBK22218`

[6] Abstracts of the XXIIIrd World Congress of Psychiatric Genetics (WCPG): Final symposia and plenary abstracts. *European Neuropsychopharmacology.* (2015).

[7] Sklar P., Ripke S., Scott L.J., et al. Large-scale genome-wide association analysis of bipolar disorder identifies a new susceptibility locus near ODZ4. *Nature Genetics.* 43(10):977-983. doi:10.1038/ng.943. (2011)

[8] Stahl E., Forstner A., McQuillin A., Ripke S., the Bipolar Disorders Working Group of the PGC, Ophoff R., Scott L., Cichon S., Andreassen O., Sklar P., Kelsoe J. and Breen G., Genomewide association study identifies 30 loci associated with bipolar disorder. *bioRxiv.* `doi = 10.1101173062`, `http:www.biorxiv.orgcontentearly20170808173062.1.full.pdf`, (2017)

some affinity propagation method used to cluster columns and rows ; In this case biclusters are 'rank-0' ;

[9] Kaiser S., Santamaria R., Khamiakova T., Sill M., Theron R., Quintales L., Leisch F. and De Troyer E. BiCluster Algorithms (biclust) version 1.2.0. Date 2015-05-08 *Repository CRAN Repository/R-Forge/Project biclust Repository/R-Forge/Revision 225* 2015-05-12

[10] Prelic A., Bleuler S., Zimmermann P., Wil A., Buhlmann P., Gruissem W., Hennig L., Thiele L. and Zitzler E. A Systematic Comparison and Evaluation of Biclustering Methods for Gene Expression Data Bioinformatics. *Oxford Univ Press*, 22, 1122-1129 (2006)

[11] Cheng Y., and Church G.M. Biclustering of Expression. *Data Proceedings of the Eighth International Conference on Intelligent Systems for Molecular Biology*, 1, 93-103 (2000)

[12] Murali T., and Kasif S. Extracting Conserved Gene Expression Motifs from Gene Expression Data. *Pacific Symposium on Biocomputing*, 77-88 (2003)

[13] Li G., Ma Q., Tang H., Paterson A.H. and Xu Y. QUBIC: a qualitative biclustering algorithm for analyses of gene expression data. *Nucleic Acids Res.* Aug;37(15):e101. doi: 10.1093/nar/gkp491. Epub 2009 Jun 9. (2009)

[14] Wang Z., Li G., Robinson R.W. and Huang X. UniBic: Sequential row-based biclustering algorithm for analysis of gene expression data. *Nature* Scientific Reports 6, Article number: 23466. doi:10.1038/srep23466 (2016)

[15] Wigner E.P. Characteristic vectors of bordered matrices with infinite dimensions. *The Annals of Mathematics*, 62(3):548-564, (1955)
